# *Plasmodium falciparum* Erythrocyte Membrane Protein 1 Diversity in Seven Genomes – Divide and Conquer

**DOI:** 10.1371/journal.pcbi.1000933

**Published:** 2010-09-16

**Authors:** Thomas S. Rask, Daniel A. Hansen, Thor G. Theander, Anders Gorm Pedersen, Thomas Lavstsen

**Affiliations:** 1 Center for Biological Sequence Analysis, Department of Systems Biology, Technical University of Denmark, Lyngby, Denmark; 2 Centre for Medical Parasitology, Department of Medical Microbiology and Immunology, University of Copenhagen, Copehagen, Denmark; University of California Davis, United States of America

## Abstract

The *var* gene encoded hyper-variable *Plasmodium falciparum* erythrocyte membrane protein 1 (PfEMP1) family mediates cytoadhesion of infected erythrocytes to human endothelium. Antibodies blocking cytoadhesion are important mediators of malaria immunity acquired by endemic populations. The development of a PfEMP1 based vaccine mimicking natural acquired immunity depends on a thorough understanding of the evolved PfEMP1 diversity, balancing antigenic variation against conserved receptor binding affinities. This study redefines and reclassifies the domains of PfEMP1 from seven genomes. Analysis of domains in 399 different PfEMP1 sequences allowed identification of several novel domain classes, and a high degree of PfEMP1 domain compositional order, including conserved domain cassettes not always associated with the established group A–E division of PfEMP1. A novel iterative homology block (HB) detection method was applied, allowing identification of 628 conserved minimal PfEMP1 building blocks, describing on average 83% of a PfEMP1 sequence. Using the HBs, similarities between domain classes were determined, and Duffy binding-like (DBL) domain subclasses were found in many cases to be hybrids of major domain classes. Related to this, a recombination hotspot was uncovered between DBL subdomains S2 and S3. The VarDom server is introduced, from which information on domain classes and homology blocks can be retrieved, and new sequences can be classified. Several conserved sequence elements were found, including: (1) residues conserved in all DBL domains predicted to interact and hold together the three DBL subdomains, (2) potential integrin binding sites in DBLα domains, (3) an acylation motif conserved in group A *var* genes suggesting N-terminal N-myristoylation, (4) PfEMP1 inter-domain regions proposed to be elastic disordered structures, and (5) several conserved predicted phosphorylation sites. Ideally, this comprehensive categorization of PfEMP1 will provide a platform for future studies on *var*/PfEMP1 expression and function.

## Author Summary

About one million African children die from malaria every year. The severity of malaria infections in part depends on which type of the parasitic protein PfEMP1 is expressed on the surface of the infected red blood cells. Natural immunity to malaria is mediated through antibodies to PfEMP1. Therefore hopes for a malaria vaccine based on PfEMP1 proteins have been raised. However, the large sequence variation among PfEMP1 molecules has caused great difficulties in executing and interpreting studies on PfEMP1. Here, we present an extensive sequence analysis of all currently available PfEMP1 sequences and show that PfEMP1 variation is ordered and can be categorized at different levels. In this way, PfEMP1 belong to group A–E and are composed of up to four components, each component containing specific DBL or CIDR domain subclasses, which in some cases form entire conserved domain combinations. Finally, each PfEMP1 can be described in high detail as a combination of 628 homology blocks. This dissection of PfEMP1 diversity also enables predictions of several functional sequence motifs relevant to the fold of PfEMP1 proteins and their ability to bind human receptors. We therefore believe that this description of PfEMP1 diversity is necessary and helpful for the design and interpretation of future PfEMP1 studies.

## Introduction

*Plasmodium falciparum* erythrocyte membrane protein 1 (PfEMP1) mediates adhesion of infected erythrocytes (IE) to various host cells on the vascular lining, during the blood stage of malaria infection [1]–[2]. Naturally acquired protective antibodies in malaria-exposed individuals target PfEMP1, suggesting it is possible to develop PfEMP1 based vaccines [3]–[9].

The majority of the parasite's ∼60 PfEMP1-encoding *var* genes are situated in subtelomeric regions close to other variant antigen-encoding genes such as the *rif* and *stevor* gene families, while the remaining ∼40% are found centrally in the chromosomes. Based on sequence similarity, *var* 5′ UTR sequences can be divided into upstream sequence (UPS) classes A, B, C or E. These UPS classes correlate with chromosomal position of the genes, as well as domain complexity of the encoded PfEMP1 [10]–[11]. Subtelomeric UPSA and UPSB genes are oriented tail to tail (3′ to 3′), while central UPSC genes are oriented head to tail in a tandem repeat manner [12], which has lead to the definition of group A, B and C *var*/PfEMP1, and two intermediate groups B/A and B/C, that contain *var*/PfEMP1 with chromosomal position or domain composition different from that predicted from their UPS class. The hyper-variable *var* gene repertoire is to a large extent generated by frequent meiotic ectopic recombination in the mosquito abdomen, probably facilitated by alignment of *var* genes in the nuclear periphery [13]–[14]. There is also evidence suggesting that mitotic recombination occur, and that this allows further diversification of the *var* gene repertoire during human infection [15]. Comparison of the clones 3D7, IT4 and HB3 revealed only two *var* genes, *var1* and *var2csa*, that were conserved in all three genomes, and a semi-conserved gene, *var3*, found in IT4 and 3D7. The three conserved *var* genes are more than 75% identical over multiple domains, whereas most other PfEMP1 (even proteins with the same domain architecture) display less than 50% amino acid sequence identity between individual domains [16]. *Var2csa* is particularly unique as it has a unique UPSE, encodes unique Duffy binding-like (DBL) domains, as well as a distinct acidic terminal segment (ATS) [17].

Thus, parasite genomes appear to harbor essentially similar *var* repertoires, each reflecting the worldwide *var* diversity that has ensured the optimal survival of the parasite population. The clinical significance of the described *var* groups has been demonstrated in several studies, and indicates the existence of underlying functional differences in adhesion characteristics of the expressed PfEMP1 variants. This relationship is best illustrated by the malaria syndrome occurring in pregnant women, which is precipitated by the accumulation, in the placenta, of parasites expressing VAR2CSA that mediates binding to proteoglycans on syncytiotrophoblasts [17]–[21]. Several lines of evidence indicate that the relatively rapid development of immunity to severe childhood malaria is mediated through antibodies directed against a restricted semi-conserved subset of parasite antigens [22]–[23] that are associated with the development of severe disease [24]–[25]. In particular group A and to some extent group B *var* genes have been linked to disease severity in studies of expression of these variants in patients with symptomatic and asymptomatic infections [26]–[33]. A recent study has corroborated these findings and qualified which group A and B PfEMP1 variants may be associated with severe malaria disease, by demonstrating a sequential and ordered acquisition of antibodies to PfEMP1 domains in Tanzanian plasma donors [34].

**Figure 1:**
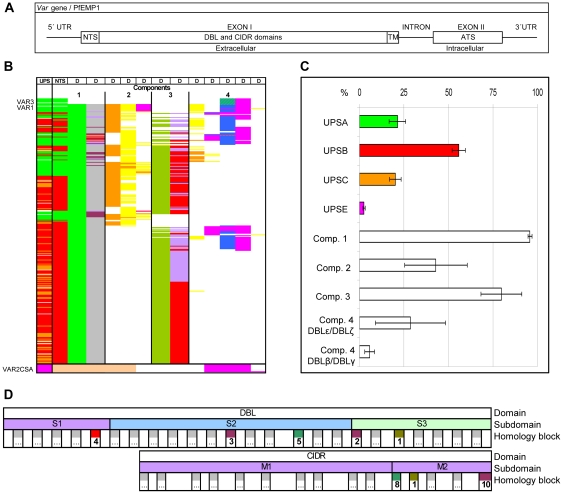
PfEMP1 annotation overview. (A) Schematic of the *var* gene locus. (B) 399 *var* exon1 annotated with UPS class and encoded major NTS, DBL and CIDR domain classes and their arrangement in four components. Color code for UPS column: Green: UPSA; Red: UPSB; Orange: UPSC; Pink: UPSE. Color code for NTS column: Green NTSA, Red: NTSB, Cream: NTSpam. Color code for DBL and CIDR domains (D columns): Bright Green: DBLα; Orange: DBLβ; Yellow: DBLγ; Olive green: DBLδ; Pink: DBLε; Blue: DBLζ; Blue stripes: DBLα of VAR3. Grey: CIDRα; Red: CIDRβ; Light purple: CIDRγ; Dark purple: CIDRδ. (C) Average distribution (% +/− 95% confidence intervals) of UPSA–E flanked and component 1–4 containing genes in the seven sequenced genomes 3D7, HB3, DD2, IT4, PFCLIN, RAJ116 and IGH. (D) Schematic presentation of DBL and CIDR subdomains and homology blocks. The numbered blocks represent the core homology blocks found in all DBL domains (HB2, 3, 4 and 5), all CIDR domains (HB8 and 10) or both domain types (HB1), further described in Figure 5.

In contrast to pregnancy malaria, it is still unclear which human receptor binding, if any particular, is linked to severe forms of childhood malaria. Parasite adhesion has been demonstrated to endothelial cells, immune system cells, uninfected erythrocytes and platelets. Several human cell receptors, including the extensively studied CD36 and intercellular adhesion molecule 1 (ICAM-1), have been implicated in adhesion, although no consensus on association between receptor binding and severe malaria has been reached (reviewed in [35]). PfEMP1 is responsible for parasite adhesion, as several single domains of the large multi-domain PfEMP1 molecules have been shown to bind human receptors. From N- to C-terminal, PfEMP1 has previously been described as composed of an N-terminal segment (NTS), Duffy binding-like (DBL) domains, Cys rich inter-domain regions (CIDR), C2 domains, one transmembrane region (TM) and the acidic terminal segment (ATS) (Figure 1A). Six major classes of DBL domains have been proposed based on amino acid sequence similarity: DBLα, β, γ, δ, ζ, and ε. DBL domains have been further characterized by definition of 10 semi-conserved homology blocks (HBa-j) interspersed by hyper-variable regions [36], and by definition of three structural subdomains (S1–3) [37] (Figure 1D). It has been shown that various DBLβ domains have affinity for ICAM-1 [38]–[40], whereas DBLδ adheres to platelet-endothelial cell adhesion molecule 1 (PECAM-1) and DBLα has been associated with binding to heparin sulfate (HS), blood group A antigen and complement receptor 1 (CR1) [41]–[42]. CR1 binding is associated with IE adhesion to uninfected erythrocytes, a phenomenon known as rosetting, which appears to be mediated to some degree by group A PfEMP1 [42]–[44].

CIDR domains have been divided into three classes: CIDRα, β, and γ [2], [10], [16], [36], and described as consisting of three regions, those being the minimal CD36 binding region denoted M2, flanked by less conserved M1 and M3 regions [36], [45]. Several CIDRα class domains have been found to mediate binding to the human CD36 receptor [1], [45]–[46], however, such binding is limited to group B and C PfEMP1, indicating that group A variants have a distinct function [47]. Furthermore, CIDRα domains have been found to bind immunoglobulin M and PECAM-1 [41].

Although it is evident that the organization of PfEMP1 sequence diversity is of relevance for malaria pathogenicity, the vast sequence variation of the protein family continues to impede experimental procedures and interpretations. In order to better understand and determine the potential targets for a PfEMP1-based vaccine against severe malaria, it is therefore essential to establish a rigorous classification and solid reference frame of PfEMP1 diversity.

In this work, PfEMP1 repertoires from seven genomes are annotated with updated domain boundary definitions. The data includes four thoroughly sequenced *P. falciparum* genomes that have not previously been classified: DD2 from Indochina (9.55× coverage), RAJ116 from India (7.3× coverage), IGH-CR14 from India (10.19× coverage), and the Ghanaian isolate PFCLIN (8× coverage). Domain architectures of 399 PfEMP1 are aligned, revealing conserved domain architectural features. The homology block concept, first described by Smith *et al.* (2000) [36], is extended from DBL domains to the entire PfEMP1 by application of a novel iterative homology search technique, defining 628 homology blocks covering on average 83% of any PfEMP1 with only 4% self-overlap. The homology blocks describe relations between sequences in finer detail than domains, revealing that domain subclasses often consist of fragments from different domain super-classes, probably as a result of extensive recombination. Evidence for a recombination hotspot is also found. The definition of conserved blocks in PfEMP1 allows identification of conserved functional elements, such as predicted sites for post-translational modifications, which may significantly affect both substrate binding and immune evasion.

## Results/Discussion

The *var* gene sequence analysis was based on two different bioinformatics approaches. First, phylogenetic trees were constructed using re-assessed PfEMP1 domain borders, with the aim of reclassifying and annotating the main PfEMP1 features UPS, NTS, DBL, CIDR and ATS. Secondly, a novel iterative homology detection method, defining a set of homology blocks, was used to describe domain similarities and to guide *var* gene recombination site and functional predictions.

### Grouping and componential composition of PfEMP1

In total 399 PfEMP1 sequences were annotated and their domains aligned. The alignments confirmed what recent studies of the DBL fold [48]–[49] and binding affinities [38] have implied; that the domain borders, by which PfEMP1 domain subclasses have been classified [36], needed revision. The redefined domain borders introduced by this study are specified in Text S1, and lead to two fundamental nomenclature changes: omitting the term “C2” from DBLβ domains, as also suggested in [39]; and the separation of M3 sequences from CIDR domains. Distance tree analysis of all DBL domains confirmed the expected phylogenetic grouping of DBL into six major classes (DBLα, β, γ, δ, ε, and ζ), as well as five smaller distinct classes (the four N-terminal DBL domains of VAR2CSA [10], and the DBLα of VAR3 which grouped in a separate cluster between DBLα and DBLζ). Five major CIDR domain classes were defined: CIDRα, β, γ, δ, and pam (Figure S1). The CIDRδ class has not previously been identified, probably due to the difference in sequence depth between this and previous CIDR classification (655 vs. 36) [36]. The inter-domain 2 (ID2) of VAR2CSA is partially homologous to CIDR domains [50], and was therefore included here as CIDRpam, although particularly different from other CIDR domains. NTS sequences were divided into three classes, NTSA, NTSB, and NTSpam (Figure S2L and Figure S3Y), while ATS sequences were divided into ATSA, ATSB, ATSpam, ATSvar1, and ATSvar3 (Figure S2M).

The 5′ upstream sequences of *var* genes were analyzed by two different methods: Markov clustering (MCL) [51]–[53], and neighbor joining (NJ) clustering (based on multiple sequence alignments). The two analyses yielded congruent trees, although additional subclusters could be identified in the NJ tree (Figure S2N and O). All previously suggested UPS subgroups [16] (UPSA1–2, UPSB1–4, UPSC1–2 and UPSE) could be identified, although with some modifications and four additional subgroups (UPSA3 and UPSB5–7).

Although the number of available *var* sequences varied between the seven studied genomes (39 to 63), the genomes contained similar *var* UPS distributions (Figure 1), and as expected, UPSE flanked *var2csa*, NTSA and ATSA were exclusively encoded by UPSA flanked genes, whereas other NTS and ATS classes were found in UPSB and C flanked genes. The general observation that UPSA and UPSB genes are located head to head in the telomeres was also confirmed (data not shown), although only limited information on chromosomal location was available. Based on domain annotation of the extracellular part of PfEMP1 (Figure 1 and Figure S4), these could be described as consisting of four components: component 1 (present in ∼95% of all PfEMP1) containing the N-terminal NTS-DBLα-CIDR domains, component 2 (present in ∼43% of all PfEMP1) containing one to three DBLβ and DBLγ domains, component 3 (present in ∼80% of all PfEMP1) containing DBLδ-CIDRβ/γ domains, and component 4 containing C-terminal domain combinations of DBLζ and DBLε domains (present in ∼28% of all PfEMP1) or single DBLβ or DBLγ domains (present in ∼8% of all PfEMP1). The complexity of domain structure followed the UPS classification, in agreement with established group A, B and C PfEMP1 nomenclature [10]–[11]. There was an overrepresentation of component 2 encoding genes in group A compared to group B or C *var* (p<0.0001; X^2^ test of component 2 prevalence in group A–C), and component 4 was found in both group A and B but rarely C.

PfEMP1 inter-domain (ID) sequences were also aligned and classified. Most ID sequences were found to flank component 3, and characteristic for these sequences were long Pro-rich stretches, charged polyAsp/Glu stretches, and an amino acid composition biased towards Ala, Asp, Glu, Pro, Lys, and Val. The sequences downstream of component 3 could be classified, and were either of a M3A type if flanked by component 4, or M3AB if flanked by TM-ATS (Figure S3Z and Figure S4). Due to less functional constraints, ID sequences may have more relaxed requirements to the position of recombination break points, compared to within domains. The ID sequence variation supports the division of PfEMP1 into the four components, which suggest that the low-complexity ID sequence may act as recombination break points.

#### Inter-domain elasticity

The function of the ID sequences is unknown, although one possibility is that these regions confer elasticity to the PfEMP1 proteins, as suggested for similar sequence in the PEVK region of the human striated muscle protein titin (also known as connectin). The PEVK region of titin contains several PPAK domains, a 26–28 residue repeat consisting of low-complexity sequence biased towards Pro, Ala, Val, Lys, and Glu, and these domains are interspersed by polyGlu regions. The PEVK region length is correlated with elongation ability of sarcomeres in striated muscle [54], and the secondary structure has been found to be disordered [55].

The PfEMP1 ID regions are found in lengths up to ∼200 residues, and the amino acid composition is very similar to the one found in titin PEVK. Hits to the Pfam PPAK domain definition [56] in four PfEMP1 supports the sequence similarity (E<0.1 in DD2var52, IT4var64, HB3var34 and PFCLINvar47). The acidic and basic residues can potentially form random structures based on polar interactions, mixed with Pro which introduces kinks in the protein backbone, together forming a structure with spring-like properties. Elasticity could enhance the ability of infected erythrocytes to adhere to endothelial cells by providing a smooth deceleration, as well as extend the time given to establish strong molecular interactions with targets. It is likely that the variant disordered structure of the inter-domain regions impede antibody targeting.

### PfEMP1 groups contain specific subclasses of DBL and CIDR domains

**Figure 2:**
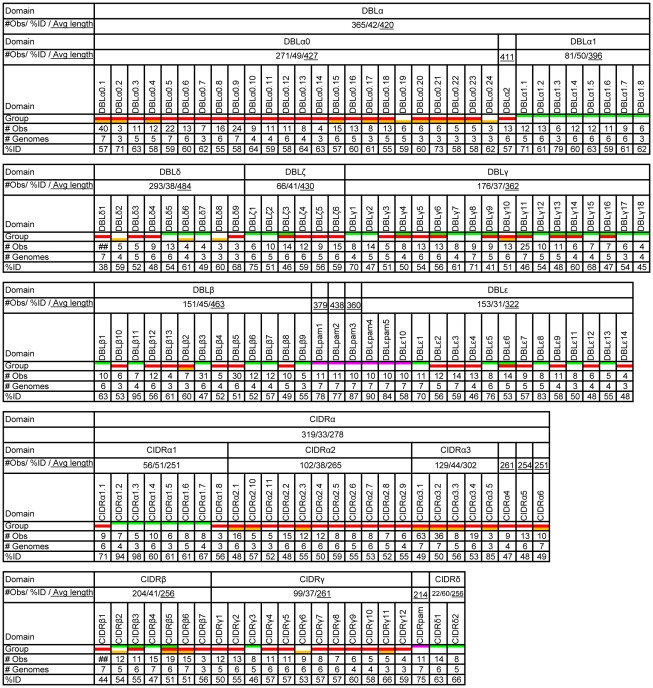
DBL and CIDR domain class characteristics. Number of observations (#obs) of CIDR and DBL domain classes in 399 PfEMP1 (Figure S5), number of genomes represented in the classes (#genomes) (of the seven genomes 3D7, HB3, DD2, IT4, PFCLIN, RAJ116 and IGH), and the average shared sequence identity of major and minor subclasses (%ID). A color was added under domain classes where at least 25% of the observed domains were found in UPSA (green), UPSB (red), UPSC (yellow) or UPSE (pink).

The redefinition of domain borders, and the large increase in sequence data, called for a detailed subclassification of PfEMP1 domains. This was done by a distance tree analysis described in detail in Text S1, summarized for DBL and CIDR domains in Figure 2. The sequence diversity of the major DBL and CIDR domain classes differed both with respect to homogeneity (i.e. shared AA %-identity), and the degree to which subclasses could be distinguished. The previously observed division of DBLα into DBLα1 and DBLα0 [10]–[11] was confirmed, however a third distinct class of sequences, DBLα2, was also identified. Sequences of DBLα1 grouped relatively evenly into eight subclasses, including the particularly distinct DBLα1.3 of VAR3 (note description of nomenclature usage in Text S1), whereas the DBLα0 sequences spread more unevenly into 24 subclasses (Figure S2A,B and Figure S3I–K). The homology block analysis of VAR3 (described in the homology block section below) revealed that the N-terminal part of DBLα1.3 is similar to other DBLα domains, but interestingly, the C-terminal half of the domain is essentially a DBLζ3 domain. All DBLε and DBLζ domains grouped evenly into distinct subclasses, while DBLβ and DBLγ domains were divided into less distinct subclasses of varying sizes, and most (∼90%) of DBLδ sequences could not be subclassified. The homogeneity of the six major classes differed with DBLβ domains being the most (45%) and DBLε the least (31%) homogenous classes. In particular subclasses DBLε1/2/11/13 were distinctively different from the majority of DBLε domains (Figure S2E and Figure S3N–Q). Similar to DBL domains, the level of subclassification of major CIDR domain types varied. Most members of CIDRα3.1 and CIDRβ subclasses could not be separated, whereas other CIDR domains grouped in evenly sized subclasses. The homogeneity of CIDR classes varied with CIDRα1 and CIDRδ domains exhibiting higher sequence similarities than the other CIDR classes. Sequence conservation logos for all large CIDR classes can be found in Figure S3A–H.

Annotation of the PfEMP1 using detailed DBL and CIDR subclassification (Figure S4) showed that most classes could be linked to a specific UPS class (Figure 2). When domain classes were found frequently in genes of more than one group, they were most often shared between group A and B or group B and C, but rarely A and C. These observations support the validity of the subclassification, and the notion that group A–C *var* genes predominantly recombine separately.

In conclusion, the phylogenetic domain analysis allowed classification of all PfEMP1 domains, and defined several novel domain classes. In addition, PfEMP1 domain variation was described in an unprecedented level of detail, by the allocation of the DBL and CIDR domains into subclasses. This classification is based on domain similarities averaged over the whole domains, opposed to local similarities which may vary across the length of the domains, as described in the homology block analysis below. The validity of the classification must be experimentally tested, but the association between domain and UPS class suggests, that at least some of the domain subclasses confer specialized cytoadhesion properties.

### Identification of conserved PfEMP1 domain cassettes

**Figure 3:**
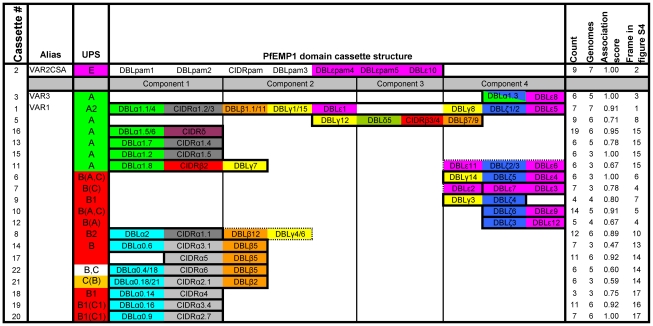
Overview of distinct PfEMP1 domain cassettes. A PfEMP1 domain cassette was defined as a *var* gene sequence encoding two or more DBL or CIDR domains with subclasses that could be predicted from each other. In a few cases domain cassettes (filled frames) could be expanded with additional domains but in limited number of genes or genomes (punctured frames). A cassette was given an association score calculated as the average of all domain pair associations of a domain cassette. Each domain pair association (A–B) was calculated by dividing the number of times the domain combination was observed in the dataset by the least number of times either A or B was found in the dataset. The association score does not include the UPS association. Associated UPS classes are colored according to the UPS class most often observed flanking the cassette. Less frequent flanking UPS classes are in brackets. The number of times a given domain cassette was observed (count) and the number of genomes in which it is present (genomes) within the seven genomes, 3D7, HB3, DD2, IT4, IGH, RAJ116 and PFCLIN are given. The frame number in Figure S4, detailing the genetic context of the domain cassette is also given.

Conserved domain compositional features in PfEMP1 molecules were studied in alignments of annotated PfEMP1 sequences. Alignments guided by conserved C-terminal and N-terminal domain architectures are given in Figure S4A and B, respectively. In particular the alignments were investigated to identify domain cassettes, which were defined as two or more consecutive domains belonging to particular subclasses and present in three or more of the 7 genomes (summarized in Figure 3).

The three conserved *var* genes *var1*, *var2csa* and *var3* (Figure 3, cassettes 1–3), all encoding unique DBL domains, were present in all seven *P. falciparum* genomes, except *var3* which was not present in HB3 and IGH. As previously reported, fragments of *var1* and *var2csa*, but not *var3*, were found in *P. reichenowi* [57]. Some variation in domain composition was observed within the three conserved gene families. Thus, in RAJ116, *var2csa* encoded an extra C-terminal DBLε domain, and in DD2, the *var1* gene encoded C-terminal domains different from the other *var1* genes. *Var1* of 3D7 and IT4 appeared to lack an exon2 sequence, whereas five *var1* genes had a premature stop codon at similar positions in their exon2. Domain pairs characteristic for *var1* (DBLγ1/15-DBLε1 and DBLζ1/2-DBLε) were found in other group A *var* genes (IT4var9, IGHvar32, DD2var23 and HB3var06). Taken together, this indicates that *var1* often is found as truncated gene, and that the particular functional properties of VAR1 may have moved to other PfEMP1 variants. Similarly, a VAR3 sequence corresponding to 80% of the exon1 as well as exon2 was found in the 3′ end of RAJ116var03, consistent with how DBLζ and DBLε domains are positioned in other PfEMP1. The domain composition variation within the three most conserved *var* genes highlight the importance of ectopic recombination of large single or multi domain elements for the generation of PfEMP1 diversity.

Among the novel domain composition phenomena, domain cassette 5 (Figure 3) was the most prominent. This four domain C-terminal cassette was found exclusively in ten group A PfEMP1, and in six of the seven *P. falciparum* genomes as well as in *P. reichenowi.*

Interestingly, nearly all DBLζ and DBLε domains were found in C-terminal domain cassettes (domain cassettes 1,3,6,7 and 9–12) and often occurred in genes encoding CIDRγ1/2/9 domains (approx. three of four CIDRγ1/2/9 domains flank DBLζ and DBLε domains). The unambiguous partition of DBLε subclasses and the positional and compositional similarities between different DBLε, could suggest that specialized functions reside in these structures.

In the PfEMP1 N-terminal, DBLα subclasses correlated well with the subclasses of their neighboring CIDR domain (Figure S4B). As expected, all group A PfEMP1 except VAR3 exclusively contained the domains DBLα1-CIDRα1/β2/δ/γ3, but furthermore, group A PfEMP1 appeared to be divided into those harboring either DBLα1.5/6/8-CIDRβ2/γ3/δ (includes cassettes 11 and 16 in Figure 3; Figure S4, frame 15) or DBLα1-CIDRα1. Within group B and C PfEMP1 two major groups were observed, those encoding DBLα0 domains associated with CIDRα2, and those encoding DBLα0 domains associated with CIDRα3. In addition, eight distinct CIDRα containing cassettes were found, including domain cassette 8 which is particularly noteworthy, as it is associated with UPSB2 (7 of 12 domain cassette 8 encoding genes are flanked by 7 of 11 UPSB2) and contains DBLα2, which formed a separate cluster from DBLα0 and α1 in the DBLα tree. Domain cassette 8 may be expanded further in a less well defined form with two domains (DBLβ12-DBLγ4 or DBLγ6) (Figure S4A, frame 10).

Several more elusive domain architectural constraints were observed, which may crystallize into domain cassettes if higher sequence depth is acquired. These included the group A specific domain combinations DBLα1.4-CIDRα1.6/7-DBLβ3, which both could represent the core of what have been proposed as VAR4 (represented by PFD1235w; Figure S4A, frame 9) as well as DBLβ7-DBLγ-DBLγ (Figure S4A, frame 9).

The present description of PfEMP1 diversity was based on analysis of seven near complete genome sequences: four Asian, two African [58], and one Central American isolate. None of the described domain architectural constraints were found exclusively in the African or Asian isolates, which strongly imply that there is no basic difference between the PfEMP1 repertoires of *P. falciparum* around the world. However, more *P. falciparum* genome sequences are desirable to gain a better resolution of conserved domain cassettes.

In general there were no correlation between occurrences of N-terminal and C-terminal domain cassettes, and whereas group A PfEMP1 shared no N-terminal domain cassettes with group B or C PfEMP1, C-terminal domain cassettes were more often shared among PfEMP1 groups. The three conserved *var* genes have already attracted warranted attention, but while the binding specificity of VAR2CSA and its relevance in pregnancy malaria is well established, no function or clinical importance has been assigned to VAR1 and VAR3. Several studies have aimed to define the PfEMP1 molecular background for severe malaria in children. Most *ex vivo* studies [27], [29]–[31] have relied on relating phenotypic or clinical data to the phylogeny of partial DBLα tags amplified from parasite cDNA, or direct quantitative PCR measurements of group A, B and C *var* genes. Although these approaches target some of the best conserved PfEMP1 phenomena, both methods disregard the structures unlinked to the PfEMP1 N-terminal, and fail to reflect some of the most evident of the conserved N-terminal domain cassettes. Nevertheless, the consensus drawn from these studies and *in vitro* studies of model parasite lines [28] emphasize the importance of group A PfEMP1 in severe malaria, and interestingly, often the particularly distinct group A domain cassette 5 [9], [28], [34].

Although several of the domain classes and PfEMP1 structural constraints presented here are vaguely defined and by themselves difficult to rank according to clinical relevance, the PfEMP1 diversity described by groups, components, domain classes and cassettes offers an operational model for design and interpretations of future experimental studies.

### PfEMP1 homology blocks

DBL domains consist of hyper-variable and conserved regions, as previously described [2], [36], [59], and in a comparison of DBL similarity, Smith *et al.* (2000) were able to define a set of ten homology blocks with an average length of 21 amino acids, conserved in all DBL domain classes [36]. To describe in detail these frequent shifts in conservation level across PfEMP1, an iterative method was developed that automatically defines a set of homology blocks in a set of unaligned protein sequences. The method is especially appropriate for the frequently recombining *var* genes, as the short homology blocks are less inclined to group unrelated sequences which may be forced together in longer domain alignments.

The term homology block (HB) refers to a sequence profile defined from a multiple sequence alignment, here described by a hidden Markov model (HMM) [60]. Sequences with similarity above a threshold to the sequence profile are termed members, hits or occurrences of the homology block, and the members of a homology block can be defined in a sequence by searching with the HMM.

Starting from a full sequence database, homology blocks were one after one first defined and then excluded from the database. Each homology block was defined to be the sequence profile with the highest number of occurrences in the database, i.e. the most conserved sequence, with boundaries optimized to match this criterion. Sequence similarity was assessed with HMM log-odds scores, and a significance threshold of S≥9.97 bits was used for all homology blocks, to ensure that each member of a homology block was at least 1000 times more likely to be related to the sequence profile, than to a random sequence with amino acid frequencies as in the database. Thus, a set of homology blocks was defined, where each homology block comprises all related sequence stretches in the database. The method is described in detail in Text S2.

The analysis was performed on a database with 311 PfEMP1 sequences containing information on the entire molecule or a full exon1. Twenty DBL containing paralogs were also included to enable estimates of evolutionary relationships. The minimal length of the homology blocks was set to seven amino acids, as this is approximately the length required to reach the sequence similarity significance threshold. Sequences with less than five homologs in the database were not included in the homology block set, since PfEMP1 from more than seven *P. falciparum* genomes were in the dataset, and the main interest was to determine sequence features conserved in most of these genomes.

**Figure 4:**
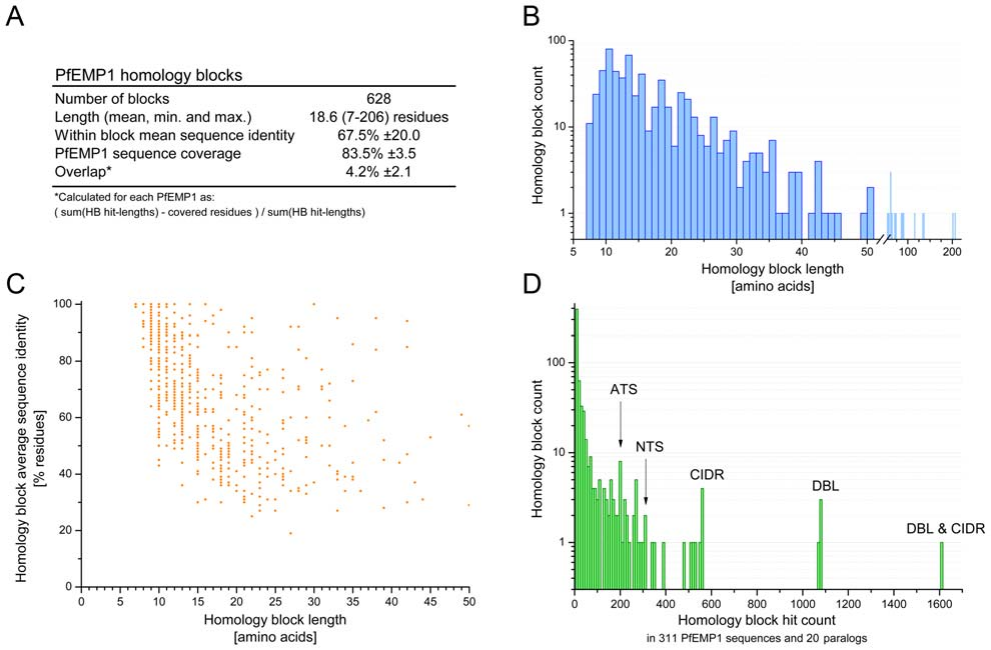
Characteristics for 628 PfEMP1 homology blocks. (A) Length corresponds to the alignment length of the multiple sequence alignment defining the HB. Sequence identity in the table is given as mean and SD for the distribution of all homology block avg. pairwise identities. HB coverage and overlap were calculated per PfEMP1 and mean and SD are given for these distributions. (B) Length distribution for HBs. The most frequent length was 10 residues. (C) Scatter plot showing avg. pairwise sequence identity for HBs of differing length. (D) Histogram showing number of HBs with same prevalences in the database. The bin size of the histogram is 10 hits. One HB was found with a prevalence of 1605 hits in the PfEMP1 database, representing a HB present in nearly all DBL and CIDR domains. Similarly, a number of homology blocks were found specifically in each of the domains DBL, CIDR, NTS and ATS. Most homology blocks had between 5 and 15 hits.

Characteristics for the resulting 628 homology blocks are shown in Figure 4. On average 83.5% of a PfEMP1 sequence was described by homology blocks, and the remaining fragments were either shorter than seven residues, or had fewer than five homologous sequences. Overlap between homology blocks were mainly concentrated in areas with low complexity sequence, such as the inter-domain regions, and amounted to an average of 4.2% of HB occurrences in a PfEMP1 sequence (Figure 4A). The most frequent HB length was 10 residues, while the average was 19 residues (Figure 4B). HB average sequence identity was between 19–100%, and as might be expected for the shortest sequences, only similarities with high identity could be detected within the significance threshold (Figure 4C). The analyzed PfEMP1 sequences contained 311 NTS, 199 ATS, 1043 DBL and 552 CIDR domains, while the paralogs contained 30 DBL domains. One homology block occurred 1605 times in the database and was found in all DBL and CIDR domains, except 20 (not present in DBLpam2, DBLε7 and DBLε12), while four other blocks were found in all DBL domains and six blocks were strongly correlated with CIDR (Figure 4D). The homology blocks were numbered according to their frequency in the database, with the most frequent being HB number one.

88 PfEMP1 were not in the HB definition sequence set, and when the 628 defined homology blocks were predicted in these proteins, 82.5% (SD ±4.9%) of each PfEMP1 were on average covered by HBs, similar to the coverage in the definition sequences (Figure 4A), showing that the homology blocks describe universal PfEMP1 sequence features.

The VarDom server was developed to provide an interactive graphical interface to analyze information on domain classes, homology blocks and their distribution in PfEMP1 sequences. Alignments and other related files can be downloaded, and it is possible to submit new sequences to annotate them with domains and homology blocks, to classify them and relate them to other sequence groups in the seven genomes: http://www.cbs.dtu.dk/services/VarDom/ In the following, the HB distribution in PfEMP1 is presented, and several references are made to specific homology blocks. These blocks as well as the sequences they occur in can be inspected using the VarDom server.

### Homology blocks describe the conserved core of DBL and CIDR domains

**Figure 5:**
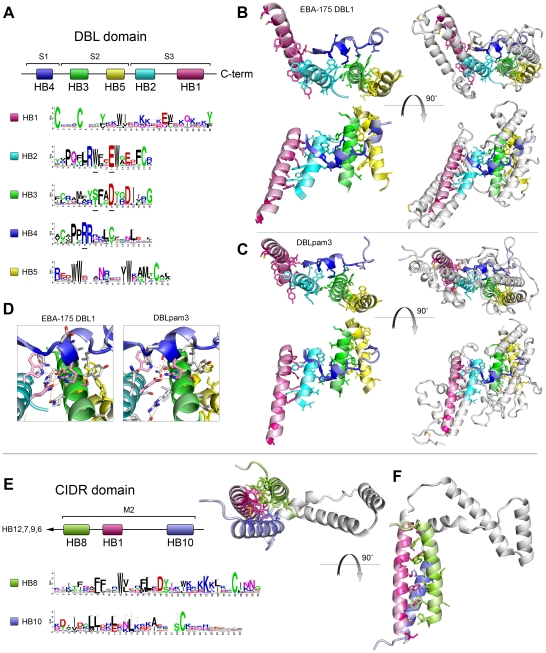
Conserved domain cores. (**A–D**) Five most conserved PfEMP1 homology blocks form DBL-core structure. (A) Schematic showing relative positions in DBL domains of HB one to five (S1–3 indicate subdomains) and sequence conservation logos for each homology block alignment. The height of each position in the logos indicate the amino acid conservation level, and the height of the individual amino acids reflect their relative frequencies on the position and thus their contribution to the conservation. A small sample bias correction has been subtracted in the logos, on alignment positions containing few (<40) amino acids, and error bar height is 2× the correction. Polar amino acids are green, neutrally charged are purple, basic are blue, acidic are red, and hydrophobic amino acids are black. HB numbering is based on level of conservation in PfEMP1 and related sequences. (B) HBs shown on PfEBA-175 DBL1 structure, and (C) on VAR2CSA DBLpam3 structure. Side chains are shown for conserved positions with conservation level higher than 50% of maximum, corresponding to 2.16 bits. DBL areas which are not part of HB1–5 are shown as lightgray in rightmost column, while left side shows only HB1–5, color coding as in panel A. Coloring intensity in the structure is proportional to conservation level in the HBs. (D) Polar interactions between conserved positions in EBA-175 and DBLpam3. The conserved pink residues are underlined in Figure 5A. (**E–F**) Conserved sequence blocks in CIDR domains. Relative homology block positions, and sequence logos (E). HB12, 7, 9 and 6 are all strongly correlated with CIDR domains. (F) HBs shown on the structure of the M2 part of MC179 CIDRα domain. Disulfide bridges are shown in orange.

The five most prevalent homology blocks in PfEMP1 (HB1–5) were present in nearly all DBL domains. The relative positions of these five HBs in DBL domains were conserved (Figure 5A), and within the HBs several amino acid positions were strongly conserved in all DBL domains. Figure 5B shows occurrences of HB1–5 in DBL1 (a.k.a. F1) of the paralog PfEBA-175 and DBLpam3 (previously DBL3X) of VAR2CSA. The DBL structure consists of subdomain 1 (S1) with mixed helix-sheet structure, and two helix bundles (S2 and S3) [37], [49]. Disulfide bonds between conserved Cys residues mainly serve to hold together each individual subdomain, demanding other types of interactions to hold a stable domain structure [37], [50], [61]–[62]. HB1, which was also found in CIDR domains, described a complete α-helix with one side conserved, giving a pattern of conserved residues spaced by 3 residues for each helix-turn (Figure 5A). The conserved side of HB1 faced HB2, which was found to be the most conserved sequence in DBL domains, with a mean amino acid sequence identity of 56%. HB2 was part of a longer helical structure and interfacing with HB1, HB3 from the other helix bundle, and HB4 which formed the non-surface exposed part of S1 (Figure 5B and C). All these interactions probably constitute the main selection pressure, keeping HB2 relatively conserved. HB3 in S2 corresponded to HB2 in S3, with interactions to HB2, HB5 and HB4, and with mean sequence identity of 47% it was found to be the second most conserved part of DBL domains. HB5 was mainly conserved on one side of the helix like HB1, suggesting for both that they may be frequently exposed on the surface of PfEMP1.

Side chains in conserved amino acid positions were mainly directed towards other conserved parts, although some were pointing outwards probably to interact with other less conserved domain parts (Figure 5B and C). Functions for some of the conserved amino acids in HB1–5 were identical in both structures (Figure 5A and D), where they formed polar and hydrophobic interactions between the three subdomains. Besides from the conserved polar interactions shown, the conserved Pro on position 4 in HB4, which introduced a kink in the β-sheet structure of S1, was in a position allowing it to interact hydrophobically with the also conserved Trp on position 8 in HB2. It may thus contribute to hold the β-sheet in place. In general the conserved positions of HB1–5 described a set of residues, which in the known DBL domain structures interact to hold together the three DBL subdomains, so they can be said to constitute the conserved core structures and interactions of DBL domains.

HB1–5 were found among the 10 homology blocks defined by Smith *et al.* (2000) [36], where HB4 = HBb, HB3 = HBd, HB5 = HBf, HB2 = HBh and HB1 = HBj. The remaining homology blocks, defined in that paper, were not found to be conserved in all DBL classes, based on the chosen similarity significance threshold.

Homology blocks specific for all CIDR domains were also found, and they were present in the most conserved part of CIDR, the designated minimal CD36 binding region or M2 [36], [45], for which the structure is known [48] (Figure 5E and F). HB8, HB1 and HB10 were found to correspond to helix 1, 2 and 3 respectively in the three-helix bundle, and the similarity of this bundle to subdomain 3 of DBL was confirmed by the presence of HB1 in all CIDR and DBL domains. The conservation of these three helices suggests that this structure is common to all CIDR domains. Interestingly, four HBs (HB12, 7, 9 and 6) situated in subdomain S3 of DBLα and DBLδ domains, were exclusively found flanking all CIDR domains, strongly supporting the link between CIDR and DBL domains.

Side chains of conserved residues in HB1, 8 and 10 were mainly directed towards the center of the CIDR three-helix bundle (Figure 5F), where they interacted to keep the structure together. Some parts of the structure have not been solved, including the C-terminal end of HB8 with several conserved basic residues and a Cys likely to form a disulfide bridge to position 1 in HB1. A few conserved residues in HB8 were directed away from the helix bundle core. Among these were the basic position 24 and possibly also 28 as the distance fits with a helix turn. These residues may thus be involved in interactions with surrounding parts of the PfEMP1 such as the helix-loop of CIDR, or even substrate binding, and they may be target for the cross-reactive antibodies inhibiting CD36 binding described by Mo *et al.* (2008) [63].

### Alignment of DBL homology blocks

**Figure 6:**
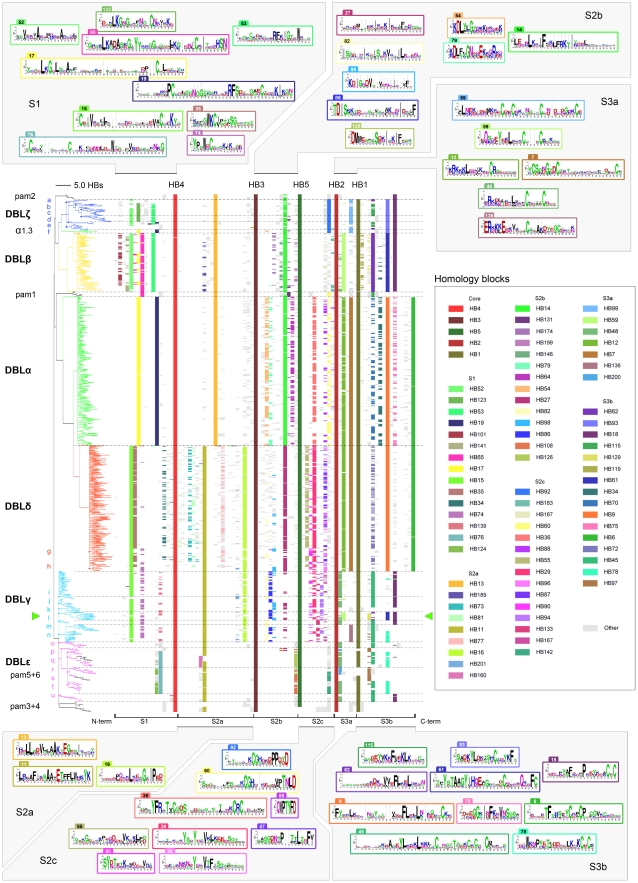
DBL homology block alignment. HBs in 1043 DBL sequences aligned, and sorted by NJ-clustering based on differences in HB composition. Tree distances show the number of different HBs in the DBL domains. The sequences are divided into 6 segments by the conserved core HB1–5 (Figure 5), and the corresponding subdomain parts are noted below the alignment. Only the 80 most frequent of 378 HBs are colored. Sequence conservation logos as described in Figure 5 are shown for selected HBs, where number tabs indicate the HB number. Logos are when possible placed in order of appearance in the alignment. Letters next to the tree identifies groups marked by dots in the tree, matching domain subclassification based on amino acid alignments: (**a**) ζ3, (**b**) ζ5, (**c**) ζ6, (**d**) ζ4, (**e**) ζ1, (**f**) ζ2, (**g**) δ5, (**h**) δ4/8/9, (**i**) γ7, (**j**) γ11/15, (**k**) γ1, (**l**) γ2/9, (**m**) γ8, (**n**) γ5/6/12/16/17, (**o**) ε2, (**p**) ε7, (**q**) ε4, (**r**) pam6/ε3, (**s**) pam5/ε5/ε12, (**t**) ε6/9, (**u**) ε1/11/13. The green pointers mark products of recombination between DBLγ and DBLβ domains, with break point around HB2. Additional information for all HBs can be found by querying the VarDom server with the HB numbers, as given in the legend or on the logos. Labeled homology block alignments can be found in Figure S7.

Just as PfEMP1 can be represented as strings of amino acid symbols or strings of domain names, they can be represented at an intermediate level as a string of homology blocks. To study similarities between DBL domains, the homology block sequences of 1043 DBL domains, consisting of 378 different HBs, were studied (Figure 6). Occurrences of the same homology block were vertically aligned (Figure 6, center), and rows in the alignment were sorted according to a NJ-tree (Figure 6, left) built based on differences in HB composition of the DBL sequences. The five core homology blocks divides DBL domains into six regions, and sequence conservation logos are shown for representative homology blocks in each region (Figure 6, top and bottom).

Many of the domain classes derived from trees based on amino acid alignments (Figure 2 and Figure S2), were also found by the tree based purely on the absence or presence of homology blocks (Figure 6), and these groups can thus be described by a specific homology block combination. Most major classes formed monophyletic groups, with the exception of DBLγ and DBLε, which formed one big cluster with several well-defined subgroups. Minor subgroups were mainly found in DBLζ, γ and ε (Figure 6, tree group a–u), and many correlated well with domain classes based on amino acid alignments. Most subgroups of DBLα, β, and δ were too subtle to be distinguished. The DBLα0-DBLα1 division was not clearly found, although HB36 may approximately describe the difference, by being present in 205 of 230 DBLα0 domains, and in none of the 61 DBLα1. HB36 was absent in all cys2 sequences but present in all cys4 sequences, thus describing the division between group 1–3 and 4–6 in the DBLα sequence tag classification [64].

Domain subclasses (Figure 2) could often be described by subclass specific homology blocks. For instance DBLζ4 was described by HB283 and HB284. Other subclasses were characterized by HBs shared exclusively with other major domain classes, examples being DBLζ1, which shared HB19 with DBLα (Figure 6e S1, blue), and DBLγ2/9 domains, which were characterized by having a DBLβ S3 subdomain (Figure 6 S3, green pointers). Similarly, the S3 subdomain of VAR1 DBLε1 was very similar to the one present in a number of DBLγ sequences (Figure 6 S3, tree group u). Cassettes could also be identified, exemplified by HB331, which occurred exclusively in the N-terminal of DBLβ domains in domain cassette 5 (Figure 3).

DBLα1.3 of VAR3 contained HB17 and HB19 which were characteristic for DBLα domains (Figure 6 S1), but S2c and S3 in DBLα1.3 were very characteristic for DBLζ, sharing several DBLζ specific homology blocks: HB92, HB99, HB592, HB93, and HB18. Thus, homology block analysis of VAR3 suggests that DBLα1.3 is a DBLα-ζ hybrid, and it will be interesting to see if the function of this domain is similar to any of the two combined classes alone. The finding of DBLζ elements in VAR3 associates this PfEMP1 with the domain combination DBLζ-DBLε, often found in component 4 cassettes (Figure 3, Component 4), which could imply functional analogies between VAR3 and these cassettes.

DBLpam1 and 2 shared homology blocks with DBLα/β/ζ, while DBLpam4 and to a high degree DBLpam5 and 6 shared blocks with DBLγ/δ/ε (Figure 6). Interestingly, DBLpam1 contained HB65 (Figure 6 S1, pink), a sequence that was mainly found in DBLβ. However, in the C-terminal end DBLpam1 shared HB60 with DBLα (Figure 6 S2c, yellow) and HB115 with DBLζ1/5/6 (Figure 6 S3b, green, tree group b, c and e). Thus, DBLpam1 appeared to contain elements from all of DBLα, β and ζ. The shared homology blocks, as well as the fact that the hybrid domains DBLα1.3 and DBLpam1 appears to be functional, suggests a more recent common ancestry and possibly related functions of DBLα, β, ζ, pam1 and pam2 domains.

Similarities between major DBL classes also varied considerably across the length of the domains (Figure 6), and a major homology break point, where similarities differed on each side, was observed for many sequences around HB2, the most conserved DBL homology block.

In the N-terminal, a clear division was found between DBLα/β/ζ and DBLγ/δ/ε, best defined by HB11 and HB13, respectively (Figure 6, S2a). At this end of DBL domains, only the core homology blocks HB1–5 occurred in both groups, indicating low levels of recombination between these groups, and possibly different functions. Within these groups, DBLζ had high similarity to DBLβ, most significantly in the S1 subdomain, and DBLδ was very reminiscent of the DBLγ in the N-terminal, some sequences were even identical on the homology block level (Figure 6g, h, j, and k).

The C-terminal of DBL domains could also be divided into two major groups, consisting of the S3 subdomains of DBLα/δ and DBLζ/β/γ/ε, respectively (Figure 6, S3). DBLα and δ shared four homology blocks connecting to the downstream CIDR domains. S3 homology blocks in DBLζ and β were uniform and specific to each class, whereas DBLγ and ε S3 were more diverse (Figure 6, S3).

N- and C-terminal ends of several major DBL domain classes thus appear to have different sequence similarities, most likely reflecting that the sequences have been joined through recombination, often with a break point around HB2, and they therefore have different evolutionary histories. Phylogenetic classification based on whole domain sequence alignments will tend to be an average of such different histories.

#### Evolutionary relationships among DBL subdomain sequences suggest intra-DBL recombination break point

**Figure 7:**
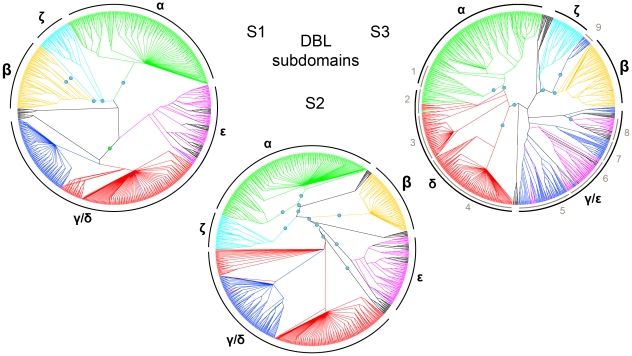
Evolutionary relatedness of DBL subdomain sequences. A cladogram is shown for each of the three DBL subdomains S1–3, where boundaries for the subdomains were chosen at the edges of HB4 and HB2, as shown in Figure 6. Colors indicate major DBL domain classes estimated from alignment of the whole domains: Green: DBLα; Orange: DBLβ; Blue: DBLγ; Red: DBLδ; Magenta: DBLε; Cyan: DBLζ. VAR2CSA sequences are black. Blue dots indicate major bipartitions supported by at least 50% of 1000 bootstraps. The green dot in S1 marks a bipartition with bootsrap value 0.39. Subdomain clade correlation with whole domain classes is indicated around the trees in black; Clades were split if supported by 50% of the bootstraps.

Identification of adjacent genetic regions with different evolutionary histories is a widely used method for detecting recombination break points in distantly related sequences [65]–[66]. To get a complete picture of evolutionary relations among subdomain sequences, with the aim to determine if recombination has occurred with break point between S2 and S3, phylogenetic trees based on amino acid alignments were built for the three DBL subdomains (Figure 7). Trees in Figure 7 are included as Figure S6 with labels and bootstrap values.

Relations among sequences of the S3 DBL subdomain clearly differed from those of S1 and S2 (Figure 7). DBLα and DBLδ S3 subdomains were found to be closely related, separated from the remaining sequences in all 1000 bootstraps, whereas in S2, DBLα was most closely related to DBLβ and ζ, supported by 99% of the bootstraps. Similarly, DBLγ and DBLε S3 subdomains were closely related, while S2 sequences of DBLγ were closely related to DBLδ, separated from DBLε by several highly supported branches. This strongly indicates that the evolutionary histories for S2 and S3 subdomains are different, as also suggested by the homology block analysis (Figure 6), and that recombination most likely has occurred with break point between these subdomains.

In agreement with the homology block analysis, the division between DBLα/β/ζ and DBLγ/δ/ε was well supported by bootstrap values in both S1 and S2, as was the separation of each of the domain classes DBLα, β, and ζ (Figure 7, S1 and S2). For S1 and S2, DBLδ and DBLγ sequences were clustered together with low bootstrap support for separation within this group, although a specific set of DBLδ sequences had particularly close relations to DBLγ (Figure 7, S1 and S2). The relationship was most pronounced in the S2 subdomain, where 46 DBLδ sequences represented in all seven genomes, and including all non-DBLδ1 subclasses, were found closer to the DBLγ clade. The 3D7 genes containing these DBLδ sequences were MAL6P1.4, PF11_0521, PF13_0003 and PF11_0008. The latter *var* gene has been found to be the target for protective antibodies [9], [34], and together with PF13_0003 contains cassette 5 (Figure 3).

DBLα1 S3 sequences flanked by CIDRα1 domains were well supported as a subgroup (Figure 7 S3-1). Interestingly, all those DBLα domains that were not followed by CIDRα (DBLα1.5/6/8 domains), had an S3 subdomain which clustered with DBLδ S3 sequences (Figure 7 S3-2), indicating recombination between DBLα and DBLδ. Similarly, DBLδ clusters were found for DBLδ domains followed by CIDRγ (Figure 7 S3-3), and CIDRβ (Figure 7 S3-4). These associations between S3 and CIDR indicate that the recombination break point occurs within the DBL domain when CIDR domains are exchanged, and further supports a functional dependency between CIDR and their upstream DBL domains.

DBLγ and DBLε S3 subdomains were found mixed in one cluster with low bootstrap support (Figure 7 S3-5, 6, 7, 8), although the subgroups were to some degree specific for either DBLγ or DBLε. One DBLγ clade was composed of S3 subdomains of DBLγ5/6/12/16/17 (Figure 7 S3-7), captured by HB136 (Figure 6n) and found in a set of 36 PfEMP1 nearly void of DBLε and ζ domains. Two small DBLγ subgroups, DBLγ1/15 of VAR1, and a group comprising DBLγ2/9 domains, were found separately, and the latter group was closely related to DBLβ S3 sequences (Figure 7-S3-9), as expected from the homology block alignment (Figure 6k and l). These DBLγ-β hybrid domains appeared in 16 PfEMP1, found in 6 of 7 genomes (not HB3), the 3D7 gene being PF07_0050.

DBLε S3 sequences were dichotomized with a bootstrap support of 80%. One clade contained all DBLpam5, two DBLpam6, as well as DBLε5/7/12 (Figure 7 S3-8). The S3-8 cluster was characterized well by HB97 (Figure 6p and s), which was also present in several paralogs, such as PFA0665w DBL2 and PFD1155w DBL2, indicating that HB97 describes an ancient conserved domain element, a notion supported by its presence in the conserved genes *var1* and *var2csa*. The presence of HB97 in paralogs and many DBLε domains, suggests that of all PfEMP1 domain classes, DBLε may bear the highest resemblance to a common ancestral DBL domain.

The subdomain sequence comparison thus corroborates observations on homology block and domain level. The relations found between S3 subdomain sequences differ markedly from relations between S1 and S2 sequences, which supports the theory of a recombination hotspot between subdomain S2 and S3. The homology block analysis further suggests that the break point often occurs around HB2.

The subdomains S1 and S2 of DBLγ and DBLδ domains appear to be closely related, whereas the S3 subdomain sequences are distantly related, indicating recombination with break point around HB2. Furthermore, HB2 recombination products have been identified with 5′ DBLγ and 3′ DBLβ/ε sequences, as well as with 5′ DBLα and 3′ DBLδ sequences.

The area around HB2 is a hotspot in the sense that recombination has occurred at this position more frequently than at other sites during the history of the *var* genes. It is however difficult to say if this area has an especially elevated recombination frequency, or if the high number of observed recombination events is purely due to functional selection, i.e. there has been recombination all over the gene, but mainly recombinants with break points near HB2 have been retained due to better functionality. Recombination between DBLβ and DBLγ appears to be rare, judging from the fact that DBLγ-β hybrid domains are represented in 6 of 7 genomes (Figure 7-S3-9, Figure S6), and that these sequences form a cluster in the HB61 tree. This is suggestive of a common ancestral sequence dating back before geographic separation of the genomes. Recombination between DBLα and DBLδ with break point in the HB2 area, resulting in S3 and CIDR domain exchange, may be a more frequent event, judging from the fact that all four combinations of DBLα/δ-CIDRβ/γ occur, which are likely to be the product of at least two recombination events. Corroborating this, S3 subdomains followed by CIDR1β and CIDR2β clustered together, separate from a cluster of S3 sequences followed by CIDR1γ and CIDR2γ (Figure 7 S3-2). These sequence relations were also found in the phylogeny for HB7, indicating that the break point of these recombination events occurred upstream of HB7, and thus near HB2.

Frequent recombination around HB2 could suggest independent functions for S1+S2 and S3, as proposed for VAR2CSA domains where S3 generally was found to be less surface-exposed [50]. This may be particularly true for DBLγ/δ S1+S2 sequences, as they apparently can be combined successfully with very diverse downstream sequences, including DBLβ S3 subdomains and CIDR domains.

Recombination is also likely to occur between more closely related domains, e.g. within a domain class. This will probably occur more frequently due to higher sequence similarity, but will result in more subtle changes. DNA must be analyzed to detect such subtle changes optimally, and this could be done by studying the phylogenetic trees built for each homology block. This comprehensive task is however not within the scope of the current study. A recombination analysis has previously been performed on sequences encoding DBLpam3 domains [59], and interestingly the most significant recombination hotspot in this DBL class was also found near HB2.

#### Potential integrin binding of DBLα0 domains

Integrins are a family of cell surface membrane receptors, mediating binding to the extracellular matrix, as well as interacting with plasma proteins and counter receptors on other cells, thereby involving them in basic processes such as cell adhesion, cell migration and cell-cell communication. Integrins are heterodimers composed of two membrane anchored subunits, α and β of which the human genome encodes 18 and 8 variants respectively, combining into 24 known, human receptors [67]. Integrin subunit homologs are found in both complex and simple metazoan organisms including sponges and corals [68], and the wide distribution, both in species and across tissue types, makes the receptors an attractive target for pathogens, such as various bacteria, viruses, fungi, and parasites, which use these receptors for adhesion or internalization in the host [69]–[72]. Disintegrin domains in snake venom toxins, as well as ornatin from leech toxins, bind integrins to inhibit their function in platelet aggregation [73]. It has previously been shown that IE adhesion to human dermal microvascular endothelial cells (HDMEC) can be inhibited by anti-α_v_ antibodies (i.e. antibodies targeting the v variant of integrin α subunits), suggesting that IE can bind to α_v_β_3_ integrins [74].

The amino acid trimer motif Arg-Gly-Asp (RGD) is commonly found in integrin binding proteins, including disintegrins, ornatin, and many extracellular matrix proteins. The RGD motif mediates binding to several integrin receptor variants, a binding which often can be out-competed by synthetic RGD peptides, confirming the surprising simplicity of this adhesive interaction [75]. RGD as well as other integrin binding motifs are often found in loops bounded by Cys residues, and the motif together with the flanking residues may determine the integrin type specificity [76]–[77].

The 3D7 proteome was searched for occurrences of the RGD motif, and a high number of motifs was found to be present in PfEMP1 (23 out of 244 motifs, P = 5.8*10^−6^, cumulative binomial distribution with x = 23 motifs, p(RGD) = (244 motifs / 4099411 AA), n = 138055 AA). PfEMP1 domains from seven genomes were then searched, and significantly higher numbers of RGD motifs than what should be expected for random reasons (taking the skewed PfEMP1 amino acid distribution into account) were found in DBLα0 (56 motifs in 229 domains, P = 5.2*10^−14^, cum. binom. distrib. with x = 56 motifs, p(RGD) = 1.77*10^−4^, n = 98157 AA) and to a lesser degree in NTS (12 motifs in 311 domains, P = 1.1*10^−4^, cum. binom. distrib. with x = 12 motifs, p(RGD) = 1.77*10^−4^, n = 20511 AA). Only one motif was found per DBLα0 domain, and all seven genomes had RGD-containing DBLα0 domains. Interestingly all RGD motifs were evenly distributed in three fixed positions in DBLα0: (1) HB19 position 6–8, (2) HB12 position 14–16 and (3) HB7 position 15–17.

The three RGD sites in DBLα0 were predicted to be situated in loop regions by domain structure homology modeling (data not shown), and especially RGD position 2 and 3 were exposed on a loop in subdomain S3, between the helices covered by HB1 and HB2, held in place by several Cys residues.

PfEMP1 similarity to disintegrin and ornatin was found by searching 311 PfEMP1 against the Pfam domain database [56], resulting in six hits to the disintegrin domain, and five hits to ornatin (E<1 for all hits). 10 of these 11 hits were situated in DBLα0, overlapping the second RGD position mentioned above, and not all of the hit sequences contained an RGD motif.

The finding of two independent significant sequence features pointing towards integrin binding, and on top of this, the co-localization of these features in DBLα0, suggests that some DBLα0 domains are likely to mediate integrin binding, which may also be the phenomenon observed by Siano *et al.* (1998) [74].

In relation to this, pentamidine is an RGD analogue used for treatment of many pathogen-caused diseases including malaria [78], and it is possible that this drug may work partly as integrin antagonist, thus to some extent inhibiting IE binding to endothelial cells.

### CIDR homology block alignment

**Figure 8:**
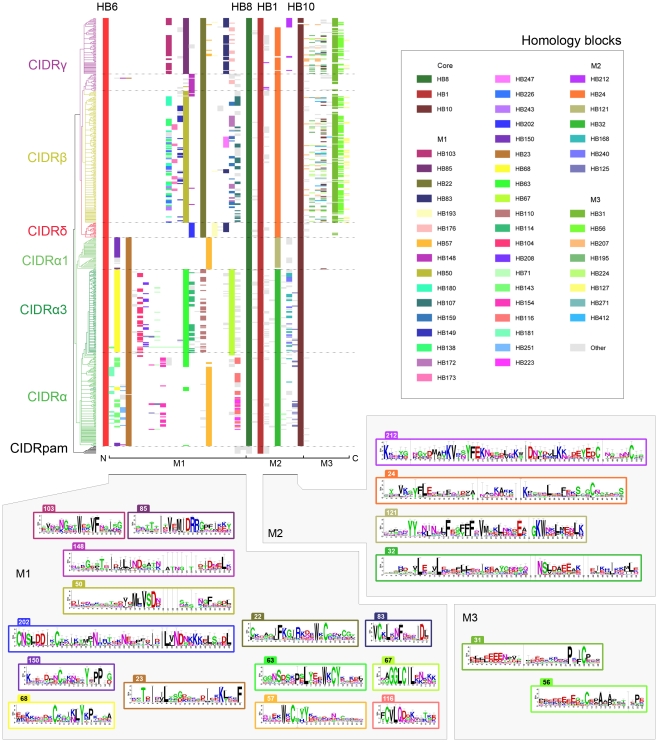
CIDR and M3 homology block alignment. Homology blocks in CIDR domains and M3 regions were aligned, and clustered based on differences in HB composition. The cladogram is colored according to amino acid level domain classification. Only the 54 most frequent HBs are colored, out of a total 158 HBs. Sequence conservation logos are shown for selected HBs in the regions M1–3. Core homology blocks HB1, 8 and 10 are described in Figure 5, while HB6 is the C-terminal of the upstream DBLα/δ domain (Figure 6). Alignments and logos for all HBs can be found by querying the VarDom server with the HB numbers.

158 homology blocks found in 552 CIDR domains were aligned and clustered by HB composition (Figure 8). CIDR domains could be divided into two major groups, CIDRβ/γ/δ containing HB22, and CIDRα with HB23 (Figure 8 M1). No significant homology block similarities were observed between CIDRα and CIDRβ/γ/δ, except the core homology blocks. The CIDRβ, γ and δ domain classes could each be distinguished by class-specific homology blocks, as could each of the CIDRα1 and CIDRα3 subclasses (Figure 8). HB148 described a distinct subgroup of CIDRγ sequences with high similarity to CIDRβ (Figure 8 M1, purple). HB148 was present in 32 PfEMP1 including amongst other PF11_0008 and MAL6P1.4 associated with severe disease [34] and IT4var60 expressed on rosetting IE [16]. Two other interesting CIDR homology blocks, HB450 and HB451, were strongly associated with the previously mentioned conserved domain cassette 8 (Figure 3).

In M2, which for some CIDRα has been proven to mediate CD36 binding [45]–[46], four types of sequences were found to fill the helix loop between the conserved core HBs (Figure 8 M2). CIDRα1 domains, which have been shown not to bind CD36 [47], shared HB121 in the M2 helix loop, which was markedly different from HB32 shared by the remaining CIDRα in this region. CIDRβ/δ/γ domains were characterized by HB24 in M2, except CIDRγ6/8 domains with a differing helix loop defined by HB212 (Figure 8 M2).

**Figure 9:**
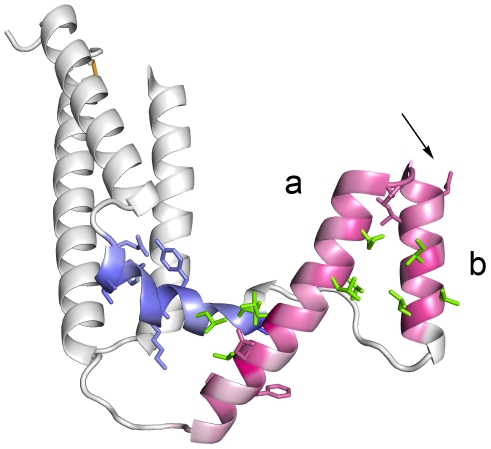
Helix-loop of MC179 CIDRα. HB32 (red) covering helix a and b, and HB372 (blue) covering helix c. Side chains conserved by more than 2.16 bits are shown. Green side chains are conserved hydrophobic residues. The arrow indicates Asn in the possibly surface exposed semi-conserved motif YGN at the apex of helix a and b. The conservation of residues in HB372 with 9 sequences has a high margin of error.

Using the VarDom server, two HBs were found in the helix-loop of the MC179 CIDRα structure: HB32 covering helix a and b (see logo in Figure 8 M2), and HB372 covering the small helix c, a sequence which is mainly present in CIDRα2 domains (Figure 9). Though the structure appeared twisted in the crystal so helix a and b were slightly separated, it was found likely that semi-conserved HB32 hydrophobic positions 17, 18 and 21 in helix a, under monomeric circumstances interact with conserved HB32 hydrophobic positions 45, 48, 51 and 52 in helix b to keep the helices together (logo in Figure 8 M2; Figure 9, green residues). Similarly, the highly conserved HB32 positions 8 and 12 in helix a binds helix c through conserved hydrophobic interactions (Figure 9, green residues). The Asp-Ile-Glu (DIE) motif at HB32 position 44–46 supports CD36 binding, as binding ability has been found to be disrupted when the motif is substituted with the motif Gly-His-Arg [46]. This substitution of a conserved hydrophobic Ile with a charged His residue in helix b, is likely to result in a different conformation of these helices, emphasizing the importance of this helix pairing in CD36 binding. HB32 position 33–41 shows that in a subset of CIDRα (28% of the HB32 sequences), an insertion containing several acidic residues appears at the apex between helix a and b. In the majority of CIDRα, this apex contains a semi-conserved Tyr-Gly-Asn (YGN) motif on position 25 to 28 in HB32, which may also be surface-exposed in the monomeric structure. Phosphorylation sites are predicted in all HB32 sequences, and when present, the Tyr in YGN is also predicted as target for this modification. Phosphorylation is involved in CD36 binding, though only phosphorylation of the CD36 receptor has been shown [79]–[80].

A summary of homology block combinations specific for major DBL and CIDR classes can be found in Table S1. Most major classes can be distinguished by a few homology blocks, the exception being the mixed groups DBLγ and DBLε. Table S1 only shows combinations involving presence of homology blocks, and CIDRγ is hard to describe in this way, though it can easily be described by the presence of HB22, combined with the absence of HB50 and HB202 (Figure 8). These domain class specific homology blocks should be useful when analyzing functional differences, as well as for oligonucleotide array and recombinant protein design.

PfEMP1 DBL domain relations to CIDR and paralog domains were also studied by means of the homology blocks, and the results are described in Text S3, including: PFA0665w containing distantly related DBL and ATS elements, PfDBLMSP with DBLε-like domains, paralog specific homology blocks, and support for the association between the CIDRpam and other CIDR domains.

### NTS homology blocks

**Figure 10:**
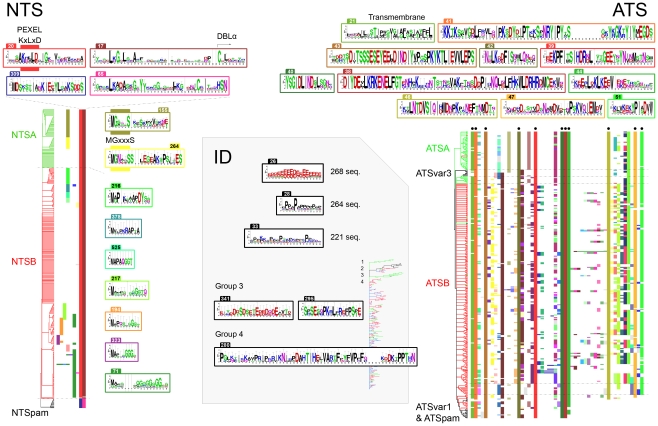
NTS, ID and ATS homology blocks. (**NTS**) Above the HB alignment, sequence conservation logos are shown for the two most conserved NTS homology blocks. The lower pair were found in NTS of VAR2CSA, and HB65 was also found in several DBLβ domains (Figure 6). The proposed PEXEL motif is noted above the HB20 logo, which together with several downstream positions was conserved in all PfEMP1 except VAR2CSA. On the right side of the alignment, logos covering the N-terminal methionine are shown. A conserved N-terminal N-myristoylation motif was found in NTSA HB155 and HB264. (**ATS**) Sequence logos for conserved ATS homology blocks marked by black dots in the alignment. The cladogram is colored according to ATS annotation based on amino acid alignment. Three conserved homology blocks were absent in VAR1 and VAR2CSA ATS. (**ID**) Inter-domain HBs were defined as HBs which occur with a frequency >50% outside other defined regions. Logos for three of the most conserved ID homology blocks are shown, with number of occurrences in the database with 311 PfEMP1 sequences. The phylogram is based on PfEMP1 differences in ID HB composition, where four interesting groups were distinguished: (1) VAR1, (2) VAR2CSA and PfEMP1 with C-terminal similarities to VAR2CSA defined by HB206, (3) group with UPSA flanked *var* including PFD1235w defined by HB295 and HB341 (4) UPSB flanked *var* defined by HB280. The tree is colored according to UPS type, where UPSA is green, UPSB is red, UPSC is blue and UPSE is black. Homology block sequence logos specific for group 3 and 4 in the phylogram are shown.

NTS homology blocks were aligned and sorted according to HB composition (Figure 10 NTS). Two homology blocks, HB20 and HB17, were found in the NTS of all PfEMP1 except VAR2CSA. HB20 described the pentameric motif [KR]xLx[EQD] known as the *Plasmodium* export element (PEXEL), which is required for protein transport to the host erythrocyte [81]. The motif constituted part of a longer motif with conserved positions every 3–4 amino acids, suggesting a conserved side of a structure predicted to be helical [36]. Even more highly conserved were the initial positions of HB17, the LkGxLxxA motif (Figure 10 NTS), which may be an extension of the PEXEL structure or of the downstream DBLα domain. NTSpam lacks the typical PEXEL motif despite of being present on the IE surface, which could be explained by a unique PEXEL motif in HB309 or HB65, both having three conserved hydrophobic positions with a basic and acidic residue conserved on each side of the middle position (HB309 position 7–15, HB65 position 5–9), like PEXEL in HB20 position 4–11.

#### Possible N-terminal N-myristoylation of group A PfEMP1 may anchor N-terminal in membrane and cause alternate transportation to IE membrane

HB155 and HB264 were found in the N-terminal of group A PfEMP1, containing the characteristic motif MGxxx[S/T] required for the lipid modification N-myristoylation (Figure 10 NTS). N-terminal N-myristoylation is the covalent attachment of a 14-carbon myristate group to N-terminal Gly through an amide bond, after removal of the start Met residue [82]. This reaction generally takes place in the cytoplasm during protein synthesis and entails transfer of the lipid chain from myristoyl-CoA, catalyzed by N-myristoyltransferase [83] (reviewed by Resh 2006 [84]). Myristate is able to insert hydrophobically into a lipid-bilayer, and thus create an unstable binding to a membrane [85]. Attachment of an N-myristoylated protein to the membrane can be stabilized by the presence of basic residues interacting with negatively charged membrane phospholipids [86], or by further acylation of the protein [87]. Two important roles for N-myristoylation are in membrane anchoring and protein trafficking [88].

**Figure 11:**
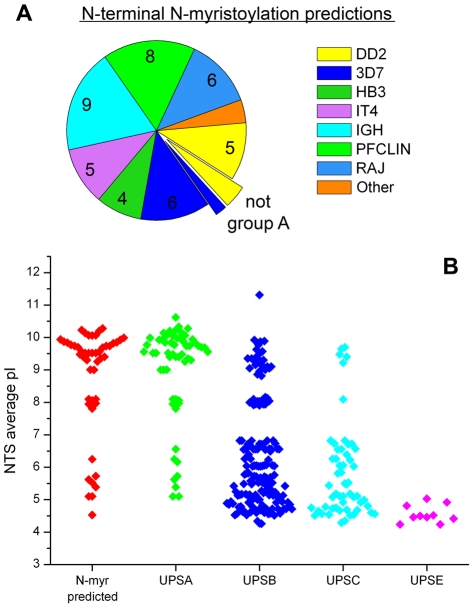
N-terminal N-myristoylation predictions. (A) 48 positive NMT predictions in 311 PfEMP1 N-terminals. All except three were group A PfEMP1. According to the predictions, the post-translational modification was well conserved in all seven genomes. (B) Average pI of NTS in 311 PfEMP1. Three groups (basic, neutral and acidic) can be clearly distinguished.

N-myristoylation is conserved across eukaryotic species [89], and several experimentally confirmed N-terminally myristoylated proteins in *P. falciparum* share the common eukaryotic motif MGxxx[S/T] [90]–[94]. The myristoylation predictor NMT, which is trained on several eukaryotic species including protozoans [95]–[96], correctly predicts that the terminals of these five experimentally analyzed *P. falciparum* proteins are N-myristoylated. The two homology blocks, HB155 and HB264 were present in 41 PfEMP1 N-terminals (Figure 10 NTS) that were all predicted to be N-myristoylated by the NMT predictor. Prediction results for 311 PfEMP1 sequences are summarized in Figure 11A, which shows that the N-myristoylation motif was found predominantly in group A PfEMP1. Remarkably, all seven *P. falciparum* genomes had a set of PfEMP1 with conserved N-myristoylation motifs (Figure 11A).

N-myristoylation may act as a localization signal and affect trafficking of PfEMP1, like PfGRASP which is dependent on a functional myristoylation motif for localization to the golgi apparatus through a brefeldin A independent pathway [94], [97]. PfGRASP has a terminal sequence (MGAGQTK) which is very similar to IT4var08 (MGAGQST) and RAJ116var05 (MGASQSK), the latter getting the highest score of all PfEMP1 by the NMT predictor.

It is still unknown if the PEXEL motif is cleaved and acetylated in PfEMP1, like in some other exported proteins [98]–[99]. If NTS is not removed by PEXEL cleavage, then the N-myristoylated N-terminal can be translocated across the membrane [100]–[101], and exposed on the IE surface. The unstable membrane binding caused by N-terminal N-myristoylation could by itself play a major role in mediating adherence of IE to host cell membranes. The unspecific binding of several acylated PfEMP1 to any part of a host cell (e.g. endothelial cell) membrane, possibly combined with receptor binding mediated by other parts of PfEMP1, could together form a strong interaction. A mechanism known as myristoyl switching has been found in some acylated proteins, where ligand binding induces a conformational change, regulating if the fatty acid is hidden in a hydrophobic pocket within the protein or if it is exposed for membrane interactions [102].

Stable membrane anchoring is also possible, as the N-terminals of some PfEMP1 possess several basic residues that can act in synergy with the lipid chain to bind the membrane. Generally UPSA have a higher pI (i.e. are more basic) than other PfEMP1 N-terminals (Figure 11B). Other types of less site-specific acylation, such as S-acylation at some of the many Cys residues, may also help tether the protein to the membrane [87].

The potentially affected group A PfEMP1 have been associated with severe malaria [9], [28]. Considering the implications for vaccine design, it should therefore be thoroughly investigated if any of the PfEMP1 variants are indeed myristoylated *in vivo*.

### Inter-domain homology blocks

52 homology blocks had more than 50% of their occurrences in inter-domain regions, i.e. outside defined domains. Three of the most frequent inter-domain homology blocks are shown in Figure 10-ID. The 52 inter-domain homology blocks were mainly low complexity sequences, occurring in repeats and overlapping each other. To determine the distribution of these homology blocks in PfEMP1 sequences, a NJ-tree was constructed based on ID homology block composition of the PfEMP1 (Figure 10 ID). In general the homology blocks were uniformly scattered amongst PfEMP1 sequences, although four groups were distinguished with representatives in at least 6 of the 7 genomes. VAR1 and VAR2CSA had unique conserved inter-domain sequences with low amounts of the low-complexity sequence found in many other PfEMP1, and therefore, they formed separate groups (Figure 10 ID, tree group 1 and 2). Interestingly, one cluster was defined by two unique inter-domain homology blocks, HB341 and HB295 (Figure 10 ID, group 3). This cluster of 11 group A PfEMP1 with similar DBLβ/γ containing domain composition (part of frame 9 in Figure S4A) captured all occurrences of double DBLβ domains, was represented in 6 of 7 genomes (not RAJ116), and the 3D7 genes were PFD1235w and PF11_0521, which have been linked to severe malaria and ICAM-1 binding respectively [28], [38]. The fourth distinct group was defined by HB280 (Figure 10 ID, group 4), conserved in 5 of 7 genomes (not 3D7 and HB3) and comprised 11 proteins, including among others the ICAM-1 binding associated IT4var14 (A4var) [40]. All members in the fourth group lacked other ID HBs, most were flanked by UPSB1, and 10 of 11 had the same C-terminal domain combination ending with DBLγ-DBLζ4 (Figure 3, cassette 9; Figure S4A, frame 7). The conservation of an ID region together with the semi-conserved domain architecture and UPS sequences, suggests a more recent common ancestor for genes in these groups. It will be interesting to see if the members of these groups share receptor-binding properties.

The Cys-containing M3 regions (M3A and M3AB) were found to be positionally linked to the upstream CIDR domain, while the amino acid composition correlated more highly with the downstream domain architecture. Two homology blocks were able to capture most occurrences of the two Cys-residues found after CIDRβ and γ, despite of the surrounding low-complexity sequence, seeing that a few other positions besides the Cys were conserved (Figure 8 M3).

### ATS homology blocks

Homology blocks of the conserved ATS were aligned and sorted according to domain composition, to describe variation in the intracellular part of PfEMP1 (Figure 10 ATS). ATS starts N-terminally with the transmembrane region, which was captured by HB21. The intron splice site between exon 1 and exon 2 lies immediately downstream of the transmembrane part, so the short basic stretch which follows transmembrane regions, and interacts with the negatively charged membrane phospholipids, was found in the following HB41. ATSA, which is associated with UPSA, was distinguished as sequences where HB69 and HB112 occurred simultaneously.

ATSvar1, ATSB17, and the ATS of VAR2CSA, were characterized by lacking the final three homology blocks conserved in all other ATS (Figure 10 ATS, HB46/47/51, Figure S7D).

ATSB17 was found in six group C PfEMP1, distributed in six genomes (not IT4), and containing several DBLβ/γ domains. The two *var2csa* genes in the HB3 genome had an ATSB14 more similar to the ATS of non-VAR2CSA PfEMP1, however these were truncated before the final three homology blocks. Other VAR2CSA ATS had normal length but contained unique sequences instead of the three conserved homology blocks. The five *var1* genes possessing an exon 2, were all flanked by a 3′UTR encoding the three missing homology blocks. Compared to a common ATS, ATSvar1 was missing ∼150 AA, ATSB17 was lacking ∼100 AA, whereas the ATS of VAR2CSA was missing or differed from the final 100–130 AA.

The finding that VAR1 and VAR2CSA both have a shortened ATS, could suggest that ATSvar1 is functional despite of truncation, and question the hypothesis that VAR1 exclusively exists as a pseudogene.

The final three ATS homology blocks could be a non-essential functional element in PfEMP1, for example acting as signal peptide during transport to the erythrocyte membrane, which would result in differences for VAR1, VAR2CSA, and ATSB17 PfEMP1, compared to other PfEMP1.

### Conserved homology block residues may comprise phosphorylation sites

Phosphorylation occurs mainly at three types of residues: Ser, Thr and Tyr, and all three residues were markedly conserved in several homology blocks. Phosphorylation is a common modification of proteins expressed during the erythrocyte stages, and has been associated with differences in IE adhesion properties [103]. Ser/Thr phosphorylation of the PfEMP1 ATS was recently shown to alter its association with parasite-encoded knob-associated His-rich protein (KAHRP), and to regulate cytoadherence of IE [104].

Judging from phosphorylation site predictions and conservation levels in the homology blocks, some examples of conserved potential phosphorylation sites were, in DBL domains (Figure 6): HB19 position 28 (DBLα S1), HB82 position 11 (DBLα S2b), HB36 position 8 (DBLα0 S2c), and Tyr in HB29 (DBLδ and γ S2c). In CIDR one of many examples is the mentioned YGN motif in CIDRα HB32 (Figure 8). Several sites of all three types are conserved in the ATS HB41, HB43, and HB69 (Figure 10 ATS). Phosphorylation sites have been predicted for all PfEMP1 sequences, and the conservation of these can be inspected for each homology block on the VarDom server.

It will be interesting to see if some of these sites are surface-exposed and thus accessible to kinases, as the introduction of large, negatively charged phosphate groups could result in conformational changes, or contribute to charged binding surfaces, and thus result in functional and antigenic variation.

### Overall PfEMP1 homology block architecture

**Figure 12:**
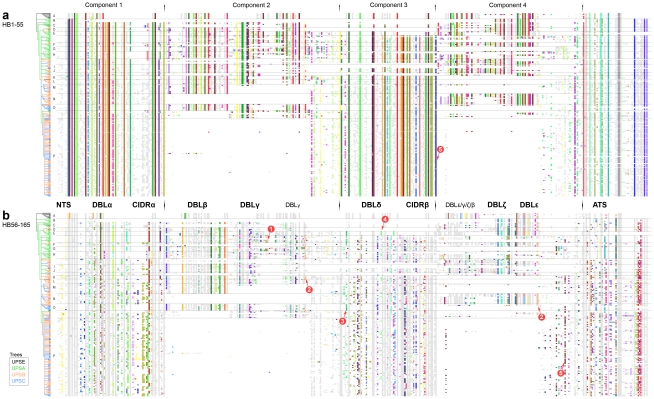
PfEMP1 homology block alignment. (**a**) and (**b**) are the same alignment, with HB1–55 colored in (a), and HB56–165 colored in (b). The sequences are sorted according to HB composition, and the tree is colored according to UPS class. The division of PfEMP1 into four components is indicated at the top of the figure. Between (a) and (b) is noted the most prevalent major domain class for that area in the alignment. The five core homology blocks should be distinguishable in (a), as well as less frequent homology blocks especially in (b). The alignment with all details can be found in Figure S7E, and the labeled tree in Figure S8. Alignment features (red arrows): (**1**) DBLγ-β hybrid domains; (**2**) The light orange column is HB78, present in both DBLγ and DBLε (Figure 6n, r, and t) and associated with C-terminal of comp. 2 and 4; (**3**) HB74 in DBLγ-like DBLδ domains, as in Figure 6g, h and Figure 7-S1, S2; (**4**) HB82 in DBLγ8 of VAR1, also found in DBLδ domains; and (**5**) M3 homology blocks. Notable clades in the tree: (**A**) VAR2CSA; (**B**) VAR3; (**C**) bootstrap 28%, 4 genomes, UPSA3, includes IT4var60 (rosetting); (**D**) bootstrap 25%, 3 genomes, incl. PFL0020w and PF08_0141; (**E**) VAR1; (**F**) 6 genomes, incl. MAL6P1.4; (**G**) 5 genomes, incl. PFD1235w and PF11_0521 (ICAM-1); (**H**) 5 genomes, incl. PF11_0008 and PF13_0003; (**I**) 4 genomes, incl. PF07_0050 and IT4var31 (CD36, ICAM-1); (**J**) 4 genomes, incl. IT4var14 (CD36, ICAM-1); (**K**) bootstrap 27%, 5 genomes, UPSB2, incl. PF08_0140 and IT4var06; (**L**) bootstrap 26%, 3 genomes, incl. IT4var16 (CD36, ICAM-1) and IT4var27 (rosetting); (**M**) bootstrap 18%, all genomes incl. MAL6P1.252 and PFL1950w; (**N**) bootstrap 68%, 5 genomes, UPSB; (**O**) bootstrap 49%, 5 genomes, UPSC1, incl. IT4var01 (rosetting) and TM284S2var1 (rosetting, IgG); and (**P**) Comp.1-Comp.3-ATS architecture (a.k.a. Type 1 *var*), UPSB and UPSC.

Homology block sequences of full-length PfEMP1 were aligned, to determine HB associations with specific positions in the whole proteins, as well as to find groups of PfEMP1 with similar HB compositions. Sequences were sorted according to NJ-clustering based on Manhattan distances between feature vectors consisting of exon 1 HB counts. The homology block alignment shown in Figure 12 gives a detailed overview of the diversity and structure in the PfEMP1 family. A labeled version of the alignment and the tree can be found in Figure S7E and Figure S8, respectively.

The differences between UPSA, B, and C flanked *var* genes were not clear enough to form separate clades in the tree, though homology blocks specific for UPSA-flanked *var* were observed in both ends of the alignment (Figure 12a comp.1 and Figure 12b ATS). The three conserved genes were all clearly distinguished (Figure 12,clade A, B, and E), as well as many small PfEMP1 groups, generally with low bootstrap support, as expected from uncorrelated domains in N- and C-terminal (Figure S4).

A list of homology blocks specific for each of the four components are summarized in Table S2. These specific homology blocks may be helpful for functional analysis of the PfEMP1, as well as for genotyping purposes.

### Conclusion

The reclassification of PfEMP1 domains by alignment and distance tree analysis introduced a few larger and several smaller new subclasses. Although the classification is a result of a phylogenetic approximation of the different evolutionary histories of the domain sequence blocks, identification of conserved PfEMP1 domain architectures was possible. These structures represent a novel perspective on the PfEMP1 architecture. DBL and CIDR domains appear to be inherited in conserved domain structures that to a large extent fall within four major components. These conserved domain structures although large and complex may well represent functional units of the whole PfEMP1 molecule.

Apart from the known conserved *var* genes, *var1*, *var2csa*, and *var3*, 18 domain cassettes and several less well-defined structural phenomena were observed for the seven sequenced genomes. The established division of group A, B and C was confirmed although importantly, N- and C-terminal conserved domain structures occurred independently of each other, with distinct C-terminal DBLε-containing structures transcending the three conserved genes, as well as group A, B, and C.

Homology blocks covering on average 83.5% of a PfEMP1 sequence were defined, describing the PfEMP1 family on a more detailed level than domains, yet more simplified than the amino acid level. Local similarities between domain classes were thus described, and homology blocks specific for PfEMP1 domain classes, components, and cassettes, were found. The HB analysis also revealed a recombination hotspot between subdomain S2 and S3 in DBL domains, which has helped shape the antigen repertoire. Thus, several DBL domains are hybrids of different major classes - an observation important for functional studies as well as antibody cross-reactivity and vaccine design.

Several conserved elements were described by the homology blocks, including: (1) DBL domain core interactions conserved in all DBL domains, holding the subdomains together, (2) an acylation motif found to be conserved in group A *var* genes, suggesting N-terminal N-myristoylation of a subset of PfEMP1, (3) conserved residues predicted to be phosphorylation sites, and (4) PfEMP1 inter-domain regions, which are proposed to be elastic disordered structures.

The novel iterative homology block detection method is potentially applicable to any protein dataset, and would be especially suitable for compositional analysis of other frequently recombining gene families.

The VarDom server was introduced, where all presented information on domain classes and homology blocks can be retrieved, and new sequences can be classified and related to other PfEMP1 proteins in the seven genomes. Ideally, the server will allow better interpretation and facilitate the development of new approaches in PfEMP1 research. For example analysis of *var* expression data from microarrays and short high through-put sequence reads or the design of recombinant proteins for immunizations or functional studies could all benefit from this detailed account of PfEMP1 diversity and ultimately aid the development of PfEMP1 based malaria interventions.

## Methods

### Datasets

Annotated *var* genes and *var* gene containing contigs were retrieved using BLAST, from NCBI nucleotide database and from genome assemblies of *P. falciparum* clones 3D7, HB3, DD2, IT4/FCR3, PFCLIN, RAJ116, IGH and *P. Reichenowi* clone PREICH at PlasmoDB, Broad and Sanger Institute servers, querying 3D7 *var* sequences. For all *var* genes with intact N-terminal segments, 2000 bp 5′ UTRs were also retrieved where possible. In total 399 annotated genes and open reading frames spanning over the length of at least two DBL/CIDR domains were kept for the sequence alignment and distance tree analysis, whereas the homology block dataset consisted of the 311 full length or exon1 sequences, as well as 20 DBL-containing paralogs from *Plasmodium falciparum*, *vivax*, *yoelii* and *knowlesi*. For meaningful interpretations, the first approach required sequence lengths spanning at least two PfEMP1 features, whereas the latter, was based on whole or exon1 sequences to avoid generating false homology block break-points. Nucleotide sequences of all *var* genes analyzed in this study are available in Dataset S1.

### Domain alignment and phylogeny

Large phylogenies comprising all DBL or CIDR sequences were inferred by multiple sequence alignment using MUSCLE (version 3.7) followed by application of the neighbor-joining algorithm implemented in MEGA (version 4.0.2) [105]. Major domain classes were deduced and named according to previously defined classes [10].

Major domain-class sequences were further subclassified through a recursive process involving: (1) re-alignment of sequences, (2) construction of a maximum likelihood tree, and (3) split of sequences into two clusters at a tree bipartition validated by at least 50% of the bootstraps. If a suitable bipartition was found, the process would be repeated for each of the two formed clusters. If the sequences on the other hand were not divided, they were all assigned to the same subclass and given a number. In addition to bootstrap support, two other properties were used to evaluate bipartitions and determine if and where the trees should be split: the number of genomes represented in each cluster, and the within-cluster average distance (WCAD), which was used as a measure for the relatedness of clustered sequences. See Text S1 for details on domain border and distance tree cluster definitions.

Multiple sequence alignments of PfEMP1 domains were performed with AQUA [106], which optimizes alignments generated by MUSCLE (version 3.7) [107] and MAFFT (version 6.611b) [108], using refinement and evaluation implemented in RASCAL (version 1.34) [109] and NORMD (version 1.3) [110], respectively. Maximum likelihood trees were built using the multithreaded version (pthreads) of RAxML (version 7.2.5) [111]–[112]. The gamma model for substitution rate heterogeneity was used together with the WAG [113] amino acid substitution model with empirically determined amino acid frequencies. WAG and JTT [114] were found to be the most likely substitution models by fitting of models implemented in RAxML to fixed trees built from the different domain alignments and subsequent ML comparison. Within-cluster average distances were based on distances calculated using the JTT model implemented in Protdist from the PHYLIP package (version 3.69) [115].

### Upstream sequences

Sequences were aligned with MAFFT (version 6.240) using the L-INS-i algorithm for multiple sequence alignment [108]. A neighbor-joining tree was generated and bootstrapped using Clustalw (version 2.0.9 for tree construction and version 1.83 for bootstrapping because version 2.0.9 crashed during bootstrap) [116].

Sequences were clustered using the Markov clustering algorithm (version 08-312) [51]–[53]. The Markov clustering algorithm is a graph-theoretical clustering method, which uses an all-against-all pairwise sequence alignment as input, generated with the blastn algorithm implemented in blastall (version 2.2.18) [117]. The inflation parameter of the Markov Cluster Algorithm was varied in steps of 0.2 from 1.2 to 5.0, and resource scheme 7 (most accurate) was used. A distinct clustering was generated for each value of the inflation parameter, and all the clusters were summarized in a consensus clustering. Briefly, each clustering was converted to a multifurcating tree with a branch representing each cluster. A consensus tree representing the consensus clustering was then constructed, using the majority rule consensus method (include all bipartitions with a frequency larger than 0.5) [118], with the extension that less frequent bipartitions were also included as long as they continued to resolve the tree and did not contradict more frequent groups. Based on the results of the two clustering methods, a consensus annotation of the 5′ upstream sequences of the *var* genes was reached (Figure S5).

Trees were rendered and edited using Dendroscope (version 2.3) [119].

### Homology block alignment and trees

The iterative homology search procedure used for defining the set of 628 homology blocks is described in Text S2.

Alignment of homology blocks was performed with a python implementation of the Smith-Waterman algorithm with linear (non-affine) gap penalty and a substitution matrix of the identity type [120].

To estimate trees based on homology block composition, homology block feature vectors were constructed for each sequence, either binary (DBL, CIDR, ATS, ID and NTS trees) or with counts (PfEMP1 tree), and accordingly distances were calculated as either Hamming or Manhattan distances. Trees were constructed as extended 50% majority rule consensus trees, based on 1000 neighbor joining bootstrap trees, built from distance matrix using ordinary neighbor joining implemented in Clearcut (version 1.0.8) [121].

Sequence logos were generated using WebLogo (version 2.8) [122], where small sample (<40 amino acids) bias is compensated for by subtraction of an error estimate on each position, the error bars are 2 times the estimated error.

### Prediction of phosphorylation sites and N-terminal N-myristoylation

Phosphorylation sites were predicted using NetPhos 2.0 [123]. N-terminal N-myristoylation was predicted with the NMT myristoylation predictor which is trained for several eukaryotic species including protozoans [95]–[96].

## Supporting Information

Dataset S1 ***Var*** **gene sequences.** *Var* gene cDNA encoding the PfEMP1 analyzed in this study. Sequence names in this fasta-file are the same as used everywhere else in this study, as well as on the VarDom server.
(2.84 MB TXT)

Figure S1 **Major DBL and CIDR domain classes.** (A) NJ tree based on amino acid alignment of 1242 DBL sequences. Blue dots mark branches dividing DBL domains into six major groups and four N-terminal VAR2CSA DBL classes. (B) NJ tree based on amino acid alignment of 655 CIDR sequences. Blue dots mark branches dividing CIDR domains into four major groups as well as the CIDRα1 and CIDRpam subclasses. Leaf names are omitted from the figure to improve graphical presentation.
(2.49 MB PNG)

Figure S2 **Trees showing subclassification of all major PfEMP1 domain classes.** ML trees based on amino acid alignments of each of the following domain classes are shown in panels **A–M**: DBLα0, α1, β, δ, ε, γ, ζ; CIDRα, β, γ, δ; NTS; ATS. Sequence names as well as start and stop position of the domains are given in the trees, followed by classification of the domain. Panel **N** and **O**: Assignment of sequences to UPS groups by Markov clustering (N) and neighbor joining (O). The UPS groups were named as indicated by the text color. The background colors show the group membership assigned by Kraemer *et al.* 2007 [16]. Sequences found upstream of domain cassette 8 (Figure 3) are marked with black squares. (**N**) The branch labels show the fraction of Markov clusters with this group present. (**O**) The branch labels show the bootstrap values as fractions of 1000 bootstraps. Monophyletic subgroups with a bootstrap support above 0.7 and containing sequences from at least four different strains of *P. falciparum* are highlighted with thick red branches. Some subgroups were further expanded (without bootstrap support) to form larger monophyletic groups: UPSA2 and UPSB3 are expanded to include additional sequences annotated to UPSA2 and UPSB3 respectively by Kraemer *et al.* 2007 [16], UPSB2 is expanded to include two genes with same domain architecture, and UPSC1 is expanded to include three sequences that fall between UPSC1 and UPSC2 but within the larger monophyletic group comprising all UPSC sequences. The sequences are shown with thick black branches. The additional sequences included by this expansion are denoted with an asterisk in the annotation in Figure S4 and S5. UPSA3 and UPSB1 are groups that contain all the sequences not assigned to any other subgroup in UPSA and UPSB respectively. ND: Not Determined.
(1.11 MB ZIP)

Figure S3 **PfEMP1 domain class logos.** Sequence conservation logos for major PfEMP1 domain classes (panel A–Z): CIDRα, α1, α2, α3, β, δ, γ, pam; DBLα0, α1 (without α1.3), α1.3, β, δ, ε (without ε1, ε2, ε11, ε13, εpam), ε1, ε2, ε11, ε13, εpam4, εpam5, γ, pam1, pam2, pam3, ζ; NTSA, NTSB, and M3AB.
(2.42 MB ZIP)

Figure S4 **Annotated PfEMP1 sequences aligned according to C-terminal (A) and N-terminal (B) domain compositions.** Gene names, parasite genome, 5′ UPS classes, PfEMP1 domain annotation (D = domain, ID = Inter Domain) and origin of sequence data (if sequence is not previously reported as *var* gene) are given. Sequences which partially contain unexpected identical sequence stretches to other sequences suggesting an incorrect contig assembly are noted “HBD” followed by the name of the potentially redundant sequence. Red arrows indicate component 1–4. Frames indicate clusters of correlated domain classes. 1:VAR1; 2: VAR2CSA; 3: VAR3; 4: DBLζ and DBLε domain combinations of component 4; 5: Cassette 10; 6: Cassette 6; 7: Cassette 9; 8: Cassette 5; 9: Other Group A PfEMP1 all containing component 2; 10: Cassette 8; 11: Group B and C genes containing component 2; 12: Group B and C PfEMP1 with no component 2 or 4; 13: Cassette 14; 14: Cassette 17,21 and 22; 15: DBLα1-CIDR subclass correlations including cassette 11,13,15 and 16; 16: DBLα0 subclasses associated with CIDRα3 subclasses; 17: DBLα0 subclasses associated with CIDRα2 subclasses. N-terminal segment (NTS), Duffy binding-like (DBL), Cys-rich inter-domain region (CIDR) and acidic terminal segment (ATS) are named according to the distance tree classification. Inter domains are annotated as either short if <32 AA (green) or long if >31 (yellow) and “A” or “B” if encoding M3A or M3AB.
(0.15 MB PDF)

Figure S5 **Schematic representation of annotated** ***var*** **genes sorted by genome origin.** Gene names, 5′UTR class, domain architecture and origin of sequence data (if sequence is not previously reported as *var* gene) is given. Sequences are noted “F” (Fragment) in comments if predicted not to span a full length exon1, and “HBD” if incorrect contig assembly is suspected followed by the name of the sequence which partially contains unexpected identical sequence stretches. N-terminal segment (NTS), Duffy binding-like (DBL), Cys-rich inter-domain region (CIDR) and acidic terminal segment (ATS) are named according to the distance tree classification.
(0.05 MB PDF)

Figure S6 **Phylogenetic trees for DBL subdomains S1, S2 and S3, as in** **Figure 7** **but with labels.** Edge values are fractions of 1000 bootstraps, and each subdomain is given as: protein name, start position, end position, and the domain class the subdomain is a part of.
(0.22 MB PDF)

Figure S7 **Homology block alignments.** Homology block alignments for (panel A–E): DBL, CIDR, NTS, ATS, and whole PfEMP1, with details of Figure 6, Figure 8, Figure 10 and Figure 12.
(0.82 MB ZIP)

Figure S8 **Tree in** **Figure 12** **with labels.** Bootstrap values are given as fractions of 1000 bootstraps.
(0.33 MB PDF)

Table S1 **Examples of HB combinations specific for DBL and CIDR domain classes.** Domain counts and number of matches of the HB combination are given for the sequence set with 311 PfEMP1 sequences. The domain combination (17, 19) signifies a sequence where both HB17 and HB19 are present. These homology blocks are suggested for use in oligonucleotide array design, as well as for functional analysis of the domain types. The list is not exhaustive, and can be supplemented using Figure 6 and Figure 8, as well as the VarDom server.
(0.11 MB PDF)

Table S2 **Homology blocks specific for component 1–4 (** **Figure 12** **).** Homology block numbers are given in parenthesis, and number of occurrences in the component with 311 sequences, is given next to the number of occurrences elsewhere. These homology blocks are suggested for use in oligonucleotide array design, as well as for functional analysis of the components. The table is not exhaustive.
(0.09 MB PDF)

Text S1 **PfEMP1 domain classification by alignment and distance tree analysis.**
(0.15 MB PDF)

Text S2 **Defining PfEMP1 homology blocks.**
(0.81 MB PDF)

Text S3 **PfEMP1 DBL domain relations to CIDR and paralog DBL domains.**
(0.31 MB PDF)
