# Supplementary figures and images for: Plasmodium falciparum Erythrocyte Membrane Protein 1 Diversity in Seven Genomes – Divide and Conquer
Source: PLoS Comput Biol. 2010 Sep 16;6(9):e1000933. doi: 10.1371/journal.pcbi.1000933 (PMC2940729; doi:10.1371/journal.pcbi.1000933)

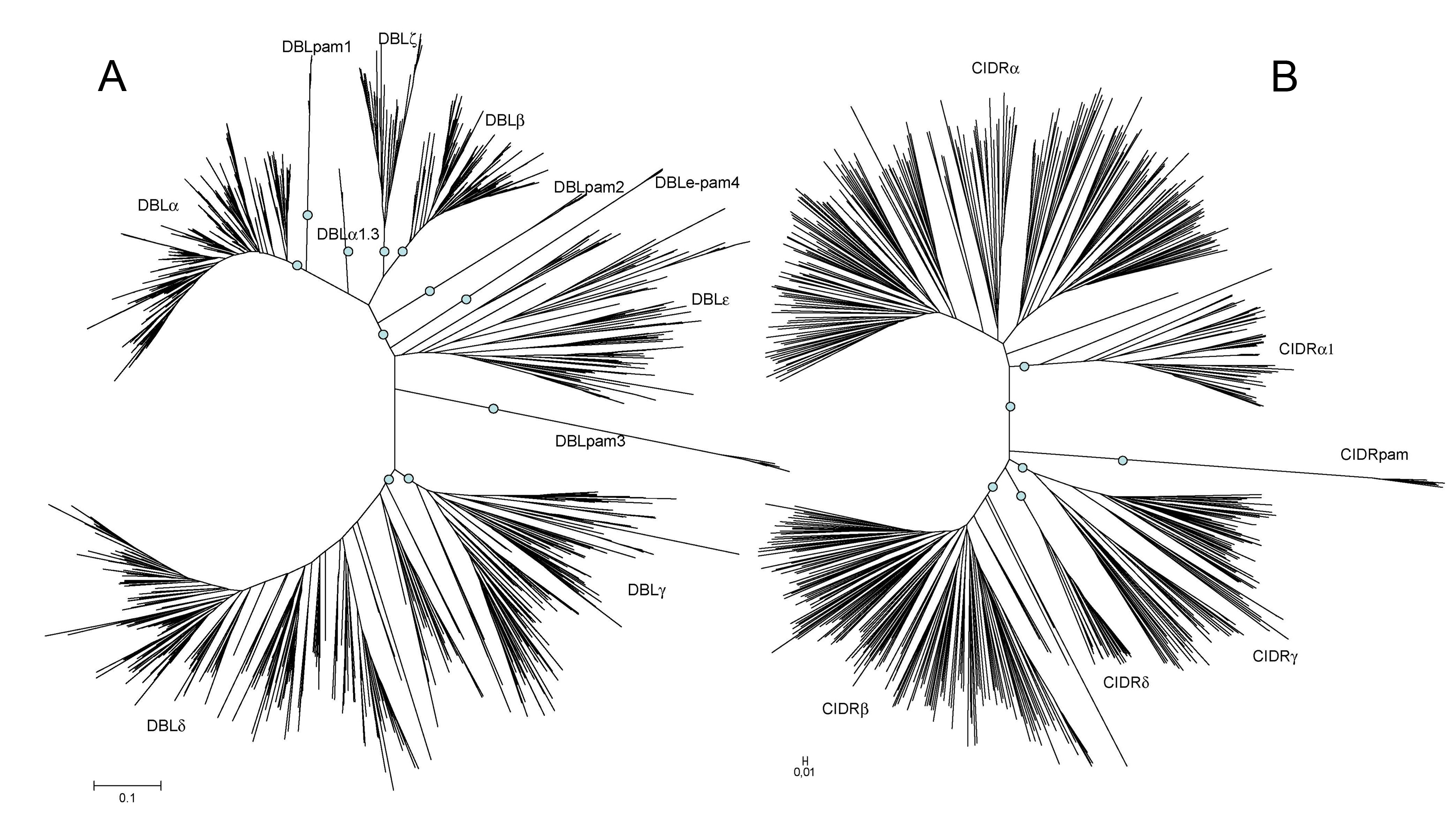

Supplement: Figure S1 — Major DBL and CIDR domain classes. (A) NJ tree based on amino acid alignment of 1242 DBL sequences. Blue dots mark branches dividing DBL domains into six major groups and four N-terminal VAR2CSA DBL classes. (B) NJ tree based on amino acid alignment of 655 CIDR sequences. Blue dots mark branches dividing CIDR domains into four major groups as well as the CIDRα1 and CIDRpam subclasses. Leaf names are omitted from the figure to improve graphical presentation. (2.49 MB PNG) [file pcbi.1000933.s002.png]

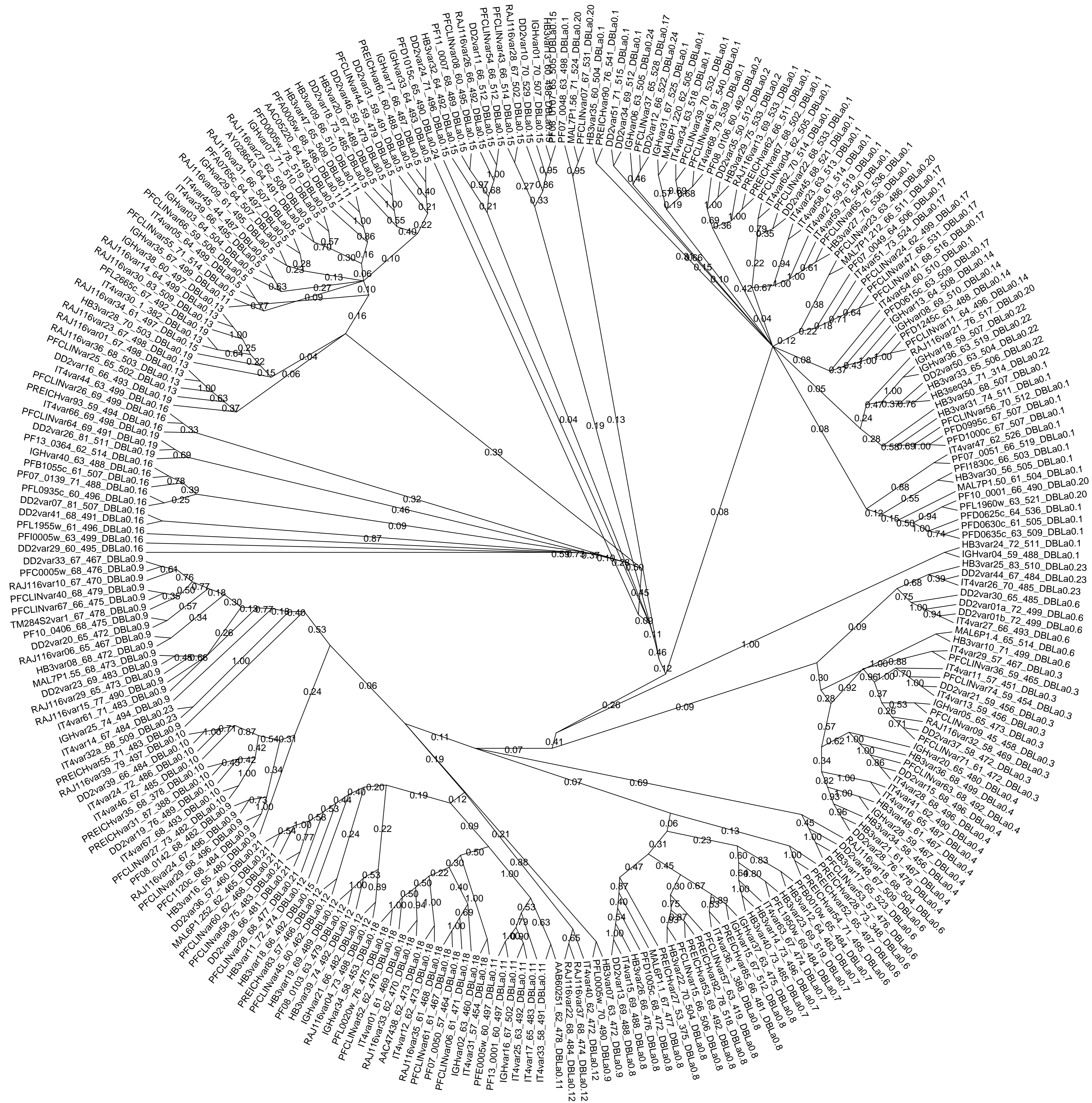

Supplement: Figure S2 — Trees showing subclassification of all major PfEMP1 domain classes. ML trees based on amino acid alignments of each of the following domain classes are shown in panels A–M: DBLα0, α1, β, δ, ε, γ, ζ; CIDRα, β, γ, δ; NTS; ATS. Sequence names as well as start and stop position of the domains are given in the trees, followed by classification of the domain. Panel N and O: Assignment of sequences to UPS groups by Markov clustering (N) and neighbor joining (O). The UPS groups were named as indicated by the text color. The background colors show the group membership assigned by Kraemer et al. 2007 [16]. Sequences found upstream of domain cassette 8 (Figure 3) are marked with black squares. (N) The branch labels show the fraction of Markov clusters with this group present. (O) The branch labels show the bootstrap values as fractions of 1000 bootstraps. Monophyletic subgroups with a bootstrap support above 0.7 and containing sequences from at least four different strains of P. falciparum are highlighted with thick red branches. Some subgroups were further expanded (without bootstrap support) to form larger monophyletic groups: UPSA2 and UPSB3 are expanded to include additional sequences annotated to UPSA2 and UPSB3 respectively by Kraemer et al. 2007 [16], UPSB2 is expanded to include two genes with same domain architecture, and UPSC1 is expanded to include three sequences that fall between UPSC1 and UPSC2 but within the larger monophyletic group comprising all UPSC sequences. The sequences are shown with thick black branches. The additional sequences included by this expansion are denoted with an asterisk in the annotation in Figure S4 and S5. UPSA3 and UPSB1 are groups that contain all the sequences not assigned to any other subgroup in UPSA and UPSB respectively. ND: Not Determined. (1.11 MB ZIP) [file pcbi.1000933.s003.zip › Figure S2A - Tree DBLa0.pdf]

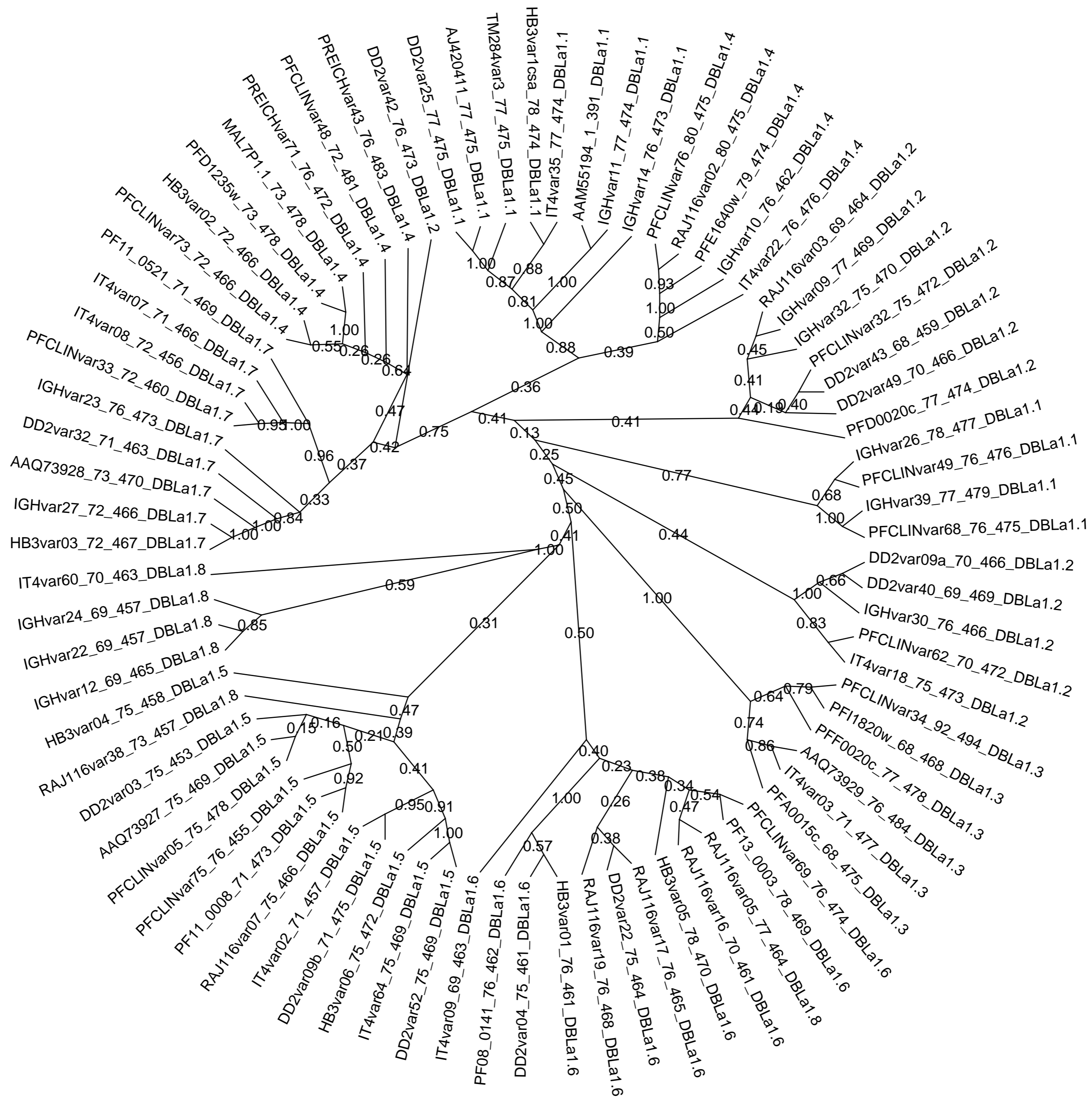

Supplement: Figure S2 — Trees showing subclassification of all major PfEMP1 domain classes. ML trees based on amino acid alignments of each of the following domain classes are shown in panels A–M: DBLα0, α1, β, δ, ε, γ, ζ; CIDRα, β, γ, δ; NTS; ATS. Sequence names as well as start and stop position of the domains are given in the trees, followed by classification of the domain. Panel N and O: Assignment of sequences to UPS groups by Markov clustering (N) and neighbor joining (O). The UPS groups were named as indicated by the text color. The background colors show the group membership assigned by Kraemer et al. 2007 [16]. Sequences found upstream of domain cassette 8 (Figure 3) are marked with black squares. (N) The branch labels show the fraction of Markov clusters with this group present. (O) The branch labels show the bootstrap values as fractions of 1000 bootstraps. Monophyletic subgroups with a bootstrap support above 0.7 and containing sequences from at least four different strains of P. falciparum are highlighted with thick red branches. Some subgroups were further expanded (without bootstrap support) to form larger monophyletic groups: UPSA2 and UPSB3 are expanded to include additional sequences annotated to UPSA2 and UPSB3 respectively by Kraemer et al. 2007 [16], UPSB2 is expanded to include two genes with same domain architecture, and UPSC1 is expanded to include three sequences that fall between UPSC1 and UPSC2 but within the larger monophyletic group comprising all UPSC sequences. The sequences are shown with thick black branches. The additional sequences included by this expansion are denoted with an asterisk in the annotation in Figure S4 and S5. UPSA3 and UPSB1 are groups that contain all the sequences not assigned to any other subgroup in UPSA and UPSB respectively. ND: Not Determined. (1.11 MB ZIP) [file pcbi.1000933.s003.zip › Figure S2B - Tree DBLa1.pdf]

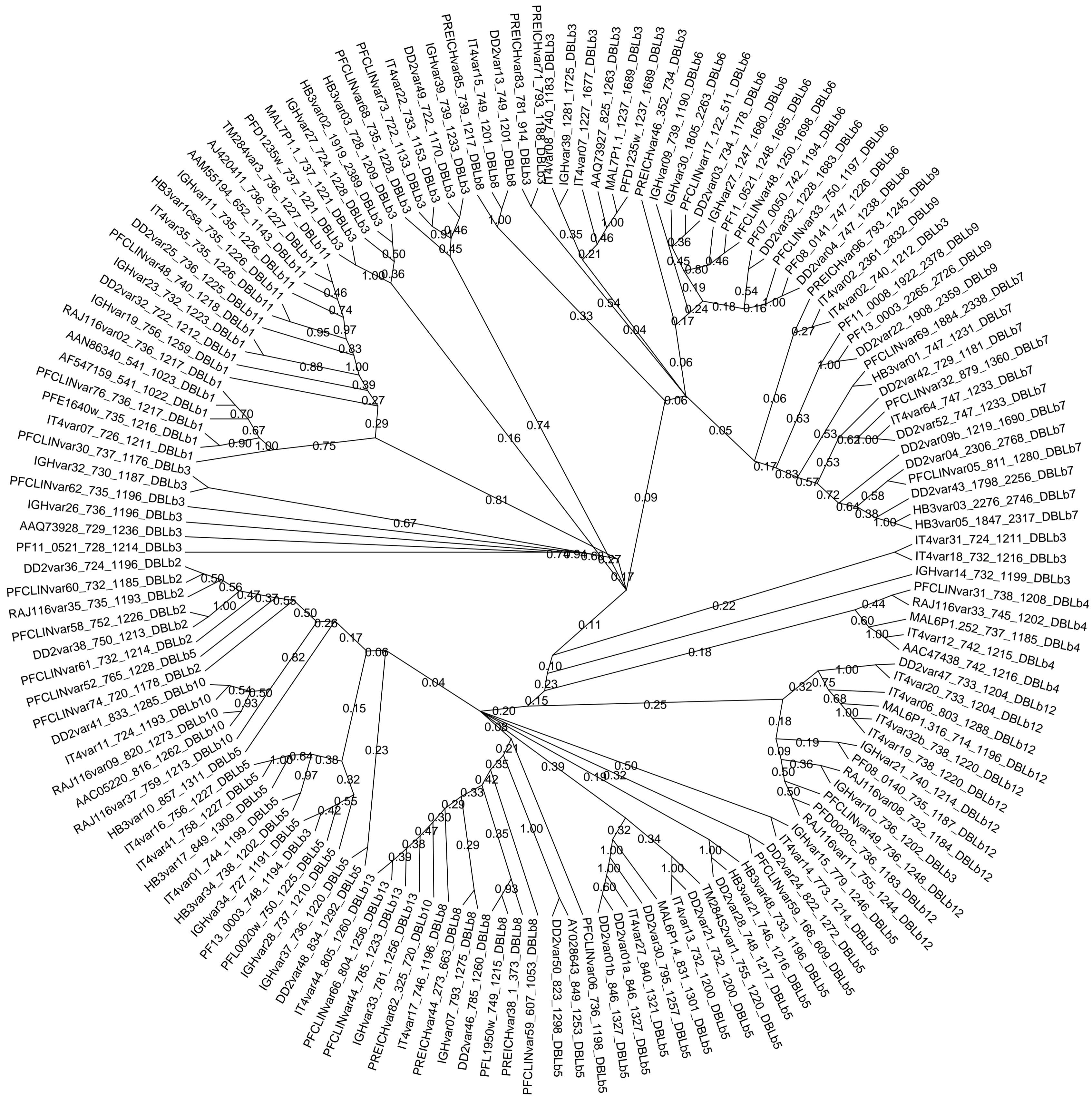

Supplement: Figure S2 — Trees showing subclassification of all major PfEMP1 domain classes. ML trees based on amino acid alignments of each of the following domain classes are shown in panels A–M: DBLα0, α1, β, δ, ε, γ, ζ; CIDRα, β, γ, δ; NTS; ATS. Sequence names as well as start and stop position of the domains are given in the trees, followed by classification of the domain. Panel N and O: Assignment of sequences to UPS groups by Markov clustering (N) and neighbor joining (O). The UPS groups were named as indicated by the text color. The background colors show the group membership assigned by Kraemer et al. 2007 [16]. Sequences found upstream of domain cassette 8 (Figure 3) are marked with black squares. (N) The branch labels show the fraction of Markov clusters with this group present. (O) The branch labels show the bootstrap values as fractions of 1000 bootstraps. Monophyletic subgroups with a bootstrap support above 0.7 and containing sequences from at least four different strains of P. falciparum are highlighted with thick red branches. Some subgroups were further expanded (without bootstrap support) to form larger monophyletic groups: UPSA2 and UPSB3 are expanded to include additional sequences annotated to UPSA2 and UPSB3 respectively by Kraemer et al. 2007 [16], UPSB2 is expanded to include two genes with same domain architecture, and UPSC1 is expanded to include three sequences that fall between UPSC1 and UPSC2 but within the larger monophyletic group comprising all UPSC sequences. The sequences are shown with thick black branches. The additional sequences included by this expansion are denoted with an asterisk in the annotation in Figure S4 and S5. UPSA3 and UPSB1 are groups that contain all the sequences not assigned to any other subgroup in UPSA and UPSB respectively. ND: Not Determined. (1.11 MB ZIP) [file pcbi.1000933.s003.zip › Figure S2C - Tree DBLb.pdf]

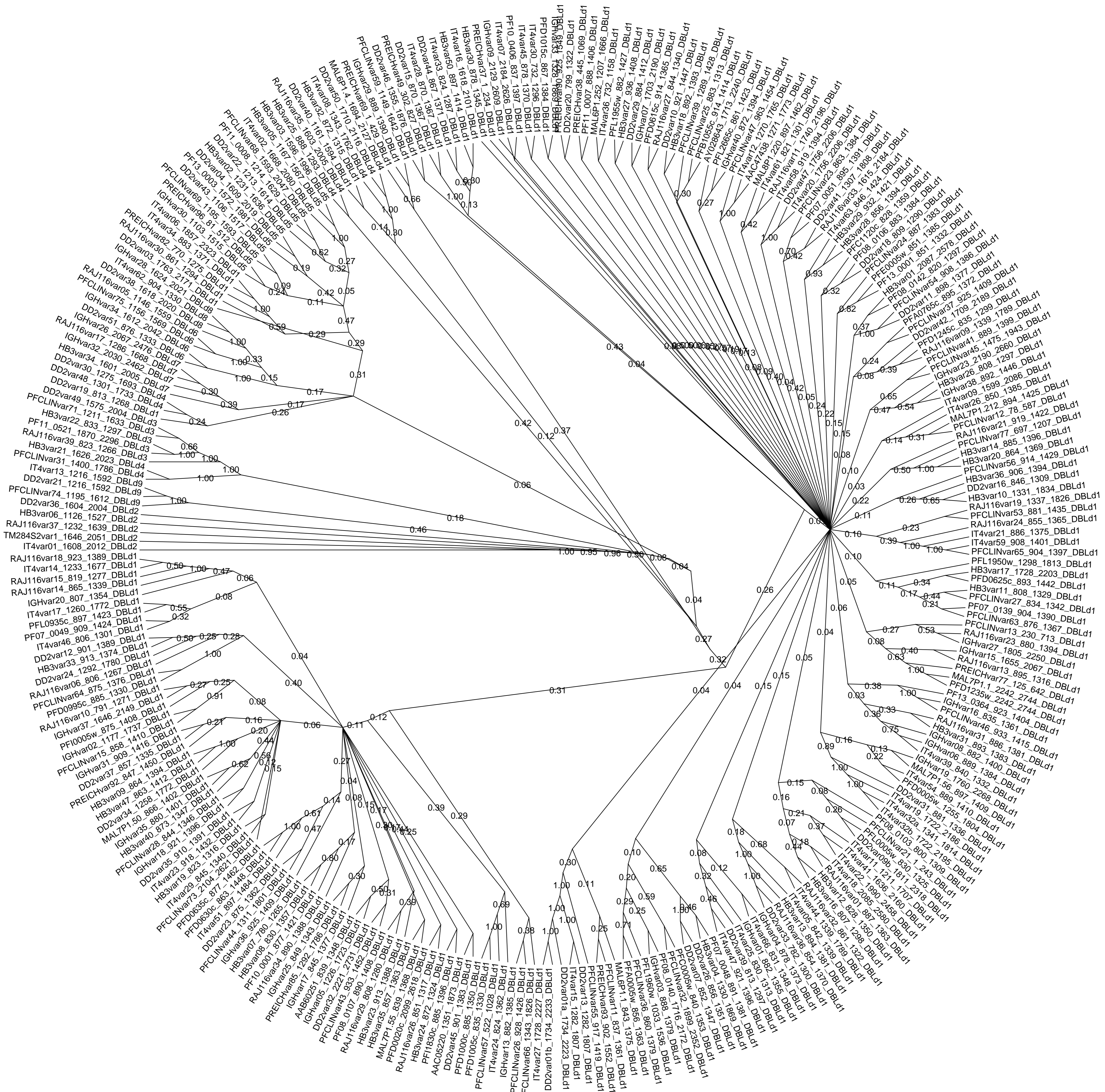

Supplement: Figure S2 — Trees showing subclassification of all major PfEMP1 domain classes. ML trees based on amino acid alignments of each of the following domain classes are shown in panels A–M: DBLα0, α1, β, δ, ε, γ, ζ; CIDRα, β, γ, δ; NTS; ATS. Sequence names as well as start and stop position of the domains are given in the trees, followed by classification of the domain. Panel N and O: Assignment of sequences to UPS groups by Markov clustering (N) and neighbor joining (O). The UPS groups were named as indicated by the text color. The background colors show the group membership assigned by Kraemer et al. 2007 [16]. Sequences found upstream of domain cassette 8 (Figure 3) are marked with black squares. (N) The branch labels show the fraction of Markov clusters with this group present. (O) The branch labels show the bootstrap values as fractions of 1000 bootstraps. Monophyletic subgroups with a bootstrap support above 0.7 and containing sequences from at least four different strains of P. falciparum are highlighted with thick red branches. Some subgroups were further expanded (without bootstrap support) to form larger monophyletic groups: UPSA2 and UPSB3 are expanded to include additional sequences annotated to UPSA2 and UPSB3 respectively by Kraemer et al. 2007 [16], UPSB2 is expanded to include two genes with same domain architecture, and UPSC1 is expanded to include three sequences that fall between UPSC1 and UPSC2 but within the larger monophyletic group comprising all UPSC sequences. The sequences are shown with thick black branches. The additional sequences included by this expansion are denoted with an asterisk in the annotation in Figure S4 and S5. UPSA3 and UPSB1 are groups that contain all the sequences not assigned to any other subgroup in UPSA and UPSB respectively. ND: Not Determined. (1.11 MB ZIP) [file pcbi.1000933.s003.zip › Figure S2D - Tree DBLd.pdf]

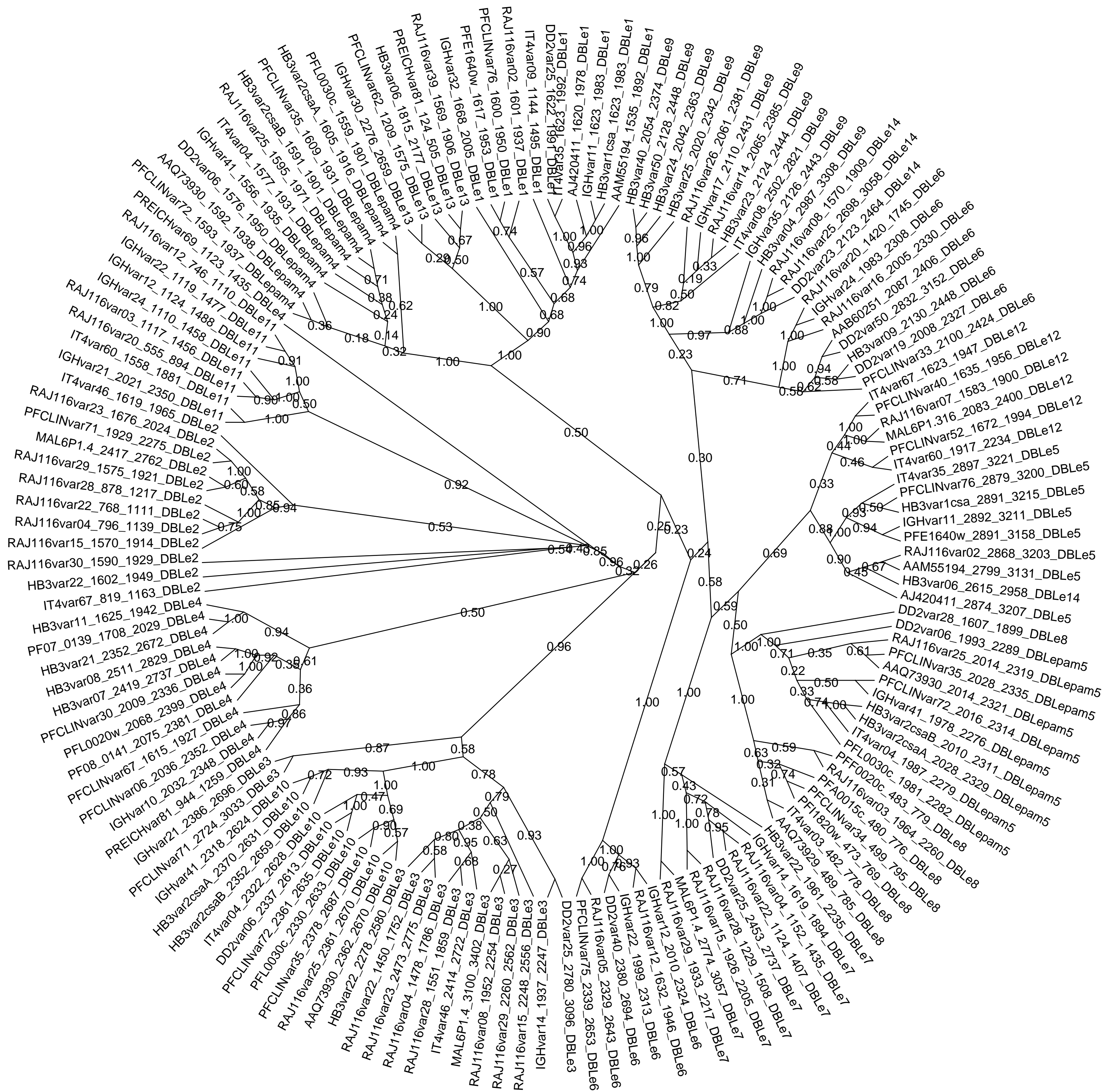

Supplement: Figure S2 — Trees showing subclassification of all major PfEMP1 domain classes. ML trees based on amino acid alignments of each of the following domain classes are shown in panels A–M: DBLα0, α1, β, δ, ε, γ, ζ; CIDRα, β, γ, δ; NTS; ATS. Sequence names as well as start and stop position of the domains are given in the trees, followed by classification of the domain. Panel N and O: Assignment of sequences to UPS groups by Markov clustering (N) and neighbor joining (O). The UPS groups were named as indicated by the text color. The background colors show the group membership assigned by Kraemer et al. 2007 [16]. Sequences found upstream of domain cassette 8 (Figure 3) are marked with black squares. (N) The branch labels show the fraction of Markov clusters with this group present. (O) The branch labels show the bootstrap values as fractions of 1000 bootstraps. Monophyletic subgroups with a bootstrap support above 0.7 and containing sequences from at least four different strains of P. falciparum are highlighted with thick red branches. Some subgroups were further expanded (without bootstrap support) to form larger monophyletic groups: UPSA2 and UPSB3 are expanded to include additional sequences annotated to UPSA2 and UPSB3 respectively by Kraemer et al. 2007 [16], UPSB2 is expanded to include two genes with same domain architecture, and UPSC1 is expanded to include three sequences that fall between UPSC1 and UPSC2 but within the larger monophyletic group comprising all UPSC sequences. The sequences are shown with thick black branches. The additional sequences included by this expansion are denoted with an asterisk in the annotation in Figure S4 and S5. UPSA3 and UPSB1 are groups that contain all the sequences not assigned to any other subgroup in UPSA and UPSB respectively. ND: Not Determined. (1.11 MB ZIP) [file pcbi.1000933.s003.zip › Figure S2E - Tree DBLe.pdf]

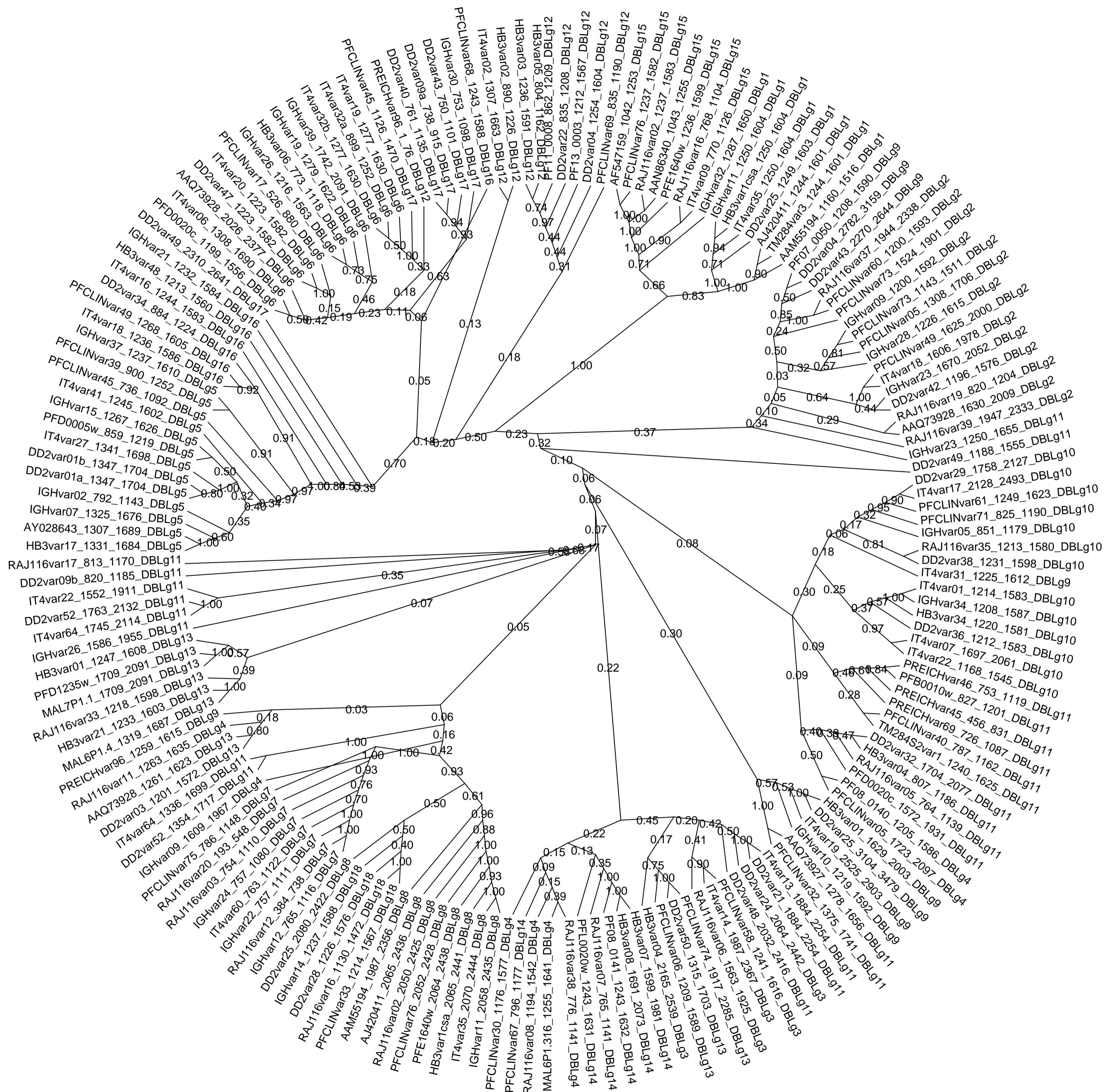

Supplement: Figure S2 — Trees showing subclassification of all major PfEMP1 domain classes. ML trees based on amino acid alignments of each of the following domain classes are shown in panels A–M: DBLα0, α1, β, δ, ε, γ, ζ; CIDRα, β, γ, δ; NTS; ATS. Sequence names as well as start and stop position of the domains are given in the trees, followed by classification of the domain. Panel N and O: Assignment of sequences to UPS groups by Markov clustering (N) and neighbor joining (O). The UPS groups were named as indicated by the text color. The background colors show the group membership assigned by Kraemer et al. 2007 [16]. Sequences found upstream of domain cassette 8 (Figure 3) are marked with black squares. (N) The branch labels show the fraction of Markov clusters with this group present. (O) The branch labels show the bootstrap values as fractions of 1000 bootstraps. Monophyletic subgroups with a bootstrap support above 0.7 and containing sequences from at least four different strains of P. falciparum are highlighted with thick red branches. Some subgroups were further expanded (without bootstrap support) to form larger monophyletic groups: UPSA2 and UPSB3 are expanded to include additional sequences annotated to UPSA2 and UPSB3 respectively by Kraemer et al. 2007 [16], UPSB2 is expanded to include two genes with same domain architecture, and UPSC1 is expanded to include three sequences that fall between UPSC1 and UPSC2 but within the larger monophyletic group comprising all UPSC sequences. The sequences are shown with thick black branches. The additional sequences included by this expansion are denoted with an asterisk in the annotation in Figure S4 and S5. UPSA3 and UPSB1 are groups that contain all the sequences not assigned to any other subgroup in UPSA and UPSB respectively. ND: Not Determined. (1.11 MB ZIP) [file pcbi.1000933.s003.zip › Figure S2F - Tree DBLg.pdf]

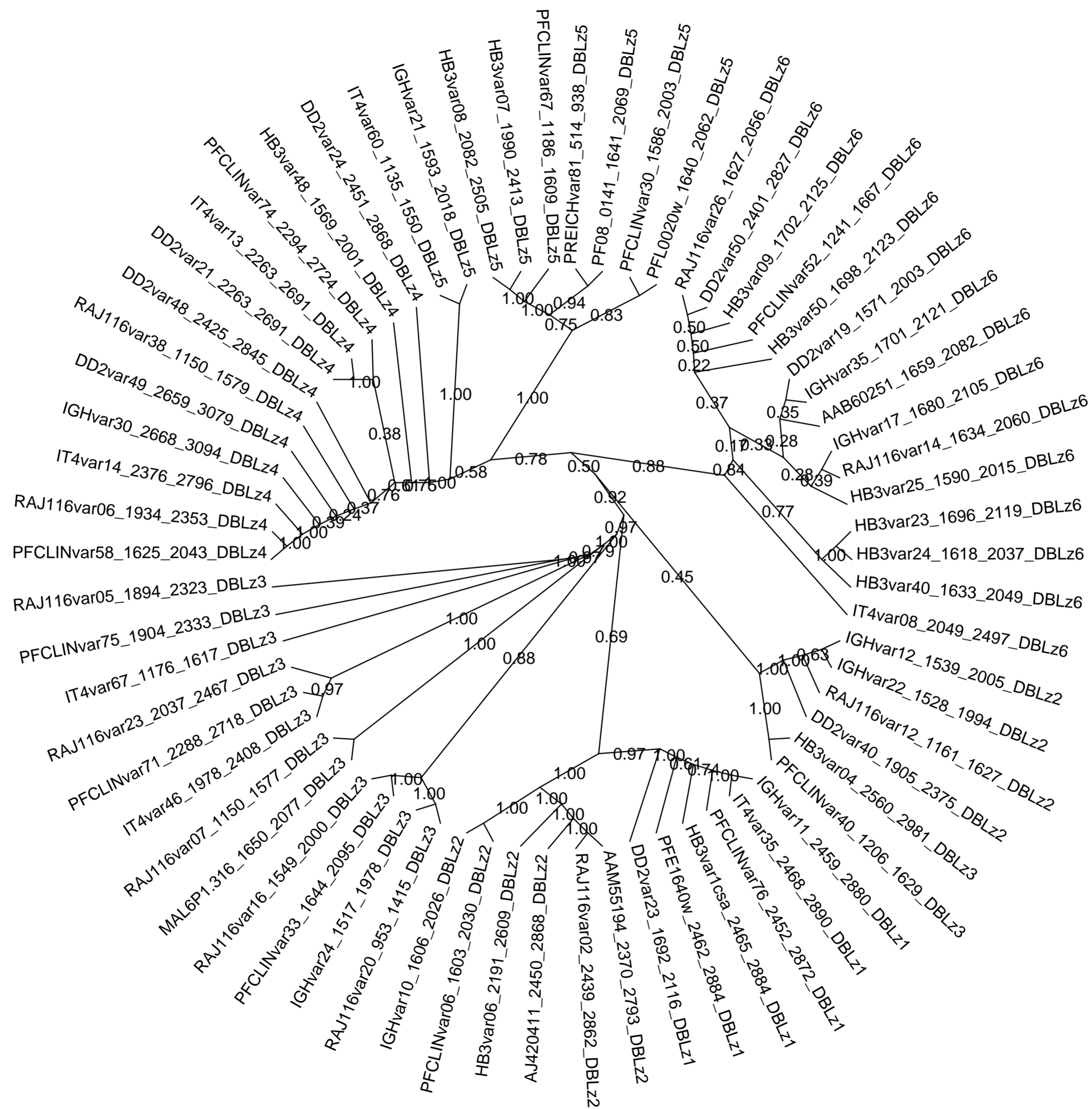

Supplement: Figure S2 — Trees showing subclassification of all major PfEMP1 domain classes. ML trees based on amino acid alignments of each of the following domain classes are shown in panels A–M: DBLα0, α1, β, δ, ε, γ, ζ; CIDRα, β, γ, δ; NTS; ATS. Sequence names as well as start and stop position of the domains are given in the trees, followed by classification of the domain. Panel N and O: Assignment of sequences to UPS groups by Markov clustering (N) and neighbor joining (O). The UPS groups were named as indicated by the text color. The background colors show the group membership assigned by Kraemer et al. 2007 [16]. Sequences found upstream of domain cassette 8 (Figure 3) are marked with black squares. (N) The branch labels show the fraction of Markov clusters with this group present. (O) The branch labels show the bootstrap values as fractions of 1000 bootstraps. Monophyletic subgroups with a bootstrap support above 0.7 and containing sequences from at least four different strains of P. falciparum are highlighted with thick red branches. Some subgroups were further expanded (without bootstrap support) to form larger monophyletic groups: UPSA2 and UPSB3 are expanded to include additional sequences annotated to UPSA2 and UPSB3 respectively by Kraemer et al. 2007 [16], UPSB2 is expanded to include two genes with same domain architecture, and UPSC1 is expanded to include three sequences that fall between UPSC1 and UPSC2 but within the larger monophyletic group comprising all UPSC sequences. The sequences are shown with thick black branches. The additional sequences included by this expansion are denoted with an asterisk in the annotation in Figure S4 and S5. UPSA3 and UPSB1 are groups that contain all the sequences not assigned to any other subgroup in UPSA and UPSB respectively. ND: Not Determined. (1.11 MB ZIP) [file pcbi.1000933.s003.zip › Figure S2G - Tree DBLz.pdf]

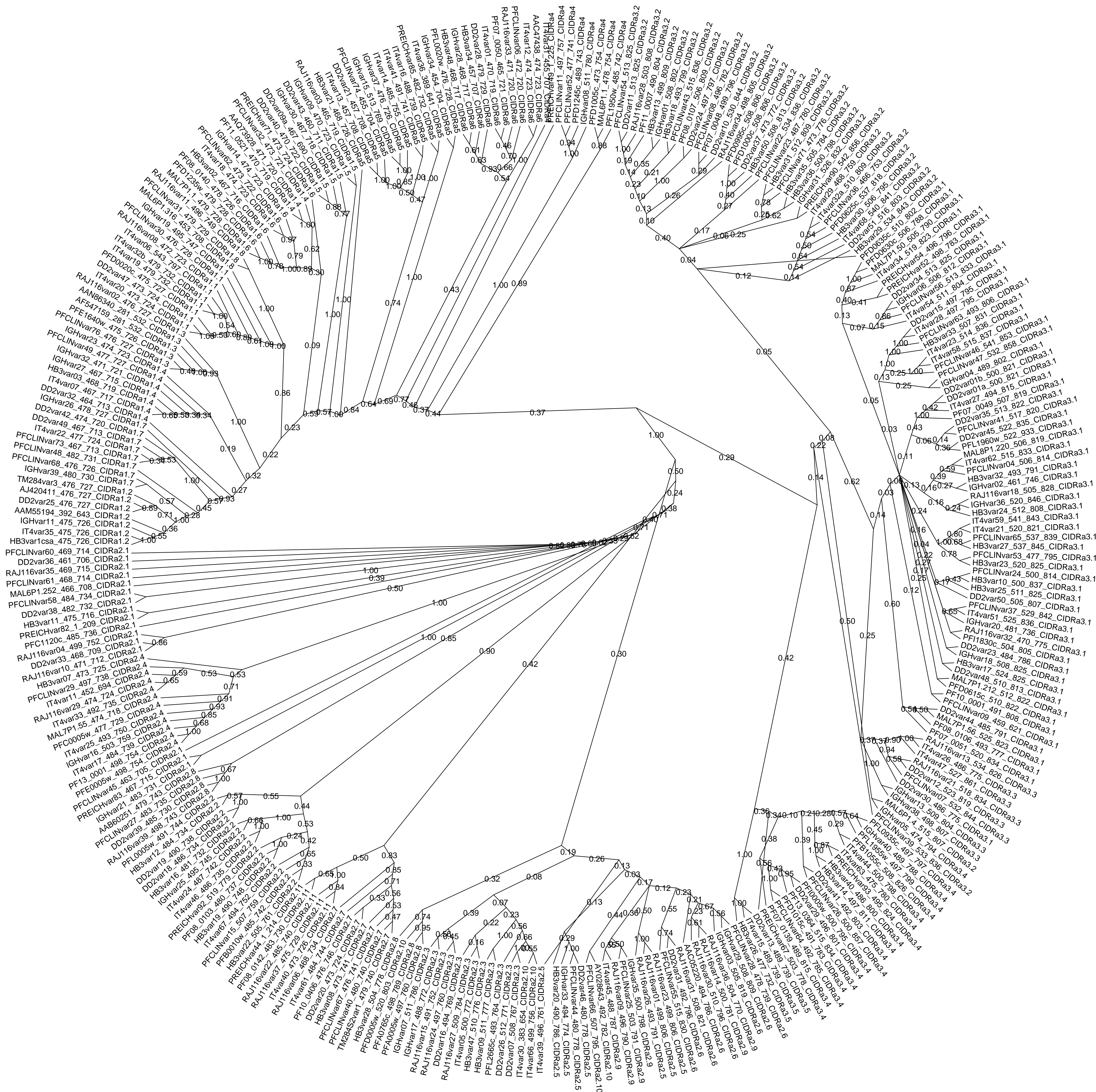

Supplement: Figure S2 — Trees showing subclassification of all major PfEMP1 domain classes. ML trees based on amino acid alignments of each of the following domain classes are shown in panels A–M: DBLα0, α1, β, δ, ε, γ, ζ; CIDRα, β, γ, δ; NTS; ATS. Sequence names as well as start and stop position of the domains are given in the trees, followed by classification of the domain. Panel N and O: Assignment of sequences to UPS groups by Markov clustering (N) and neighbor joining (O). The UPS groups were named as indicated by the text color. The background colors show the group membership assigned by Kraemer et al. 2007 [16]. Sequences found upstream of domain cassette 8 (Figure 3) are marked with black squares. (N) The branch labels show the fraction of Markov clusters with this group present. (O) The branch labels show the bootstrap values as fractions of 1000 bootstraps. Monophyletic subgroups with a bootstrap support above 0.7 and containing sequences from at least four different strains of P. falciparum are highlighted with thick red branches. Some subgroups were further expanded (without bootstrap support) to form larger monophyletic groups: UPSA2 and UPSB3 are expanded to include additional sequences annotated to UPSA2 and UPSB3 respectively by Kraemer et al. 2007 [16], UPSB2 is expanded to include two genes with same domain architecture, and UPSC1 is expanded to include three sequences that fall between UPSC1 and UPSC2 but within the larger monophyletic group comprising all UPSC sequences. The sequences are shown with thick black branches. The additional sequences included by this expansion are denoted with an asterisk in the annotation in Figure S4 and S5. UPSA3 and UPSB1 are groups that contain all the sequences not assigned to any other subgroup in UPSA and UPSB respectively. ND: Not Determined. (1.11 MB ZIP) [file pcbi.1000933.s003.zip › Figure S2H - Tree CIDRa.pdf]

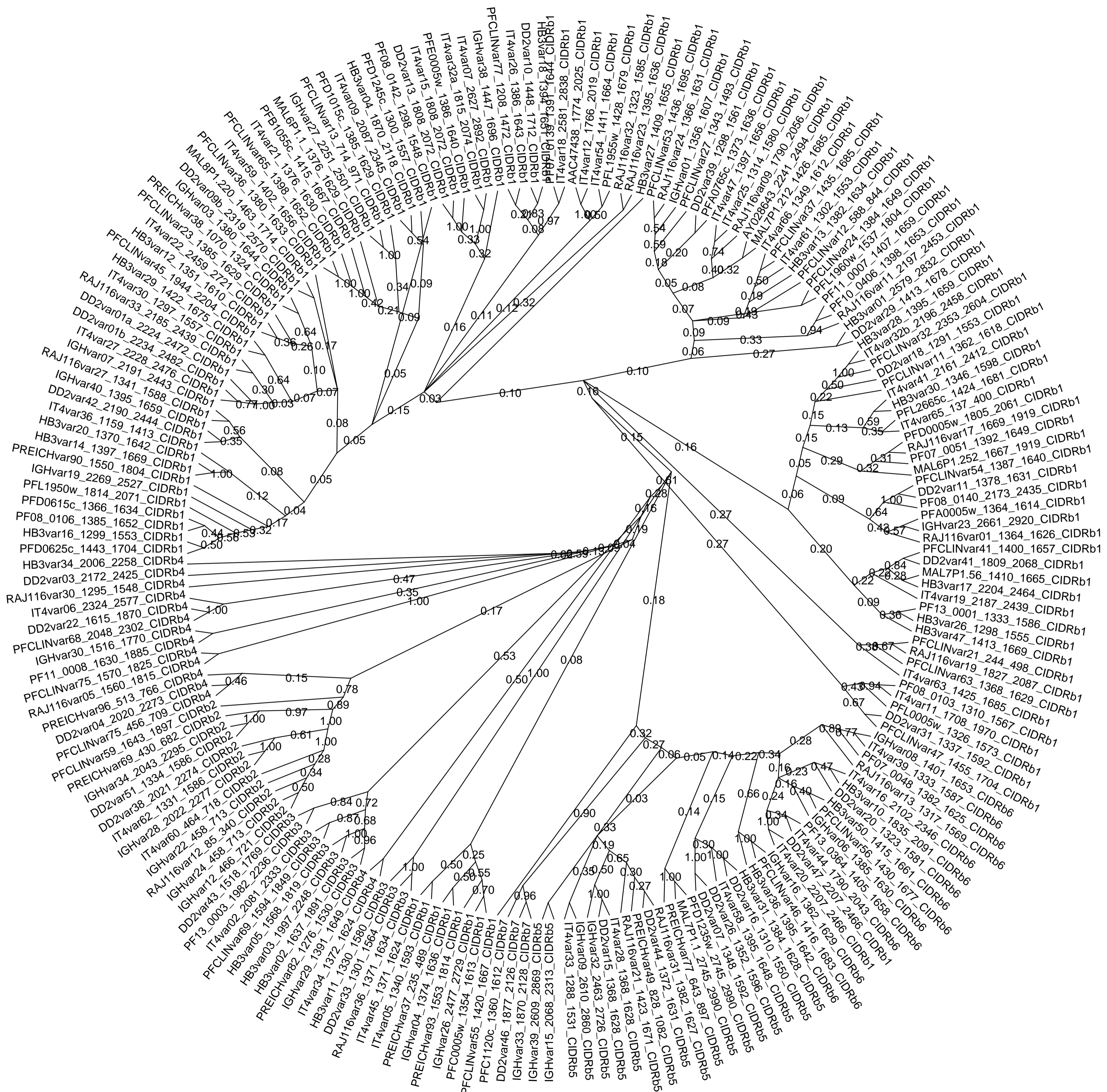

Supplement: Figure S2 — Trees showing subclassification of all major PfEMP1 domain classes. ML trees based on amino acid alignments of each of the following domain classes are shown in panels A–M: DBLα0, α1, β, δ, ε, γ, ζ; CIDRα, β, γ, δ; NTS; ATS. Sequence names as well as start and stop position of the domains are given in the trees, followed by classification of the domain. Panel N and O: Assignment of sequences to UPS groups by Markov clustering (N) and neighbor joining (O). The UPS groups were named as indicated by the text color. The background colors show the group membership assigned by Kraemer et al. 2007 [16]. Sequences found upstream of domain cassette 8 (Figure 3) are marked with black squares. (N) The branch labels show the fraction of Markov clusters with this group present. (O) The branch labels show the bootstrap values as fractions of 1000 bootstraps. Monophyletic subgroups with a bootstrap support above 0.7 and containing sequences from at least four different strains of P. falciparum are highlighted with thick red branches. Some subgroups were further expanded (without bootstrap support) to form larger monophyletic groups: UPSA2 and UPSB3 are expanded to include additional sequences annotated to UPSA2 and UPSB3 respectively by Kraemer et al. 2007 [16], UPSB2 is expanded to include two genes with same domain architecture, and UPSC1 is expanded to include three sequences that fall between UPSC1 and UPSC2 but within the larger monophyletic group comprising all UPSC sequences. The sequences are shown with thick black branches. The additional sequences included by this expansion are denoted with an asterisk in the annotation in Figure S4 and S5. UPSA3 and UPSB1 are groups that contain all the sequences not assigned to any other subgroup in UPSA and UPSB respectively. ND: Not Determined. (1.11 MB ZIP) [file pcbi.1000933.s003.zip › Figure S2I - Tree CIDRb.pdf]

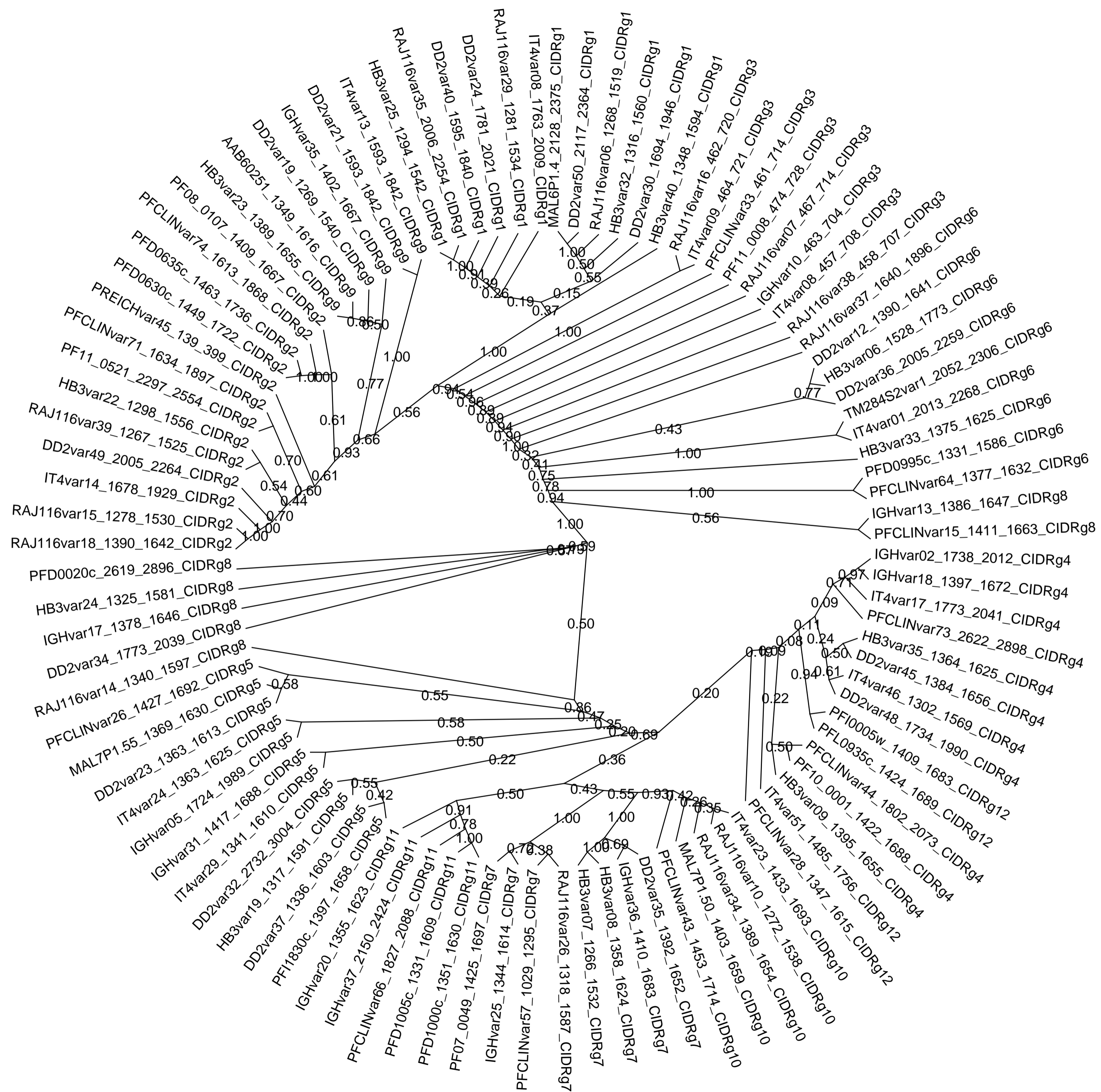

Supplement: Figure S2 — Trees showing subclassification of all major PfEMP1 domain classes. ML trees based on amino acid alignments of each of the following domain classes are shown in panels A–M: DBLα0, α1, β, δ, ε, γ, ζ; CIDRα, β, γ, δ; NTS; ATS. Sequence names as well as start and stop position of the domains are given in the trees, followed by classification of the domain. Panel N and O: Assignment of sequences to UPS groups by Markov clustering (N) and neighbor joining (O). The UPS groups were named as indicated by the text color. The background colors show the group membership assigned by Kraemer et al. 2007 [16]. Sequences found upstream of domain cassette 8 (Figure 3) are marked with black squares. (N) The branch labels show the fraction of Markov clusters with this group present. (O) The branch labels show the bootstrap values as fractions of 1000 bootstraps. Monophyletic subgroups with a bootstrap support above 0.7 and containing sequences from at least four different strains of P. falciparum are highlighted with thick red branches. Some subgroups were further expanded (without bootstrap support) to form larger monophyletic groups: UPSA2 and UPSB3 are expanded to include additional sequences annotated to UPSA2 and UPSB3 respectively by Kraemer et al. 2007 [16], UPSB2 is expanded to include two genes with same domain architecture, and UPSC1 is expanded to include three sequences that fall between UPSC1 and UPSC2 but within the larger monophyletic group comprising all UPSC sequences. The sequences are shown with thick black branches. The additional sequences included by this expansion are denoted with an asterisk in the annotation in Figure S4 and S5. UPSA3 and UPSB1 are groups that contain all the sequences not assigned to any other subgroup in UPSA and UPSB respectively. ND: Not Determined. (1.11 MB ZIP) [file pcbi.1000933.s003.zip › Figure S2J - Tree CIDRg.pdf]

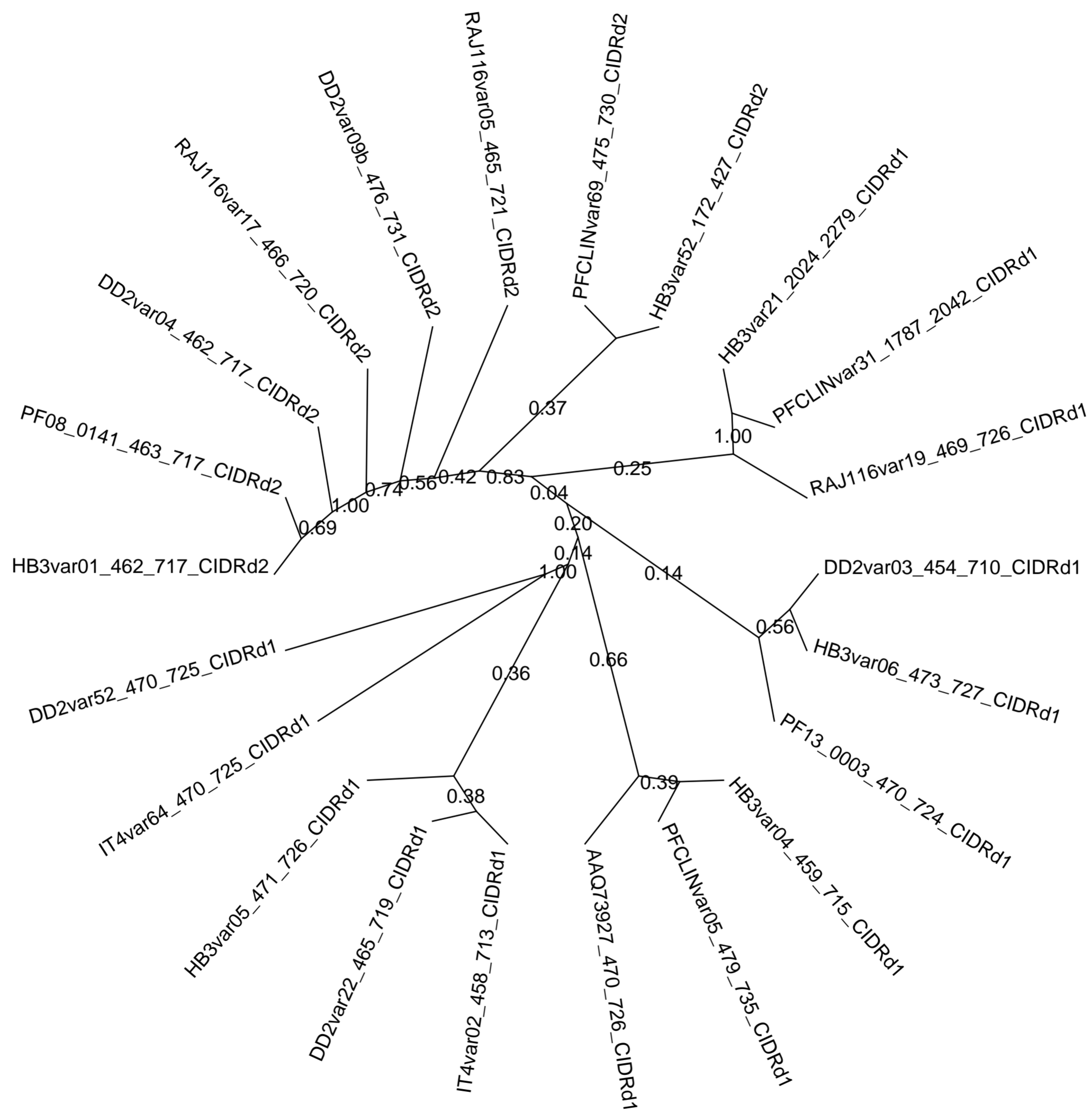

Supplement: Figure S2 — Trees showing subclassification of all major PfEMP1 domain classes. ML trees based on amino acid alignments of each of the following domain classes are shown in panels A–M: DBLα0, α1, β, δ, ε, γ, ζ; CIDRα, β, γ, δ; NTS; ATS. Sequence names as well as start and stop position of the domains are given in the trees, followed by classification of the domain. Panel N and O: Assignment of sequences to UPS groups by Markov clustering (N) and neighbor joining (O). The UPS groups were named as indicated by the text color. The background colors show the group membership assigned by Kraemer et al. 2007 [16]. Sequences found upstream of domain cassette 8 (Figure 3) are marked with black squares. (N) The branch labels show the fraction of Markov clusters with this group present. (O) The branch labels show the bootstrap values as fractions of 1000 bootstraps. Monophyletic subgroups with a bootstrap support above 0.7 and containing sequences from at least four different strains of P. falciparum are highlighted with thick red branches. Some subgroups were further expanded (without bootstrap support) to form larger monophyletic groups: UPSA2 and UPSB3 are expanded to include additional sequences annotated to UPSA2 and UPSB3 respectively by Kraemer et al. 2007 [16], UPSB2 is expanded to include two genes with same domain architecture, and UPSC1 is expanded to include three sequences that fall between UPSC1 and UPSC2 but within the larger monophyletic group comprising all UPSC sequences. The sequences are shown with thick black branches. The additional sequences included by this expansion are denoted with an asterisk in the annotation in Figure S4 and S5. UPSA3 and UPSB1 are groups that contain all the sequences not assigned to any other subgroup in UPSA and UPSB respectively. ND: Not Determined. (1.11 MB ZIP) [file pcbi.1000933.s003.zip › Figure S2K - Tree CIDRd.pdf]

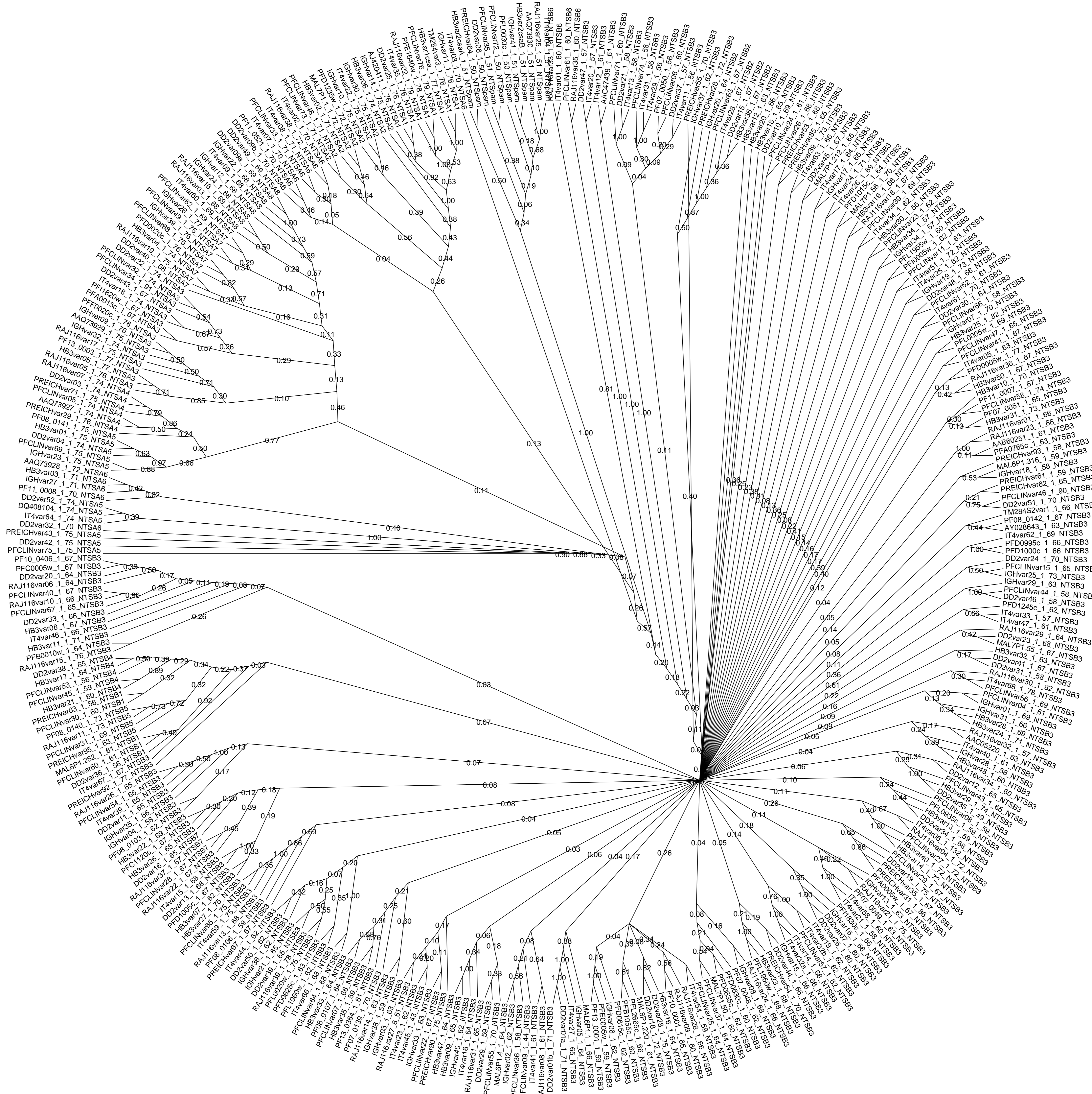

Supplement: Figure S2 — Trees showing subclassification of all major PfEMP1 domain classes. ML trees based on amino acid alignments of each of the following domain classes are shown in panels A–M: DBLα0, α1, β, δ, ε, γ, ζ; CIDRα, β, γ, δ; NTS; ATS. Sequence names as well as start and stop position of the domains are given in the trees, followed by classification of the domain. Panel N and O: Assignment of sequences to UPS groups by Markov clustering (N) and neighbor joining (O). The UPS groups were named as indicated by the text color. The background colors show the group membership assigned by Kraemer et al. 2007 [16]. Sequences found upstream of domain cassette 8 (Figure 3) are marked with black squares. (N) The branch labels show the fraction of Markov clusters with this group present. (O) The branch labels show the bootstrap values as fractions of 1000 bootstraps. Monophyletic subgroups with a bootstrap support above 0.7 and containing sequences from at least four different strains of P. falciparum are highlighted with thick red branches. Some subgroups were further expanded (without bootstrap support) to form larger monophyletic groups: UPSA2 and UPSB3 are expanded to include additional sequences annotated to UPSA2 and UPSB3 respectively by Kraemer et al. 2007 [16], UPSB2 is expanded to include two genes with same domain architecture, and UPSC1 is expanded to include three sequences that fall between UPSC1 and UPSC2 but within the larger monophyletic group comprising all UPSC sequences. The sequences are shown with thick black branches. The additional sequences included by this expansion are denoted with an asterisk in the annotation in Figure S4 and S5. UPSA3 and UPSB1 are groups that contain all the sequences not assigned to any other subgroup in UPSA and UPSB respectively. ND: Not Determined. (1.11 MB ZIP) [file pcbi.1000933.s003.zip › Figure S2L - Tree NTS.pdf]

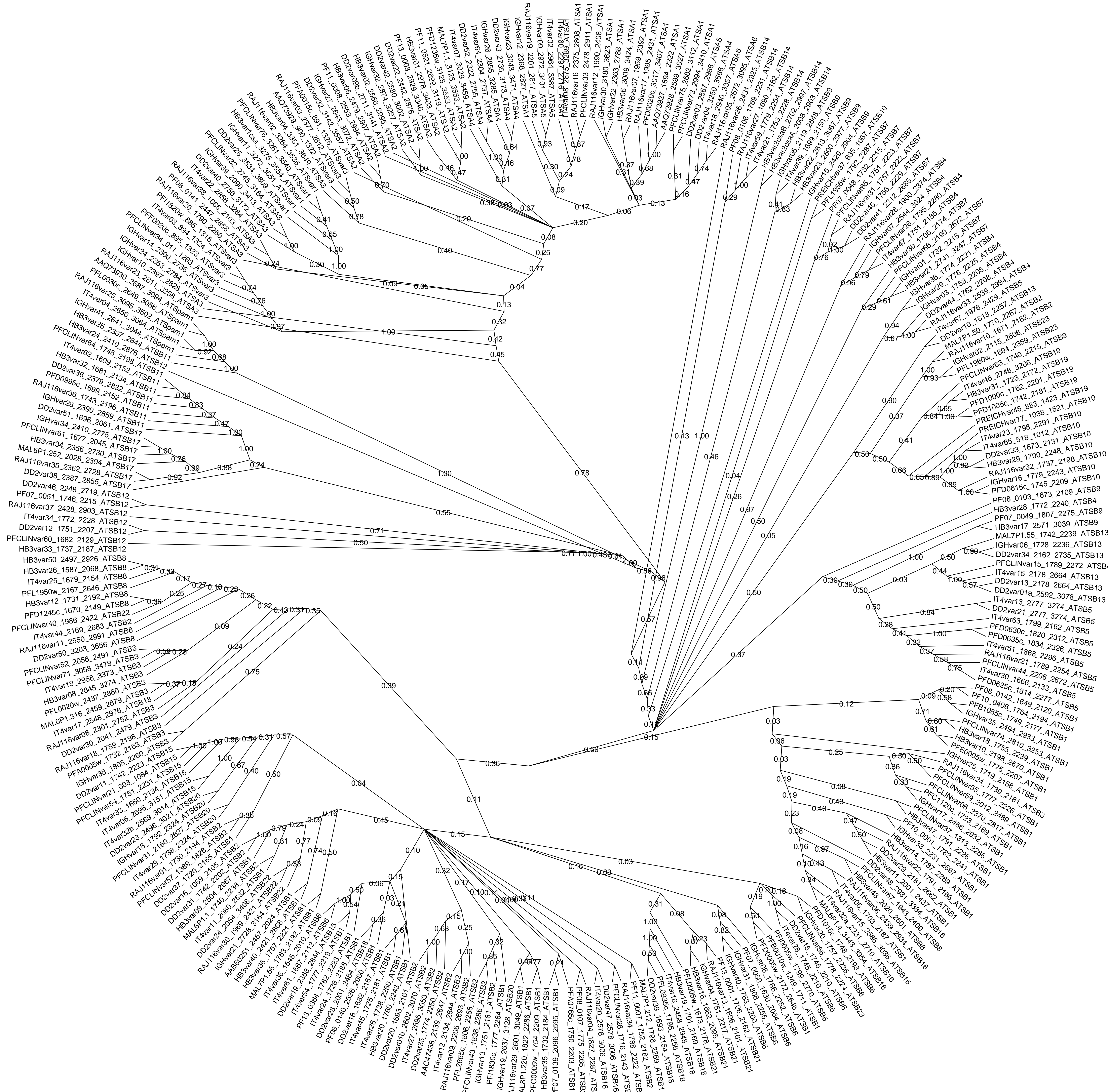

Supplement: Figure S2 — Trees showing subclassification of all major PfEMP1 domain classes. ML trees based on amino acid alignments of each of the following domain classes are shown in panels A–M: DBLα0, α1, β, δ, ε, γ, ζ; CIDRα, β, γ, δ; NTS; ATS. Sequence names as well as start and stop position of the domains are given in the trees, followed by classification of the domain. Panel N and O: Assignment of sequences to UPS groups by Markov clustering (N) and neighbor joining (O). The UPS groups were named as indicated by the text color. The background colors show the group membership assigned by Kraemer et al. 2007 [16]. Sequences found upstream of domain cassette 8 (Figure 3) are marked with black squares. (N) The branch labels show the fraction of Markov clusters with this group present. (O) The branch labels show the bootstrap values as fractions of 1000 bootstraps. Monophyletic subgroups with a bootstrap support above 0.7 and containing sequences from at least four different strains of P. falciparum are highlighted with thick red branches. Some subgroups were further expanded (without bootstrap support) to form larger monophyletic groups: UPSA2 and UPSB3 are expanded to include additional sequences annotated to UPSA2 and UPSB3 respectively by Kraemer et al. 2007 [16], UPSB2 is expanded to include two genes with same domain architecture, and UPSC1 is expanded to include three sequences that fall between UPSC1 and UPSC2 but within the larger monophyletic group comprising all UPSC sequences. The sequences are shown with thick black branches. The additional sequences included by this expansion are denoted with an asterisk in the annotation in Figure S4 and S5. UPSA3 and UPSB1 are groups that contain all the sequences not assigned to any other subgroup in UPSA and UPSB respectively. ND: Not Determined. (1.11 MB ZIP) [file pcbi.1000933.s003.zip › Figure S2M - Tree ATS.pdf]

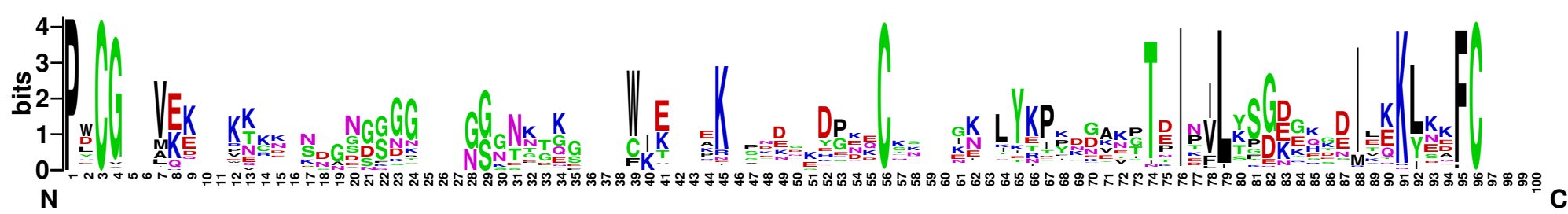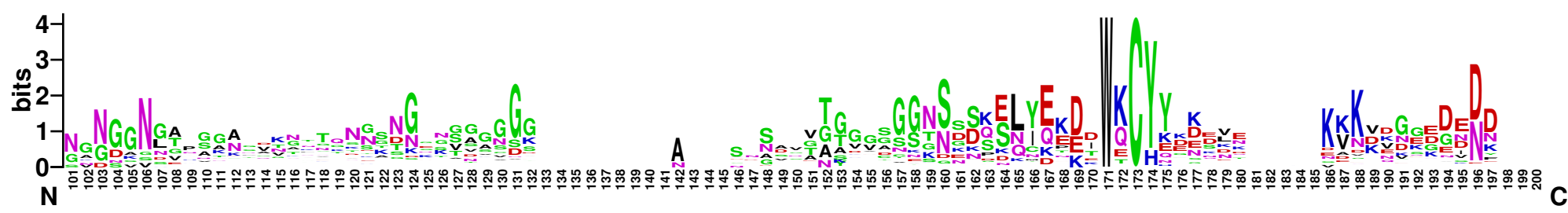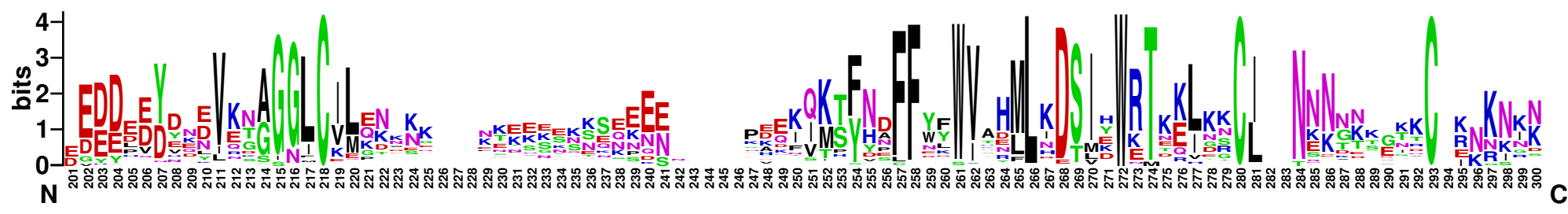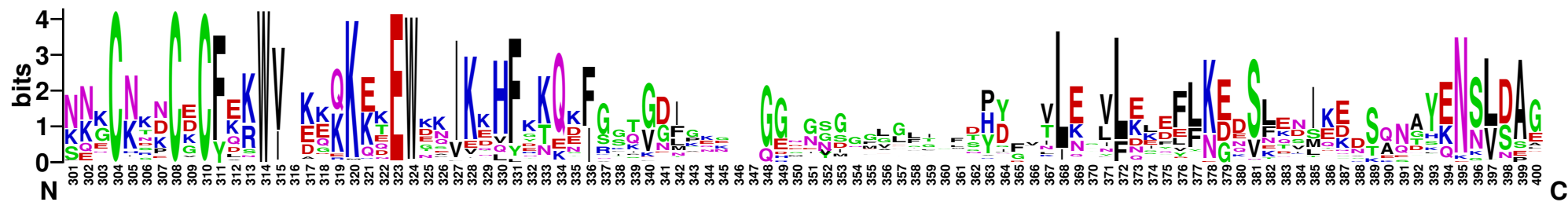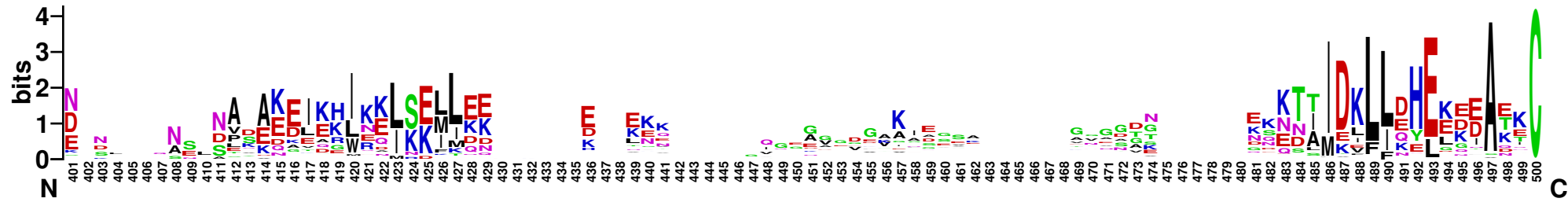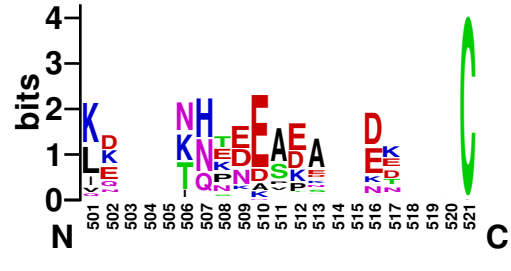

Supplement: Figure S3 — PfEMP1 domain class logos. Sequence conservation logos for major PfEMP1 domain classes (panel A–Z): CIDRα, α1, α2, α3, β, δ, γ, pam; DBLα0, α1 (without α1.3), α1.3, β, δ, ε (without ε1, ε2, ε11, ε13, εpam), ε1, ε2, ε11, ε13, εpam4, εpam5, γ, pam1, pam2, pam3, ζ; NTSA, NTSB, and M3AB. (2.42 MB ZIP) [file pcbi.1000933.s004.zip › Figure S3A - Logo CIDRa.pdf]

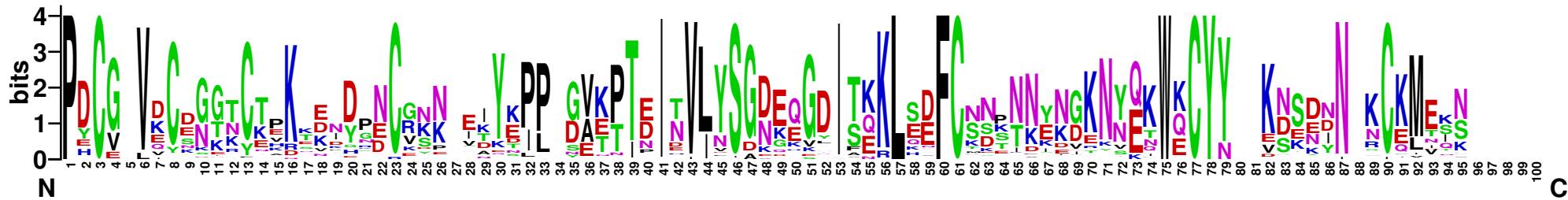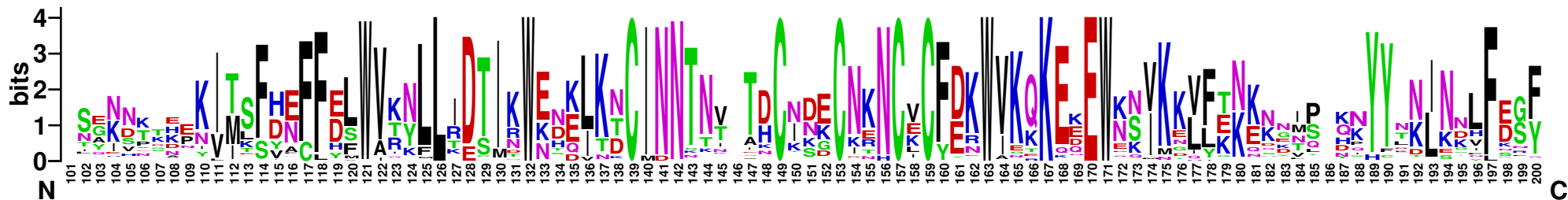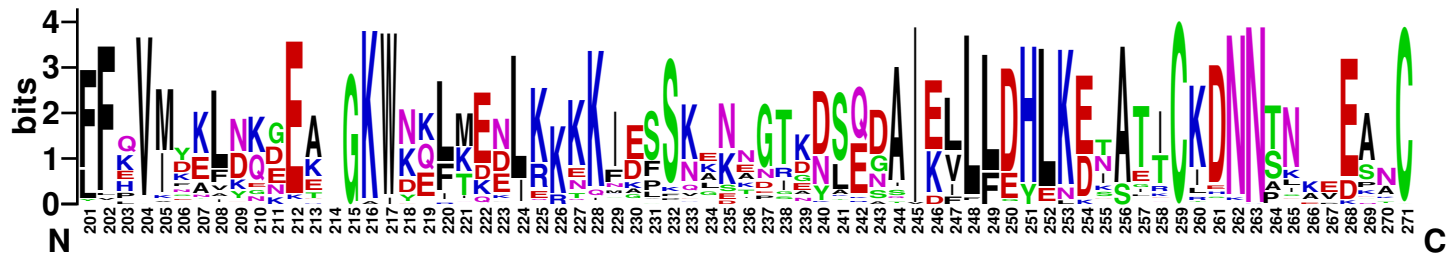

Supplement: Figure S3 — PfEMP1 domain class logos. Sequence conservation logos for major PfEMP1 domain classes (panel A–Z): CIDRα, α1, α2, α3, β, δ, γ, pam; DBLα0, α1 (without α1.3), α1.3, β, δ, ε (without ε1, ε2, ε11, ε13, εpam), ε1, ε2, ε11, ε13, εpam4, εpam5, γ, pam1, pam2, pam3, ζ; NTSA, NTSB, and M3AB. (2.42 MB ZIP) [file pcbi.1000933.s004.zip › Figure S3B - Logo CIDRa1.pdf]

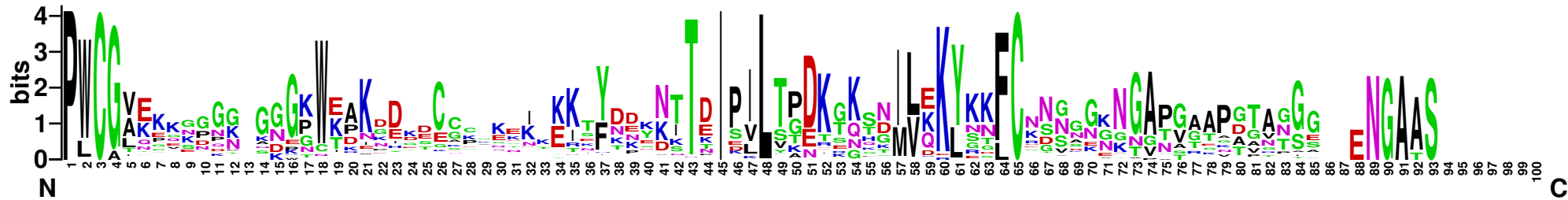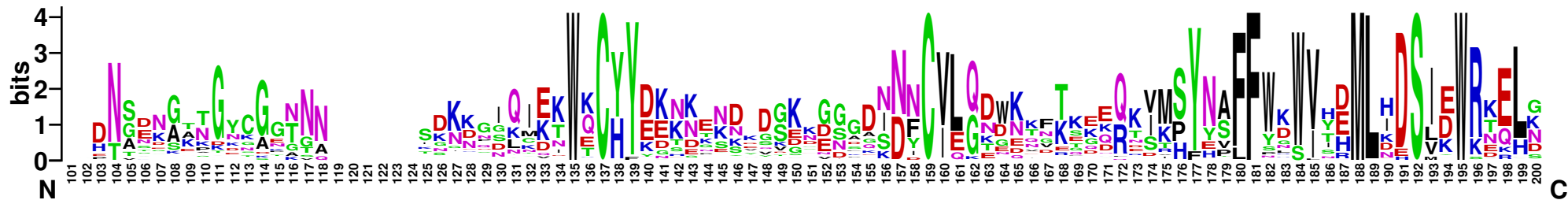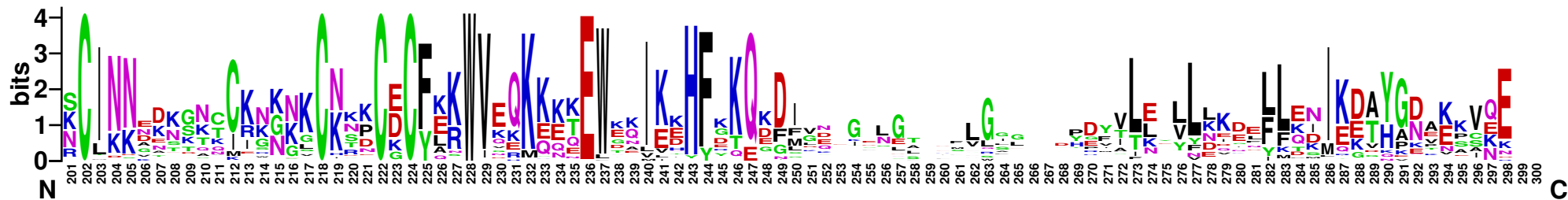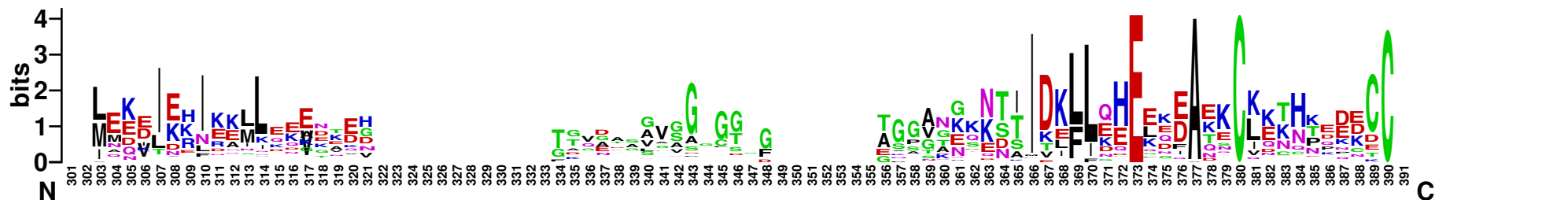

Supplement: Figure S3 — PfEMP1 domain class logos. Sequence conservation logos for major PfEMP1 domain classes (panel A–Z): CIDRα, α1, α2, α3, β, δ, γ, pam; DBLα0, α1 (without α1.3), α1.3, β, δ, ε (without ε1, ε2, ε11, ε13, εpam), ε1, ε2, ε11, ε13, εpam4, εpam5, γ, pam1, pam2, pam3, ζ; NTSA, NTSB, and M3AB. (2.42 MB ZIP) [file pcbi.1000933.s004.zip › Figure S3C - Logo CIDRa2.pdf]

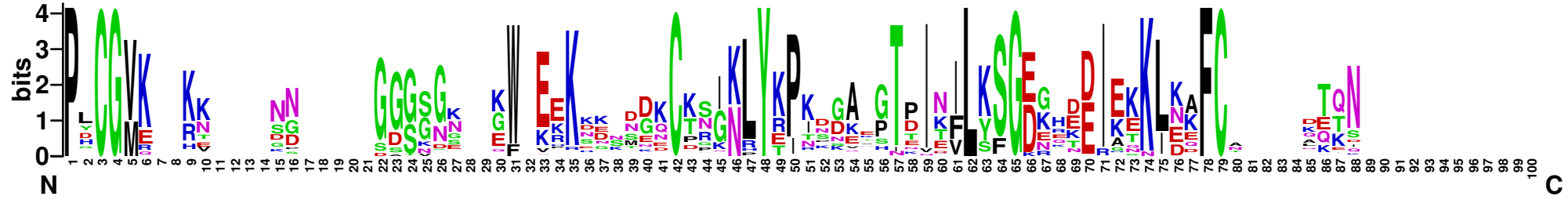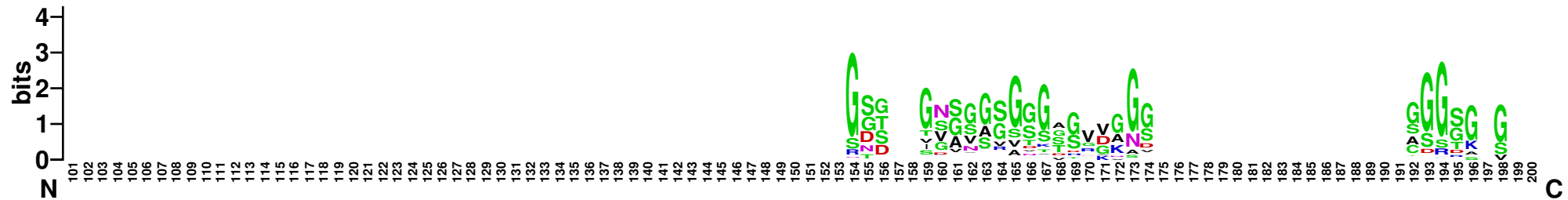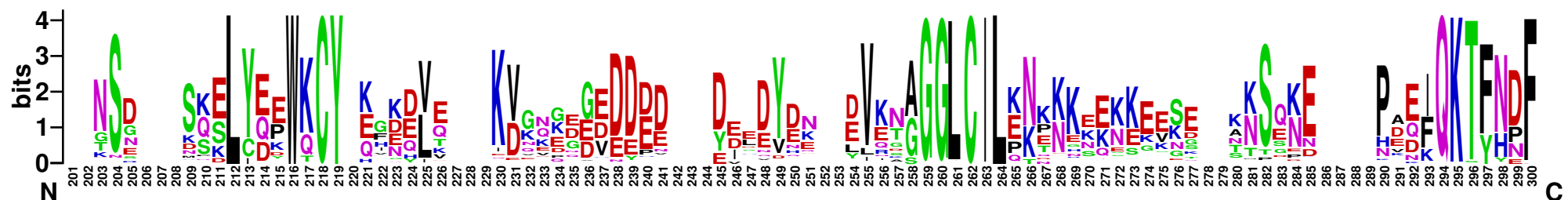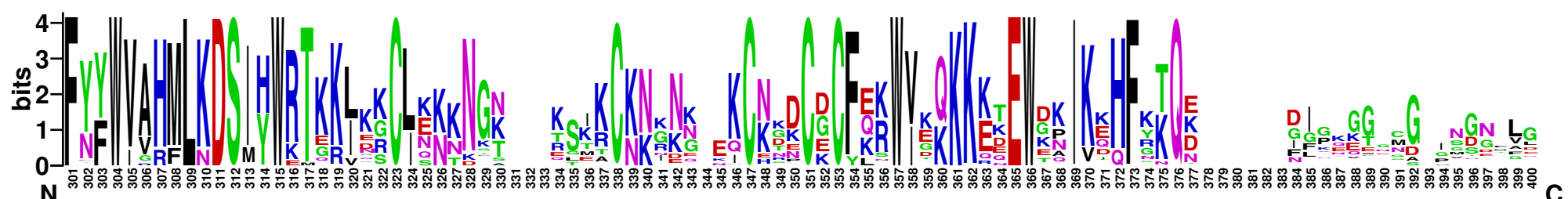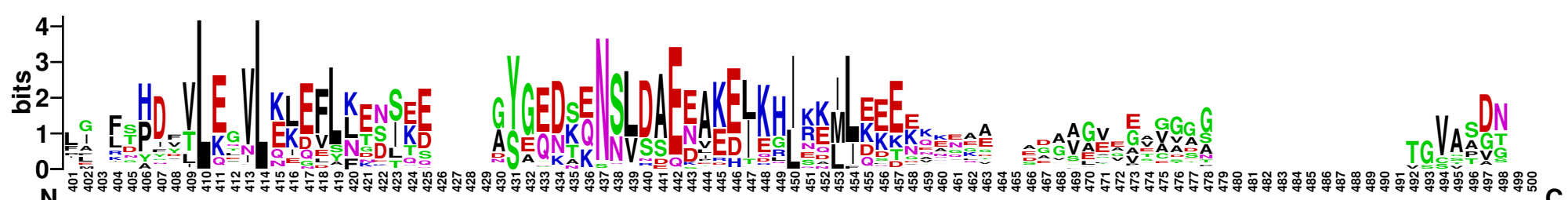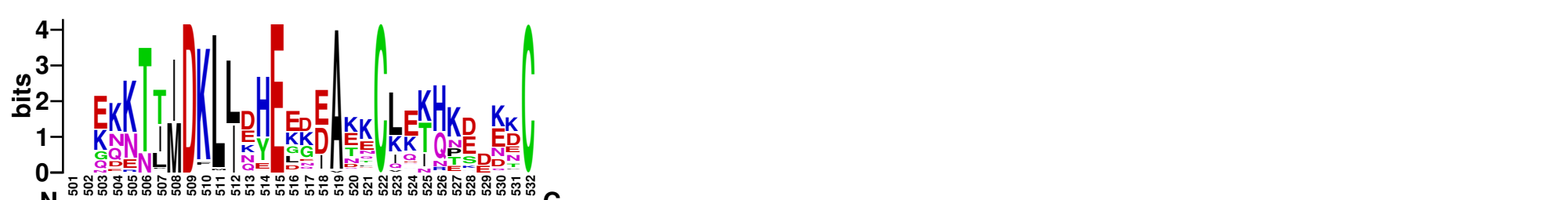

Supplement: Figure S3 — PfEMP1 domain class logos. Sequence conservation logos for major PfEMP1 domain classes (panel A–Z): CIDRα, α1, α2, α3, β, δ, γ, pam; DBLα0, α1 (without α1.3), α1.3, β, δ, ε (without ε1, ε2, ε11, ε13, εpam), ε1, ε2, ε11, ε13, εpam4, εpam5, γ, pam1, pam2, pam3, ζ; NTSA, NTSB, and M3AB. (2.42 MB ZIP) [file pcbi.1000933.s004.zip › Figure S3D - Logo CIDRa3.pdf]

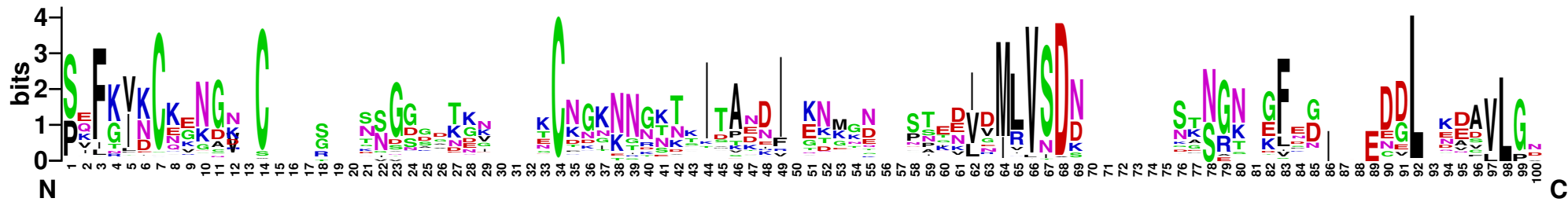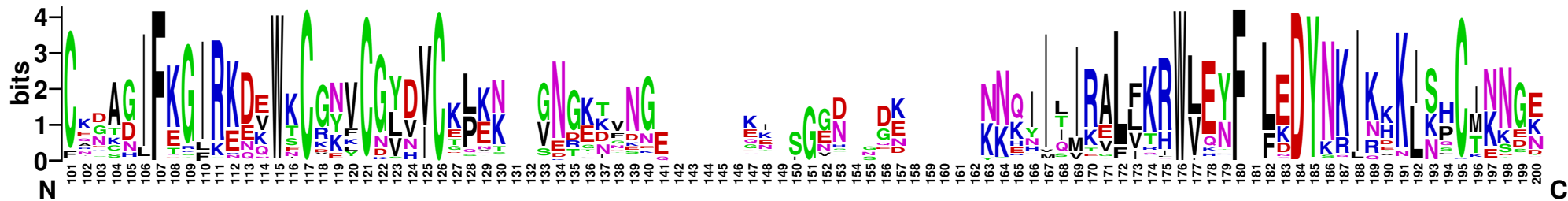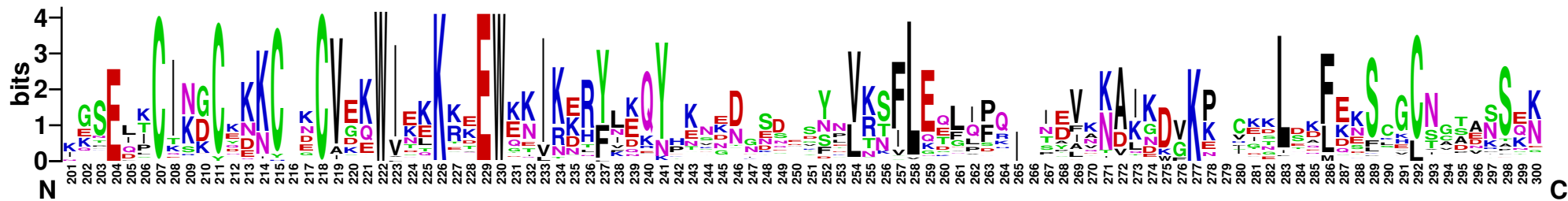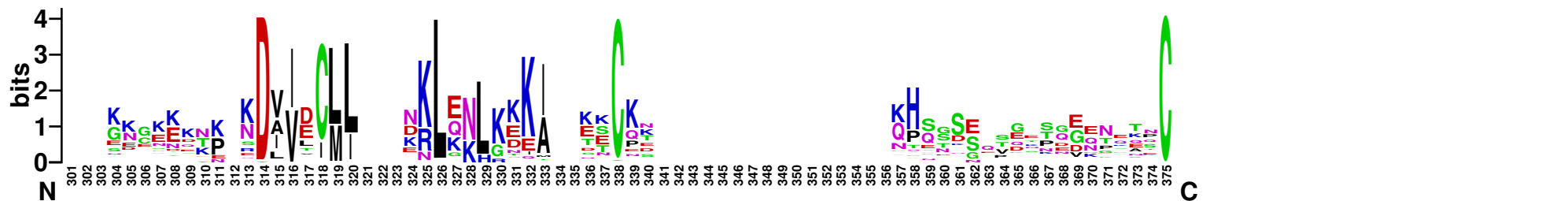

Supplement: Figure S3 — PfEMP1 domain class logos. Sequence conservation logos for major PfEMP1 domain classes (panel A–Z): CIDRα, α1, α2, α3, β, δ, γ, pam; DBLα0, α1 (without α1.3), α1.3, β, δ, ε (without ε1, ε2, ε11, ε13, εpam), ε1, ε2, ε11, ε13, εpam4, εpam5, γ, pam1, pam2, pam3, ζ; NTSA, NTSB, and M3AB. (2.42 MB ZIP) [file pcbi.1000933.s004.zip › Figure S3E - Logo CIDRb.pdf]

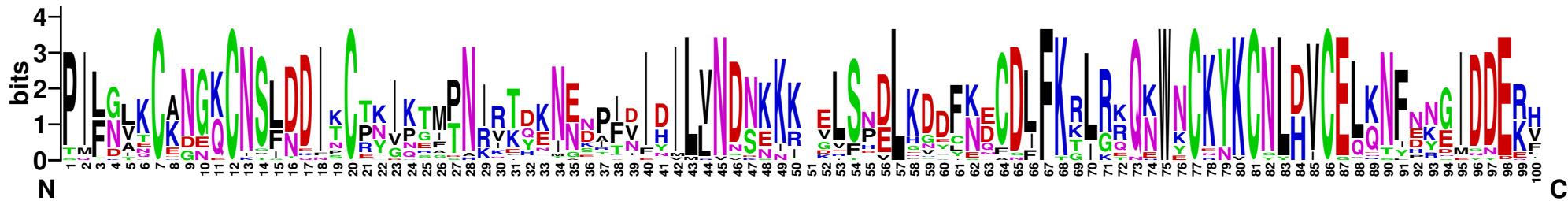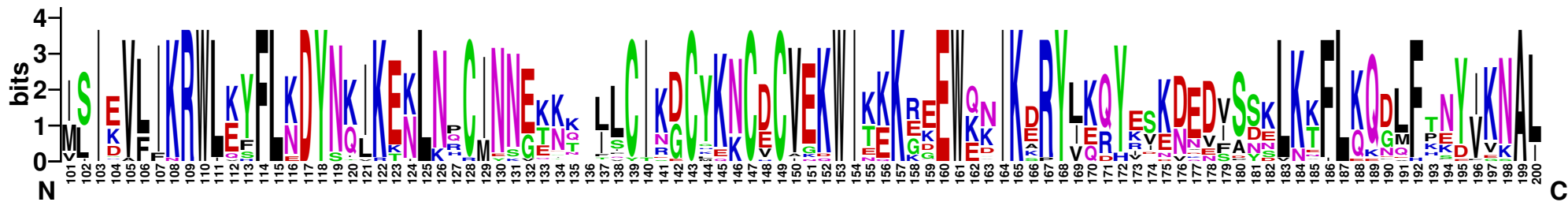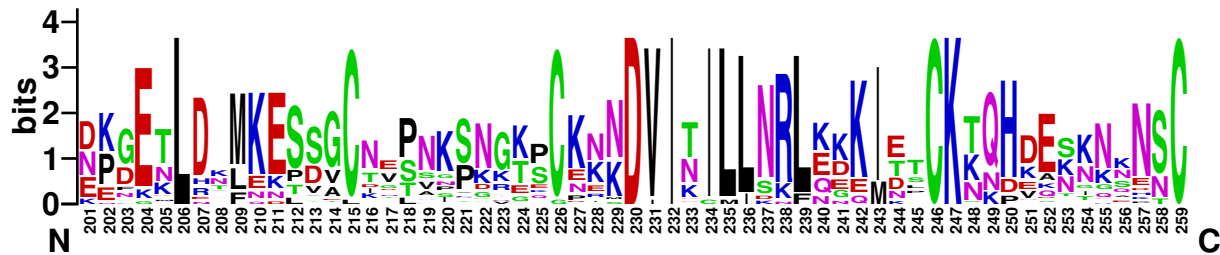

Supplement: Figure S3 — PfEMP1 domain class logos. Sequence conservation logos for major PfEMP1 domain classes (panel A–Z): CIDRα, α1, α2, α3, β, δ, γ, pam; DBLα0, α1 (without α1.3), α1.3, β, δ, ε (without ε1, ε2, ε11, ε13, εpam), ε1, ε2, ε11, ε13, εpam4, εpam5, γ, pam1, pam2, pam3, ζ; NTSA, NTSB, and M3AB. (2.42 MB ZIP) [file pcbi.1000933.s004.zip › Figure S3F - Logo CIDRd.pdf]

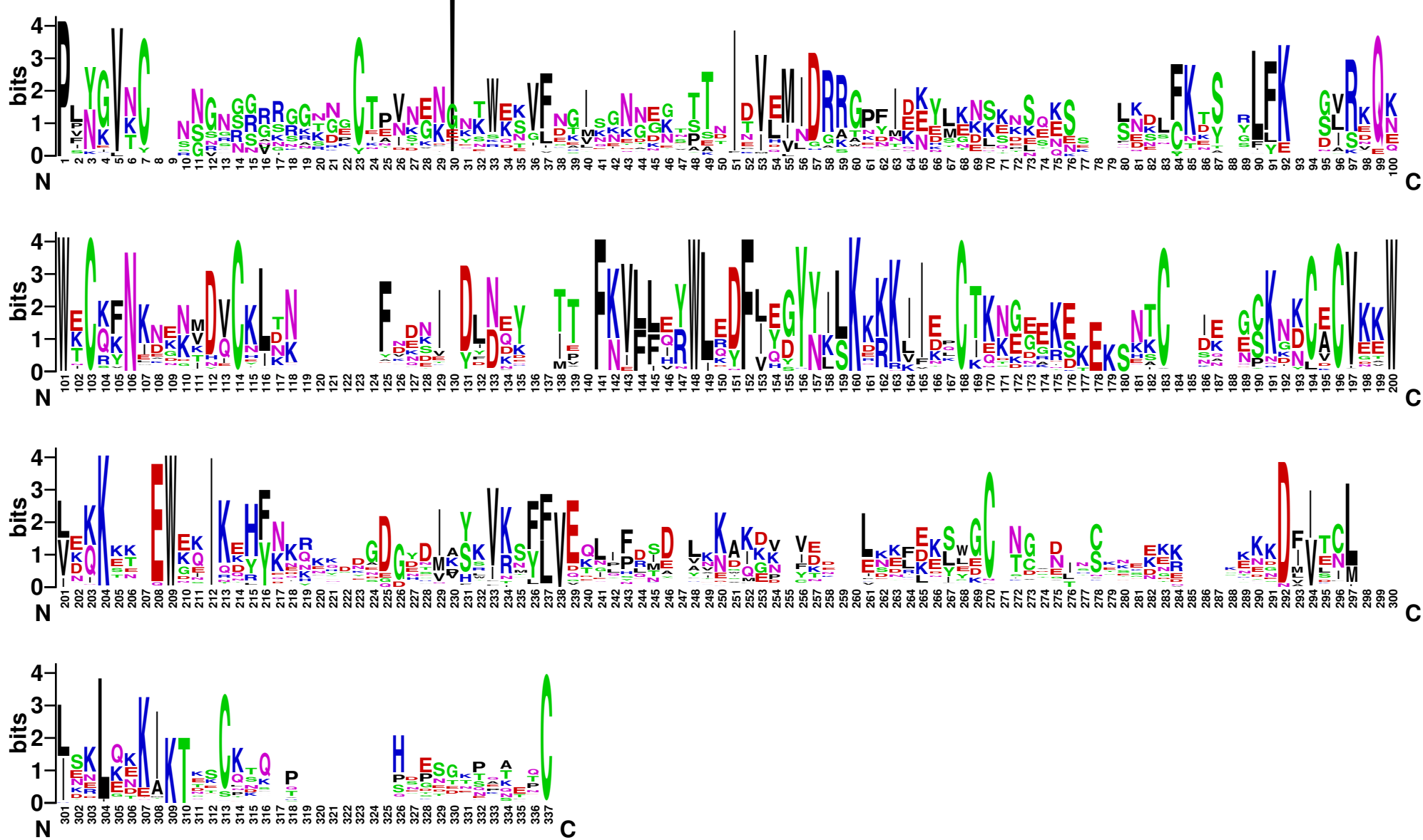

Supplement: Figure S3 — PfEMP1 domain class logos. Sequence conservation logos for major PfEMP1 domain classes (panel A–Z): CIDRα, α1, α2, α3, β, δ, γ, pam; DBLα0, α1 (without α1.3), α1.3, β, δ, ε (without ε1, ε2, ε11, ε13, εpam), ε1, ε2, ε11, ε13, εpam4, εpam5, γ, pam1, pam2, pam3, ζ; NTSA, NTSB, and M3AB. (2.42 MB ZIP) [file pcbi.1000933.s004.zip › Figure S3G - Logo CIDRg.pdf]

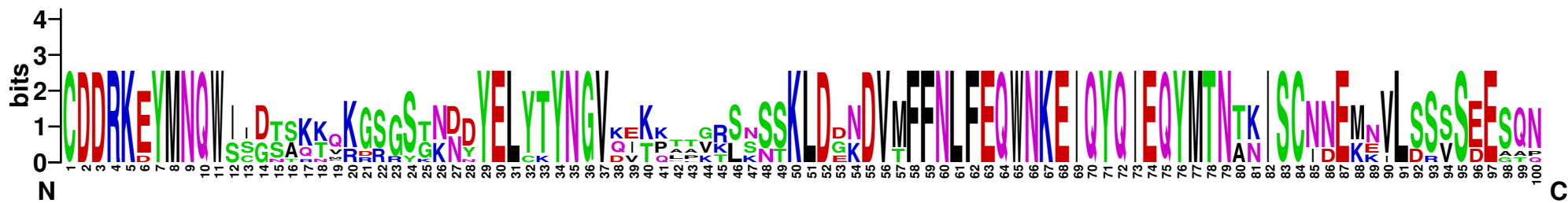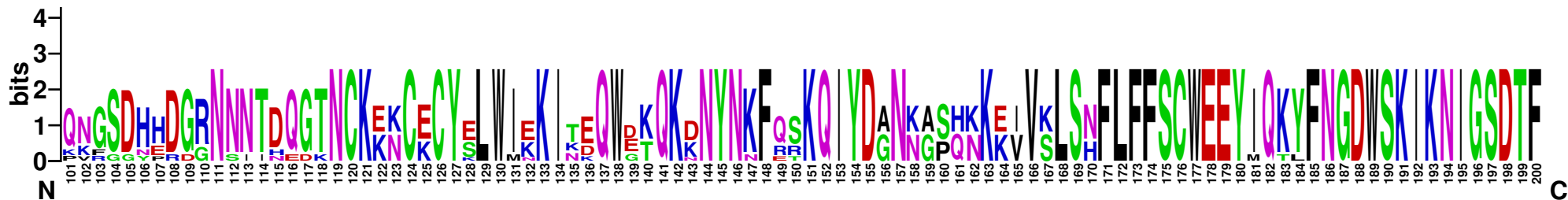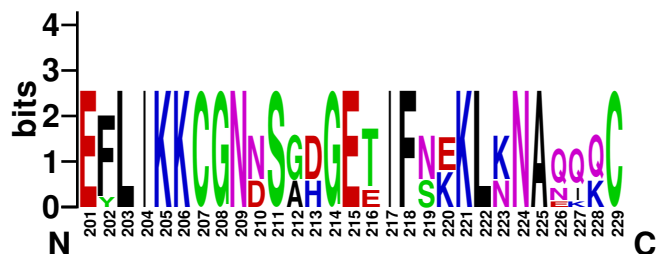

Supplement: Figure S3 — PfEMP1 domain class logos. Sequence conservation logos for major PfEMP1 domain classes (panel A–Z): CIDRα, α1, α2, α3, β, δ, γ, pam; DBLα0, α1 (without α1.3), α1.3, β, δ, ε (without ε1, ε2, ε11, ε13, εpam), ε1, ε2, ε11, ε13, εpam4, εpam5, γ, pam1, pam2, pam3, ζ; NTSA, NTSB, and M3AB. (2.42 MB ZIP) [file pcbi.1000933.s004.zip › Figure S3H - Logo CIDRpam.pdf]

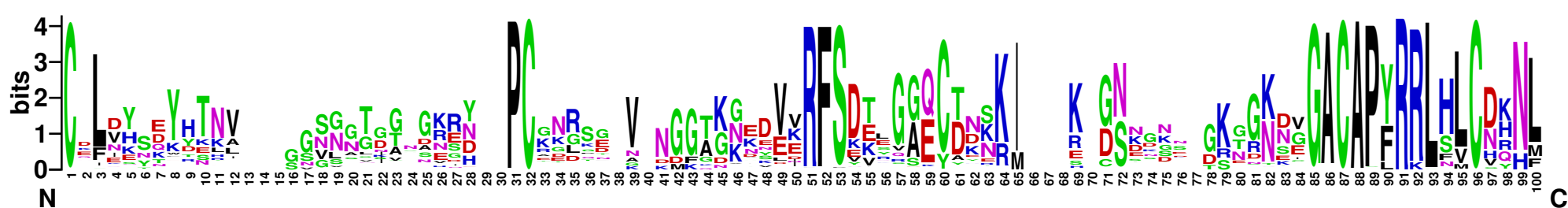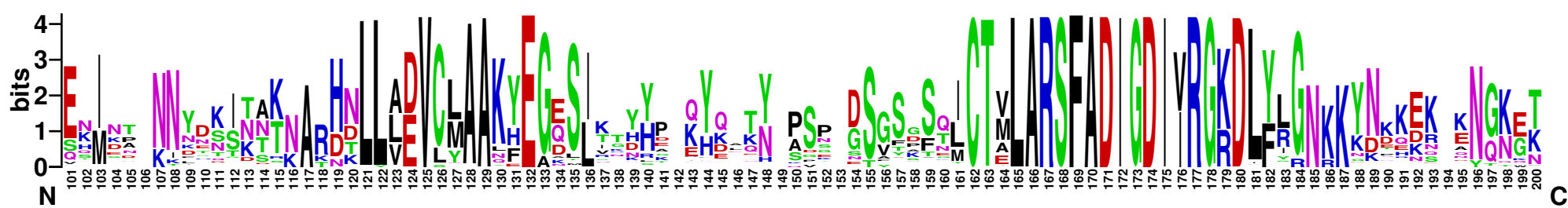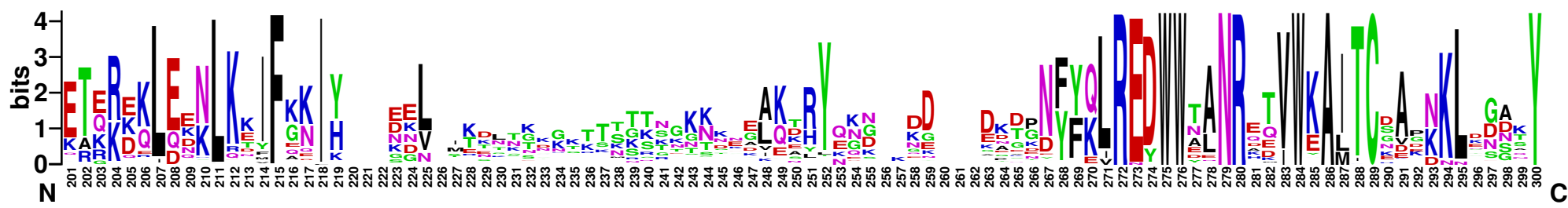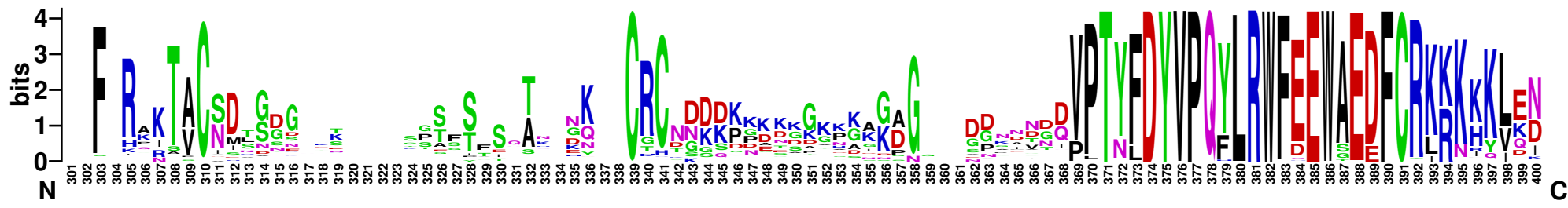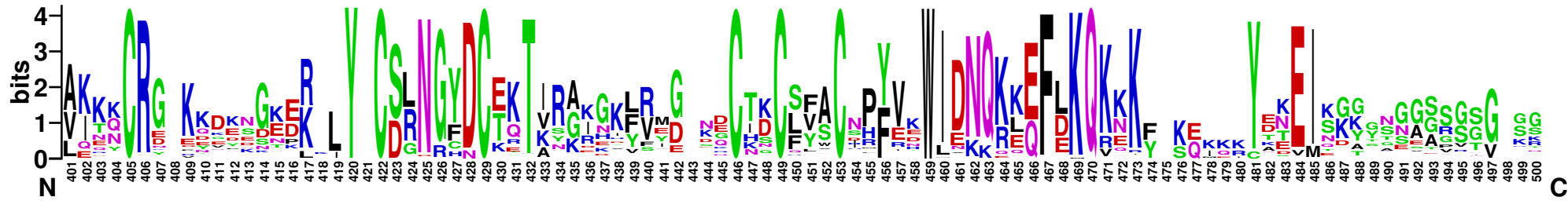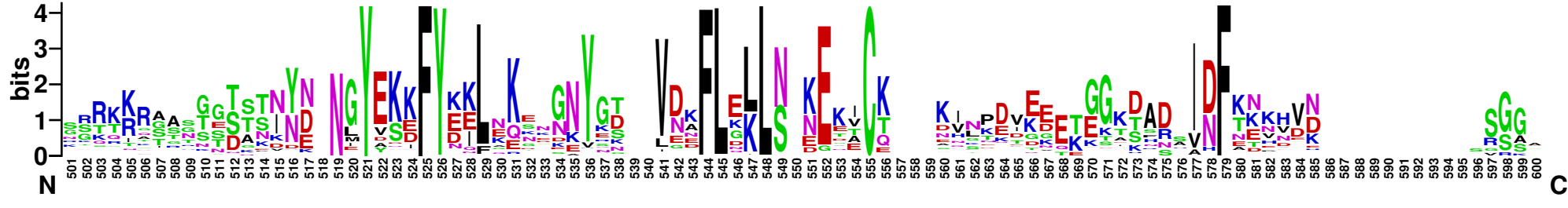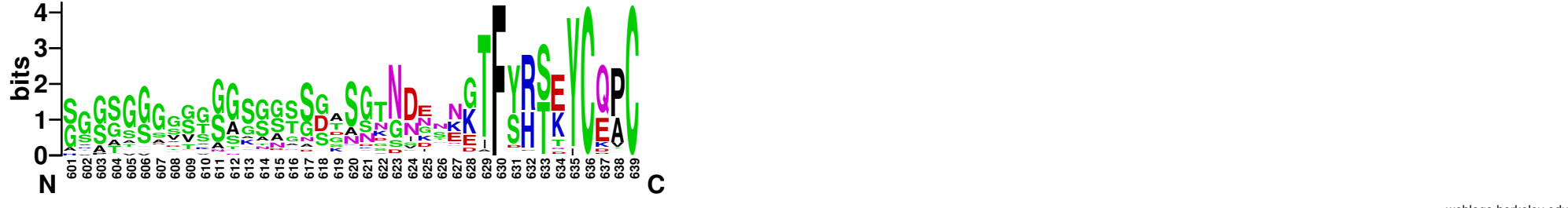

Supplement: Figure S3 — PfEMP1 domain class logos. Sequence conservation logos for major PfEMP1 domain classes (panel A–Z): CIDRα, α1, α2, α3, β, δ, γ, pam; DBLα0, α1 (without α1.3), α1.3, β, δ, ε (without ε1, ε2, ε11, ε13, εpam), ε1, ε2, ε11, ε13, εpam4, εpam5, γ, pam1, pam2, pam3, ζ; NTSA, NTSB, and M3AB. (2.42 MB ZIP) [file pcbi.1000933.s004.zip › Figure S3I - Logo DBLa0.pdf]

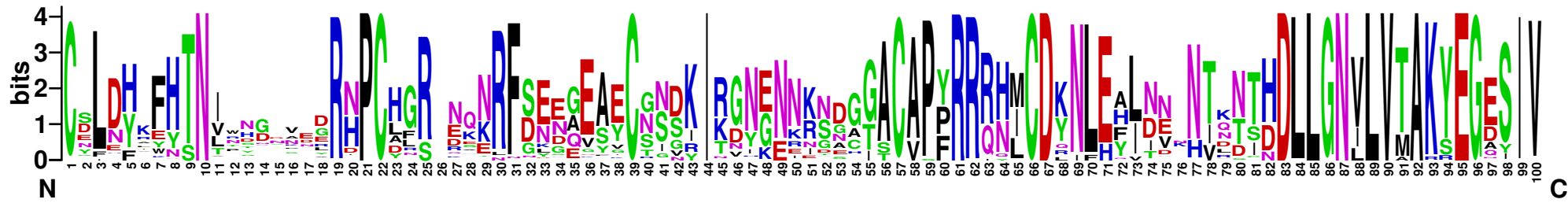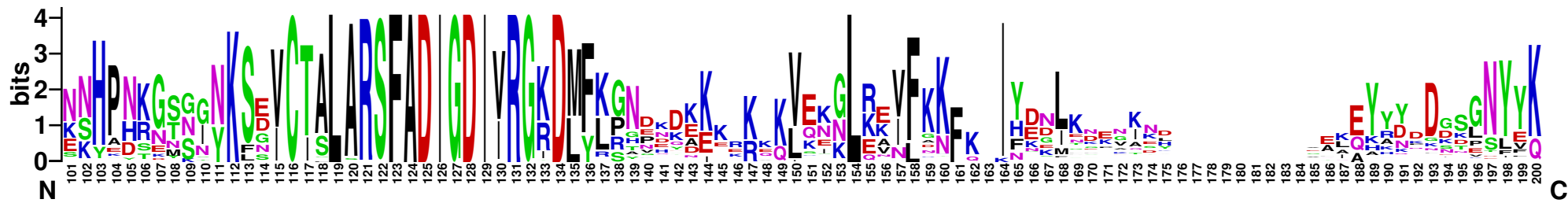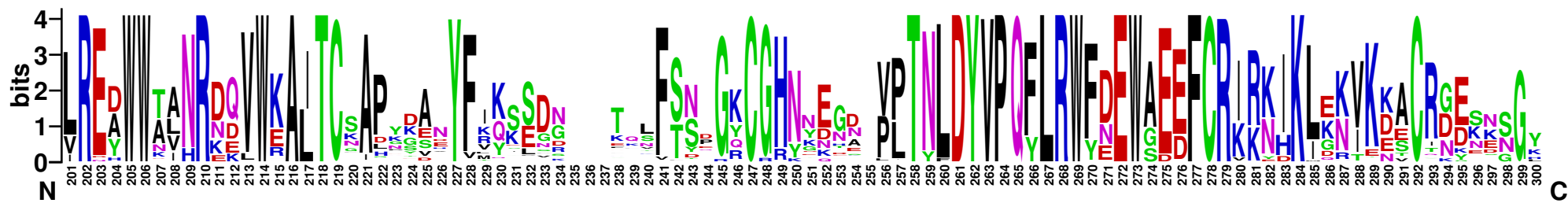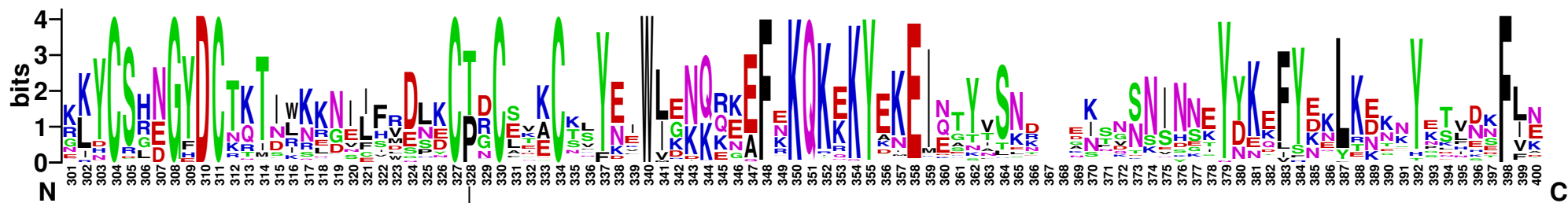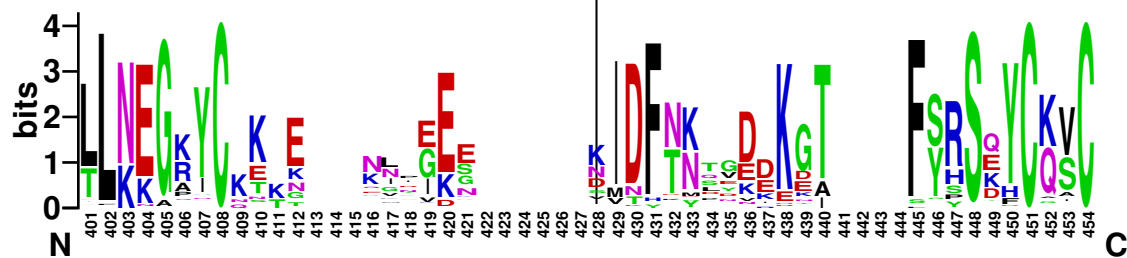

Supplement: Figure S3 — PfEMP1 domain class logos. Sequence conservation logos for major PfEMP1 domain classes (panel A–Z): CIDRα, α1, α2, α3, β, δ, γ, pam; DBLα0, α1 (without α1.3), α1.3, β, δ, ε (without ε1, ε2, ε11, ε13, εpam), ε1, ε2, ε11, ε13, εpam4, εpam5, γ, pam1, pam2, pam3, ζ; NTSA, NTSB, and M3AB. (2.42 MB ZIP) [file pcbi.1000933.s004.zip › Figure S3J - Logo DBLa1 without DBLa1.3.pdf]

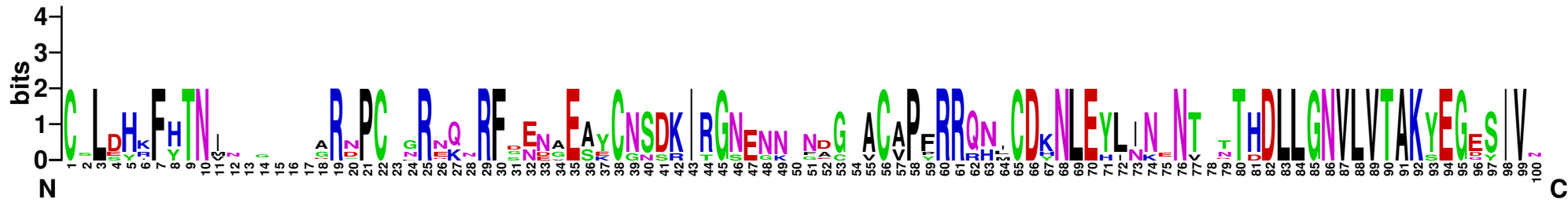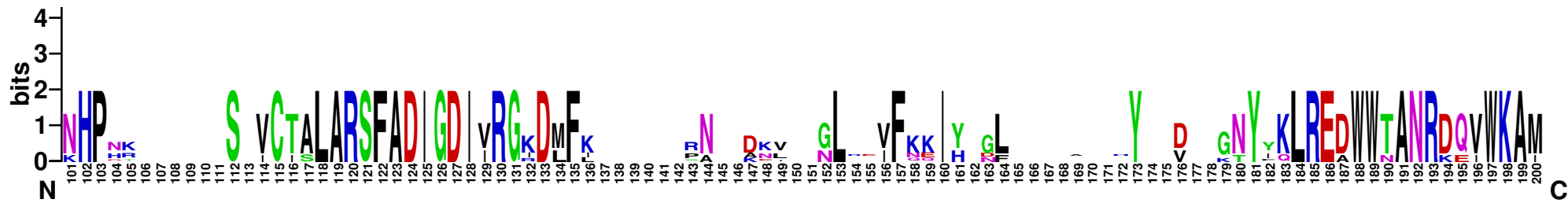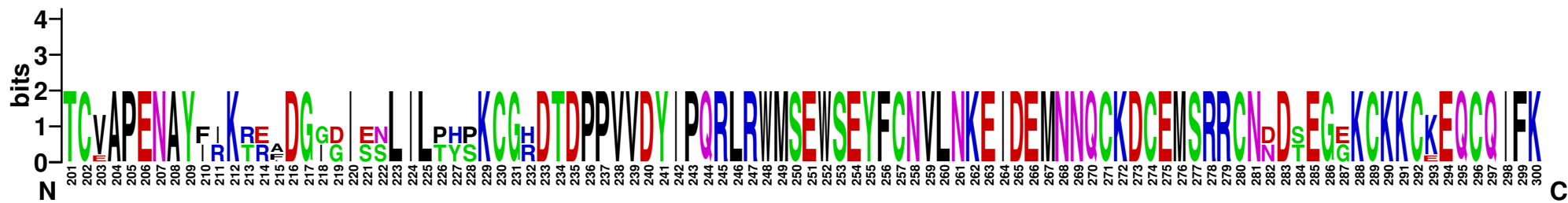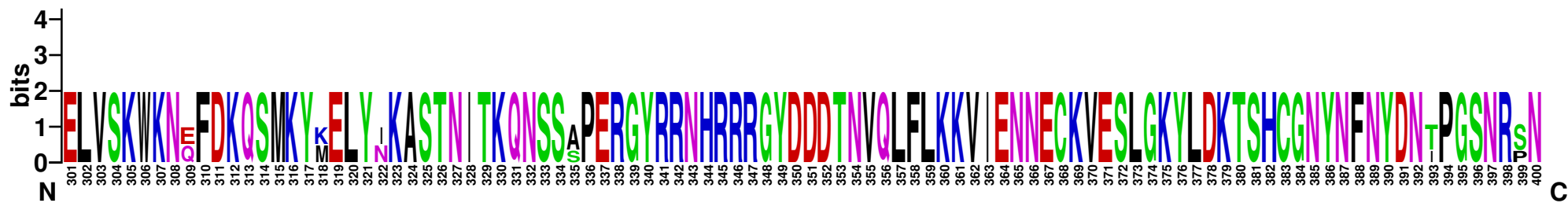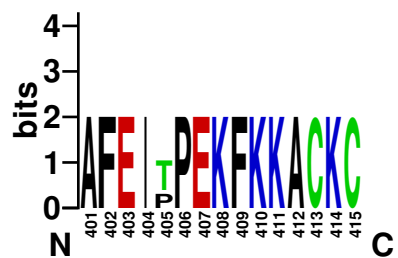

Supplement: Figure S3 — PfEMP1 domain class logos. Sequence conservation logos for major PfEMP1 domain classes (panel A–Z): CIDRα, α1, α2, α3, β, δ, γ, pam; DBLα0, α1 (without α1.3), α1.3, β, δ, ε (without ε1, ε2, ε11, ε13, εpam), ε1, ε2, ε11, ε13, εpam4, εpam5, γ, pam1, pam2, pam3, ζ; NTSA, NTSB, and M3AB. (2.42 MB ZIP) [file pcbi.1000933.s004.zip › Figure S3K - Logo DBLa1.3.pdf]

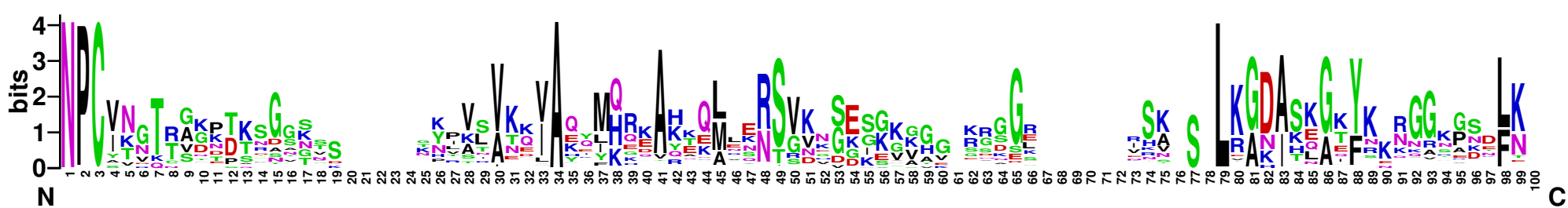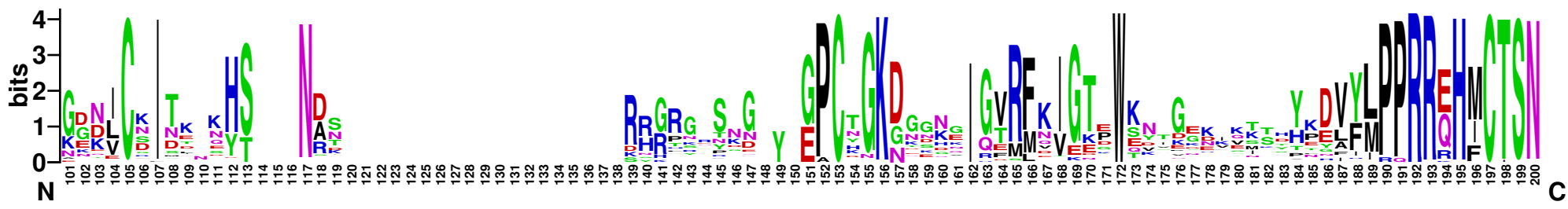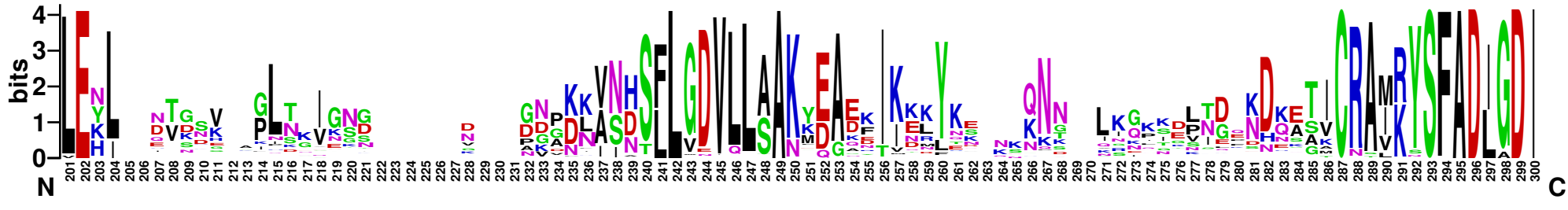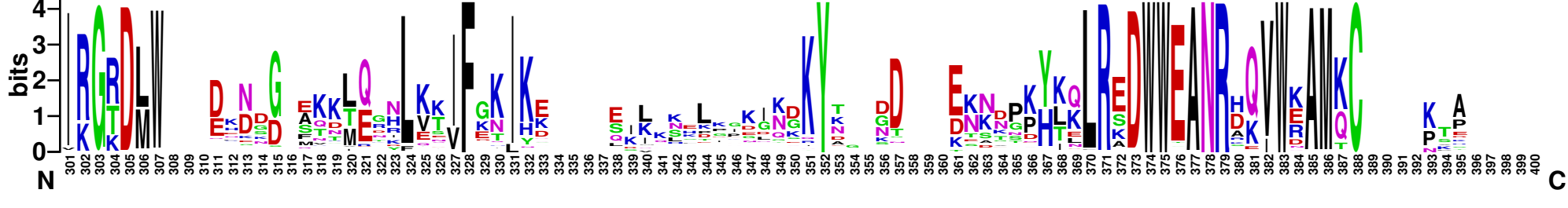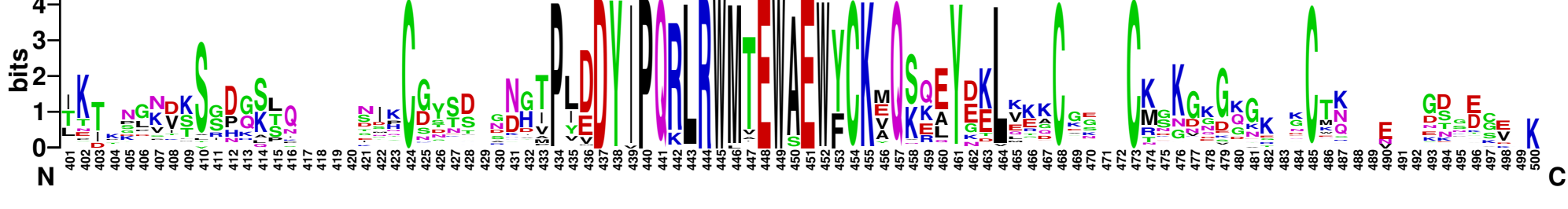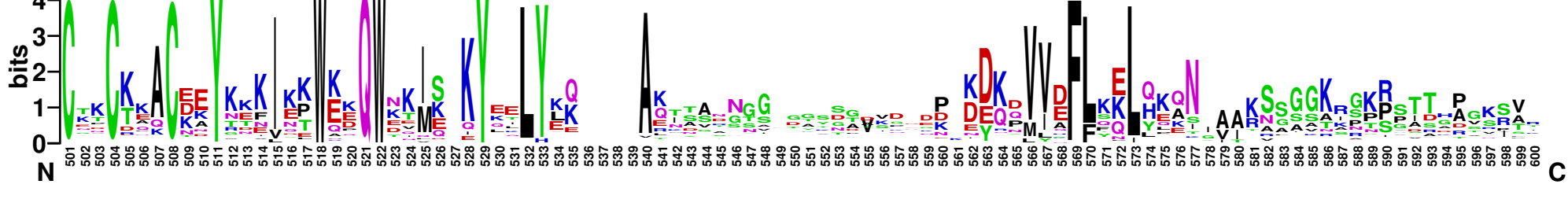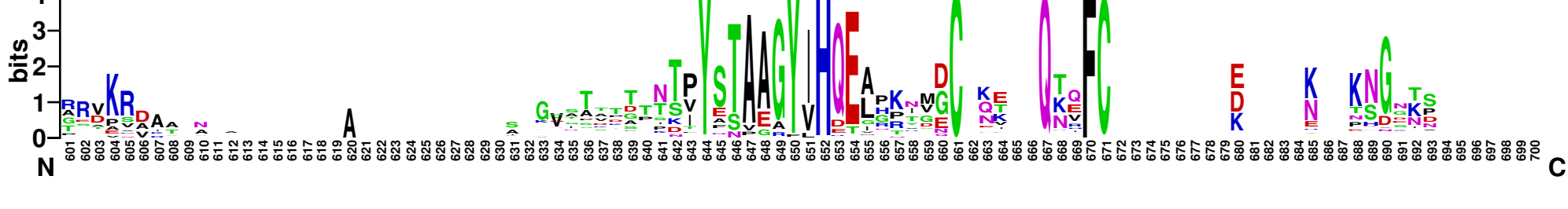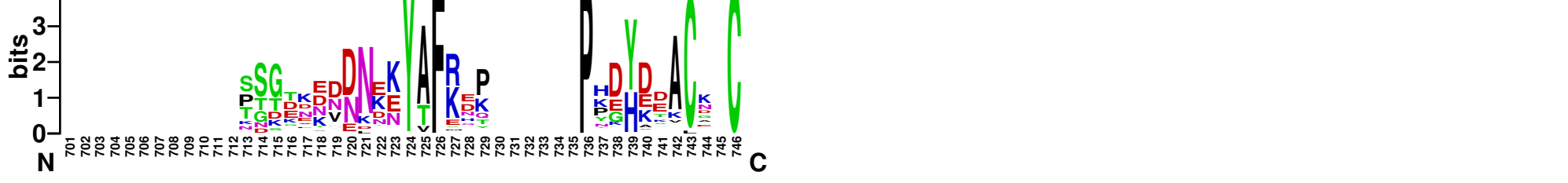

Supplement: Figure S3 — PfEMP1 domain class logos. Sequence conservation logos for major PfEMP1 domain classes (panel A–Z): CIDRα, α1, α2, α3, β, δ, γ, pam; DBLα0, α1 (without α1.3), α1.3, β, δ, ε (without ε1, ε2, ε11, ε13, εpam), ε1, ε2, ε11, ε13, εpam4, εpam5, γ, pam1, pam2, pam3, ζ; NTSA, NTSB, and M3AB. (2.42 MB ZIP) [file pcbi.1000933.s004.zip › Figure S3L - Logo DBLb.pdf]

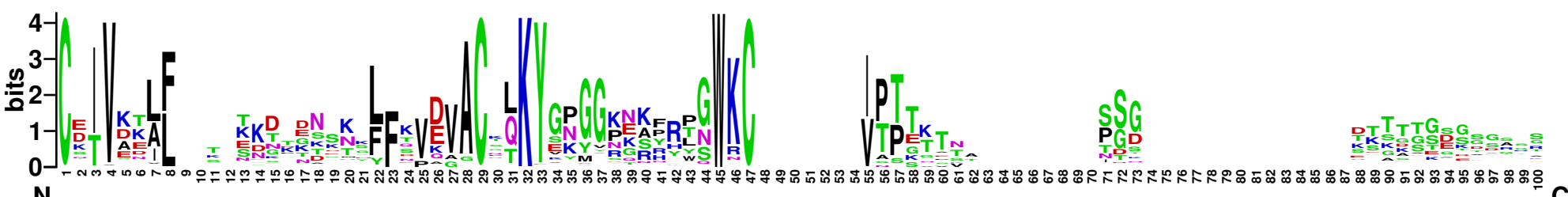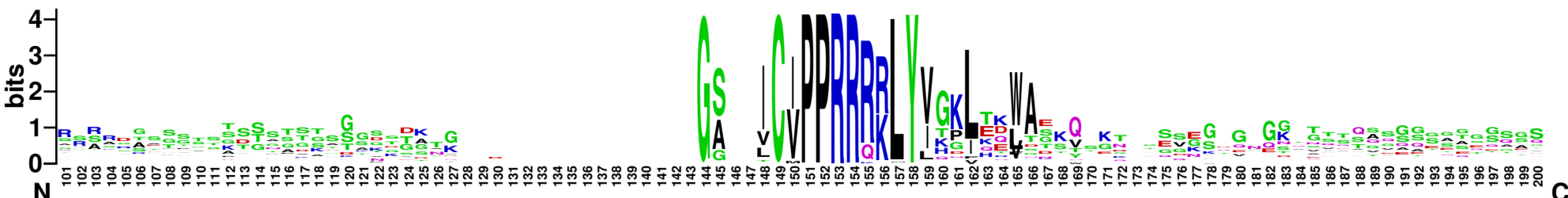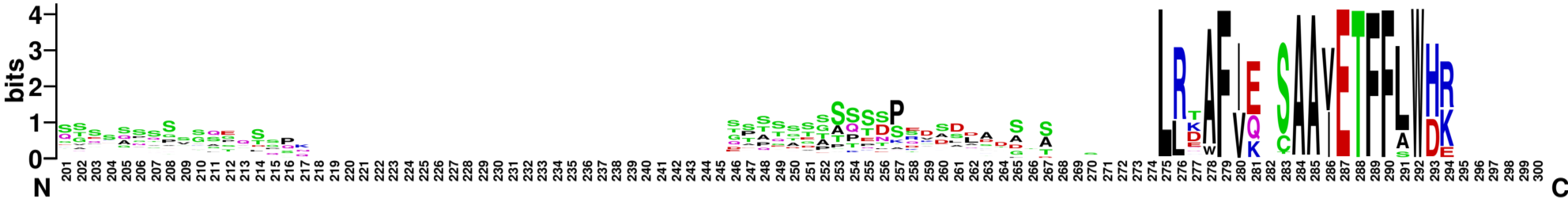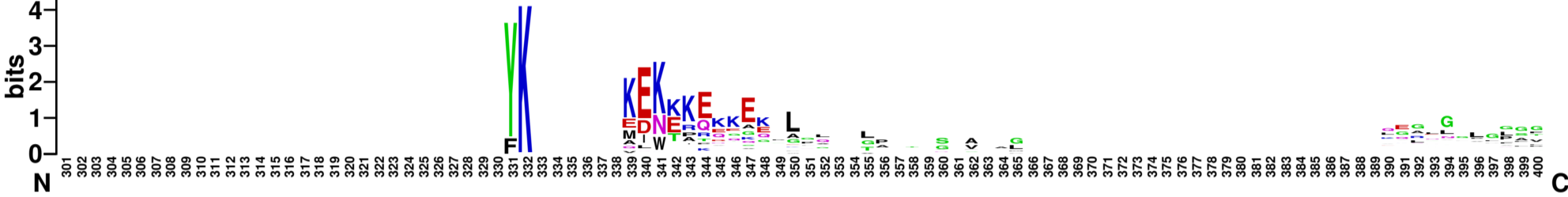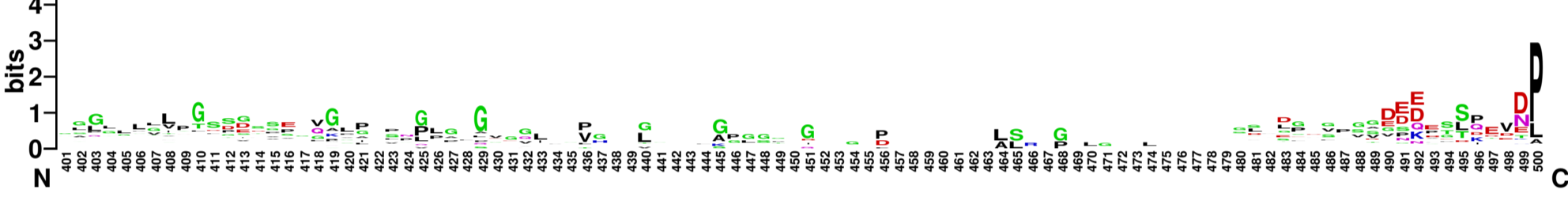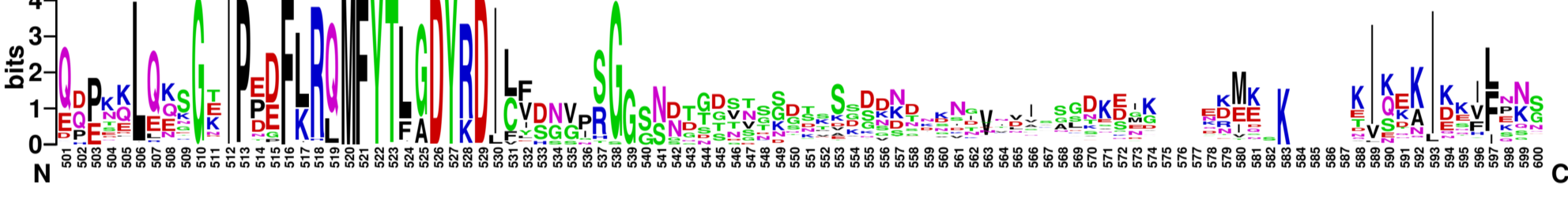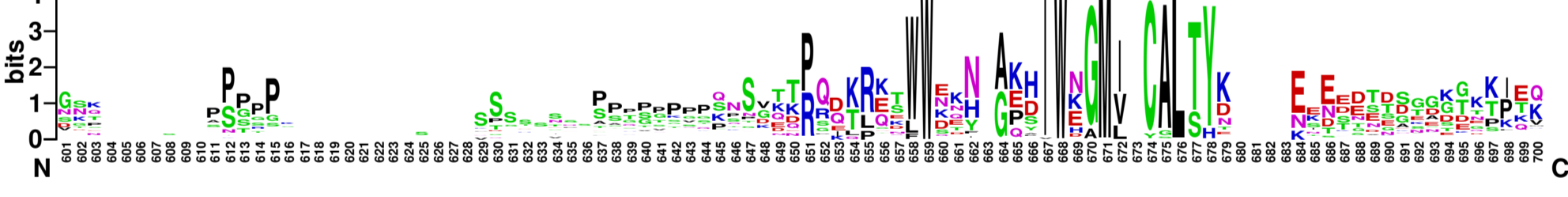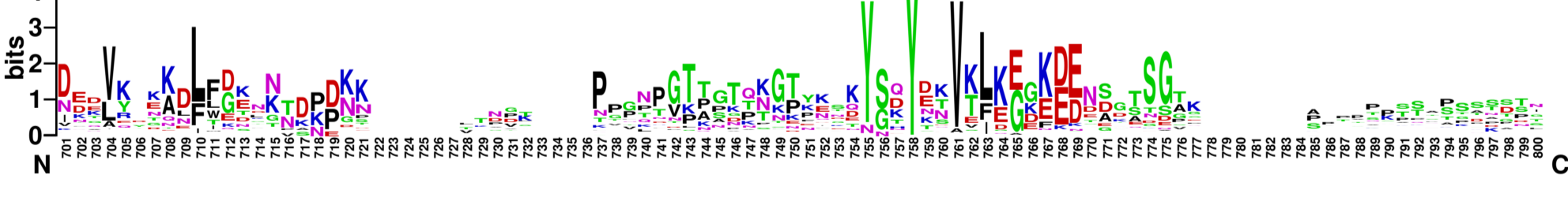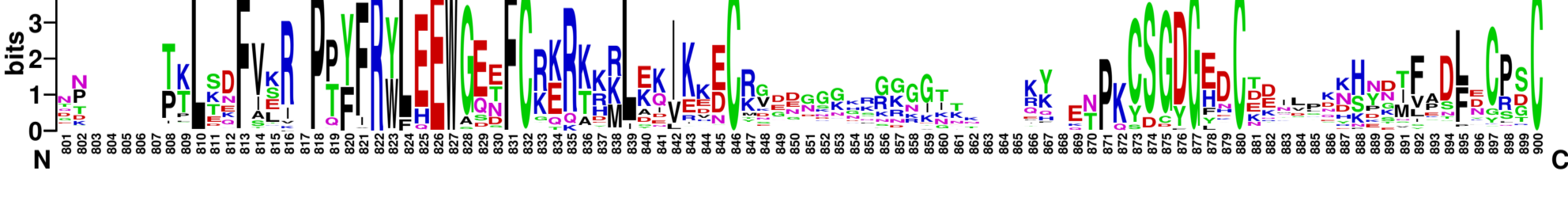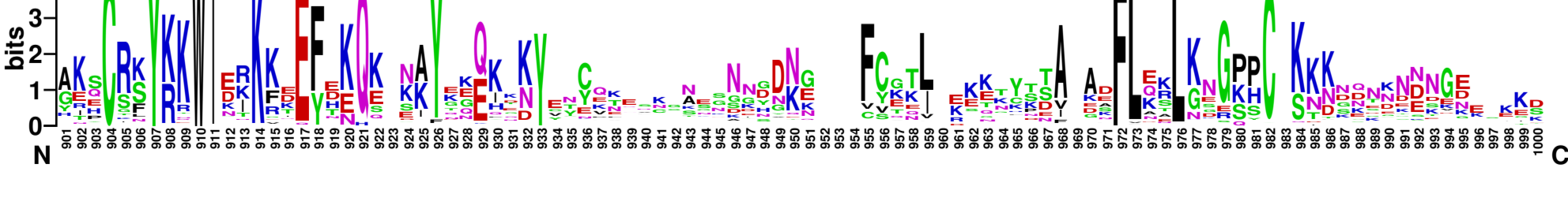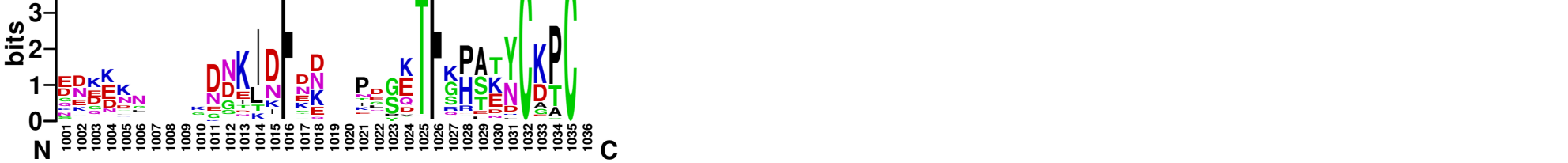

Supplement: Figure S3 — PfEMP1 domain class logos. Sequence conservation logos for major PfEMP1 domain classes (panel A–Z): CIDRα, α1, α2, α3, β, δ, γ, pam; DBLα0, α1 (without α1.3), α1.3, β, δ, ε (without ε1, ε2, ε11, ε13, εpam), ε1, ε2, ε11, ε13, εpam4, εpam5, γ, pam1, pam2, pam3, ζ; NTSA, NTSB, and M3AB. (2.42 MB ZIP) [file pcbi.1000933.s004.zip › Figure S3M - Logo DBLd.pdf]

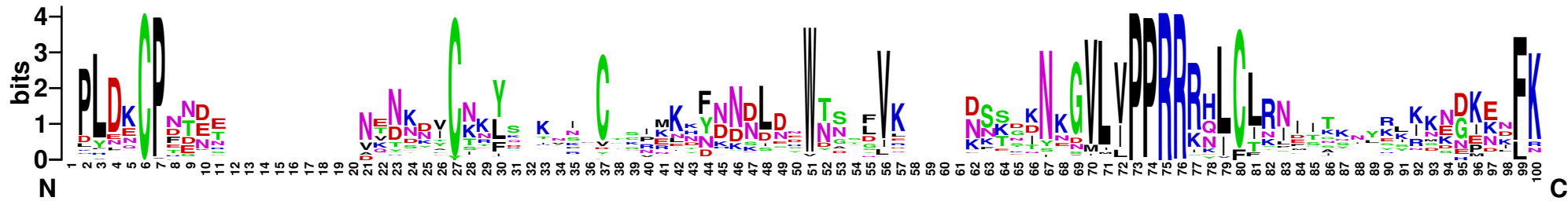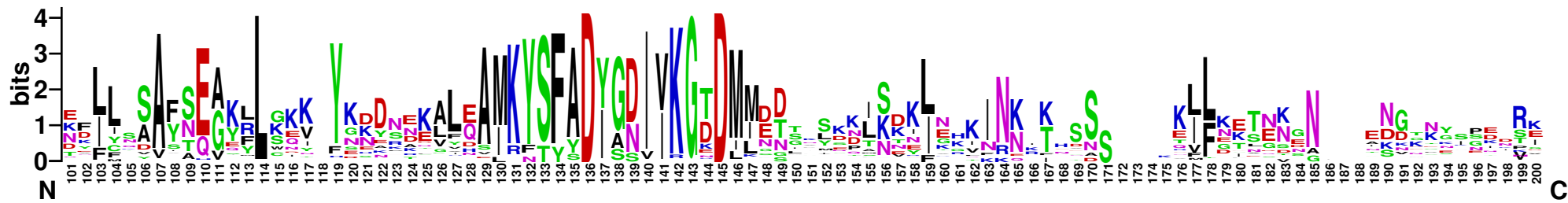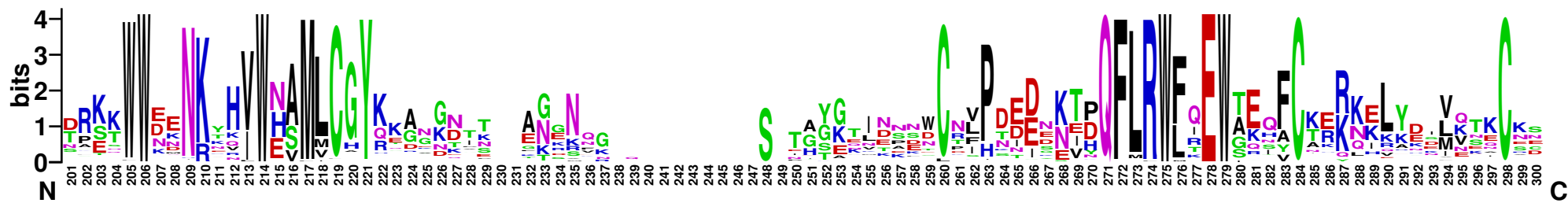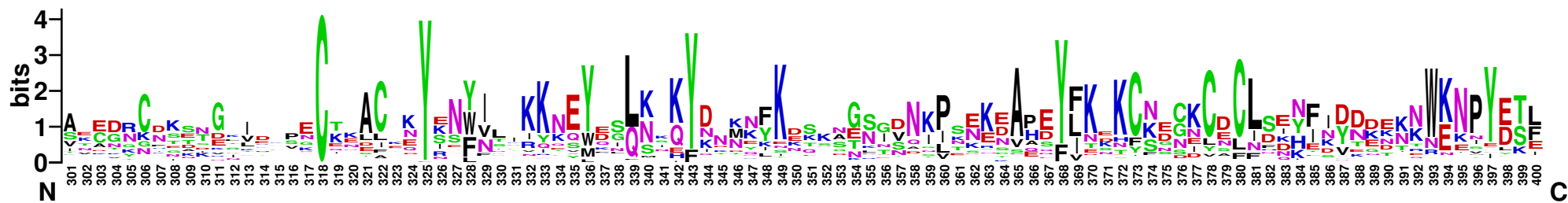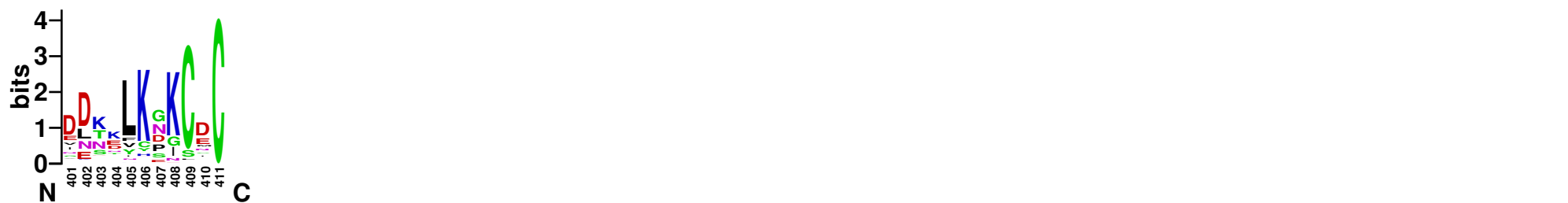

Supplement: Figure S3 — PfEMP1 domain class logos. Sequence conservation logos for major PfEMP1 domain classes (panel A–Z): CIDRα, α1, α2, α3, β, δ, γ, pam; DBLα0, α1 (without α1.3), α1.3, β, δ, ε (without ε1, ε2, ε11, ε13, εpam), ε1, ε2, ε11, ε13, εpam4, εpam5, γ, pam1, pam2, pam3, ζ; NTSA, NTSB, and M3AB. (2.42 MB ZIP) [file pcbi.1000933.s004.zip › Figure S3N - Logo DBLe without DBLe2 DBLepam DBLe1.pdf]

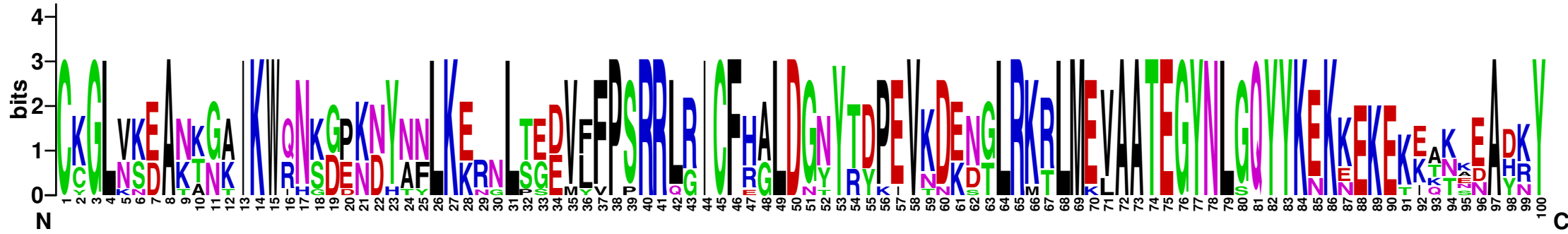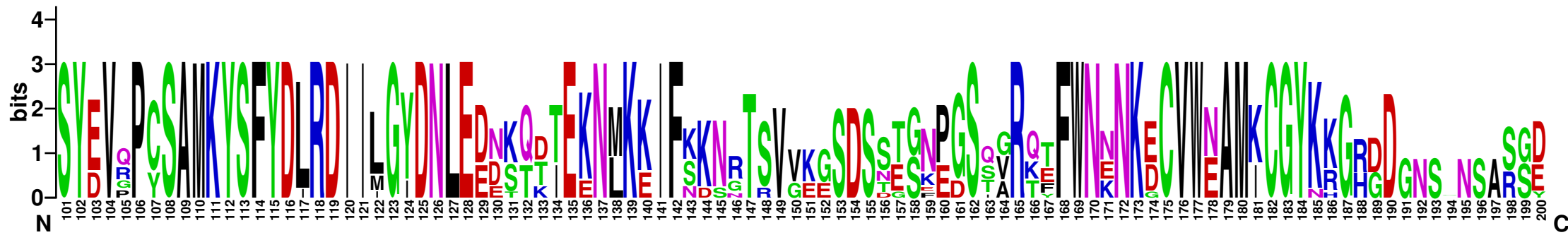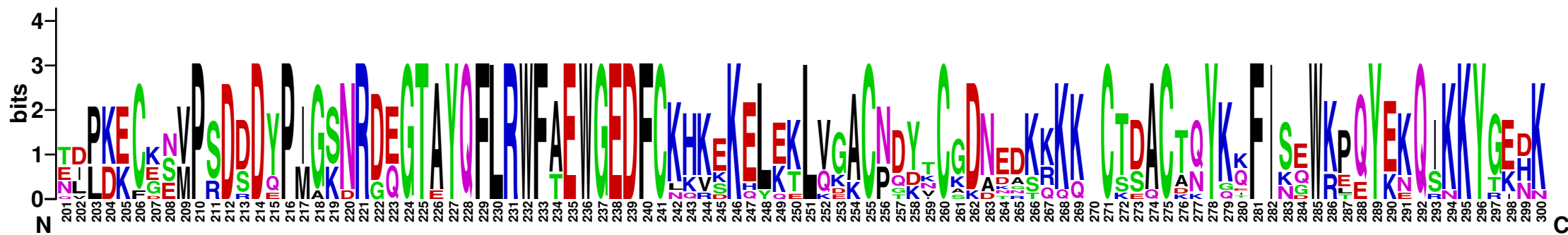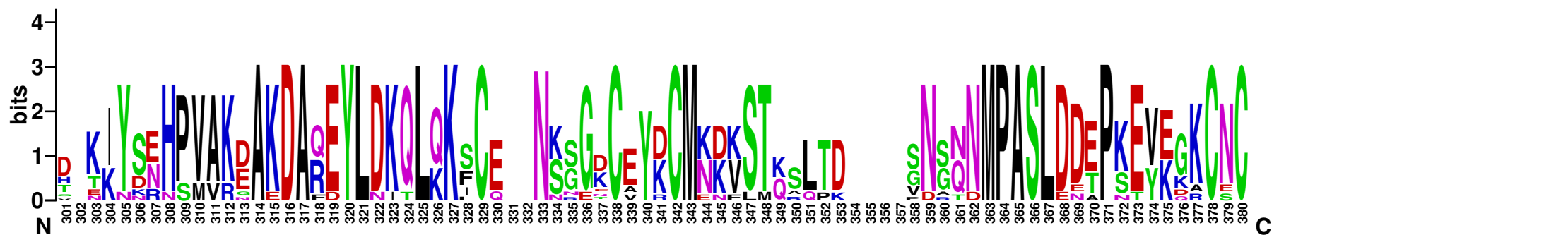

Supplement: Figure S3 — PfEMP1 domain class logos. Sequence conservation logos for major PfEMP1 domain classes (panel A–Z): CIDRα, α1, α2, α3, β, δ, γ, pam; DBLα0, α1 (without α1.3), α1.3, β, δ, ε (without ε1, ε2, ε11, ε13, εpam), ε1, ε2, ε11, ε13, εpam4, εpam5, γ, pam1, pam2, pam3, ζ; NTSA, NTSB, and M3AB. (2.42 MB ZIP) [file pcbi.1000933.s004.zip › Figure S3O - Logo DBLe1.pdf]

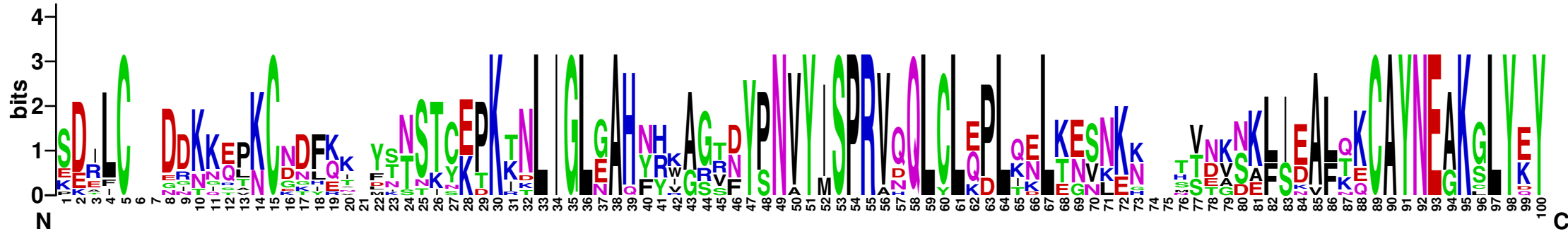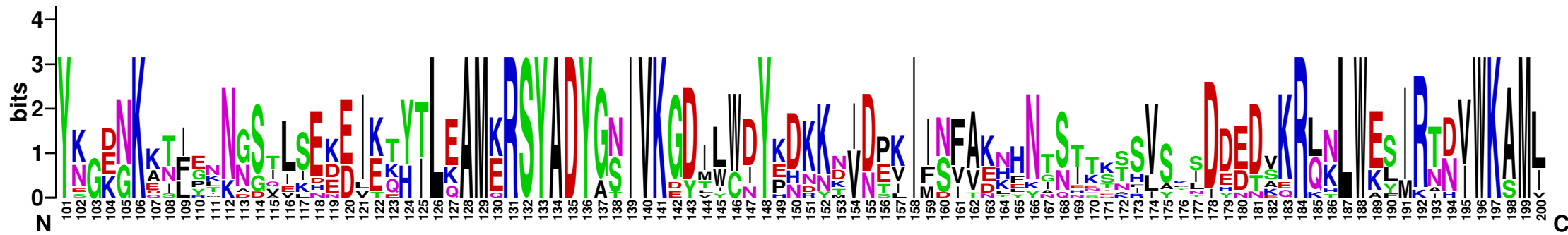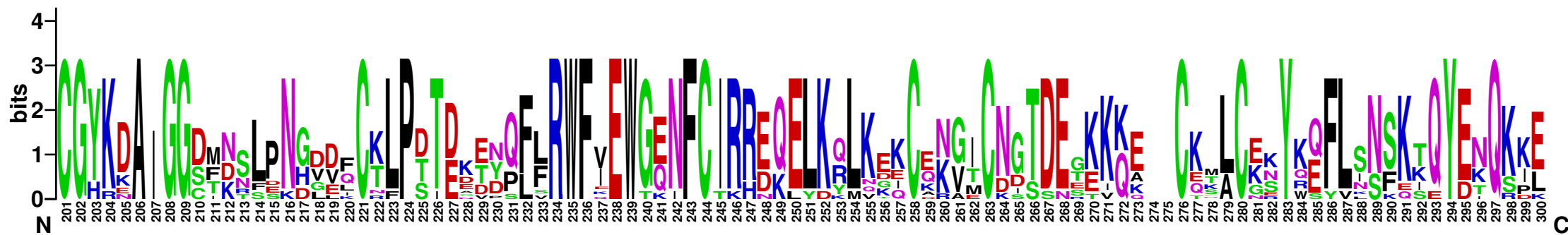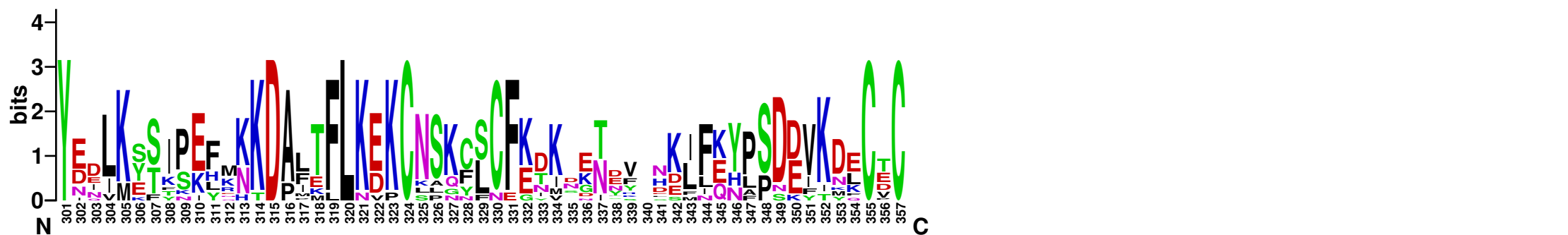

Supplement: Figure S3 — PfEMP1 domain class logos. Sequence conservation logos for major PfEMP1 domain classes (panel A–Z): CIDRα, α1, α2, α3, β, δ, γ, pam; DBLα0, α1 (without α1.3), α1.3, β, δ, ε (without ε1, ε2, ε11, ε13, εpam), ε1, ε2, ε11, ε13, εpam4, εpam5, γ, pam1, pam2, pam3, ζ; NTSA, NTSB, and M3AB. (2.42 MB ZIP) [file pcbi.1000933.s004.zip › Figure S3P - Logo DBLe2.pdf]

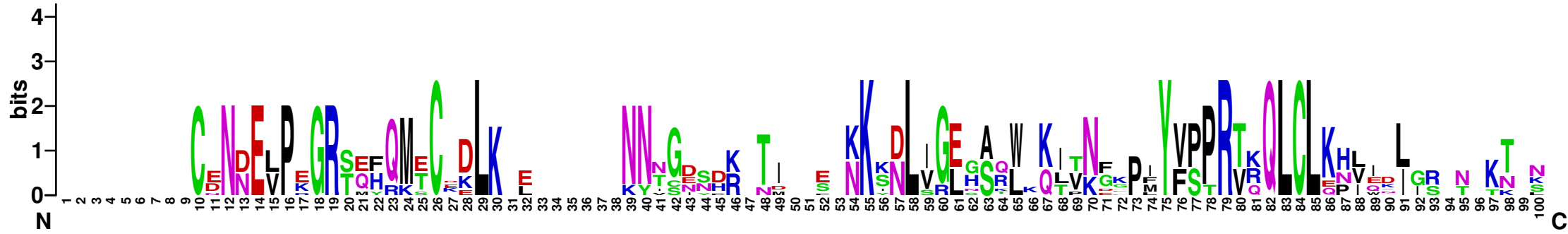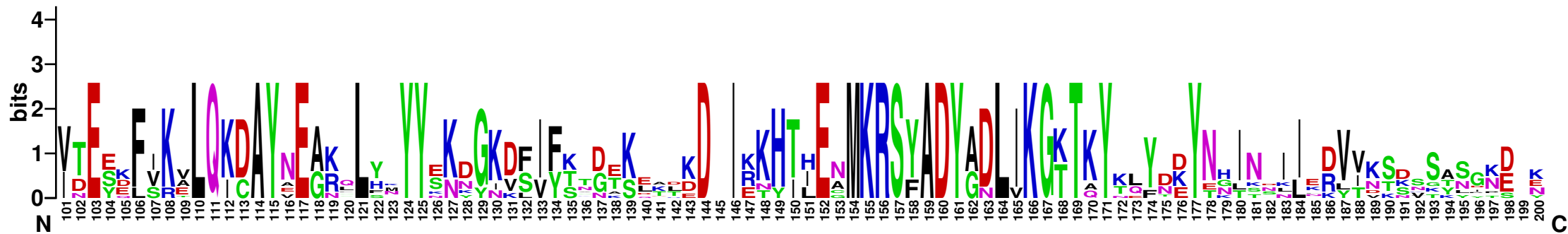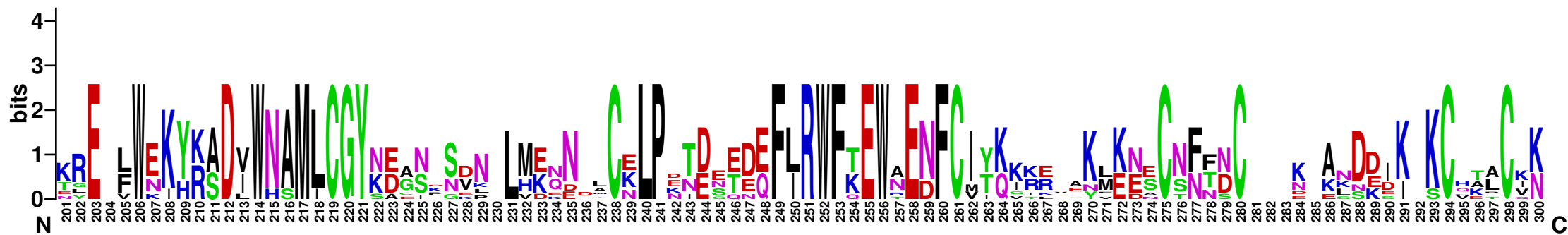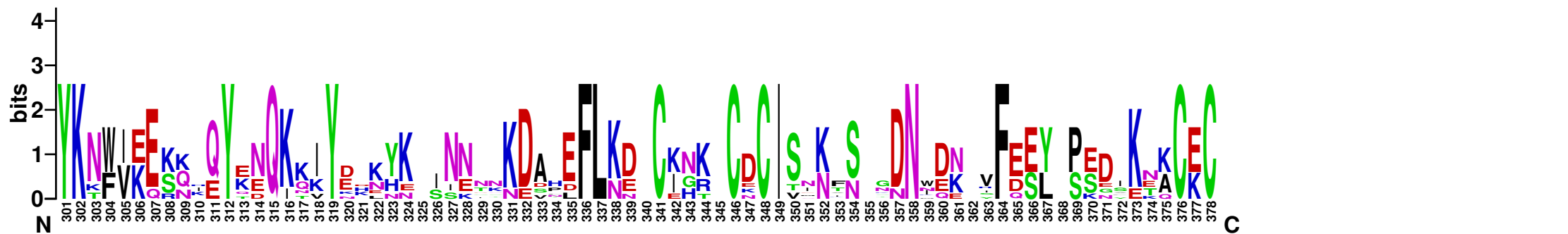

Supplement: Figure S3 — PfEMP1 domain class logos. Sequence conservation logos for major PfEMP1 domain classes (panel A–Z): CIDRα, α1, α2, α3, β, δ, γ, pam; DBLα0, α1 (without α1.3), α1.3, β, δ, ε (without ε1, ε2, ε11, ε13, εpam), ε1, ε2, ε11, ε13, εpam4, εpam5, γ, pam1, pam2, pam3, ζ; NTSA, NTSB, and M3AB. (2.42 MB ZIP) [file pcbi.1000933.s004.zip › Figure S3Q - Logo DBLe11.pdf]

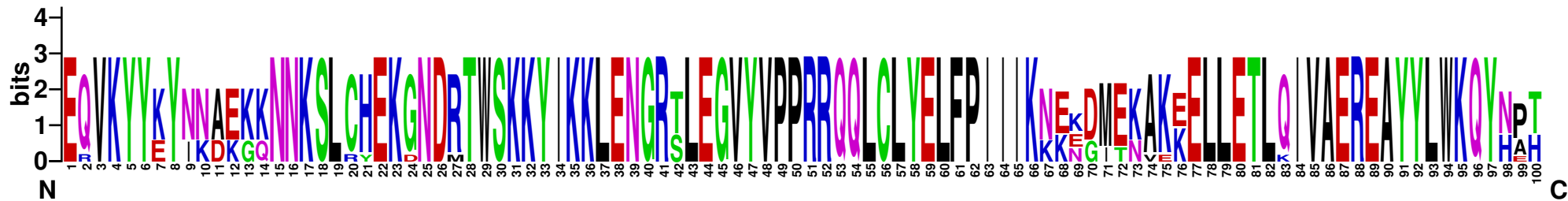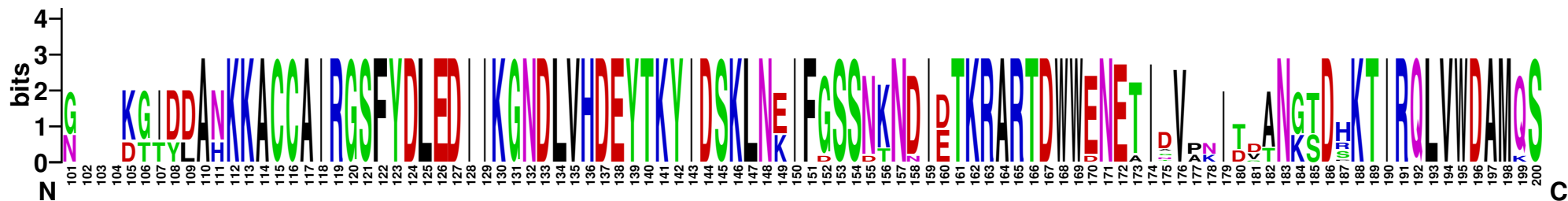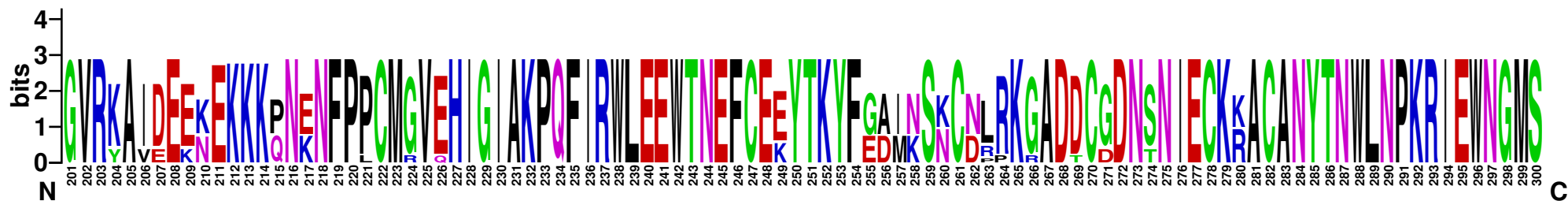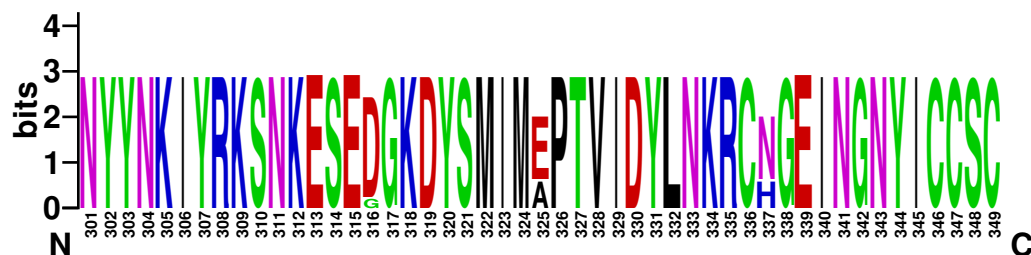

Supplement: Figure S3 — PfEMP1 domain class logos. Sequence conservation logos for major PfEMP1 domain classes (panel A–Z): CIDRα, α1, α2, α3, β, δ, γ, pam; DBLα0, α1 (without α1.3), α1.3, β, δ, ε (without ε1, ε2, ε11, ε13, εpam), ε1, ε2, ε11, ε13, εpam4, εpam5, γ, pam1, pam2, pam3, ζ; NTSA, NTSB, and M3AB. (2.42 MB ZIP) [file pcbi.1000933.s004.zip › Figure S3R - Logo DBLepam4.pdf]

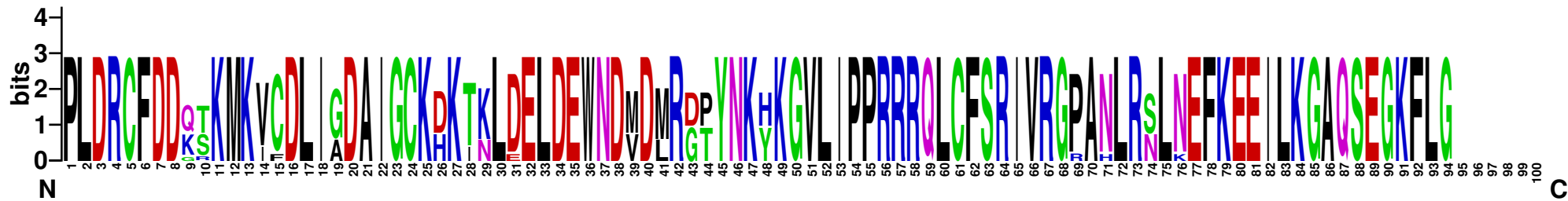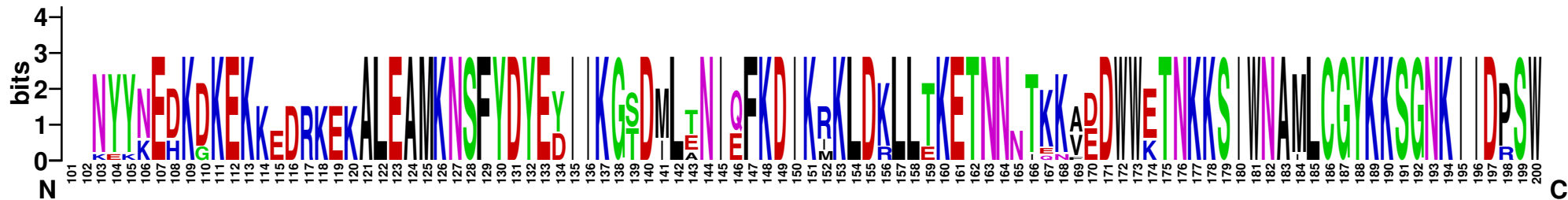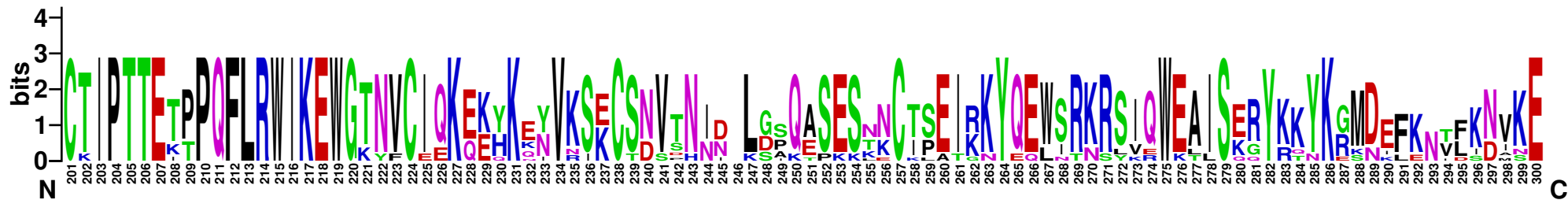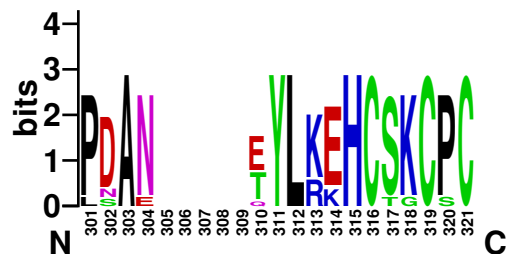

Supplement: Figure S3 — PfEMP1 domain class logos. Sequence conservation logos for major PfEMP1 domain classes (panel A–Z): CIDRα, α1, α2, α3, β, δ, γ, pam; DBLα0, α1 (without α1.3), α1.3, β, δ, ε (without ε1, ε2, ε11, ε13, εpam), ε1, ε2, ε11, ε13, εpam4, εpam5, γ, pam1, pam2, pam3, ζ; NTSA, NTSB, and M3AB. (2.42 MB ZIP) [file pcbi.1000933.s004.zip › Figure S3S - Logo DBLepam5.pdf]

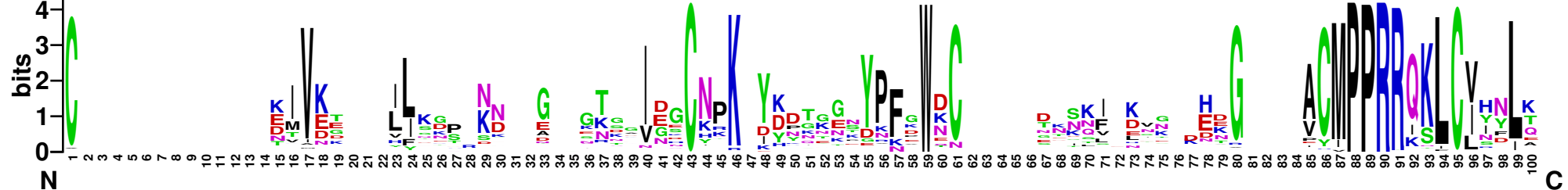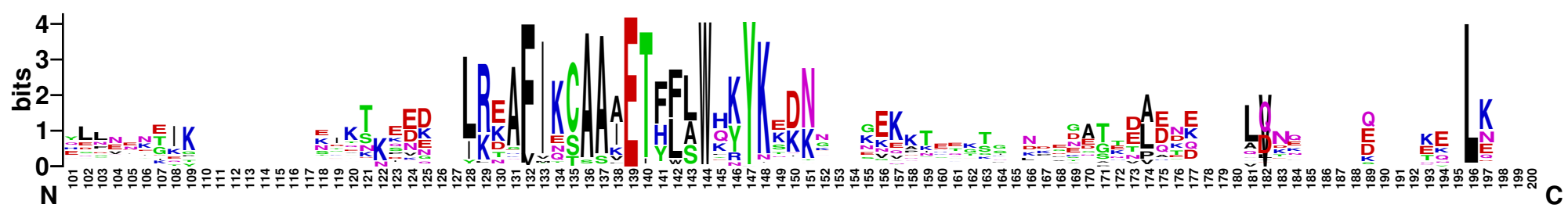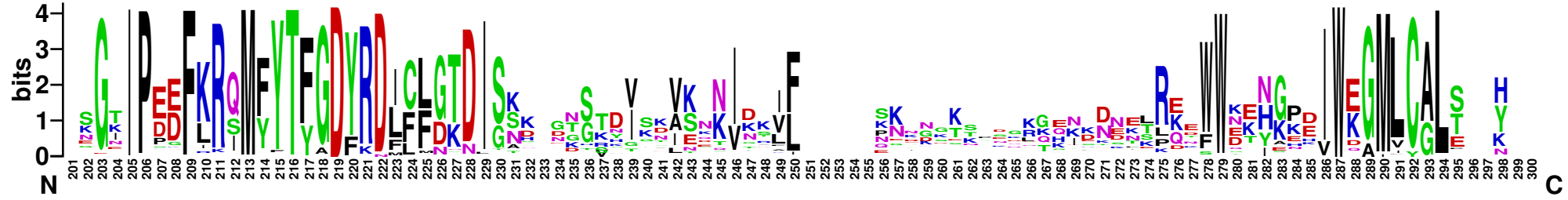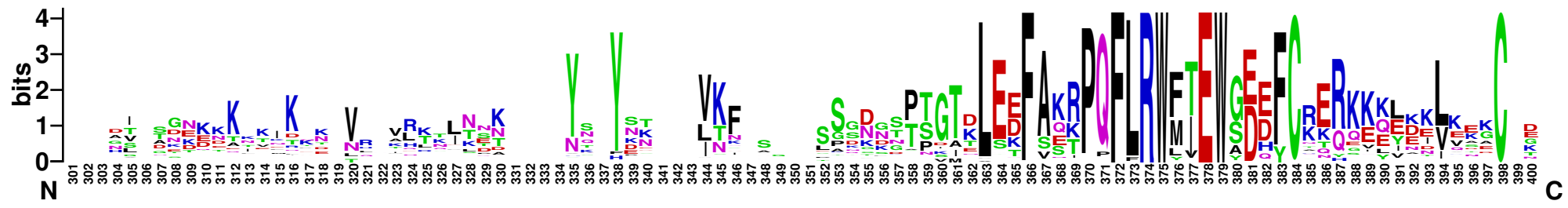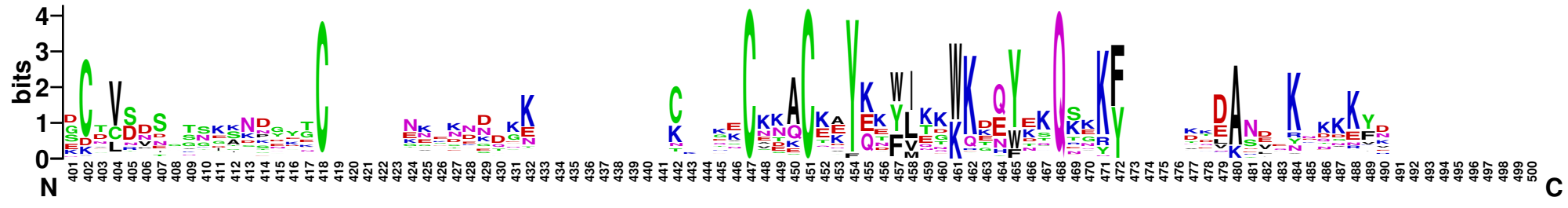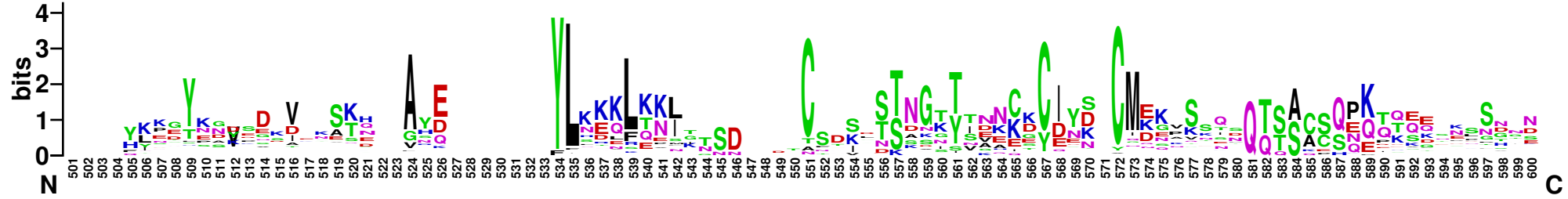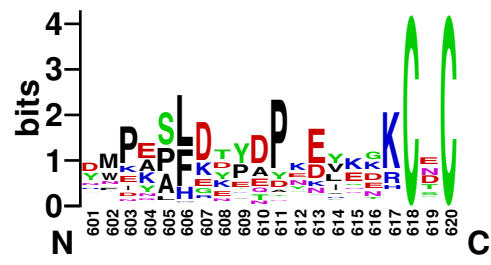

Supplement: Figure S3 — PfEMP1 domain class logos. Sequence conservation logos for major PfEMP1 domain classes (panel A–Z): CIDRα, α1, α2, α3, β, δ, γ, pam; DBLα0, α1 (without α1.3), α1.3, β, δ, ε (without ε1, ε2, ε11, ε13, εpam), ε1, ε2, ε11, ε13, εpam4, εpam5, γ, pam1, pam2, pam3, ζ; NTSA, NTSB, and M3AB. (2.42 MB ZIP) [file pcbi.1000933.s004.zip › Figure S3T - Logo DBLg.pdf]

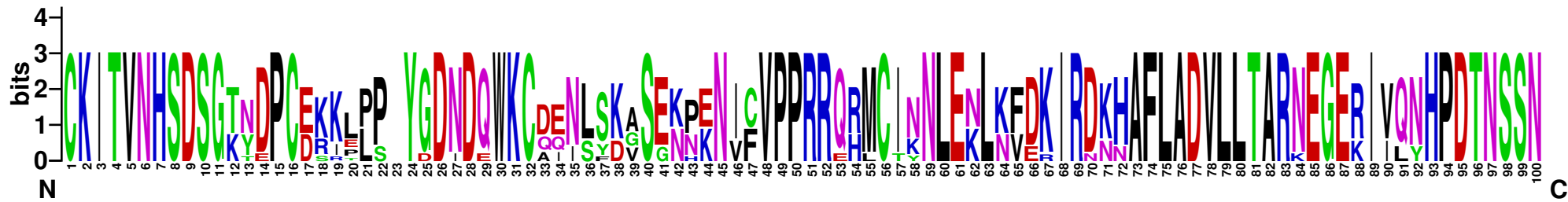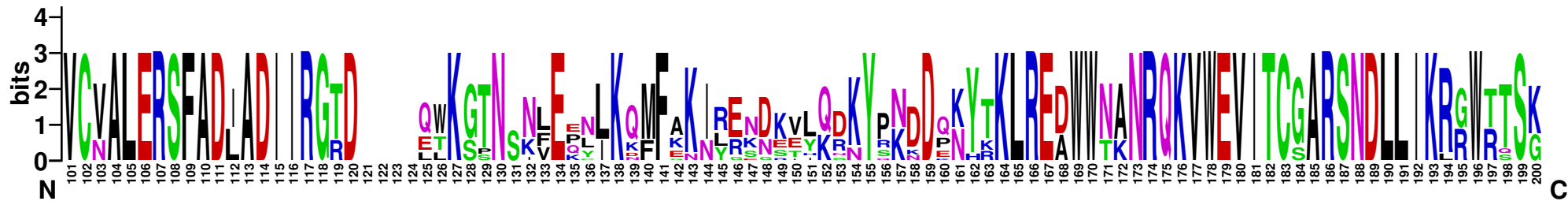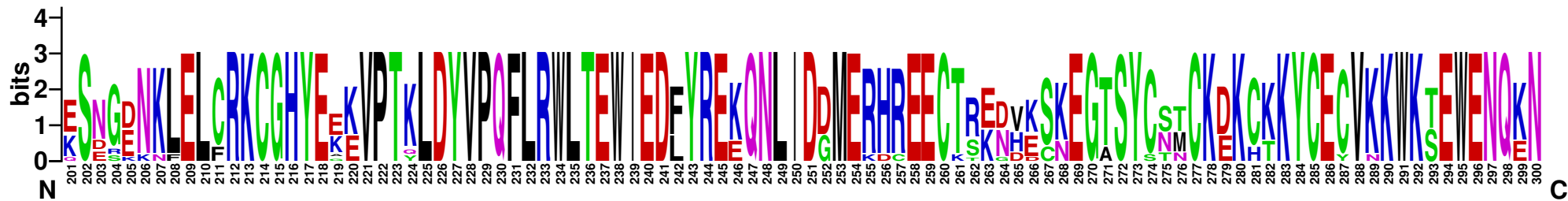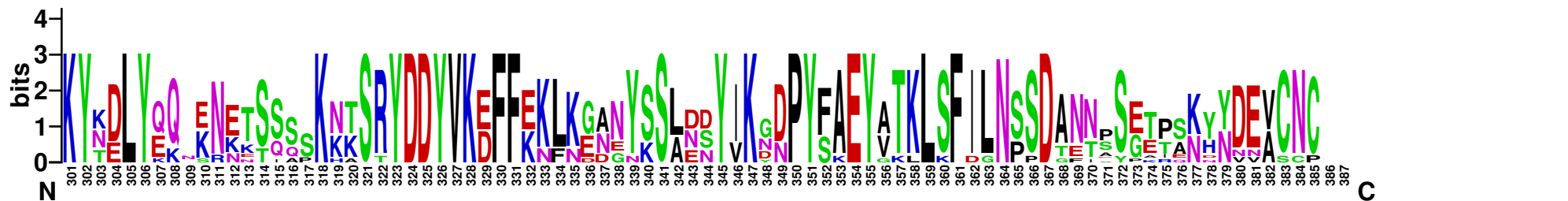

Supplement: Figure S3 — PfEMP1 domain class logos. Sequence conservation logos for major PfEMP1 domain classes (panel A–Z): CIDRα, α1, α2, α3, β, δ, γ, pam; DBLα0, α1 (without α1.3), α1.3, β, δ, ε (without ε1, ε2, ε11, ε13, εpam), ε1, ε2, ε11, ε13, εpam4, εpam5, γ, pam1, pam2, pam3, ζ; NTSA, NTSB, and M3AB. (2.42 MB ZIP) [file pcbi.1000933.s004.zip › Figure S3U - Logo DBLpam1.pdf]

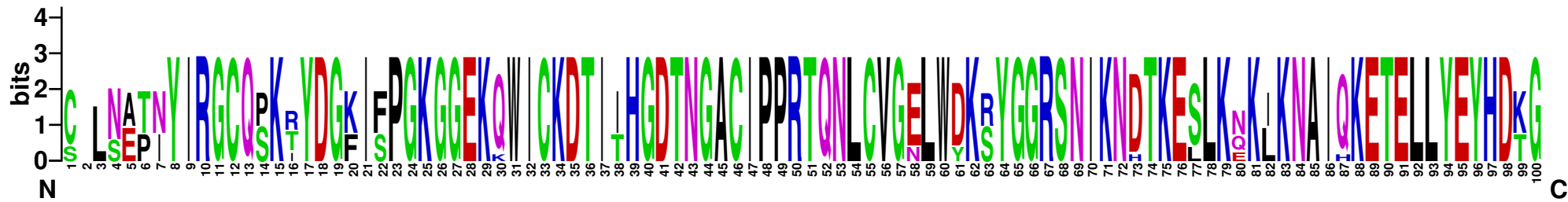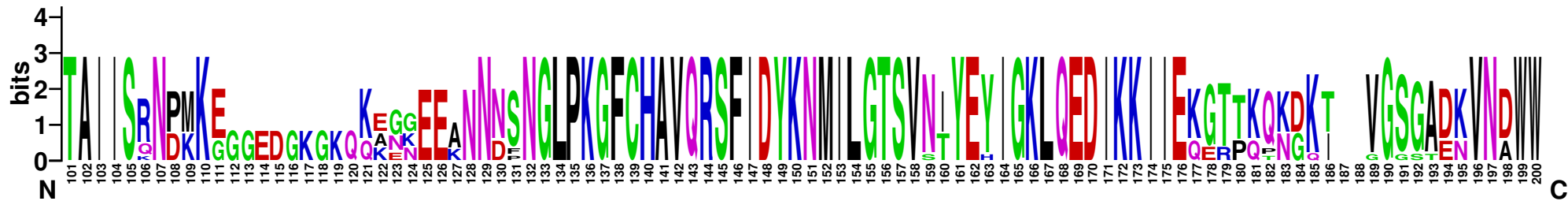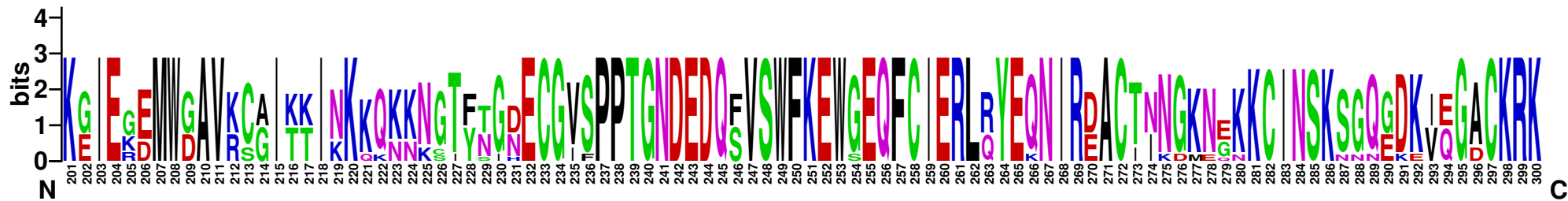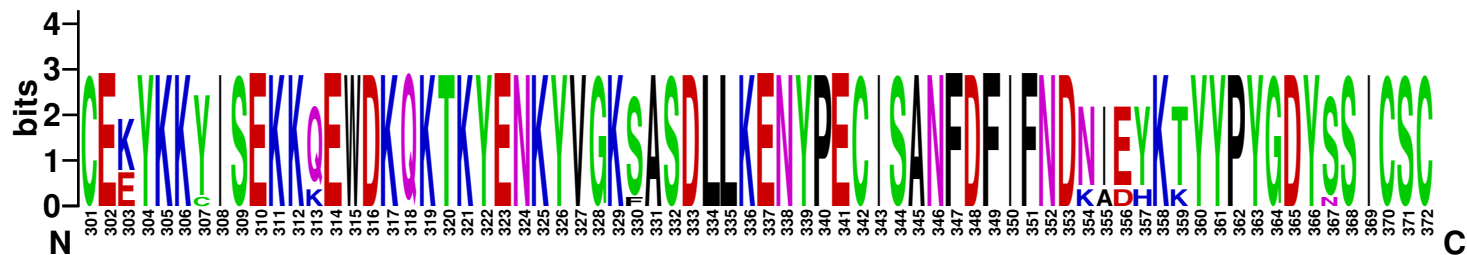

Supplement: Figure S3 — PfEMP1 domain class logos. Sequence conservation logos for major PfEMP1 domain classes (panel A–Z): CIDRα, α1, α2, α3, β, δ, γ, pam; DBLα0, α1 (without α1.3), α1.3, β, δ, ε (without ε1, ε2, ε11, ε13, εpam), ε1, ε2, ε11, ε13, εpam4, εpam5, γ, pam1, pam2, pam3, ζ; NTSA, NTSB, and M3AB. (2.42 MB ZIP) [file pcbi.1000933.s004.zip › Figure S3W - Logo DBLpam3.pdf]

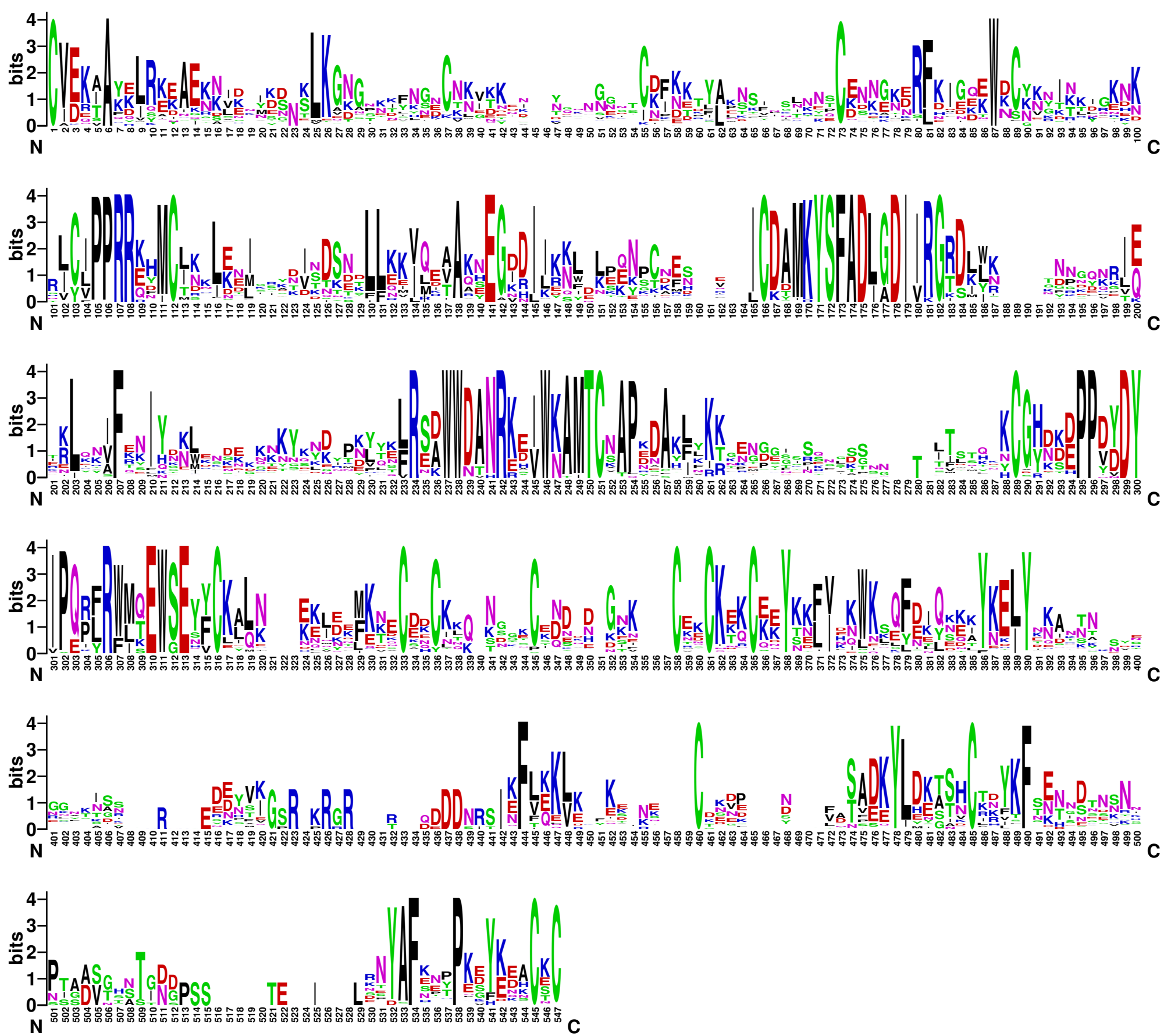

Supplement: Figure S3 — PfEMP1 domain class logos. Sequence conservation logos for major PfEMP1 domain classes (panel A–Z): CIDRα, α1, α2, α3, β, δ, γ, pam; DBLα0, α1 (without α1.3), α1.3, β, δ, ε (without ε1, ε2, ε11, ε13, εpam), ε1, ε2, ε11, ε13, εpam4, εpam5, γ, pam1, pam2, pam3, ζ; NTSA, NTSB, and M3AB. (2.42 MB ZIP) [file pcbi.1000933.s004.zip › Figure S3X - Logo DBLz.pdf]

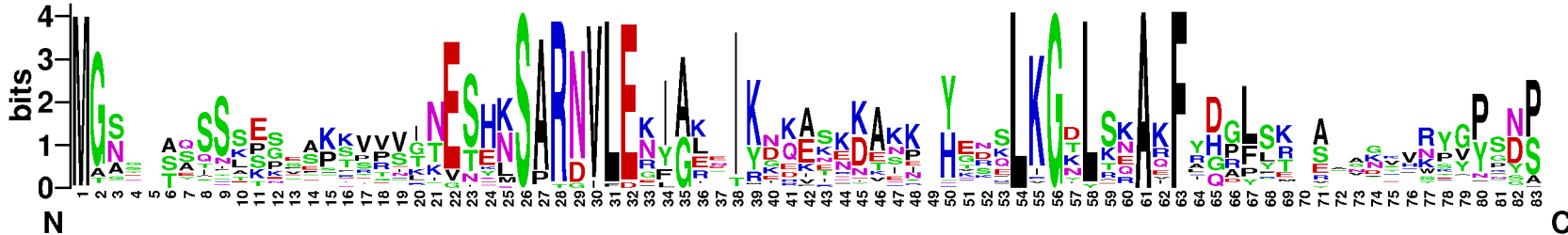

NTSA

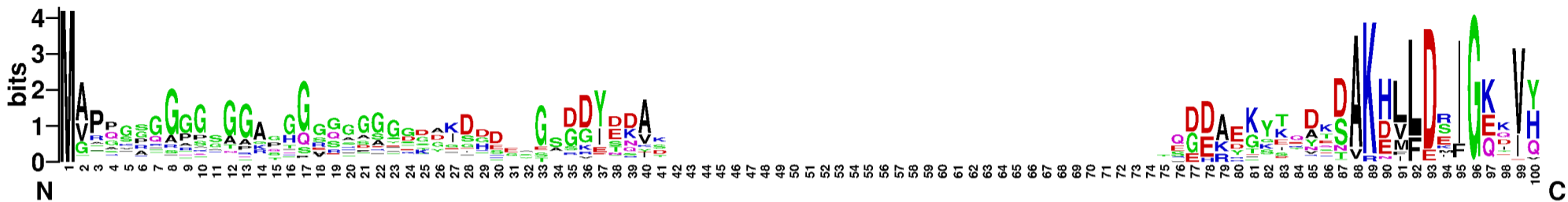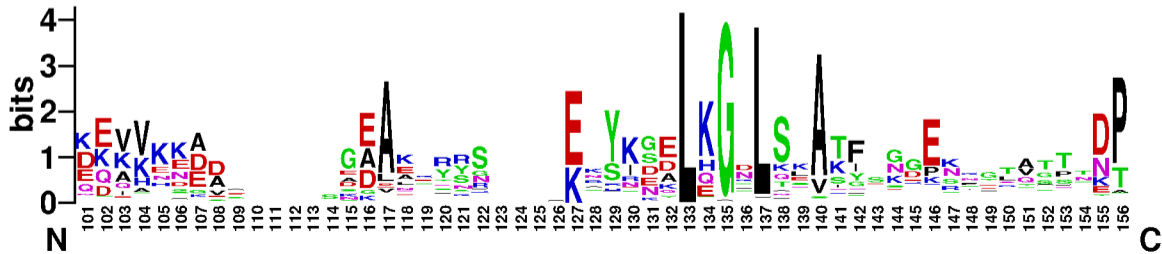

Supplement: Figure S3 — PfEMP1 domain class logos. Sequence conservation logos for major PfEMP1 domain classes (panel A–Z): CIDRα, α1, α2, α3, β, δ, γ, pam; DBLα0, α1 (without α1.3), α1.3, β, δ, ε (without ε1, ε2, ε11, ε13, εpam), ε1, ε2, ε11, ε13, εpam4, εpam5, γ, pam1, pam2, pam3, ζ; NTSA, NTSB, and M3AB. (2.42 MB ZIP) [file pcbi.1000933.s004.zip › Figure S3Y - Logo NTS.pdf]

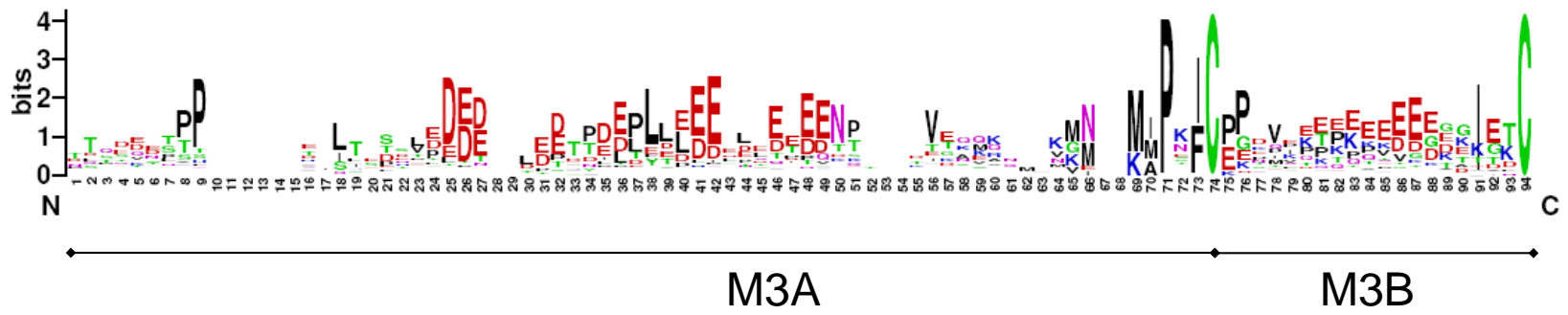

Supplement: Figure S3 — PfEMP1 domain class logos. Sequence conservation logos for major PfEMP1 domain classes (panel A–Z): CIDRα, α1, α2, α3, β, δ, γ, pam; DBLα0, α1 (without α1.3), α1.3, β, δ, ε (without ε1, ε2, ε11, ε13, εpam), ε1, ε2, ε11, ε13, εpam4, εpam5, γ, pam1, pam2, pam3, ζ; NTSA, NTSB, and M3AB. (2.42 MB ZIP) [file pcbi.1000933.s004.zip › Figure S3Z - Logo M3AB.pdf]

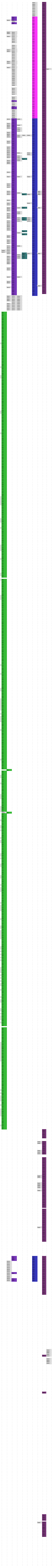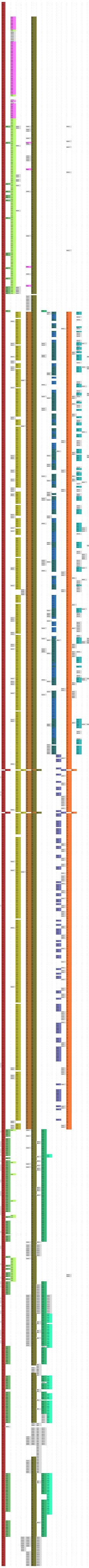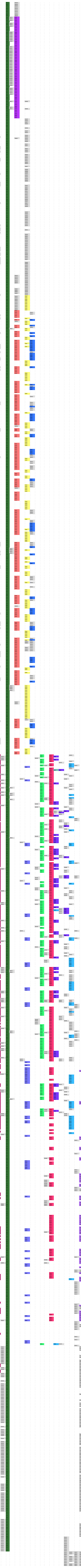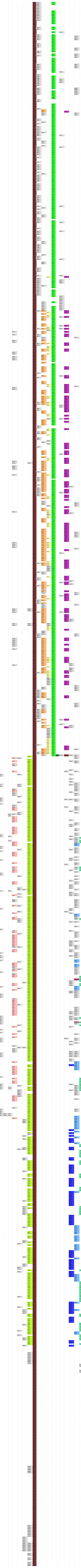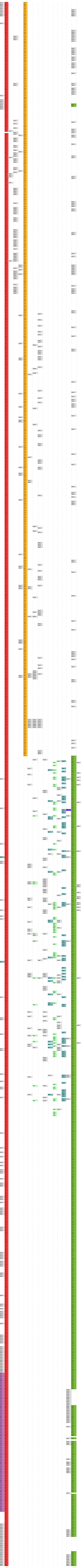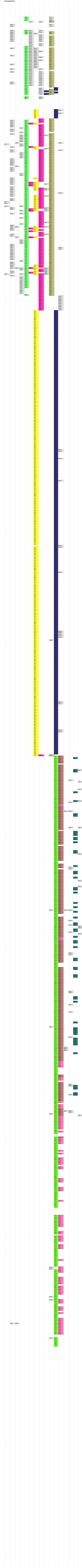

Supplement: Figure S7 — Homology block alignments. Homology block alignments for (panel A–E): DBL, CIDR, NTS, ATS, and whole PfEMP1, with details of Figure 6, Figure 8, Figure 10 and Figure 12. (0.82 MB ZIP) [file pcbi.1000933.s008.zip › Figure S7A - HB alignment - DBL.pdf]

CIDR

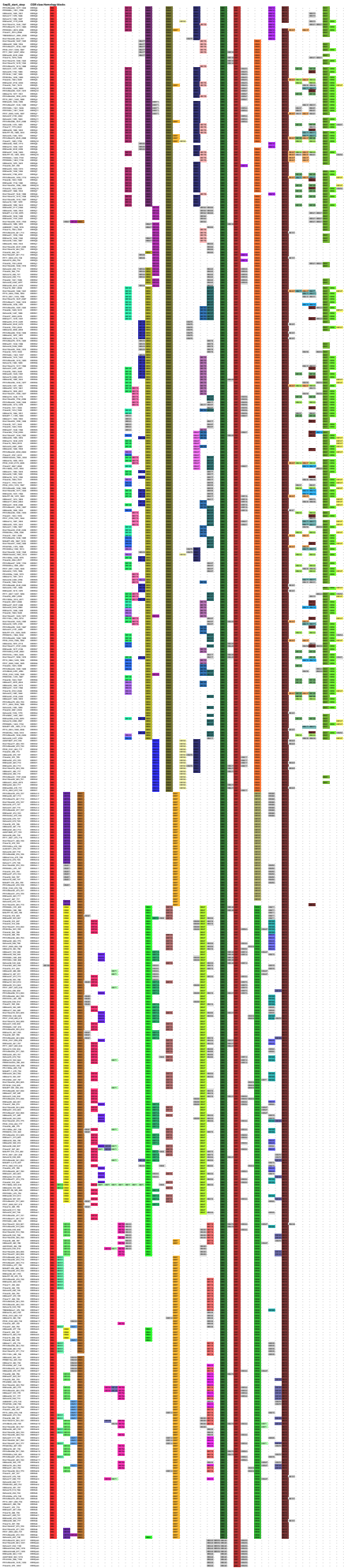

Supplement: Figure S7 — Homology block alignments. Homology block alignments for (panel A–E): DBL, CIDR, NTS, ATS, and whole PfEMP1, with details of Figure 6, Figure 8, Figure 10 and Figure 12. (0.82 MB ZIP) [file pcbi.1000933.s008.zip › Figure S7B - HB alignment - CIDR.pdf]

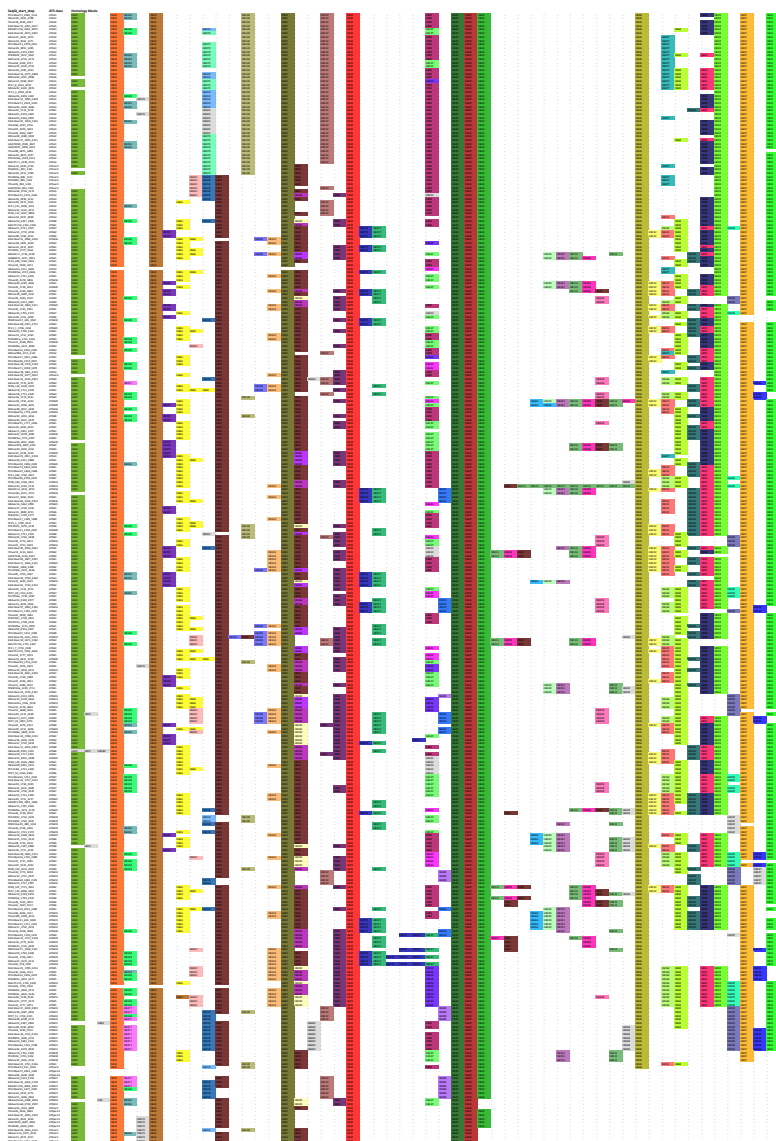

Supplement: Figure S7 — Homology block alignments. Homology block alignments for (panel A–E): DBL, CIDR, NTS, ATS, and whole PfEMP1, with details of Figure 6, Figure 8, Figure 10 and Figure 12. (0.82 MB ZIP) [file pcbi.1000933.s008.zip › Figure S7D - HB alignment - ATS.pdf]

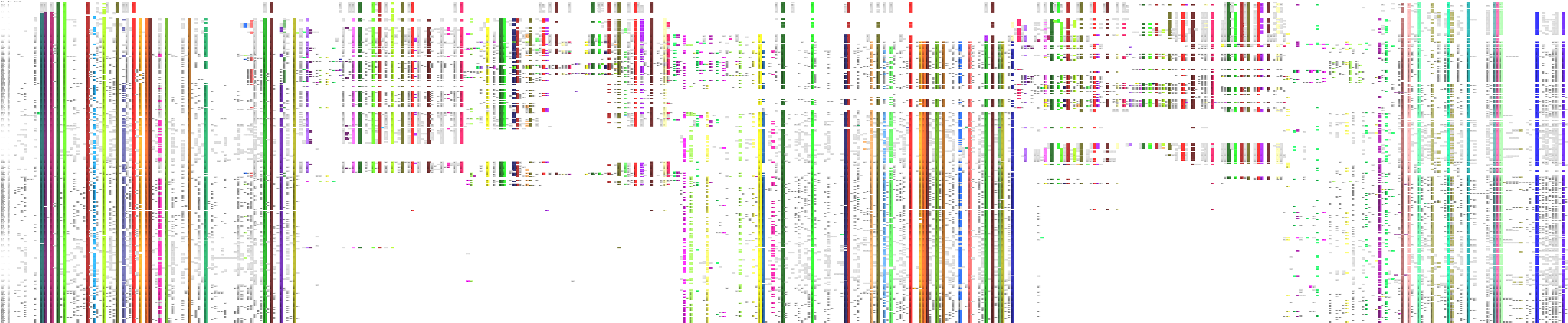

Supplement: Figure S7 — Homology block alignments. Homology block alignments for (panel A–E): DBL, CIDR, NTS, ATS, and whole PfEMP1, with details of Figure 6, Figure 8, Figure 10 and Figure 12. (0.82 MB ZIP) [file pcbi.1000933.s008.zip › Figure S7E - HB alignment - PfEMP1.pdf]

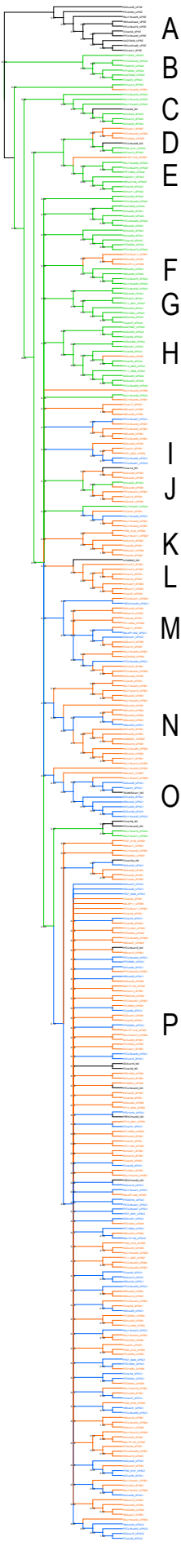

Supplement: Figure S8 — Tree in Figure 12 with labels. Bootstrap values are given as fractions of 1000 bootstraps. (0.33 MB PDF) [file pcbi.1000933.s009.pdf]
