# Supplementary material for: Plasmodium falciparum Erythrocyte Membrane Protein 1 Diversity in Seven Genomes – Divide and Conquer
Source: PLoS Comput Biol. 2010 Sep 16;6(9):e1000933. doi: 10.1371/journal.pcbi.1000933 (PMC2940729; doi:10.1371/journal.pcbi.1000933)
Supplement: Figure S2 — Trees showing subclassification of all major PfEMP1 domain classes. ML trees based on amino acid alignments of each of the following domain classes are shown in panels A–M: DBLα0, α1, β, δ, ε, γ, ζ; CIDRα, β, γ, δ; NTS; ATS. Sequence names as well as start and stop position of the domains are given in the trees, followed by classification of the domain. Panel N and O: Assignment of sequences to UPS groups by Markov clustering (N) and neighbor joining (O). The UPS groups were named as indicated by the text color. The background colors show the group membership assigned by Kraemer et al. 2007 [16]. Sequences found upstream of domain cassette 8 (Figure 3) are marked with black squares. (N) The branch labels show the fraction of Markov clusters with this group present. (O) The branch labels show the bootstrap values as fractions of 1000 bootstraps. Monophyletic subgroups with a bootstrap support above 0.7 and containing sequences from at least four different strains of P. falciparum are highlighted with thick red branches. Some subgroups were further expanded (without bootstrap support) to form larger monophyletic groups: UPSA2 and UPSB3 are expanded to include additional sequences annotated to UPSA2 and UPSB3 respectively by Kraemer et al. 2007 [16], UPSB2 is expanded to include two genes with same domain architecture, and UPSC1 is expanded to include three sequences that fall between UPSC1 and UPSC2 but within the larger monophyletic group comprising all UPSC sequences. The sequences are shown with thick black branches. The additional sequences included by this expansion are denoted with an asterisk in the annotation in Figure S4 and S5. UPSA3 and UPSB1 are groups that contain all the sequences not assigned to any other subgroup in UPSA and UPSB respectively. ND: Not Determined. (1.11 MB ZIP) [file pcbi.1000933.s003.zip › Figure S2O - Tree UPS.pdf]

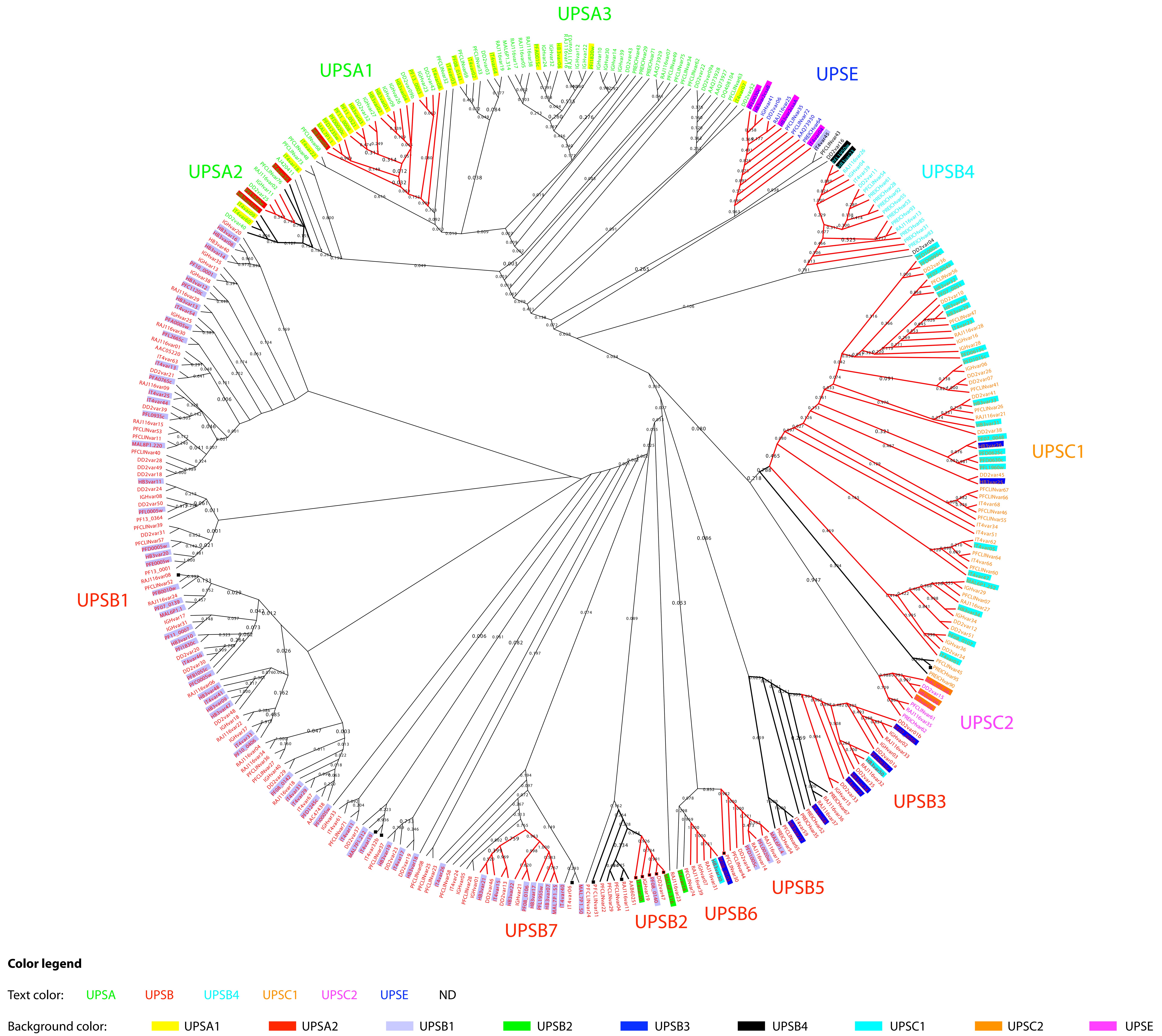
UPSA3
UPSA1
UPSE
UPSA2
UPSB4
UPSC1
UPSB1
UPSC2
UPSB3
UPSB5
UPSB6
UPSB2
UPSB7
Color legend
Text color: UPSA UPSB UPSB4 UPSC1 UPSC2 UPSE ND
Background color: UPSA1 UPSA2 UPSB1 UPSB2 UPSB3 UPSB4 UPSC1 UPSC2 UPSE
