# Supplementary material for: Plasmodium falciparum Erythrocyte Membrane Protein 1 Diversity in Seven Genomes – Divide and Conquer
Source: PLoS Comput Biol. 2010 Sep 16;6(9):e1000933. doi: 10.1371/journal.pcbi.1000933 (PMC2940729; doi:10.1371/journal.pcbi.1000933)
Supplement: Figure S5 — Schematic representation of annotated var genes sorted by genome origin. Gene names, 5′UTR class, domain architecture and origin of sequence data (if sequence is not previously reported as var gene) is given. Sequences are noted “F” (Fragment) in comments if predicted not to span a full length exon1, and “HBD” if incorrect contig assembly is suspected followed by the name of the sequence which partially contains unexpected identical sequence stretches. N-terminal segment (NTS), Duffy binding-like (DBL), Cys-rich inter-domain region (CIDR) and acidic terminal segment (ATS) are named according to the distance tree classification. (0.05 MB PDF) [file pcbi.1000933.s006.pdf]

Gene name	Parasite	UPS	NTS	Extracellular Domain Structure (Predicted)									ATS	Comments	Data base source if different
PF13_0003	3D7	A1	A3	DBLα1.6	CIDRδ1	DBLβ3	DBLγ12	DBLδ5	CIDRβ3	DBLβ9			A2		
PF11_0008	3D7	A1	A6	DBLα1.5	CIDRγ3	DBLγ12	DBLδ5	CIDRβ4	DBLβ9				A2		
PF11_0521	3D7	A1	A6	DBLα1.7	CIDRα1.4	DBLβ3	DBLβ6	DBLδ3	CIDRγ2				A2		
PFD1235w	3D7	A1	A2	DBLα1.4	CIDRα1.6	DBLβ3	DBLβ3	DBLγ13	DBLδ1	CIDRβ5			A2		
PFD0020c	3D7	A1	A7	DBLα1.2	CIDRα1.1	DBLβ12	DBLγ6	DBLγ11	DBLδ1	CIDRγ8			A1		
PFE1640w	3D7	A2	A1	DBLα1.4	CIDRα1.3	DBLβ1	DBLγ15	DBLε1	DBLγ8	DBLζ1	DBLε5				
PF08_0141	3D7	A3	A5	DBLα1.6	CIDRδ2	DBLβ6	DBLγ14	DBLζ5	DBLε4				A3		
PFA0015c	3D7	A3	A3	DBLα1.3	DBLε8								var3		
MAL6P1.314	3D7	A3	A3	DBLα1.3	DBLε8								var3		
PFI1820w	3D7	A3	A3	DBLα1.3	DBLε8								var3		
PF10_0001	3D7	B1	B3	DBLα0.20	CIDRα3.1	DBLδ1	CIDRγ4						B1		
PFI0005w	3D7	B1	B3	DBLα0.16	CIDRα3.4	DBLδ1	CIDRγ12						B6		
PFL0935c	3D7	B1	B3	DBLα0.16	CIDRα3.4	DBLδ1	CIDRγ12						B18		
PFI1830c	3D7	B1	B3	DBLα0.1	CIDRα3.1	DBLδ1	CIDRγ5						B1		
MAL7P1.50	3D7	B1	B3	DBLα0.1	CIDRα3.1	DBLδ1	CIDRγ10						B2		
PF08_0142	3D7	B1	B3	DBLα0.9	CIDRα2.11	DBLδ1	CIDRβ1						B1		
PFL0005w	3D7	B1	B3	DBLα0.9	CIDRα2.2	DBLδ1	CIDRβ1						B21		
PFA0765c	3D7	B1	B3	DBLα0.5	CIDRα2.8	DBLδ1	CIDRβ1						B1		
PFA0005w	3D7	B1	B3	DBLα0.11	CIDRα2.8	DBLδ1	CIDRβ1						B3		
PFL2665c	3D7	B1	B3	DBLα0.19	CIDRα2.3	DBLδ1	CIDRβ1						B2		
PF13_0001	3D7	B1	B3	DBLα0.11	CIDRα2.4	DBLδ1	CIDRβ1						B21		
PFE0005w	3D7	B1	B3	DBLα0.11	CIDRα2.4	DBLδ1	CIDRβ1						B1		
PFC0005w	3D7	B1	B3	DBLα0.9	CIDRα2.4	DBLδ1	CIDRβ1						B1		
PF10_0406	3D7	B1	B3	DBLα0.9	CIDRα2.7	DBLδ1	CIDRβ1						B1		
MAL7P1.212	3D7	B1	B3	DBLα0.17	CIDRα3.1	DBLδ1	CIDRβ1						B1		
MAL8P1.220	3D7	B1	B3	DBLα0.1	CIDRα3.1	DBLδ1	CIDRβ1						B1		
PFB1055c	3D7	B1	B3	DBLα0.16	CIDRα3.4	DBLδ1	CIDRβ1						B1		
PF13_0364	3D7	B1	B3	DBLα0.16	CIDRα3.4	DBLδ1	CIDRβ6						B1		
PF11_0007	3D7	B1	B3	DBLα0.15	CIDRα3.2	DBLδ1	CIDRβ1						B2		
MAL6P1.1	3D7	B1	B3	DBLα0.8	CIDRα4	DBLδ1	CIDRβ1						B2		
PFD1245c	3D7	B1	B3	DBLα0.14	CIDRα4	DBLδ1	CIDRβ1						B8		
PFC1120c	3D7	B1	B3	DBLα0.9	CIDRα2.1	DBLδ1	CIDRβ7						B1		
PF07_0139	3D7	B1	B3	DBLα0.16	CIDRα3.4	DBLδ1	CIDRβ1	DBLε4					B1		
PFB0010w	3D7	B1	B3	DBLα0.7	CIDRα2.2	DBLγ11							B1		
PFD0005w	3D7	B1	B3	DBLα0.5	CIDRα2.10	DBLγ5	DBLδ1	CIDRβ1					B1		
MAL6P1.316	3D7	B2	B3	DBLα2	CIDRα1.8	DBLβ12	DBLγ4	DBLζ3	DBLε12				B3		
PF08_0140	3D7	B2	B5	DBLα2	CIDRα1.6	DBLβ12	DBLγ4	DBLδ1	CIDRβ1				B1		
PF07_0050	3D7	B3	B3	DBLα0.18	CIDRα6	DBLβ6	DBLγ9						B6		
PFD0635c	3D7	B3	B3	DBLα0.1	CIDRα3.1	DBLδ1	CIDRγ2						B5		
PFL1950w	3D7	B4	B3	DBLα0.7	CIDRα4	DBLβ8	DBLδ1	CIDRβ1					B8		
MAL6P1.4	3D7	B5	B3	DBLα0.6	CIDRα3.2	DBLβ5	DBLγ13	DBLδ4	CIDRγ1	DBLε2	DBLε7	DBLε3	B16		
PFL0020w	3D7	B5	B3	DBLα0.18	CIDRα5	DBLβ5	DBLγ14	DBLζ5	DBLε4				B3		
PFD1005c	3D7	B5	B3	DBLα0.8	CIDRα4	DBLδ1	CIDRγ11						B19		
PF08_0103	3D7	B6	B3	DBLα0.12	CIDRα2.2	DBLδ1	CIDRβ1						B9		
MAL7P1.55	3D7	B7	B3	DBLα0.9	CIDRα2.4	DBLδ1	CIDRγ5						B13		
PF08_0106	3D7	B7	B3	DBLα0.2	CIDRα3.1	DBLδ1	CIDRβ1						B14		
PFL1955w	3D7	B7	B3	DBLα0.16	CIDRα3.4	DBLδ1	CIDRβ1						B7		
MAL6P1.252	3D7	C1	B1	DBLα0.21	CIDRα2.1	DBLβ4	DBLδ1	CIDRβ1					B17		
PFD0995c	3D7	C1	B3	DBLα0.1	CIDRα3.2	DBLδ1	CIDRγ6						B11		
PFD1000c	3D7	C1	B3	DBLα0.1	CIDRα3.2	DBLδ1	CIDRγ11						B19		
PF07_0049	3D7	C1	B3	DBLα0.17	CIDRα3.1	DBLδ1	CIDRγ7						B9		
PFD0630c	3D7	C1	B3	DBLα0.1	CIDRα3.1	DBLδ1	CIDRγ2						B5		
PF08_0107	3D7	C1	B3	DBLα0.15	CIDRα3.2	DBLδ1	CIDRγ2						B2		
PF07_0051	3D7	C1	B3	DBLα0.1	CIDRα3.1	DBLδ1	CIDRβ1						B12		
PFD0615c	3D7	C1	B3	DBLα0.17	CIDRα3.1	DBLδ1	CIDRβ1						B10		
PFL1960w	3D7	C1	B3	DBLα0.20	CIDRα3.1	DBLδ1	CIDRβ1						B23		
PFD1015c	3D7	C1	B3	DBLα0.24	CIDRα3.4	DBLδ1	CIDRβ1						B6		
PFD0625c	3D7	C1	B3	DBLα0.1	CIDRα3.2	DBLδ1	CIDRβ1						B5		
PF07_0048	3D7	C1	B3	DBLα0.1	CIDRα3.2	DBLδ1	CIDRβ6						B7		
MAL7P1.56	3D7	C2	B3	DBLα0.20	CIDRα3.1	DBLδ1	CIDRβ1						B1		
PFL0030c	3D7	E	pam	DBLpam1	DBLpam2	CIDRpam	DBLpam3	DBLεpam4	DBLεpam5	DBLε10			pam1		
DD2var32	DD2	A1	A6	DBLα1.7	CIDRα1.4	DBLβ1	DBLβ6	DBLγ11	DBLδ1	CIDRγ5			A2		supercontig 80
DD2var42	DD2	A1	A5	DBLα1.2	CIDRα1.7	DBLβ7	DBLγ2	DBLδ1	CIDRβ1				A2		supercontig 64
DD2var09b	DD2	A1	A6	DBLα1.5	CIDRδ2	DBLγ11	DBLβ7	DBLδ1	CIDRβ1				A2		supercontig 93
DD2var04	DD2	B1	A5	DBLα1.6	CIDRδ2	DBLβ6	DBLγ12	DBLδ5	CIDRβ4	DBLβ7	DBLγ9		A4		supercontig 37
DD2var25	DD2	A2	A1	DBLα1.1	CIDRα1.2	DBLβ11	DBLγ1	DBLε1	DBLγ8	DBLε7	DBLε3	DBLγ9	var1		supercontig 5
DD2var09a	DD2	A3	A8	DBLα1.2	CIDRα1.5	DBLγ17								F	DQ408011
DD2var40	DD2	A2*	A7	DBLα1.2	CIDRα1.5	DBLγ17	DBLδ1	CIDRγ1	DBLζ2	DBLε6			A3	HBD: DD2var43, DD2var09a	supercontig 13
DD2var43	DD2	A3	A3	DBLα1.2	CIDRα1.5	DBLγ17	DBLδ5	CIDRβ3	DBLβ7	DBLγ9			A4	HBD: DD2var40	supercontig 54
DD2var22	DD2	A3	A3	DBLα1.6	CIDRδ1	DBLγ12	DBLδ5	CIDRβ4	DBLβ9				A2		supercontig 14
DD2var52	DD2	A3	A5	DBLα1.5	CIDRδ1	DBLβ7	DBLγ11	DBLγ11					A4		supercontig 2
DD2var03	DD2	A3	A4	DBLα1.5	CIDRδ1	DBLβ6	DBLγ13	DBLδ1	CIDRβ4				A6		supercontig 35
DD2var49	DD2	B1	A8	DBLα1.2	CIDRα1.7	DBLβ3	DBLγ11	DBLδ3	CIDRγ2	DBLγ17	DBLζ4				supercontig 6
DQ408065	DD2			DBLε8										F	
DD2var23	DD2	B1	B3	DBLα0.9	CIDRα3.1	DBLδ1	CIDRγ5	DBLζ1	DBLε14				B20		supercontig 14
DD2var50	DD2	B1	B3	DBLα0.22	CIDRα3.1	DBLβ5	DBLγ13	DBLδ4	CIDRγ1	DBLζ6	DBLε6		B8		supercontig 5
DD2var24	DD2	B1	B3	DBLα0.15	CIDRα3.2	DBLβ5	DBLδ1	CIDRγ1	DBLγ3	DBLζ4			B22		supercontig 10
DD2var21	DD2	B1	B3	DBLα0.3	CIDRα5	DBLβ5	DBLδ9	CIDRγ9	DBLγ11	DBLζ4			B5		supercontig 2
DD2var48	DD2	B1	B3	DBLα0.6	CIDRα3.1	DBLβ5	DBLδ4	CIDRγ4	DBLγ11	DBLζ4			B16		supercontig 48
DD2var28	DD2	B1	B3	DBLα0.4	CIDRα6	DBLβ5	DBLγ18	DBLε8					B18		supercontig 9
DD2var30	DD2	B1	B3	DBLα0.6	CIDRα3.1	DBLβ5	DBLδ4	CIDRγ1					B3		supercontig 19
DD2var29	DD2	B1	B3	DBLα0.16	CIDRα3.4	DBLδ1	CIDRβ1	DBLγ10					B1		supercontig 218
DD2var19	DD2	B1	B3	DBLα0.10	CIDRα2.2	DBLδ1	CIDRγ9	DBLζ6	DBLε6				B15	HBD: DD2var18	supercontig 25
DD2var18	DD2	B1	B3	DBLα0.5	CIDRα2.2	DBLδ1	CIDRβ1						B1	HBD: DD2var19	supercontig 93
DD2var37	DD2	B1	B3	DBLα0.3	CIDRα3.2	DBLδ1	CIDRγ5						B1		supercontig 35
DD2var31	DD2	B1	B3	DBLα0.5	CIDRα2.6	DBLδ1	CIDRβ1						B2		supercontig 13
DD2var20	DD2	B1	B3	DBLα0.9	CIDRα2.7	DBLδ1	CIDRβ6						B2		supercontig 58
DD2var39	DD2	B1	B3	DBLα0.10	CIDRα2.8	DBLδ1	CIDRβ1						B18		supercontig 17
DD2var47	DD2	B2	B3	DBLα2	CIDRα1.1	DBLβ12	DBLγ6	DBLδ1	CIDRβ1				B16		supercontig 2
DD2var01a	DD2	B3	B3	DBLα0.6	CIDRα3.1	DBLβ5	DBLγ5	DBLδ1	CIDRβ1				B13		AAA75396
DD2var01b	DD2	B3	B3	DBLα0.6	CIDRα3.1	DBLβ5	DBLγ5	DBLδ1	CIDRβ1				B2	HBD: DD2var01a	supercontig 2
DD2var35	DD2	B3	B3	DBLα0.2	CIDRα3.1	DBLδ1	CIDRγ7						B2		supercontig 26
DD2var33	DD2	B3	B3	DBLα0.9	CIDRα2.1	DBLδ1	CIDRβ3						B10		supercontig 26
DD2var11	DD2	B4	B3	DBLα0.15	CIDRα3.2	DBLδ1	CIDRβ1						B15		supercontig 21
DD2var44	DD2	B5	B3	DBLα0.23	CIDRα3.1	DBLδ1	CIDRβ5						B4		supercontig 26
DD2var46	DD2	B7	B3	DBLα0.5	CIDRα2.5	DBLβ8	DBLδ1	CIDRβ7					B12		supercontig 21
DD2var13	DD2	B7	B3	DBLα0.8	CIDRα3.5	DBLβ8	DBLδ1	CIDRβ1					B13	HBD: DD2var01a	supercontig 2
DD2var07	DD2	C1	B3	DBLα0.16	CIDRα2.3	DBLδ1	CIDRβ5								AAA75399
DD2var26	DD2	C1	B3	DBLα0.19	CIDRα2.3	DBLδ1	CIDRβ5							HBD: DD2var07	supercontig 208
DD2var41	DD2	C1	B3	DBLα0.16	CIDRα3.4	DBLβ10	DBLδ1	CIDRβ1					B7	HBD: DD2var07	supercontig 187
DD2var34	DD2	C1	B3	DBLα0.1	CIDRα3.1	DBLγ16	DBLδ1	CIDRγ8					B13		supercontig 394
DD2var36	DD2	C1	B1	DBLα0.21	CIDRα2.1	DBLβ2	DBLγ10	DBLδ2	CIDRγ6				B11		supercontig 344
DD2var38	DD2	C1	B4	DBLα0.21	CIDRα2.1	DBLβ2	DBLγ10	DBLδ8	CIDRβ2				B17		supercontig 274
DD2var45	DD2	C1	B3	DBLα0.1	CIDRα3.1	DBLδ1	CIDRγ4						B7		supercontig 193
DD2var12	DD2	C1	B3	DBLα0.24	CIDRα3.3	DBLδ1	CIDRγ6						B12		supercontig 308
DD2var51	DD2	C1	B3	DBLα0.1	CIDRα3.2	DBLδ6	CIDRβ2						B17		supercontig 339
DD2var10	DD2	C1	B3	DBLα0.15	CIDRα3.2	DBLδ1	CIDRβ1						B13		supercontig 208
DD2var15	DD2	C2	B2	DBLα0.4	CIDRα3.1	DBLδ1	CIDRβ5						B6		supercontig 2
DD2var16	DD2		B7	DBLα0.19	CIDRα2.3	DBLδ1	CIDRβ5						B2	F	supercontig 113
DD2var06	DD2	E	pam	DBLpam1	DBLpam2	CIDRpam	DBLpam3	DBLεpam4	DBLεpam5	DBLε10			pam2		AY372121
IT4var08	IT4	A1	A6	DBLα1.7	CIDRγ3	DBLβ3	DBLδ4	CIDRγ1	DBLζ6	DBLε9			A1		
IT4var35	IT4	A2*	A1	DBLα1.1	CIDRα1.2	DBLβ11	DBLγ1	DBLε1	DBLγ8	DBLζ1	DBLε5				
IT4var03	IT4	A3	A6	DBLα1.3	DBLε8								var3		
IT4var02	IT4	A3	A6	DBLα1.5	CIDRδ1	DBLβ3	DBLγ12	DBLδ5	CIDRβ3	DBLβ9			A5		
IT4var07	IT4	A3	A6	DBLα1.7	CIDRα1.4	DBLβ1	DBLβ3	DBLγ10	DBLδ1	CIDRβ1			A4		
IT4var18	IT4	A3	A3	DBLα1.2	CIDRα1.6	DBLβ3	DBLγ16	DBLγ2	DBLδ1	CIDRβ1			A6		
IT4var64	IT4	A3	A5	DBLα1.5	CIDRδ1	DBLβ7	DBLγ11	DBLγ11					A4	HBD: DQ408104	
IT4var22	IT4	A3	A2	DBLα1.4	CIDRα1.7	DBLβ3	DBLγ10	DBLγ11	DBLδ1	CIDRβ1			A3	HBD: DQ408104	
DQ408104	IT4	A3	A5	DBLα1.5										F	
IT4var60	IT4		A7	DBLα1.8	CIDRβ2	DBLγ7	DBLζ5	DBLε11	DBLε12				A1		
IT4var9	IT4		A8	DBLα1.6	CIDRγ3	DBLγ15	DBLε1	DBLδ1	CIDRβ1						
IT4var14	IT4		B3	DBLα0.23	CIDRα5	DBLβ5	DBLδ1	CIDRγ2	DBLγ3	DBLζ4					
IT4var46	IT4	B1	B3	DBLα0.10	CIDRα2.2	DBLδ1	CIDRγ4	DBLε2	DBLζ3	DBLε3			B19		
IT4var10	IT4	B1	B3	DBLα0.5	CIDRα2.6	DBLβ10	DBLδ1							F	
IT4var67	IT4	B1	B3	DBLα0.10	CIDRα2.2	DBLε2	DBLζ3	DBLε12					B5		Supercontig_0000000
IT4var13	IT4	B1	B3	DBLα0.3	CIDRα5	DBLβ5	DBLδ9	CIDRγ9	DBLγ11	DBLζ4			B5		
IT4var19	IT4	B1	B3	DBLα2	CIDRα1.1	DBLβ12	DBLγ6	DBLδ1	CIDRβ1	DBLγ9			B3	HBD: ITvar32a/b	
IT4var32a	IT4		B3	DBLα0.23	CIDRα3.2	DBLγ6	DBLδ1	CIDRβ1					B16		AAD29126
IT4var32b	IT4	B1	B3	DBLα2	CIDRα1.1	DBLβ12	DBLγ6	DBLδ1	CIDRβ1				B15	HBD: ITvar32a, ITvar19	
IT4var06	IT4	B1	B3	DBLα2	CIDRα1.1	DBLβ12	DBLγ6	DBLδ1	CIDRβ4				B15		
IT4var41	IT4	B1	B3	DBLα0.4	CIDRα5	DBLβ5	DBLγ5	DBLδ1	CIDRβ1						
IT4var16	IT4	B1	B3	DBLα0.4	CIDRα5	DBLβ5	DBLγ16	DBLδ1	CIDRβ6				B18		
IT4var31	IT4	B1	B3	DBLα0.18	CIDRα4	DBLβ3	DBLγ9								
IT4var44	IT4	B1	B3	DBLα0.16	CIDRα3.4	DBLβ13	DBLδ1	CIDRβ6					B2		
IT4var11	IT4	B1	B3	DBLα0.3	CIDRα2.4	DBLβ10	DBLδ1	CIDRβ1					B1		
AAB06961	IT4	B1	B3	DBLα0.18	CIDRα6	DBLβ4	DBLδ1	CIDRβ1							
IT4var12	IT4		B3	DBLα0.18	CIDRα6	DBLβ4	DBLδ1	CIDRβ1					B2		AAC47438
IT4var17	IT4	B1	B3	DBLα0.11	CIDRα2.4	DBLβ8	DBLδ1	CIDRγ4	DBLγ10				B18		
IT4var29	IT4	B1	B3	DBLα0.3	CIDRα3.2	DBLδ1	CIDRγ5						B20		
IT4var24	IT4	B1	B3	DBLα0.10	CIDRα2.2	DBLδ1	CIDRγ5						B1		
IT4var45	IT4	B1	B3	DBLα0.5	CIDRα2.9	DBLδ1	CIDRβ1						B1		
IT4var33	IT4	B1	B3	DBLα0.11	CIDRα2.4	DBLδ1	CIDRβ5						B15		
IT4var25	IT4	B1	B3	DBLα0.11	CIDRα2.4	DBLδ1	CIDRβ1						B8		
IT4var61	IT4	B1	B3	DBLα0.9	CIDRα2.7	DBLδ1	CIDRβ1						B6		Supercontig_0002013
IT4var54	IT4	B1	B3	DBLα0.1	CIDRα3.1	DBLδ1	CIDRβ1						B1		
IT4var63	IT4	B1	B3	DBLα0.7	CIDRα3.4	DBLδ1	CIDRβ1						B5		Supercontig_0002160
IT4var26	IT4	B1	B3	DBLα0.23	CIDRα3.3	DBLδ1	CIDRβ1						B1		
IT4var40	IT4	B1	B3	DBLα0.12	CIDRα2.11										
IT4var20	IT4	B2	B3	DBLα2	CIDRα1.1	DBLβ12	DBLγ6	DBLδ1	CIDRβ1				B16		
IT4var27	IT4	B3	B3	DBLα0.6	CIDRα3.1	DBLβ5	DBLγ5	DBLδ1	CIDRβ1				B2		
IT4var58	IT4	B3*	B3	DBLα0.1	CIDRα3.1	DBLδ1	CIDRβ5							HBD: IT4var21	
IT4var59	IT4	B3*	B3	DBLα0.1	CIDRα3.1	DBLδ1	CIDRβ1						B14	HBD: IT4var21	Supercontig_0000211
IT4var21	IT4		B3	DBLα0.1	CIDRα3.1	DBLδ1	CIDRβ1						B14		AAC05730
IT4var15	IT4	B7	B3	DBLα0.8	CIDRα3.5	DBLβ8	DBLδ1	CIDRβ1					B13		
IT4var01	IT4	C1	B6	DBLα0.18	CIDRα6	DBLβ5	DBLγ10	DBLδ2	CIDRγ6						
IT4var51	IT4	C1	B3	DBLα0.17	CIDRα3.1	DBLδ1	CIDRγ12						B5		
IT4var66	IT4	C1	B3	DBLα0.19	CIDRα2.10	DBLδ1	CIDRβ1								Supercontig_0002173
IT4var23	IT4	C1	B3	DBLα0.1	CIDRα3.1	DBLδ1	CIDRγ10						B10		
IT4var05	IT4	C1	B3	DBLα0.5	CIDRα2.3	DBLδ1	CIDRβ1						B1		
IT4var34	IT4	C1	B3	DBLα0.1	CIDRα3.1	DBLδ1	CIDRβ4						B12		
IT4var47	IT4	C1	B3	DBLα0.1	CIDRα3.3	DBLδ1	CIDRβ1						B4		
IT4var62	IT4	C1	B3	DBLα0.1	CIDRα3.1	DBLδ8	CIDRβ2						B11		Supercontig_0000093
IT4var68	IT4	C1	B3	DBLα0.1	CIDRα3.2									F	Supercontig_0000093
IT4var28	IT4	C2	B2	DBLα0.4	CIDRα3.1	DBLδ1	CIDRβ5						B6		
IT4var30	IT4			DBLα0.13	CIDRα2.10	DBLδ1	CIDRβ1						B5	F	
IT4var36	IT4			DBLα0.8	CIDRα5	DBLδ1	CIDRβ1						B6	F	
IT4var39	IT4		B3	DBLα0.5	CIDRα2.5	DBLδ1	CIDRβ6						B9		
IT4var04	IT4	E	pam	DBLpam1	DBLpam2	CIDRpam	DBLpam3	DBLεpam4	DBLεpam5	DBLε10			pam1		
IT4var65	IT4			CIDRβ1									B10	F	Supercontig_0002047
HB3var04	HB3	A1	A7	DBLα1.5	CIDRδ1	DBLγ11	DBLδ1	CIDRβ1	DBLγ3	DBLζ3	DBLε9		A3		
HB3var02	HB3	A1	A2	DBLα1.4	CIDRα1.6	DBLγ12	DBLδ5	CIDRβ3	DBLβ3				A2		
HB3var03	HB3	A1	A6	DBLα1.7	CIDRα1.4	DBLβ3	DBLγ12	DBLδ5	CIDRβ3	DBLβ7				HBD: HB3var05	
HB3var05	HB3	A1	A3	DBLα1.6	CIDRδ1	DBLγ12	DBLδ5	CIDRβ3	DBLβ7				A2	HBD: HB3var03	
HB3var01	HB3	A1	A5	DBLα1.6	CIDRδ2	DBLβ7	DBLγ13	DBLγ9	DBLδ1	CIDRβ1			A2		
HB3var1csa	HB3	A2*	A1	DBLα1.1	CIDRα1.2	DBLβ11	DBLγ1	DBLε1	DBLγ8	DBLζ1	DBLε5		var1		
HB3var06	HB3	A3	A2	DBLα1.5	CIDRδ1	DBLγ6	DBLδ2	CIDRγ6	DBLε13	DBLζ2	DBLε14		A1		
HB3var52	HB3			DBLα1.6	CIDRδ2									F	Supercontig 580
HB3var40	HB3	B1	B3	DBLα0.7	CIDRα3.4	DBLδ1	CIDRγ1	DBLζ6	DBLε9				B1	HBD: HB3var50, HB3var14	
HB3var14	HB3	B1	B3	DBLα0.7	CIDRα3.4	DBLδ1	CIDRβ1						B1	HBD: HB3var40	
HB3var09	HB3	B1	B3	DBLα0.11	CIDRα2.3	DBLδ1	CIDRγ4	DBLζ6	DBLε6				B1	HBD: HB3var47	
HB3var47	HB3	B1	B3	DBLα0.11	CIDRα2.3	DBLδ1	CIDRβ1						B1	HBD: HB3var09	
HB3var08	HB3	B1	B3	DBLα0.9	CIDRα2.7	DBLδ1	CIDRγ7	DBLγ14	DBLζ5	DBLε4			B3	HBD: HB3var07	
HB3var48	HB3	B1	B3	DBLα0.4	CIDRα6	DBLβ5	DBLγ16	DBLζ4					B8		
HB3var11	HB3	B1	B3	DBLα0.12	CIDRα2.1	DBLδ1	CIDRβ3	DBLε4					B1		
HB3var10	HB3	B1	B3	DBLα0.6	CIDRα3.1	DBLβ5	DBLδ1	CIDRβ6					B1		
HB3var19	HB3	B1	B3	DBLα0.12	CIDRα2.2	DBLδ1	CIDRγ5						B18		
HB3var16	HB3	B1	B3	DBLα0.9	CIDRα2.2	DBLδ1	CIDRβ1						B21		
HB3var12	HB3	B1	B3	DBLα0.7	CIDRα2.2	DBLδ1	CIDRβ1						B8		
HB3var20	HB3	B1	B3	DBLα0.5	CIDRα2.5	DBLδ1	CIDRβ1						B1		
HB3var13	HB3	B1	B3	DBLα0.15	CIDRα3.2	DBLδ1	CIDRβ1								
HB3var18	HB3	B1	B3	DBLα0.15	CIDRα3.2	DBLδ1	CIDRβ1						B1		
HB3var24	HB3	B3	B3	DBLα0.1	CIDRα3.1	DBLδ1	CIDRγ8	DBLζ6	DBLε9				B12		
HB3var50	HB3	B3	B3	DBLα0.1	CIDRα3.2	DBLδ1	CIDRβ6	DBLζ6	DBLε9				B8	HBD: HB3var40	
HB3var27	HB3	B3*	B3	DBLα0.1	CIDRα3.1	DBLδ1	CIDRβ1								
HB3var23	HB3	B4	B3	DBLα0.7	CIDRα3.1	DBLδ1	CIDRγ9	DBLζ6	DBLε9				B9		
HB3var30	HB3	B6	B3	DBLα0.1	CIDRα3.2	DBLδ1	CIDRβ1						B7		
HB3var22	HB3	B7	B3	DBLα0.8	CIDRα2.2	DBLδ3	CIDRγ2	DBLε2	DBLε7	DBLε3			B9		
HB3var07	HB3	B7	B3	DBLα0.9	CIDRα2.4	DBLδ1	CIDRγ7	DBLγ14	DBLζ5	DBLε4				HBD: HB3var08	
HB3var21	HB3	B7	B4	DBLα0.4	CIDRα5	DBLβ5	DBLγ13	DBLδ4	CIDRδ1	DBLε4			B7		
HB3var17	HB3	B7	B4	DBLα0.6	CIDRα3.1	DBLβ5	DBLγ5	DBLδ1	CIDRβ1				B9		
HB3var25	HB3	C1	B3	DBLα0.23	CIDRα3.1	DBLβ4	CIDRγ1	DBLζ6	DBLε9				B11		
HB3var34	HB3	C1	B3	DBLα0.4	CIDRα6	DBLβ5	DBLγ10	DBLδ7	CIDRβ4				B17		
HB3var33	HB3	C1	B3	DBLα0.22	CIDRα3.1	DBLδ1	CIDRγ6						B12		
HB3var32	HB3	C1	B3	DBLα0.15	CIDRα3.1	DBLδ1	CIDRγ1						B11		
HB3var28	HB3	C1	B3	DBLα0.19	CIDRα2.8	DBLδ1	CIDRβ1						B4		
HB3var29	HB3	C1	B3	DBLα0.2	CIDRα3.2	DBLδ1	CIDRβ1						B10		
HB3var31	HB3	C1	B3	DBLα0.1	CIDRα3.2	DBLδ1	CIDRβ5						B19		
HB3var26	HB3	C1	B3	DBLα0.8	CIDRα3.5	DBLδ1	CIDRβ1						B8		
HB3var36	HB3	C2	B2	DBLα0.4	CIDRα3.2	DBLδ1	CIDRβ6						B1		
HB3var35/51	HB3		B3	DBLα0.1	CIDRα3.2	DBLδ1	CIDRγ4						B1		
HB3var2csaB	HB3	E	pam	DBLpam1	DBLpam2	CIDRpam	DBLpam3	DBLεpam4	DBLεpam5	DBLε10			B14		
HB3var2csaA	HB3	E	pam	DBLpam1	DBLpam2	CIDRpam	DBLpam3	DBLεpam4	DBLεpam5	DBLε10			B14		
HB3var39	HB3		B3	DBLα0.12										F	
IGHvar27	IGH	A1	A6	DBLα1.7	CIDRα1.4	DBLβ3	DBLβ6	DBLδ1	CIDRβ1				A2		supercontig_1.80
IGHvar26	IGH	A1	A7	DBLα1.1	CIDRα1.7	DBLβ3	DBLγ6	DBLγ11	DBLδ7	CIDRβ1			A4		supercontig_1.79
IGHvar23	IGH	A1	A5	DBLα1.7	CIDRα1.4	DBLβ1	DBLγ11	DBLγ2	DBLδ1	CIDRβ1			A4		supercontig_1.56
IGHvar09	IGH	A1	A3	DBLα1.2	CIDRα1.5	DBLβ6	DBLγ2	DBLγ4	DBLδ1	CIDRβ5			A5		supercontig_1.17
IGHvar11	IGH	A2	A1	DBLα1.1	CIDRα1.2	DBLβ11	DBLγ1	DBLε1	DBLγ8	DBLζ1	DBLε5		var1		supercontig_1.20
IGHvar10	IGH	A3	A2	DBLα1.4	CIDRγ3	DBLβ3	DBLγ9	DBLζ2	DBLε4				A3		supercontig_1.17
IGHvar22	IGH	A3	A8	DBLα1.8	CIDRβ2	DBLγ7	DBLε11	DBLζ2	DBLε6				A1		supercontig_1.52
IGHvar12	IGH	A3	A8	DBLα1.8	CIDRβ2	DBLγ7	DBLε11	DBLζ2	DBLε6				A1		supercontig_1.20
IGHvar24	IGH	A3	A8	DBLα1.8	CIDRβ2	DBLγ7	DBLε11	DBLζ3	DBLε6				var3		supercontig_1.58
IGHvar14	IGH	A3	A2	DBLα1.1	CIDRα1.6	DBLβ3	DBLγ18	DBLε7	DBLε3				var3		supercontig_1.21
IGHvar30	IGH	A3	A2	DBLα1.2	CIDRα1.5	DBLγ17	DBLδ5	CIDRβ4	DBLβ6	DBLε13	DBLζ4		A1		supercontig_1.87
IGHvar39	IGH	A3	A7	DBLα1.1	CIDRα1.7	DBLβ3	DBLβ3	DBLγ6	CIDRβ5				A3		supercontig_1.135
IGHvar32	IGH	A3	A3	DBLα1.2	CIDRα1.4	DBLβ3	DBLγ1	DBLε1	DBLδ7	CIDRβ5			A2		supercontig_1.101
IGHvar35	IGH	B1	B3	DBLα0.11	CIDRα2.9	DBLδ1	CIDRγ9	DBLζ6	DBLε9				B1		supercontig_1.322
IGHvar17	IGH	B1	B3	DBLα0.5	CIDRα2.3	DBLδ1	CIDRγ8	DBLζ6	DBLε9				B1		supercontig_1.33
IGHvar05	IGH	B1	B3	DBLα0.3	CIDRα3.4	DBLγ10	DBLδ1	CIDRγ5					B9		supercontig_1.13
IGHvar37	IGH	B1	B3	DBLα0.8	CIDRα5	DBLβ5	DBLγ5	DBLδ1	CIDRγ11						supercontig_1.540
IGHvar33	IGH	B1	B3	DBLα0.5	CIDRα2.5	DBLβ13	DBLδ1	CIDRβ7					B1		supercontig_1.102
IGHvar18	IGH	B1	B3	DBLα0.22	CIDRα3.1	DBLδ1	CIDRγ4						B20		supercontig_1.39
IGHvar13	IGH	B1	B3	DBLα0.14	CIDRα3.3	DBLδ1	CIDRγ8						B2		supercontig_1.21
IGHvar31	IGH	B1	B3	DBLα0.1	CIDRα3.2	DBLδ1	CIDRγ5						B6		supercontig_1.99
IGHvar20	IGH	B1	B2	DBLα0.4	CIDRα3.1	DBLδ1	CIDRγ11						B6		supercontig_1.47
IGHvar25	IGH	B1	B3	DBLα0.9	CIDRα2.2	DBLδ1	CIDRγ7						B1		supercontig_1.79
IGHvar38	IGH	B1	B3	DBLα0.13	CIDRα3.3	DBLδ1	CIDRβ1						B3		supercontig_1.128
IGHvar08	IGH	B1	B3	DBLα0.14	CIDRα4	DBLδ1	CIDRβ6						B6		supercontig_1.16
IGHvar40	IGH	B1	B3	DBLα0.16	CIDRα3.4	DBLδ1	CIDRβ1						B6		supercontig_1.166
IGHvar19	IGH	B2	B3	DBLα2	CIDRα1.1	DBLβ1	DBLγ6	DBLδ1	CIDRβ1				B20		supercontig_1.39
IGHvar02	IGH	B3	B3	DBLα0.18	CIDRα3.1	DBLγ5	DBLδ1	CIDRγ4					B23		supercontig_1.2
IGHvar15	IGH	B3	B3	DBLα0.8	CIDRα5	DBLβ5	DBLγ5	DBLδ1	CIDRβ5				B9		supercontig_1.27
IGHvar03	IGH	B3	B3	DBLα0.5	CIDRα2.6	DBLδ1	CIDRβ1						B4		supercontig_1.6
IGHvar04	IGH	B4	B3	DBLα0.1	CIDRα3.1	DBLδ1	CIDRβ1						B21		supercontig_1.13
IGHvar07	IGH	B6	B3	DBLα0.5	CIDRα2.3	DBLβ8	DBLγ5	DBLδ1	CIDRβ1				B4		supercontig_1.13
IGHvar21	IGH	B7	B3	DBLα0.12	CIDRα2.1	DBLβ12	DBLγ16	DBLζ5	DBLε11	DBLε3			B22		supercontig_1.47
IGHvar01	IGH	B7	B3	DBLα0.15	CIDRα3.2	DBLδ1	CIDRβ1						B7		supercontig_1.2
IGHvar34	IGH	C1	B3	DBLα0.18	CIDRα5	DBLβ5	DBLγ10	DBLδ6	CIDRβ2				B17		supercontig_1.320
IGHvar28	IGH	C1	B3	DBLα0.4	CIDRα6	DBLβ5	DBLγ2	DBLδ8	CIDRβ2				B11		supercontig_1.83
IGHvar36	IGH	C1	B3	DBLα0.22	CIDRα3.1	DBLδ1	CIDRγ7						B4		supercontig_1.468
IGHvar29	IGH	C1	B3	DBLα0.5	CIDRα2.6	DBLδ1	CIDRβ4						B4		supercontig_1.83
IGHvar16	IGH	C1	B3	DBLα0.11	CIDRα2.4	DBLδ1	CIDRβ6						B10		supercontig_1.27
IGHvar06	IGH	C1	B3	DBLα0.24	CIDRα3.1	DBLδ1	CIDRβ6						B13		supercontig_1.13
IGHvar41	IGH	E	pam	DBLpam1	DBLpam2	CIDRpam	DBLpam3	DBLεpam4	DBLεpam5	DBLε10			pam1		supercontig_1.226
PFCLINvar76	PFCLIN	A2*	A1	DBLα1.4	CIDRα1.3	DBLβ1	DBLγ15	DBLε1	DBLγ8	DBLζ1	DBLε5		var1		CONTIG21388
PFCLINvar34	PFCLIN	A3	A3	DBLα1.3	DBLε8								A1		CONTIG19469
PFCLINvar33	PFCLIN	A3	A6	DBLα1.7	CIDRγ3	DBLβ6	DBLγ18	DBLζ3	DBLε6						CONTIG21506
PFCLINvar75	PFCLIN	A3	A5	DBLα1.5	CIDRβ4	DBLγ7	DBLδ6	CIDRβ4	DBLζ3	DBLε6			A1		CONTIG20978
PFCLINvar62	PFCLIN	A3	A7	DBLα1.2	CIDRα1.6	DBLβ3	DBLε13							F	CONTIG20746
PFCLINvar69	PFCLIN	A3	A5	DBLα1.6	CIDRδ2	DBLγ12	DBLδ5	CIDRβ3	DBLβ7						CONTIG21376
PFCLINvar48	PFCLIN	A3	A2	DBLα1.4	CIDRα1.7	DBLβ1	DBLβ6							F	CONTIG20238
PFCLINvar73	PFCLIN	A3	A2	DBLα1.4	CIDRα1.7	DBLβ3	DBLγ2	DBLγ2	DBLδ1	CIDRγ4			A1		CONTIG21509
PFCLINvar49	PFCLIN	A3	A7	DBLα1.1	CIDRα1.4	DBLβ12	DBLγ16	DBLγ2							CONTIG20819
PFCLINvar32	PFCLIN	A3	A3	DBLα1.2	CIDRα1.4	DBLβ7	DBLγ11	DBLδ1	CIDRβ1				A3		CONTIG21334
PFCLINvar68	PFCLIN	A3	A7	DBLα1.1	CIDRα1.7	DBLβ3	DBLγ16	DBLδ5	CIDRβ4						CONTIG21384
PFCLINvar05	PFCLIN		A4	DBLα1.5	CIDRδ1	DBLβ7	DBLγ2	DBLγ4						F	CONTIG21033
PFCLINvar71	PFCLIN	B1	B3	DBLα0.3	CIDRα3.2	DBLγ10	DBLδ3	CIDRγ2	DBLε2	DBLζ3	DBLε3		B3		CONTIG21248
PFCLINvar52	PFCLIN	B1	B3	DBLα0.18	CIDRα4	DBLβ5	DBLζ6	DBLε12					B3		CONTIG21465
PFCLINvar40	PFCLIN	B1	B3	DBLα0.9	CIDRα2.7	DBLγ11	DBLζ3	DBLε12					B22		CONTIG21338
PFCLINvar74	PFCLIN	B1	B3	DBLα0.3	CIDRα5	DBLβ2	DBLδ9	CIDRγ2	DBLγ13	DBLζ4			B1		CONTIG21263
PFCLINvar58	PFCLIN	B1	B3	DBLα0.21	CIDRα2.1	DBLβ2	DBLγ3	DBLζ4							CONTIG21510
PFCLINvar39	PFCLIN	B1	B3	DBLα0.1	CIDRα3.2	DBLγ5	DBLδ1							F	CONTIG20335
PFCLINvar28	PFCLIN	B1	B7	DBLα0.21	CIDRα2.6	DBLδ1	CIDRγ12						B2		CONTIG20476
PFCLINvar27	PFCLIN	B1	B3	DBLα0.10	CIDRα2.8	DBLδ1	CIDRβ1							F	CONTIG19858
PFCLINvar23	PFCLIN	B1	B3	DBLα0.20	CIDRα3.2	DBLδ1	CIDRβ1								CONTIG20006
PFCLINvar53	PFCLIN	B1	B4	DBLα0.6	CIDRα3.1	DBLδ1	CIDRβ1								CONTIG21161
PFCLINvar24	PFCLIN	B1	B3	DBLα0.17	CIDRα3.1	DBLδ1	CIDRβ1								CONTIG21469
PFCLINvar37	PFCLIN	B1	B3	DBLα0.17	CIDRα3.1	DBLδ1	CIDRβ1						B1		CONTIG21465
PFCLINvar36	PFCLIN	B1	B3	DBLα0.3	CIDRα3.2	DBLδ1	CIDRβ1								CONTIG20612
PFCLINvar11	PFCLIN	B1	B3	DBLα0.14	CIDRα4	DBLδ1	CIDRβ1								CONTIG19349
PFCLINvar25	PFCLIN	B1	B3	DBLα0.13	CIDRα2.9	DBLδ1								F	CONTIG18287
PFCLINvar08	PFCLIN	B1	B3	DBLα0.15	CIDRα3.2									F	CONTIG18742
PFCLINvar57	PFCLIN	B1	B3	DBLα0.8	DBLδ1	CIDRγ7							B2		CONTIG21285
PFCLINvar31	PFCLIN	B2*	B5	DBLα2	CIDRα1.8	DBLβ4	DBLδ4	CIDRδ1					B20		CONTIG20898
PFCLINvar29	PFCLIN	B2*	B3	DBLα0.9	CIDRα2.4									F	CONTIG17861
PFCLINvar22	PFCLIN	B2*	B3	DBLα0.1	CIDRα3.2	DBLδ1								F	CONTIG19974
PFCLINvar04	PFCLIN	B2*	B3	DBLα0.1	CIDRα3.1									F	CONTIG17415
PFCLINvar65	PFCLIN	B3*	B3	DBLα0.1	CIDRα3.1	DBLδ1	CIDRβ1						B7		CONTIG20871
PFCLINvar54	PFCLIN	B4	B3	DBLα0.15	CIDRα3.2	DBLδ1	CIDRβ1						B15		CONTIG20750
PFCLINvar30	PFCLIN	B5	B1	DBLα2	CIDRα1.1	DBLβ3	DBLγ4	DBLζ5	DBLε4						CONTIG20118
PFCLINvar44	PFCLIN	B5	B3	DBLα0.5	CIDRα2.5	DBLβ13	DBLδ1	CIDRγ4					B5		CONTIG21283
PFCLINvar67	PFCLIN	C1	B3	DBLα0.9	CIDRα2.7	DBLγ14	DBLζ5	DBLε4					B8		CONTIG21202
PFCLINvar60	PFCLIN	C1	B1	DBLα0.21	CIDRα2.1	DBLβ2	DBLγ2						B12		CONTIG21333
PFCLINvar66	PFCLIN	C1	B3	DBLα0.5	CIDRα2.10	DBLβ13	DBLδ1	CIDRγ11					B7		CONTIG21267
PFCLINvar64	PFCLIN	C1	B3	DBLα0.19	CIDRα3.4	DBLδ1	CIDRγ6						B11		CONTIG21589
PFCLINvar26	PFCLIN	C1	B3	DBLα0.16	CIDRα3.4	DBLδ1	CIDRγ5						B4		CONTIG21429
PFCLINvar55	PFCLIN	C1	B3	DBLα0.5	CIDRα2.6	DBLδ1	CIDRβ1						B1		CONTIG21423
PFCLINvar46	PFCLIN	C1	B3	DBLα0.1	CIDRα3.1	DBLδ1	CIDRβ6								CONTIG20934
PFCLINvar47	PFCLIN	C1	B3	DBLα0.17	CIDRα3.1	DBLδ1	CIDRβ1								CONTIG21064
PFCLINvar41	PFCLIN	C1	B3	DBLα0.17	CIDRα3.1	DBLδ1	CIDRβ1								CONTIG20797
PFCLINvar56	PFCLIN	C1	B3	DBLα0.1	CIDRα3.1	DBLδ1	CIDRβ6						B23		CONTIG20957
PFCLINvar07	PFCLIN	C1	B3	DBLα0.20	CIDRα3.3									F	CONTIG21336
PFCLINvar61	PFCLIN	C2	B6	DBLα0.18	CIDRα2.1	DBLβ2	DBLγ10						B17		CONTIG21561
PFCLINvar63	PFCLIN	C2	B2	DBLα0.4	CIDRα3.1	DBLδ1	CIDRβ1						B9		CONTIG21273
PFCLINvar45	PFCLIN		B4	DBLα0.12	CIDRα2.1	DBLγ5	DBLγ17	DBLδ1	CIDRβ1						CONTIG20681
PFCLINvar15	PFCLIN		B3	DBLα0.8	CIDRα2.2	DBLδ1	CIDRγ8						B4		CONTIG21506
PFCLINvar43	PFCLIN		B3	DBLα0.15	CIDRα3.2	DBLδ1	CIDRγ10						B2		CONTIG21253
PFCLINvar09	PFCLIN		B3	DBLα0.3	CIDRα3.1										CONTIG17877
PFCLINvar06	PFCLIN		B3	DBLα0.18	CIDRα6	DBLβ5	DBLγ13	DBLζ2	DBLε4				B1		CONTIG20792
PFCLINvar72	PFCLIN	E	pam	DBLpam1	DBLpam2	CIDRpam	DBLpam3	DBLεpam4	DBLεpam5	DBLε10			pam2	F	CONTIG21495
PFCLINvar35	PFCLIN	E	pam	DBLpam1	DBLpam2	CIDRpam	DBLpam3	DBLεpam4	DBLεpam5	DBLε10					CONTIG20775
PFCLINvar59	PFCLIN			DBLβ5	DBLβ8	DBLδ1	CIDRβ2						B1	F	CONTIG21033
PFCLINvar17	PFCLIN			DBLβ6	DBLγ6									F	CONTIG20508
PFCLINvar77	PFCLIN			DBLδ1	CIDRβ1									F	CONTIG19170
PFCLINvar21	PFCLIN			DBLδ1	CIDRβ1								B15	F	CONTIG18706
PFCLINvar13	PFCLIN			DBLδ1	CIDRβ1									F	CONTIG19676
PFCLINvar12	PFCLIN			DBLδ1	CIDRβ1									F	CONTIG17888
RAJ116var02	RAJ116	A2	A1	DBLα1.4	CIDRα1.3	DBLβ1	DBLγ15	DBLε1	DBLγ8	DBLζ2	DBLε5		var1		supercontig_1.2
RAJ116var16	RAJ116	A3	A8	DBLα1.6	CIDRγ3	DBLγ15	DBLγ18	DBLζ3	DBLε6				A1		supercontig_1.10
RAJ116var05	RAJ116	A3	A3	DBLα1.8	CIDRδ2	DBLγ11	DBLδ6	CIDRβ4	DBLζ3	DBLε6			A6		supercontig_1.3
RAJ116var07	RAJ116	A3	A4	DBLα1.5	CIDRγ3	DBLγ14	DBLζ3	DBLε12					A1		supercontig_1.5
RAJ116var38	RAJ116	A3	A6	DBLα1.8	CIDRγ3	DBLγ4	DBLζ4						A3		supercontig_1.256
RAJ116var03	RAJ116	A3	A8	DBLα1.2	CIDRα1.5	DBLγ7	DBLε11	DBLα1.3	DBLε8				var3		supercontig_1.2
RAJ116var17	RAJ116	A3	A3	DBLα1.6	CIDRδ2	DBLγ11	DBLδ7	CIDRβ1					A1		supercontig_1.12
RAJ116var19	RAJ116	A3	A7	DBLα1.6	CIDRδ1	DBLγ2	DBLδ1	CIDRβ1					A5		supercontig_1.20
RAJ116var23	RAJ116	B1	B3	DBLα0.13	CIDRα2.5	DBLδ1	CIDRβ1	DBLε2	DBLζ3	DBLε3			A3		supercontig_1.54
RAJ116var22	RAJ116	B1	B3	DBLα0.12	CIDRα2.11	DBLε2	DBLε7	DBLε3					B1		supercontig_1.43
RAJ116var04	RAJ116	B1	B3	DBLα0.12	CIDRα2.1	DBLε2	DBLε7	DBLε3					B1		supercontig_1.2
RAJ116var15	RAJ116	B1	B3	DBLα0.9	CIDRα2.3	DBLδ1	CIDRγ2	DBLε2	DBLε7	DBLε3			B16		supercontig_1.9
RAJ116var29	RAJ116	B1	B3	DBLα0.9	CIDRα2.4	DBLδ1	CIDRγ1	DBLε2	DBLε7	DBLε3			B1		supercontig_1.119
RAJ116var08	RAJ116	B1	B3	DBLα2	CIDRα1.1	DBLβ12	DBLγ4	DBLε14	DBLε3				B3		supercontig_1.6
RAJ116var06	RAJ116	B1	B3	DBLα0.9	CIDRα2.7	DBLδ1	CIDRγ1	DBLγ3	DBLζ4				B16		supercontig_1.3
RAJ116var30	RAJ116	B1	B3	DBLα0.13	CIDRα2.6	DBLδ1	CIDRβ4	DBLε2					B22		supercontig_1.170
RAJ116var09	RAJ116	B1	B3	DBLα0.5	CIDRα2.9	DBLβ10	DBLδ1	CIDRβ1					B2		supercontig_1.7
RAJ116var34	RAJ116	B1	B3	DBLα0.15	CIDRα3.2	DBLδ1	CIDRγ10						B2		supercontig_1.190
RAJ116var18	RAJ116	B1	B3	DBLα0.6	CIDRα3.1	DBLδ1	CIDRγ2						B3		supercontig_1.15
RAJ116var01	RAJ116	B1	B3	DBLα0.13	CIDRα2.5	DBLδ1	CIDRβ1						B2		supercontig_1.1
RAJ116var24	RAJ116	B1	B3	DBLα0.9	CIDRα2.3	DBLδ1	CIDRβ1						B3		supercontig_1.62
RAJ116var11	RAJ116	B2*	B5	DBLα2	CIDRα1.8	DBLβ12	DBLγ4	DBLδ1	CIDRβ1				B8		supercontig_1.8
RAJ116var33	RAJ116	B3	B6	DBLα0.18	CIDRα6	DBLβ4	DBLγ13	DBLδ1	CIDRβ1				B4		supercontig_1.186
RAJ116var32	RAJ116	B3	B3	DBLα0.3	CIDRα3.1	DBLδ1	CIDRβ1						B10		supercontig_1.182
RAJ116var37	RAJ116	B3*	B7	DBLα0.12	CIDRα2.11	DBLβ10	DBLδ2	CIDRγ6	DBLγ2				B12		supercontig_1.245
RAJ116var36	RAJ116	B3*	B3	DBLα0.13	CIDRα2.9	DBLδ1	CIDRβ3						B11		supercontig_1.241
RAJ116var13	RAJ116	B4	B3	DBLα0.1	CIDRα3.3	DBLδ1	CIDRβ6						B21		supercontig_1.8
RAJ116var14	RAJ116	B5	B3	DBLα0.13	CIDRα2.9	DBLδ1	CIDRγ8	DBLζ6	DBLε9						supercontig_1.9
RAJ116var10	RAJ116	B5	B3	DBLα0.9	CIDRα2.1	DBLδ1	CIDRγ10						B2		supercontig_1.8
RAJ116var39	RAJ116	B6	B3	DBLα0.10	CIDRα2.8	DBLδ3	CIDRγ2	DBLε13	DBLγ2						supercontig_1.384
RAJ116var31	RAJ116	B6	B3	DBLα0.5	CIDRα2.6	DBLδ1	CIDRβ5						B7		supercontig_1.178
RAJ116var28	RAJ116	C1	B3	DBLα0.15	CIDRα3.2	DBLε2	DBLε7	DBLε3					B4		supercontig_1.107
RAJ116var27	RAJ116	C1	B3	DBLα0.5	CIDRα2.3	DBLδ1	CIDRβ1						B14		supercontig_1.104
RAJ116var21	RAJ116	C1	B3	DBLα0.20	CIDRα3.3	DBLδ1	CIDRβ5						B5		supercontig_1.30
RAJ116var35	RAJ116	C2	B6	DBLα0.18	CIDRα2.1	DBLβ2	DBLγ10	DBLδ4	CIDRγ1				B17		supercontig_1.204
RAJ116var26	RAJ116	B4	B3	DBLα0.15	CIDRα2.5	DBLδ1	CIDRγ7	DBLζ6	DBLε9				B14		supercontig_1.97
RAJ116var25	RAJ116	E	pam	DBLpam1	DBLpam2	CIDRpam	DBLpam3	DBLεpam4	DBLεpam5	DBLε10	DBLε14		pam1		supercontig_1.77
RAJ116var12	RAJ116			CIDRβ2	DBLγ7	DBLε11	DBLζ2	DBLε6					A1		supercontig_1.8
RAJ116var20	RAJ116			CIDRβ2	DBLγ7	DBLε11	DBLζ3	DBLε6					A3		supercontig_1.27
AAQ73929	Other PF	A3	A3	DBLα1.3	DBLε8								var3		
AAQ73927	Other PF	A3	A4	DBLα1.5	CIDRδ1	DBLβ3	DBLγ11						A1		
AAQ73928	Other PF	A3	A6	DBLα1.7	CIDRα1.4	DBLβ3	DBLγ13	DBLγ2	DBLγ6				A1		
AAB60251	Other PF	B2*	B3	DBLα0.11	CIDRα2.8	DBLδ1	CIDRγ9	DBLζ6	DBLε6				B1		
AAQ73930	Other PF	E	pam	DBLpam1	DBLpam2	CIDRpam	DBLpam3	DBLεpam4	DBLεpam5	DBLε10			pam1		
AF547159	Other PF			DBLα1.1	CIDRα1.3	DBLβ1	DBLγ15							F	
AAN86340	Other PF			DBLα1.1	CIDRα1.3	DBLβ1	DBLγ15							F	
AJ420411	Other PF	A3	A1	DBLα1.1	CIDRα1.2	DBLβ11	DBLγ1	DBLε1	DBLγ8	DBLζ2	DBLε5				
ACJ68328	Other PF		A7	DBLα1.6	CIDRγ6	DBLβ7	DBLγ14	DBLε5	DBLε4						
AAM55194	Other PF			DBLα1.1	CIDRα1.2	DBLβ11	DBLγ1	DBLε1	DBLγ8	DBLζ2	DBLε5			F	
TM284S2var1	Other PF		B3	DBLα0.9	CIDRα2.7	DBLβ5	DBLγ11	DBLδ2	CIDRγ6					F	
AY028643	Other PF		B3	DBLα0.8	CIDRα2.10	DBLβ5	DBLγ5	DBLδ1	CIDRβ1					F	
TM284var3	Other PF		A1	DBLα1.1	CIDRα1.2	DBLβ11	DBLγ1							F	
PREICHvar71	PREICH	A3	A4	DBLα1.4	CIDRα1.6	DBLβ3								F	c000023807
PREICHvar43	PREICH	A3	A5	DBLα1.4										F	c000023705
PREICHvar29	PREICH	A3	A4	DBLα1.4										F	c000023445
PREICHvar54	PREICH	B3*	B3	DBLα0.6	CIDRα3.1									F	c000023531
PREICHvar67	PREICH	B3*	B3	DBLα0.1	CIDRα3.4									F	c000023479
PREICHvar52	PREICH	B3*		DBLα0.6	CIDRα3.1									F	c000023533
PREICHvar35	PREICH	B3*	B3	DBLα0.10										F	c000023441
PREICHvar85	PREICH	B4	B3	DBLα0.8	CIDRα5	DBLβ8	DBLδ1							F	c000023763
PREICHvar83	PREICH	B4	B1	DBLα0.12	CIDRα2.1	DBLβ3								F	c000023128
PREICHvar92	PREICH	B4	B3	DBLα0.8	CIDRα2.2	DBLδ1								F	c000023831
PREICHvar53	PREICH	B4	B3	DBLα0.8										F	c000027354
PREICHvar31	PREICH	B4	B3	DBLα0.10										F	c000023707
PREICHvar62	PREICH	C2	B3	DBLα0.1										F	c000023044
PREICHvar90	PREICH		B3	DBLα0.1	CIDRα3.2	DBLδ1	CIDRβ1								c000124819
PREICHvar93	PREICH		B3	DBLα0.16	CIDRα3.4	DBLδ1	CIDRβ1								c000023766
PREICHvar61	PREICH		B3	DBLα0.5										F	c000023252
PREICHvar55	PREICH		B3	DBLα0.9										F	c000027275
PREICHvar28	PREICH		B3	DBLα0.6										F	c000023005
PREICHvar64	PREICH	E	pam	DBLpam1	DBLpam2	CIDRpam								F	c000130266
PREICHvar82	PREICH			CIDRα2.1	DBLβ10	DBLδ1	CIDRβ3							F	Contig7
PREICHvar81	PREICH			DBLε13	DBLζ5	DBLε4								F	c000023779
PREICHvar69	PREICH			DBLδ1	CIDRβ2	DBLγ11	DBLε4							F	c000023910
PREICHvar96	PREICH			DBLγ12	DBLδ5	CIDRβ4	DBLβ9	DBLγ9						F	c000023859
PREICHvar46	PREICH			DBLβ3	DBLγ11									F	c000023399
PREICHvar37	PREICH			DBLδ1	CIDRβ1								B10	F	c000023788
PREICHvar77	PREICH			DBLδ1	CIDRβ5								B10	F	c000023794
PREICHvar49	PREICH			CIDRα4	DBLδ1	CIDRβ5								F	c000023293
PREICHvar38	PREICH			DBLβ8	DBLδ1	CIDRβ1								F	c000023820
PREICHvar44	PREICH			CIDRα2.11	DBLβ8									F	c000023304
PREICHvar45	PREICH			CIDRγ2	DBLγ11								B19	F	c000022251
PREICHvar27	PREICH			DBLα0.8										F	c000022469
