# Supplementary material for: Plasmodium falciparum Erythrocyte Membrane Protein 1 Diversity in Seven Genomes – Divide and Conquer
Source: PLoS Comput Biol. 2010 Sep 16;6(9):e1000933. doi: 10.1371/journal.pcbi.1000933 (PMC2940729; doi:10.1371/journal.pcbi.1000933)
Supplement: Table S1 — Examples of HB combinations specific for DBL and CIDR domain classes. Domain counts and number of matches of the HB combination are given for the sequence set with 311 PfEMP1 sequences. The domain combination (17, 19) signifies a sequence where both HB17 and HB19 are present. These homology blocks are suggested for use in oligonucleotide array design, as well as for functional analysis of the domain types. The list is not exhaustive, and can be supplemented using Figure 6 and Figure 8, as well as the VarDom server. (0.11 MB PDF) [file pcbi.1000933.s010.pdf]

Table S1

Domain class specific homology blocks

**Domain** [Number of domains]	**HB combination**	**Found in domain / other**	**Domain coverage**
**DBLα** [301]	(19)	300 / 6	99.7%
	(17, 19)	299 / 1	99.3%
**DBLβ** [119]	(62)	119 / 13	100%
	(53, 61)	118 / 0	99.2%
**DBLγ** [145]	(15, 45)	109 / 0	75.2%
	(16, 45)	88 / 0	60.7%
	(15, 18)	86 / 0	59.3%
	(86)	70 / 1	48.3%
	(94)	58 / 3	40.0%
	(82, 45)	48 / 0	33.1%
	(364)	9 / 0	6.2%
	(11, 275)	9 / 0	6.2%
	All above united	144 / 4	99.3%
**DBLδ** [254]	(15, 9)	253 / 0	99.6%
	(35)	213 / 1	83.9%
**DBLζ** [61]	(92)	61 / 0	100%
	(93)	61 / 0	100%
**DBLε** [133]	(76)	87 / 0	65.4%
	(48, 78)	54 / 1	40.6%
	(108)	43 / 0	32.3%
	(310)	20 / 0	15.0%
	(265)	12 / 0	9.0%
	(260)	12 / 1	9.0%
	All above united	127 / 2	95.5%
**CIDRα** [270]	(23)	268 / 3	99.3%
	(32)	228 / 0	84.4%
**CIDRβ** [170]	(50)	169 / 1	99.4%
**CIDRγ** [94]	(85) U (176) U (212) U (384) U (522) U (565) U (22, 521) U (627)	93 / 0	98.9%
**CIDRδ** [19]	(202)	19 / 0	100%
