# Supplementary material for: Plasmodium falciparum Erythrocyte Membrane Protein 1 Diversity in Seven Genomes – Divide and Conquer
Source: PLoS Comput Biol. 2010 Sep 16;6(9):e1000933. doi: 10.1371/journal.pcbi.1000933 (PMC2940729; doi:10.1371/journal.pcbi.1000933)
Supplement: Table S2 — Homology blocks specific for component 1–4 ( Figure 12 ). Homology block numbers are given in parenthesis, and number of occurrences in the component with 311 sequences, is given next to the number of occurrences elsewhere. These homology blocks are suggested for use in oligonucleotide array design, as well as for functional analysis of the components. The table is not exhaustive. (0.09 MB PDF) [file pcbi.1000933.s011.pdf]

Table S2

Component specific homology blocks

Component	HB	Found in comp./other	HB	Found in comp./other
**Comp.1**	(19)	301 / 5	(75)	93 / 0
	(17)	300 / 36	(79)	74 / 2
	(20)	300 / 0	(88)	66 / 0
	(23)	269 / 4	(102)	51 / 0
	(32)	228 / 1	(104)	50 / 0
	(36)	203 / 1	(110)	47 / 0
	(54)	157 / 1	(114)	41 / 1
	(57)	146 / 7	(116)	41 / 0
	(60)	139 / 0	(118)	40 / 0
	(63)	132 / 0	(121)	40 / 0
	(64)	130 / 1	(130)	36 / 0
	(67)	111 / 0	(135)	35 / 0
	(68)	111 / 0	(125)	34 / 3
	(70)	106 / 0	(143)	33 / 0
	(71)	94 / 10	(150)	32 / 0
**Comp.2**	(26)	220 / 42	(139)	30 / 5
	(30)	183 / 56	(129)	28 / 9
	(49)	156 / 15	(174)	22 / 2
	(59)	125 / 18	(187)	21 / 0
	(61)	123 / 13	(189)	21 / 0
	(62)	114 / 19	(183)	20 / 2
	(65)	111 / 20	(190)	20 / 1
	(86)	60 / 11	(199)	18 / 1
	(101)	53 / 0	(209)	17 / 1
	(94)	49 / 12	(234)	15 / 0
	(119)	40 / 0	(241)	14 / 0
	(136)	34 / 1	(246)	14 / 0
	(141)	34 / 0	(232)	13 / 2
	(146)	32 / 0	(250)	13 / 0
	(134)	31 / 5	(255)	13 / 0
**Comp.3**	(22)	254 / 30	(103)	51 / 0
	(24)	240 / 29	(105)	50 / 0
	(27)	236 / 29	(106)	48 / 0
	(35)	215 / 0	(107)	47 / 0
	(29)	211 / 52	(109)	47 / 0
	(50)	164 / 6	(122)	39 / 0
	(55)	157 / 1	(138)	35 / 0
	(72)	102 / 0	(132)	34 / 2
	(73)	88 / 13	(149)	32 / 0
	(77)	83 / 2	(156)	29 / 0
	(81)	75 / 0	(158)	28 / 0
	(85)	70 / 2	(159)	27 / 0
	(87)	70 / 0	(165)	27 / 0
	(83)	60 / 14	(172)	25 / 0
	(96)	54 / 2	(173)	25 / 0
**Comp.4**	(31)	230 / 1	(137)	35 / 0
	(33)	176 / 45	(142)	34 / 0
	(56)	150 / 6	(115)	31 / 10
	(76)	87 / 0	(160)	23 / 0
	(93)	62 / 0	(195)	20 / 0
	(92)	61 / 1	(200)	19 / 0
	(99)	52 / 2	(205)	19 / 0
	(95)	50 / 6	(206)	19 / 0
	(97)	41 / 5	(207)	18 / 0
	(108)	41 / 2	(213)	18 / 0
	(123)	38 / 0	(221)	16 / 1
	(124)	38 / 0	(222)	16 / 0
	(126)	37 / 0	(224)	16 / 0
	(127)	37 / 0	(227)	16 / 0
	(120)	36 / 3	(235)	15 / 0
