# Supplementary material for: Plasmodium falciparum Erythrocyte Membrane Protein 1 Diversity in Seven Genomes – Divide and Conquer
Source: PLoS Comput Biol. 2010 Sep 16;6(9):e1000933. doi: 10.1371/journal.pcbi.1000933 (PMC2940729; doi:10.1371/journal.pcbi.1000933)
Supplement: Text S1 — PfEMP1 domain classification by alignment and distance tree analysis. (0.15 MB PDF) [file pcbi.1000933.s012.pdf]

Text S1 – PfEMP1 domain classification by alignment and distance tree analysis

Domain border definitions from domain alignments

In order to redefine NTS, DBL and CIDR domain borders, amino acid sequences for each identified DBL and CIDR domain, defined by the characteristics described by Smith *et al.* [1], plus additional 200 AA in both the N-terminal and C-terminal ends, were aligned. Based on the alignments, new universal DBL and CIDR domain borders were defined. All DBL domains could be aligned N-terminally from a Cys upstream to the first DBL amino acid defined by Smith *et al*. Consequently the NTS (N-terminal Segment) was defined shorter than previously. In addition, all DBLβ and DBLζ domains were flanked by an additional ~60 AA N-terminal sequence, which could not consistently be allocated to the neighboring domain and thus were allocated to DBLβ and DBLζ domains. In their C-terminal end, all DBL domains could be aligned to CysX(X)Cys, which in 3D crystal structures of DBL domains take part in forming one of three bundled helices. Therefore, what has previously been defined as "C2" and the first half of M1 in CIDR is now the C-terminal H3 helices (major subdomain [2], subdomain 3 [3]) of DBLβ and DBLα/DBLδ domains, respectively. The N-terminal of CIDR domains were defined immediately after the upstream DBL domain. The CIDR C-terminal has previously been defined by the start of the subsequent DBL domain. However, CIDR domains only uniformly aligned to the Cys corresponding to the end of the M2 region. The M2 region corresponds to the minimal CD36 binding domain [4] and its predicted fold is homologous to the three-helix bundle of DBL domains [5]. The region known as M3 covers the inter-domain sequence between CIDR domains and their downstream following domain. Sequences downstream of DBLα-CIDR are either long (mean length $\mu=80$ AA, 95% CI:3) if flanked C-terminally by a DBLδ-CIDR or short ($\mu=28$ AA, 95% CI:3) if not. Sequences following DBLδ-CIDR contain either one (M3A) or two (M3AB) conserved Cys spaced by variant Pro and polyAsp/Glu rich sequences (Figure S3Z). M3A were typically flanked by DBL domains whereas M3AB borders TM-ATS (Figure S4). Thus, length and amino acid composition of the sequence tagging on to CIDR domains, appeared to depend primarily on the type of the following PfEMP1 domain, rather than the phylogenetic classification of the CIDR domain. Therefore, the Cys residues in M3 may or

may not be part of the flanking CIDR domain, and in this annotation M2 is defined as the C-terminal of CIDR domains and M3 is treated separately. However, since only sequences following DBLδ-CIDR could be characterized by conserved residues, only these sequences were named M3A or M3AB, whereas all other inter-domain sequences were annotated as such.

Definition of PfEMP1 domain subclasses from distance trees

Major PfEMP1 domain classes were identified in neighbor joining (NJ) trees built from alignments of all NTS (369 sequences), DBL (1242 sequences), CIDR (655 sequences), and ATS (228 sequences) domains. Due to the sequence diversity and large number of sequences, good alignments for subclassification of each of the six major DBL classes (α, β, γ, δ, ε, and ζ) could only be obtained by realigning each major class by itself. Maximum likelihood (ML) trees were built from these alignments and DBL subclasses were defined using the following recursive approach:

ML tree bipartitions with 50% or higher bootstrap support and at least three of the seven genomes represented in both clades were considered as potential subdivisions. Clusters formed by the potential divisions were evaluated by calculating the within cluster average distance (WCAD), and the bipartition minimizing the WCAD (i.e. maximizing the within cluster average similarity) was found. The sequences were divided at the bipartition minimizing the WCAD, if the WCAD was significantly low ($p < 0.1$) compared to random division of the sequences, estimated by generating 100000 shuffled bipartitions and calculating a WCAD distribution from these. If the bipartition resulted in two small clusters (less than six of seven genomes represented in both clusters), the division was only made if the WCAD was very significant ($p < 0.0002$). If a split was made, sequences from the resulting two clusters were realigned, ML trees were built and these were then examined for the possibility of further subdivision. Clusters which could not be subdivided were each given a number, and thus all sequences were assigned to a subclass.

ML trees of all major DBL classes are shown in Figures S2A-G, logos for the major DBL classes are given in Figures S3I-X. Also CIDR domains were first divided into major classes by NJ distance trees built on alignments spanning from the end of the preceding DBL to end of M2. The Inter Domain 2 of VAR2CSA has previously been shown to be partially homologous to CIDR domains and was therefore included in this analysis [6]. CIDR domains

were divided into five major classes CIDRα, β, γ, δ and pam. The three large CIDR classes CIDRα, CIDRβ and CIDRγ were realigned separately to define subclasses using the same criteria as used for DBL domains. Traditionally, DBL and CIDR domains have been named according to their classification and their position in the PfEMP1 domain architecture given as a count of either DBL or CIDR domains (i.e. the first DBL and CIDR domain of PFD0005w would be named DBL1α1 and CIDR1α1, where the number before the Greek suffix relates to the domain count and the number after the Greek suffix specify the domain type). As the positional numbering most often is non-informative this was omitted in all figures and only used in the text when providing relevant positional information. Additionally, in the current classification, domain subclasses were differentiated by a suffix appended to the major class definer (i.e. DBLα of the 3D7 *var1* variant PFE1640w was defined as DBLα1.4). NTS and ATS were subjected to distance tree analyses similar to that of DBL and CIDR domains. ML trees for all domains are shown in Figures S2A-M, and the percentage of average shared amino acids (%IDs) of all DBL and CIDR classes are shown in Figure 2.

1. Smith JD, Subramanian G, Gamain B, Baruch DI, Miller LH (2000) Classification of adhesive domains in the Plasmodium falciparum erythrocyte membrane protein 1 family. Mol Biochem Parasitol 110: 293-310.
2. Singh SK, Singh AP, Pandey S, Yazdani SS, Chitnis CE, et al. (2003) Definition of structural elements in Plasmodium vivax and P. knowlesi Duffy-binding domains necessary for erythrocyte invasion. Biochem J 374: 193-198.
3. Higgins MK (2008) The structure of a chondroitin sulfate-binding domain important in placental malaria. J Biol Chem 283: 21842-21846.
4. Baruch DI, Ma XC, Singh HB, Bi X, Pasloske BL, et al. (1997) Identification of a region of PfEMP1 that mediates adherence of Plasmodium falciparum infected erythrocytes to CD36: conserved function with variant sequence. Blood 90: 3766-3775.
5. Klein MM, Gittis AG, Su HP, Makobongo MO, Moore JM, et al. (2008) The cysteine-rich interdomain region from the highly variable plasmodium falciparum erythrocyte membrane protein-1 exhibits a conserved structure. PLoS Pathog 4: e1000147.
6. Andersen P, Nielsen MA, Resende M, Rask TS, Dahlback M, et al. (2008) Structural insight into epitopes in the pregnancy-associated malaria protein VAR2CSA. PLoS Pathog 4: e42.
