# Supplementary material for: Plasmodium falciparum Erythrocyte Membrane Protein 1 Diversity in Seven Genomes – Divide and Conquer
Source: PLoS Comput Biol. 2010 Sep 16;6(9):e1000933. doi: 10.1371/journal.pcbi.1000933 (PMC2940729; doi:10.1371/journal.pcbi.1000933)
Supplement: Text S2 — Defining PfEMP1 homology blocks. (0.81 MB PDF) [file pcbi.1000933.s013.pdf]

Text S2 - Defining PfEMP1 homology blocks

In order to identify conserved sequence blocks in the PfEMP1 family, such as the previously found homology blocks in DBL domains [1-2], a method was developed that takes a set of unaligned protein sequences and subdivides these into blocks of homologous sequences using only sequence conservation level, dictated by a fixed similarity threshold, as criterion for delimiting the blocks. The resulting homology blocks describe local similarities between PfEMP1, and are each defined by a multiple sequence alignment, as well as a probabilistic model, i.e. a hidden Markov model (HMM) [3].

A serial iterative approach was employed, where one homology block was defined per iteration, as the most conserved sequence in the database (Figure A). The conservation of a homology block was assessed by number of occurrences in the database. Uncovering the most conserved homology block was implemented as an optimization process where boundaries and start conditions were altered in three steps: (1) Up to 100 different start sequence profiles (termed seed sequences) were crudely selected using BLAST, each to potentially form a homology block. (2) The conservation level was determined for each seed

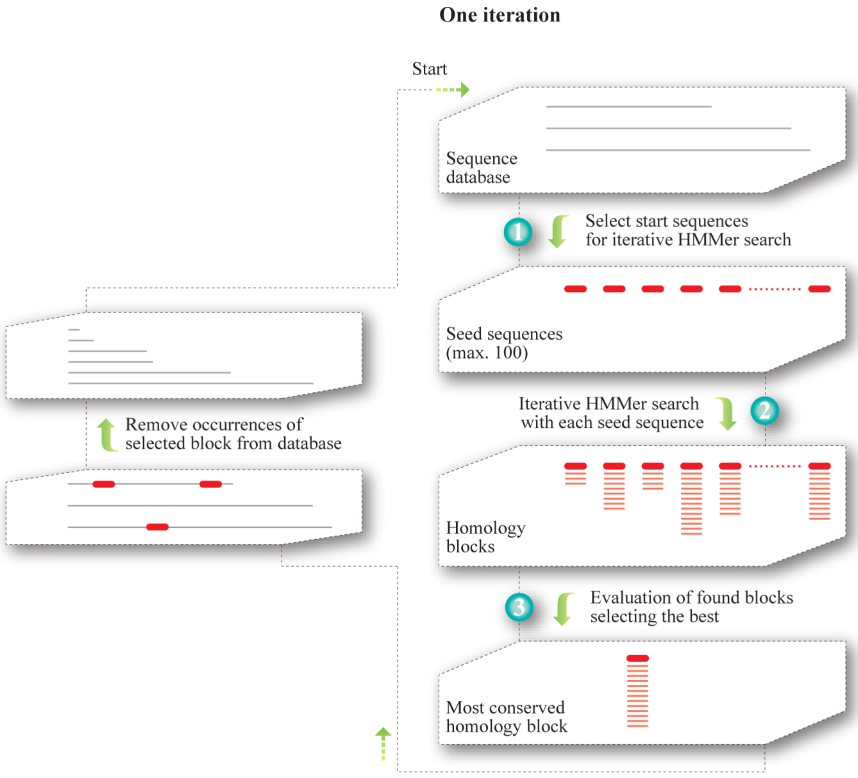

Figure A: One iteration defining one homology block. Flow diagram describing one iteration in the process of automatically defining a set of homology blocks covering the sequence database. Different stages in the iterative process are marked by boxes, processes are marked by arrows. The steps 1-3 are elaborated in Figure B, C, and D.

sequence by iterative HMM searches, while the sequence profile was continually updated with similar sequences. When the HMM was optimal, with maximal number of occurrences in the sequence set, the boundaries were gradually changed and optimized also with regard to number of occurrences. (3) One optimized homology block was finally selected, taking both reproducibility and conservation level into account. Subsequently the members of the selected homology block were removed from the database to avoid overlap in the following iteration.

Step 1 - Choosing seed sequences

Seed sequences refer to the initial sequences from which the thorough homology search in step 2 was started. Selection of these sequences was an important step because it roughly determined the length and sequence profile of the homology blocks. To obtain homology blocks which describe the short conserved sequences with few gaps, as is characteristic for the previously described homology blocks in DBL domains, ungapped BLAST was initially used to select seed sequences. These seed sequences should only cover one homology block each, to avoid defining mixed blocks leading to alignments with many gaps. Also, the seeds with most homologs should be selected first, as homology blocks with lower conservation could otherwise be extended during optimization to include parts of more conserved homology blocks. The process of selecting seed sequences is depicted in Figure B, where the entire homology block analysis is divided into three phases according to the level of local homology in the database, each phase employing different strategies.

During the first phase, ungapped all vs. all BLAST search [4] was performed. BLAST hits with expectation value $E < 1$ were used to establish a pairwise homology network to discover sequences with a high concentration of homology to other parts of the database. Each residue in the database could be part of a different set of BLAST hits, each hit with different start and end positions, so to find the most likely boundaries for the homology block that one particular residue belonged to, the boundary pair with highest BLAST hit support was determined. The hit support for a start and end position pair was defined as the number of BLAST hits starting and ending within two windows (5 amino acid width) centered at the start and end positions. So for all residues in the database, the surrounding boundary pair with highest hit support was found, and all these pairs were collected to form a set of optimally delimited sequence

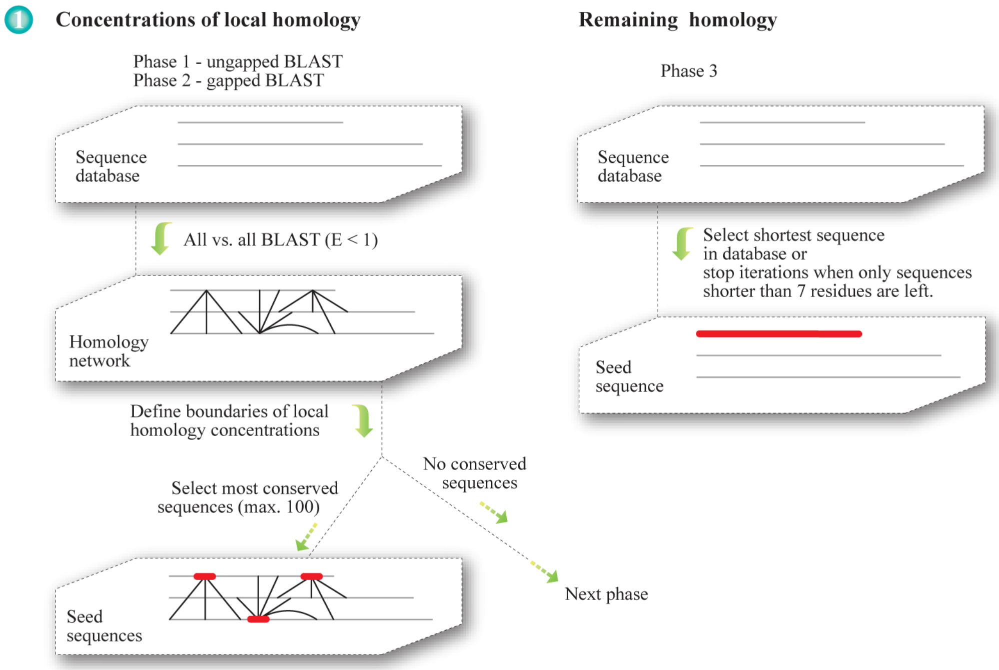

Figure B: Selection of seed sequences (step 1 in Figure A).

fragments, with varying degrees of homology to other parts of the database. Up to 100 of the most conserved sequences from the delimited set were used as seed sequences, where conservation was assessed by the number of pairwise BLAST hits overlapping the entire sequence fragment. A minimal conservation level of 2 hits was used, before proceeding to next phase. By using this approach for selection of boundaries, both homology level and coinciding start and end positions of homologous sequences, were taken into account.

In phase 2, normal gapped BLAST was used to detect homology which had escaped the ungapped BLAST, otherwise seeds were selected as in phase 1. Only few homology blocks were found during phase 2.

To make sure no significant homology was left, all remaining fragments longer than 6 residues were in phase 3 searched against the database one by one.

BLAST searches were performed without low complexity filter to avoid hit boundaries being affected by the SEG algorithm. Instead, when two hits overlapped with both query and subject sequences, which is often the case in low complexity regions, then these two hits were combined to one longer hit covering query and subject sequences from both hits. Thus the high numbers of hits in low complexity regions were reduced, so that they reflected the

true number of non-overlapping homologous sequences in the database, which made the low complexity regions comparable to other regions.

Step 2 - Iterative HMMer

The iterative HMMer (iHMMer) algorithm uses the HMMer package (version 2.3.2) [5] to detect remote homologs, by iteratively building HMMs from query sequences, searching for homologs and including the search results in next iterations query. iHMMer is inspired by psi-BLAST [6], a fast algorithm using a position specific scoring matrix (PSSM) to describe the data, but when psi-BLAST encounters a hit with insertions compared to the query sequence, the insertion is simply discarded instead of included in the PSSM, resulting in loss of potentially useful information for the following iterations. Insertions are handled more thoroughly in iHMMer where they are incorporated in the HMM, and in contrast to PSSMs, HMMs encompass transition probabilities including position specific probabilities for insertions and deletions, resulting in more accurate alignment during searches. Another feature of iHMMer is the refinement iterations which makes the result less dependent on an optimal query (seed) sequence.

An overview of an iHMMer run is shown in Figure C, starting from a single query sequence selected in step 1. HMMs were built with default settings except the null model which was set to reflect amino acid frequencies and protein lengths in the 331 PfEMP1 dataset. The PfEMP1 database was searched with the null2 model disabled to allow detection of low-complexity regions. Sequence matches to the HMM scoring 9.97 bits or higher were considered true hits. The log-odds score threshold of 9.97 bits corresponds to demanding that the probability of the hit sequence given the HMM model should be 1000 times larger than the probability of the hit sequence given the null-model, a threshold which was empirically found to be a good compromise between including false positives and missing true homologs, judged by relative positions in the PfEMP1 sequences and correlations with other homology blocks. 10% of the positive hits (minimum one hit) were each iteration aligned to the HMM using hmmalign with default parameters, thus allowing the HMM to adapt to new sequences, giving a better alignment than if all hits were added at once. iHMMer continued iterations until all hits above the score threshold were included, then the iteration where most hits were

found, was selected as the best result, though to ensure a well defined homology block, only HMMs which could refind the exact sequences it was defined from, were selected.

Up to 10 rounds of refinement were performed, each round consisting of iterations with the alignment from the best iteration as query, modified by (A) truncation of alignment with one to seven residues in each side. (B) Extension of alignment with one to seven residues in each side. (C) thorough MAFFT alignment [7] using *Plasmodium falciparum* based substitution matrix [8]. Refinement was stopped when no improvements were achieved by a refinement round.

The results from iHMMer consists of a multiple sequence alignment defining an HMM, where the HMM can refind the exact definition sequences. Each homology block was defined as the most conserved sequence in a database where previously defined blocks had been removed, so to get the actual number of sequences pertaining to each HB, the full database was subsequently searched with the HMMs and an alignment of all homology block occurrences is provided.

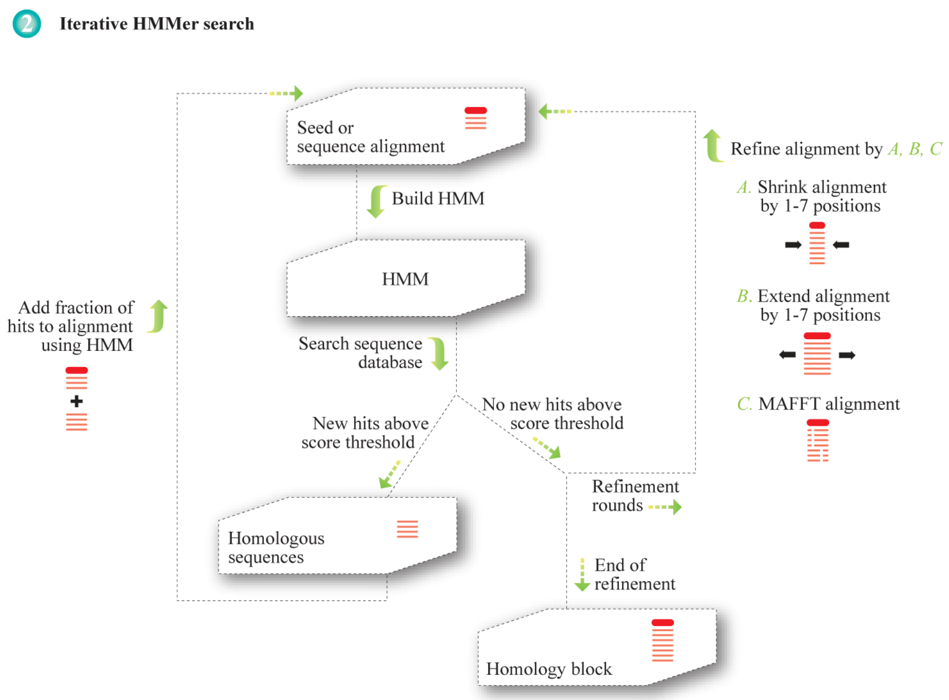

Figure C: Iterative HMM search (step 2 in Figure A).

Step 3 - Selecting the best homology block

Seed sequences which were different members of the same homology block, mostly resulted in the same number of hits when running iHMMer, although small differences did occur. When up to hundred blocks had been defined in step 1 and 2, one block was selected (Figure D) by taking into account both the number of hits as a measure of conservation, but also how many times the same block occurred, as a measure of how well parameter space had been sampled for that specific homology block, and thus how likely it was that the block was optimal. Similarity of blocks was estimated by overlap in the database, so that homology blocks were grouped, where 75% of the occurrences overlapped by 95% of the length. If homology block groups existed with more than ten members then only these groups were considered, and the most conserved member was selected. If no well sampled homology blocks were found, then the homology block with most hits in the database was selected from any group. All occurrences of the selected homology block were removed from the sequence database before the next iteration.

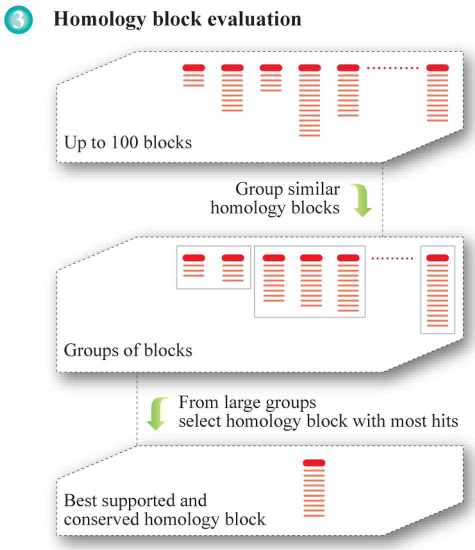

Figure D: Evaluating and selecting the best homology blocks (step 3 in Figure A).

1. Smith JD, Subramanian G, Gamain B, Baruch DI, Miller LH (2000) Classification of adhesive domains in the Plasmodium falciparum erythrocyte membrane protein 1 family. Mol Biochem Parasitol 110: 293-310.
2. Su XZ, Heatwole VM, Wertheimer SP, Guinet F, Herrfeldt JA, et al. (1995) The large diverse gene family var encodes proteins involved in cytoadherence and antigenic variation of Plasmodium falciparum-infected erythrocytes. Cell 82: 89-100.
3. Durbin R (1998) Biological sequence analysis : probabilistic models of proteins and nucleic acids. Cambridge: Cambridge University Press. xi, 357 p. p.
4. Altschul SF, Gish W, Miller W, Myers EW, Lipman DJ (1990) Basic local alignment search tool. J Mol Biol 215: 403-410.
5. Eddy S (2003) The HMMer package. http://hmmer.janelia.org/. 2.3.2 ed.
6. Altschul SF, Madden TL, Schaffer AA, Zhang J, Zhang Z, et al. (1997) Gapped BLAST and PSI-BLAST: a new generation of protein database search programs. Nucleic Acids Res 25: 3389-3402.

7. Katoh K, Misawa K, Kuma K, Miyata T (2002) MAFFT: a novel method for rapid multiple sequence alignment based on fast Fourier transform. Nucleic Acids Res 30: 3059-3066.
8. Brick K, Pizzi E (2008) A novel series of compositionally biased substitution matrices for comparing Plasmodium proteins. BMC Bioinformatics 9: 236.
