# Supplementary material for: Plasmodium falciparum Erythrocyte Membrane Protein 1 Diversity in Seven Genomes – Divide and Conquer
Source: PLoS Comput Biol. 2010 Sep 16;6(9):e1000933. doi: 10.1371/journal.pcbi.1000933 (PMC2940729; doi:10.1371/journal.pcbi.1000933)
Supplement: Text S3 — PfEMP1 DBL domain relations to CIDR and paralog DBL domains. (0.31 MB PDF) [file pcbi.1000933.s014.pdf]

Text S3 - PfEMP1 DBL domain relations to CIDR and paralog DBL domains

To facilitate identification of subgroups too indistinct to be detected with the chosen homology block similarity threshold, NJ-trees based on alignments of all homology blocks were built, and these are available on the VarDom server. The tree based on the HB1 alignment was studied to determine if it contained information on the relation between DBL and CIDR domains (Figure A). The bootstrap values of the tree were generally low due to sparse sequence information in the short alignment, however the tree topology captured many previously observed sequence relations (not shown). The ten VAR2CSA CIDRpam sequences (previously known as the VAR2CSA ID2 region) clustered closest to CIDR sequences, suggesting that CIDRpam is evolutionarily and possibly functionally closer related to CIDR domains than to DBL domains. This similarity has also previously been noted [1].

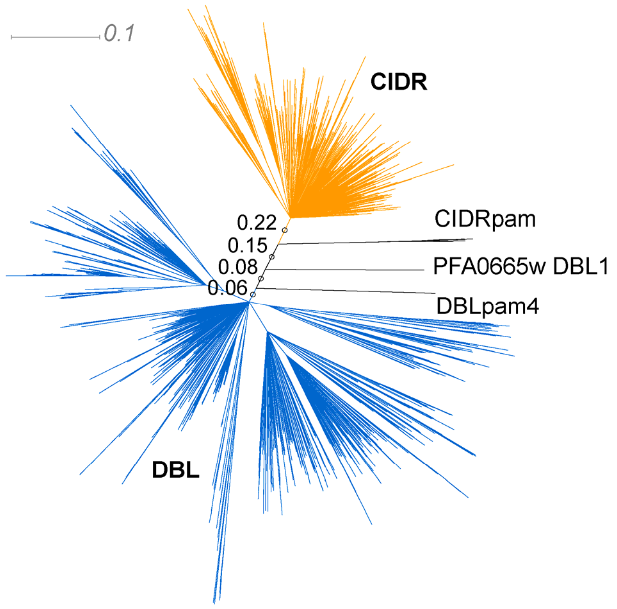

Figure A: Evolutionary history for the helix conserved in both DBL (blue) and CIDR (orange) domains. NJ-tree based on the 84 bp DNA alignment of HB1 with 1605 sequences. Bipartition support is given as a fraction of 1000 bootstraps.

PFA0665w, a previously undescribed PfEMP1 paralog, contains two DBL domains encoded by exon 1 (Figure B). PFA0665w was found to be conserved in all seven *P. falciparum* genomes as well as *P. reichenowi* with >70% identity, and expression has been detected in the HB3 clone early in the erythrocytic stages [2], suggesting that PFA0665w is not a pseudogene and that it has diverged from PfEMP1 before the split between *P. reichenowi* and *P. falciparum*. A third degraded DBL domain (DBL0) was found in the N-terminal of PFA0665w, upstream to DBL1, evidenced by the presence of HB2 in this area, as well as a weak hit to HB4 (S=5.0). A transmembrane helix was predicted by the TMHMM predictor

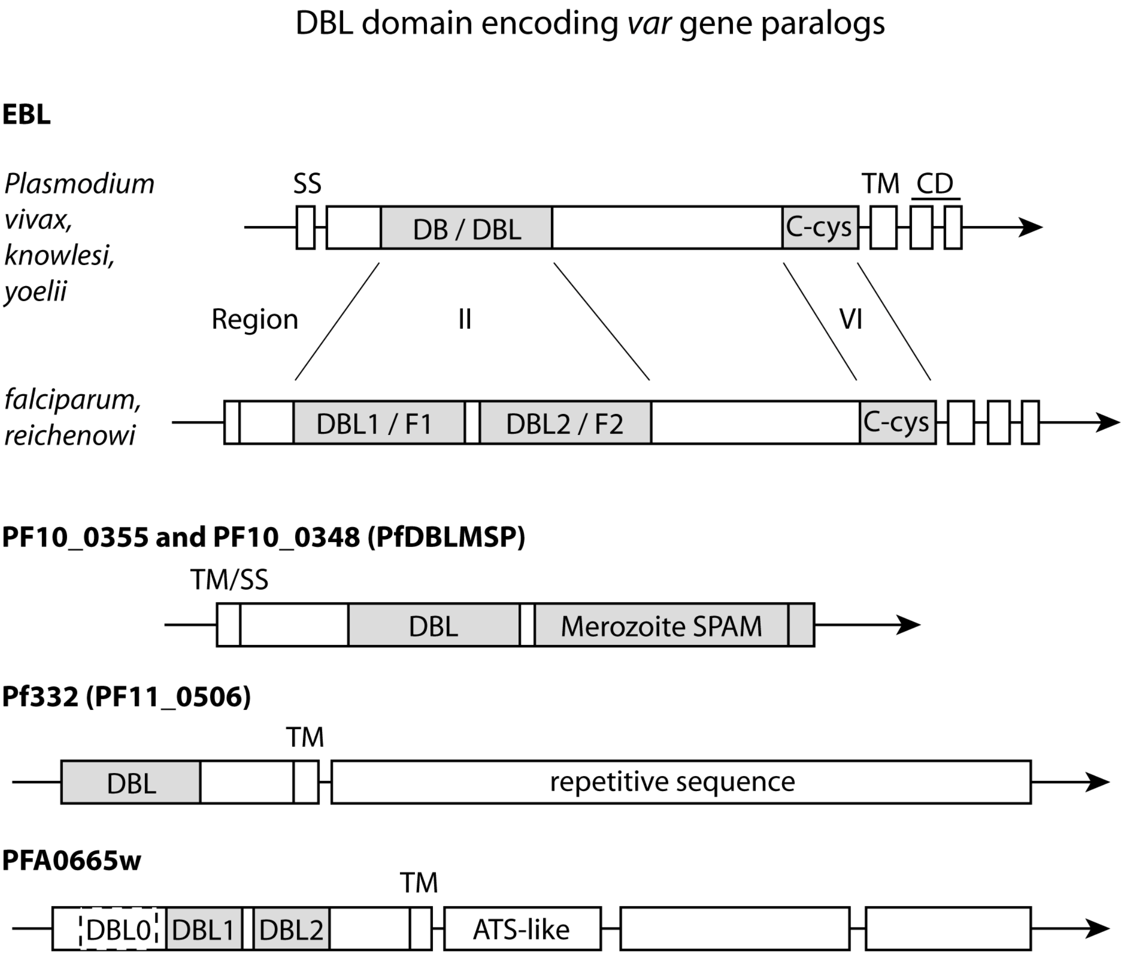

Figure B: *Var* gene paralog loci. Diagrams show the putative exon/intron structure of the DBL domain encoding *var* gene paralogs. Encoded domains are noted on the exons. SS: signal sequence; TM: transmembrane; CD: putative cytoplasmic domain; DB: Duffy binding; C-cys: Carboxyl Cys-rich region.

[3] to be encoded by the 3' end of exon 1, and not far downstream, in the ~500 amino acid stretch encoded by exon 2, was a weak but correctly positioned hit to HB43 (S=3.3) which is a conserved ATS homology block in all PfEMP1, together indicating that PFA0665w may be membrane exposed. The finding that HB43 is a very conserved part of the ATS could suggest an essential role for this region. Altogether, exon 1 and 2 of PFA0665w appears to be closely related to *var* genes.

The 3D7 genes PF10_0348 and PF10_0355 are members of a small gene family found to be conserved in *P. falciparum* and *P. reichenowi* with >70% identity, encoding proteins with one unique DBL domain followed by a merozoite SPAM domain (Figure B). PF10_0348 has

been localized on the merozoite surface and named PfDBLMSP [4]. Interestingly, two conserved regions in PfDBLMSP were identified by HB539 and HB538 in the N- and C-terminal respectively, and HB539 was predicted to contain a transmembrane helix (no signal peptide) by the Phobius predictor [5]. HB539 was also found in the N-terminal of S-antigen [6-7] (UniProt entries SANT_PLAFW and SANT_PLAFV) suggesting a similar localization of this protein. The DBL domain was most reminiscent of DBLε domains, supported by presence of HB97, HB108, HB160 and HB76. In PfEMP1, HB160 was found in a subset of DBLε domains flanking the ATS, including DBLε10 of VAR2CSA.

HB281 and HB282 were found in the C-terminal of all included members of the erythrocyte binding-like (EBL) family, covering both the transmembrane domain, several Cys residues (region VI of EBL [8]) as well as other conserved positions. HB421 was found specifically in EBL DBL1 (a.k.a. F1) domains, conserved in *P. falciparum, knowlesi* and *vivax*, and may be important for merozoite erythrocyte binding.

1. Andersen P, Nielsen MA, Resende M, Rask TS, Dahlback M, et al. (2008) Structural insight into epitopes in the pregnancy-associated malaria protein VAR2CSA. PLoS Pathog 4: e42.
2. Bozdech Z, Llinas M, Pulliam BL, Wong ED, Zhu J, et al. (2003) The transcriptome of the intraerythrocytic developmental cycle of Plasmodium falciparum. PLoS Biol 1: E5.
3. Krogh A, Larsson B, von Heijne G, Sonnhammer EL (2001) Predicting transmembrane protein topology with a hidden Markov model: application to complete genomes. J Mol Biol 305: 567-580.
4. Wickramarachchi T, Cabrera AL, Sinha D, Dhawan S, Chandran T, et al. (2009) A novel Plasmodium falciparum erythrocyte binding protein associated with the merozoite surface, PfDBLMSP. Int J Parasitol 39: 763-773.
5. Kall L, Krogh A, Sonnhammer EL (2004) A combined transmembrane topology and signal peptide prediction method. J Mol Biol 338: 1027-1036.
6. Bickle Q, Anders RF, Day K, Coppel RL (1993) The S-antigen of Plasmodium falciparum: repertoire and origin of diversity. Mol Biochem Parasitol 61: 189-196.
7. Anderson TJ, Day KP (2000) Geographical structure and sequence evolution as inferred from the Plasmodium falciparum S-antigen locus. Mol Biochem Parasitol 106: 321-326.
8. Michon P, Stevens JR, Kaneko O, Adams JH (2002) Evolutionary relationships of conserved cysteine-rich motifs in adhesive molecules of malaria parasites. Mol Biol Evol 19: 1128-1142.
