# Supplementary material for: Plasmodium falciparum Erythrocyte Membrane Protein 1 Diversity in Seven Genomes – Divide and Conquer
Source: PLoS Comput Biol. 2010 Sep 16;6(9):e1000933. doi: 10.1371/journal.pcbi.1000933 (PMC2940729; doi:10.1371/journal.pcbi.1000933)
Supplement: Figure S3 — PfEMP1 domain class logos. Sequence conservation logos for major PfEMP1 domain classes (panel A–Z): CIDRα, α1, α2, α3, β, δ, γ, pam; DBLα0, α1 (without α1.3), α1.3, β, δ, ε (without ε1, ε2, ε11, ε13, εpam), ε1, ε2, ε11, ε13, εpam4, εpam5, γ, pam1, pam2, pam3, ζ; NTSA, NTSB, and M3AB. (2.42 MB ZIP) [file Figure S3Q - Logo DBLe13.pdf › Figure S3Q - Logo DBLe13.pdf]

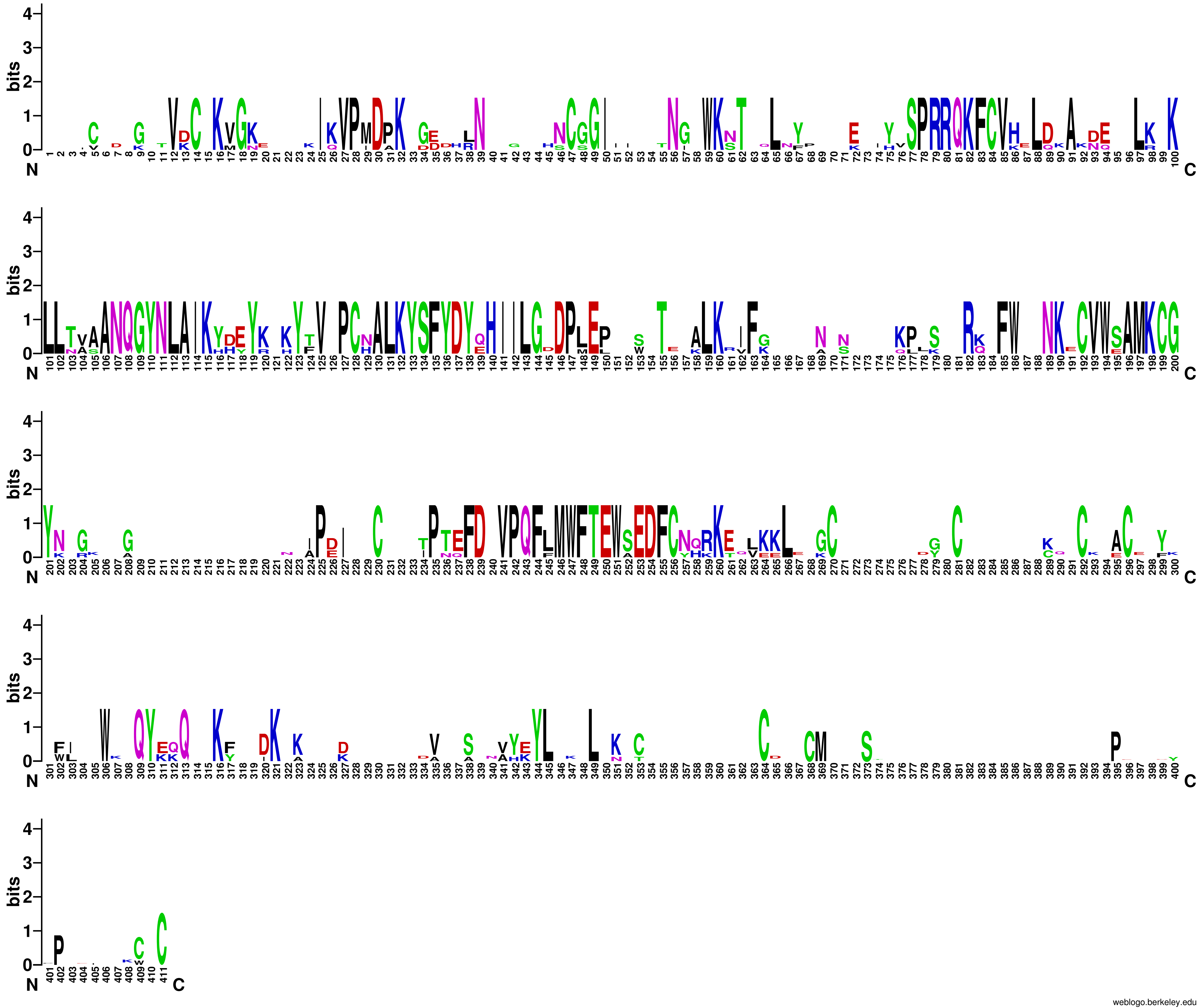
bits
N
C
weblogo.berkeley.edu
