# Supplementary material for: Plasmodium falciparum Erythrocyte Membrane Protein 1 Diversity in Seven Genomes – Divide and Conquer
Source: PLoS Comput Biol. 2010 Sep 16;6(9):e1000933. doi: 10.1371/journal.pcbi.1000933 (PMC2940729; doi:10.1371/journal.pcbi.1000933)
Supplement: Figure S4 — Annotated PfEMP1 sequences aligned according to C-terminal (A) and N-terminal (B) domain compositions. Gene names, parasite genome, 5′ UPS classes, PfEMP1 domain annotation (D = domain, ID = Inter Domain) and origin of sequence data (if sequence is not previously reported as var gene) are given. Sequences which partially contain unexpected identical sequence stretches to other sequences suggesting an incorrect contig assembly are noted “HBD” followed by the name of the potentially redundant sequence. Red arrows indicate component 1–4. Frames indicate clusters of correlated domain classes. 1:VAR1; 2: VAR2CSA; 3: VAR3; 4: DBLζ and DBLε domain combinations of component 4; 5: Cassette 10; 6: Cassette 6; 7: Cassette 9; 8: Cassette 5; 9: Other Group A PfEMP1 all containing component 2; 10: Cassette 8; 11: Group B and C genes containing component 2; 12: Group B and C PfEMP1 with no component 2 or 4; 13: Cassette 14; 14: Cassette 17,21 and 22; 15: DBLα1-CIDR subclass correlations including cassette 11,13,15 and 16; 16: DBLα0 subclasses associated with CIDRα3 subclasses; 17: DBLα0 subclasses associated with CIDRα2 subclasses. N-terminal segment (NTS), Duffy binding-like (DBL), Cys-rich inter-domain region (CIDR) and acidic terminal segment (ATS) are named according to the distance tree classification. Inter domains are annotated as either short if <32 AA (green) or long if >31 (yellow) and “A” or “B” if encoding M3A or M3AB. (0.15 MB PDF) [file pcbi.1000933.s005.pdf]

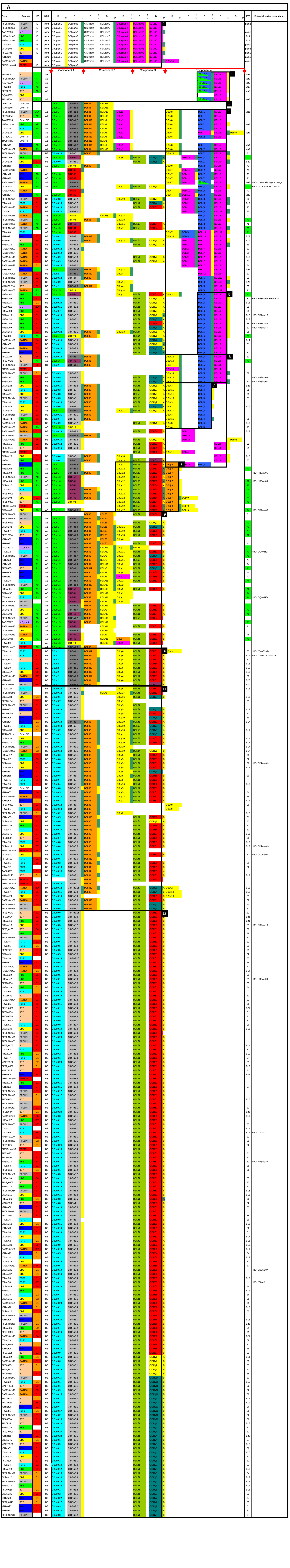
A
Gene
Parasite
UPS
NTS
ATS
Potential partial redundancy
Component 1
Component 2
Component 3
Component 4

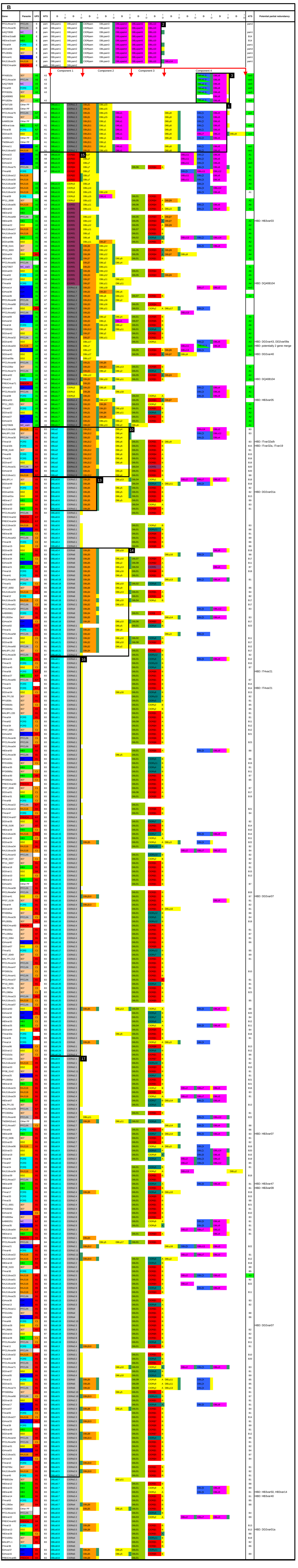
B
Gene
Parasite
UPS
NTS
ATS
Potential partial redundancy
Component 1
Component 2
Component 3
Component 4
