# Supplementary material for: Plasmodium falciparum Erythrocyte Membrane Protein 1 Diversity in Seven Genomes – Divide and Conquer
Source: PLoS Comput Biol. 2010 Sep 16;6(9):e1000933. doi: 10.1371/journal.pcbi.1000933 (PMC2940729; doi:10.1371/journal.pcbi.1000933)

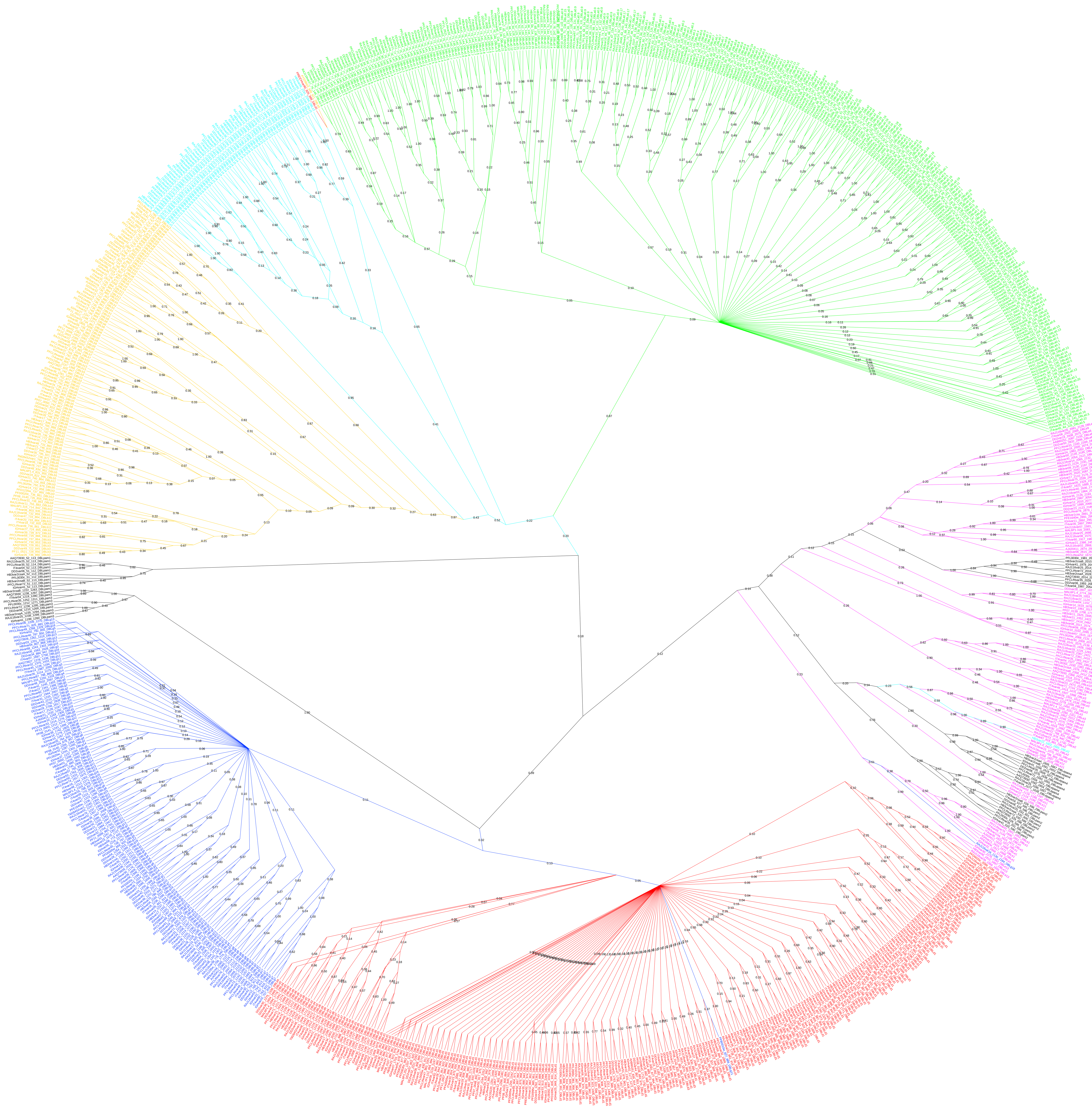

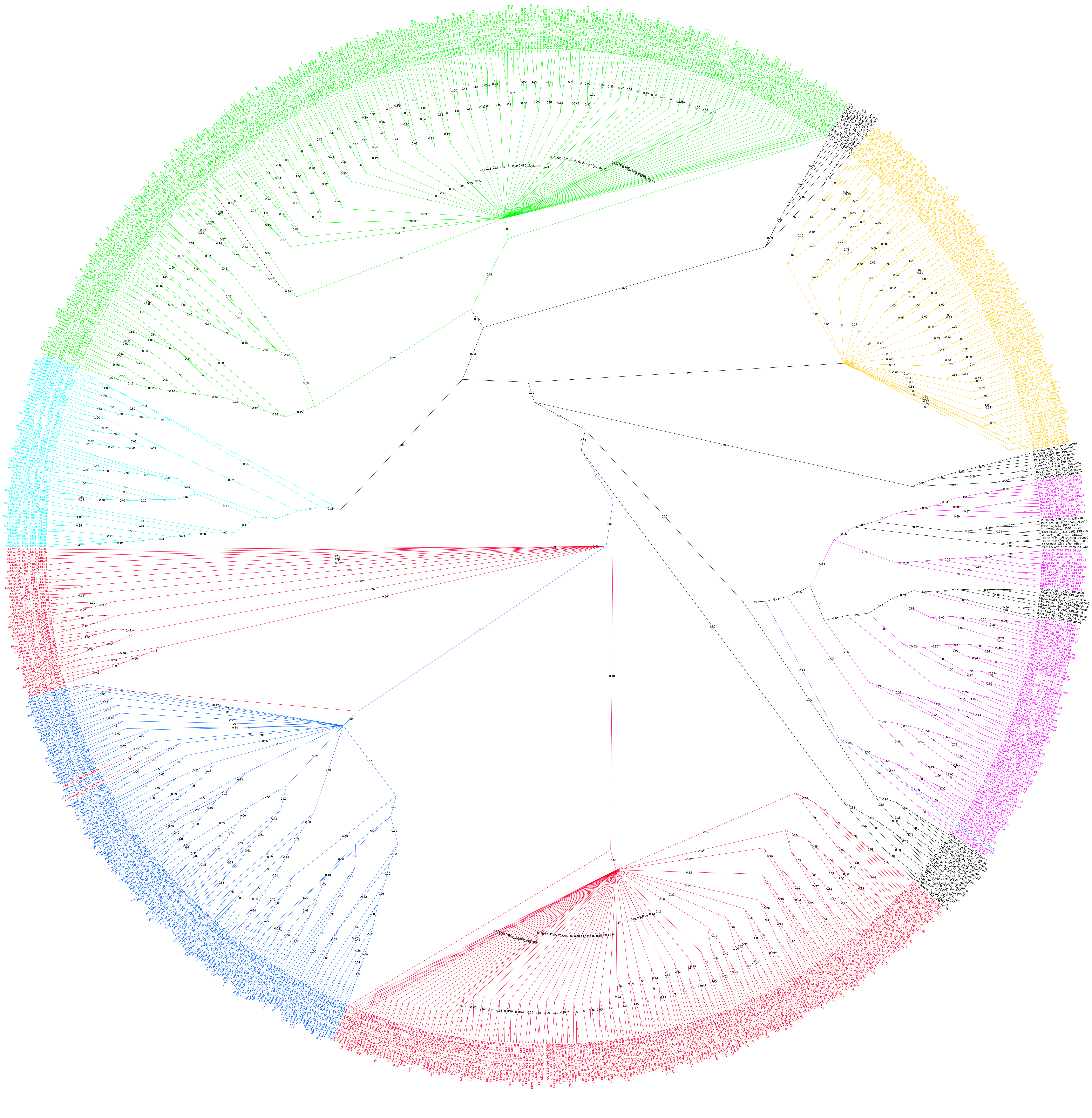

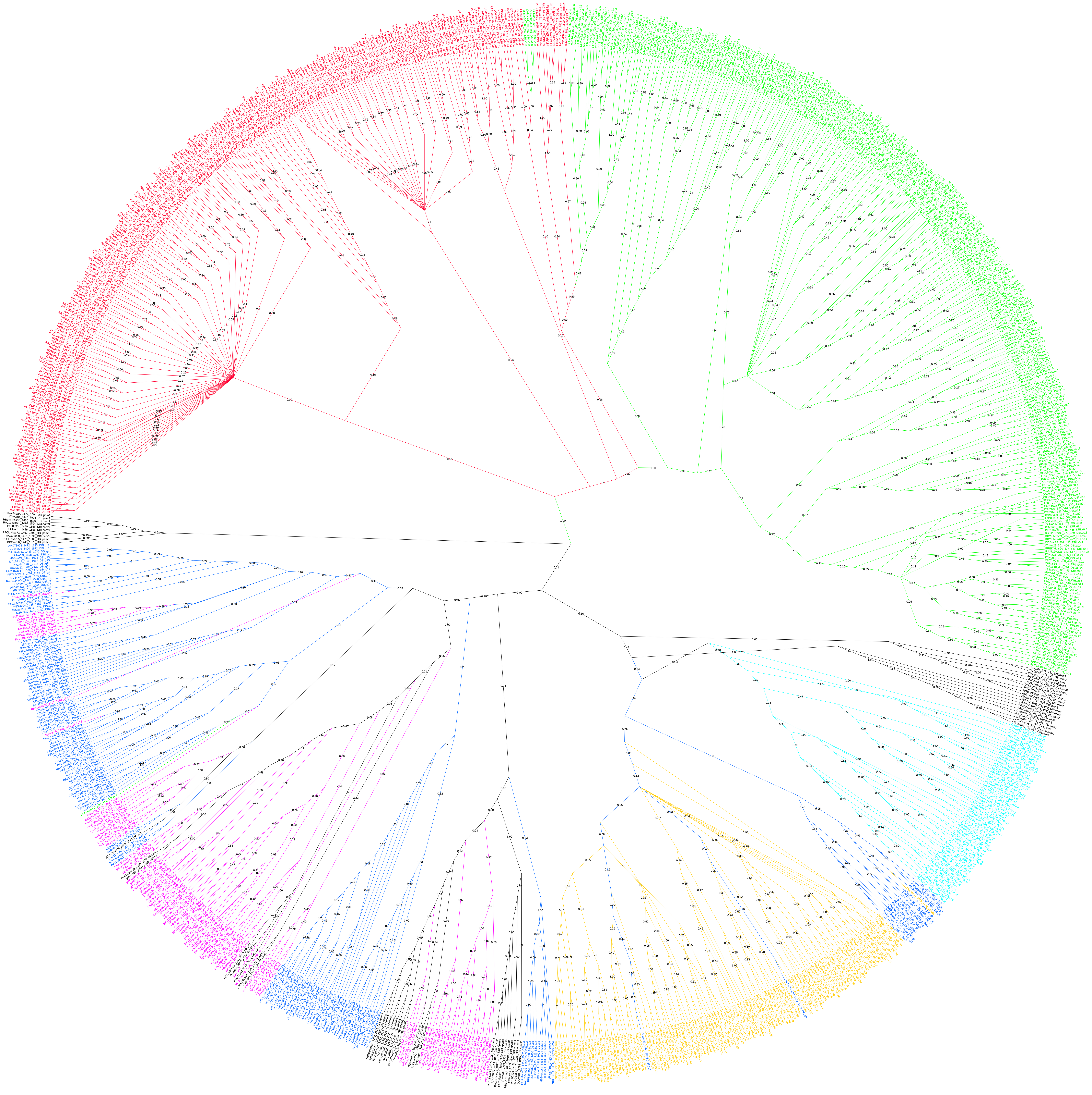

Supplement: Figure S6 — Phylogenetic trees for DBL subdomains S1, S2 and S3, as in Figure 7 but with labels. Edge values are fractions of 1000 bootstraps, and each subdomain is given as: protein name, start position, end position, and the domain class the subdomain is a part of. (0.22 MB PDF) [file pcbi.1000933.s007.pdf]
