# Supplementary material for: Plasmodium falciparum Erythrocyte Membrane Protein 1 Diversity in Seven Genomes – Divide and Conquer
Source: PLoS Comput Biol. 2010 Sep 16;6(9):e1000933. doi: 10.1371/journal.pcbi.1000933 (PMC2940729; doi:10.1371/journal.pcbi.1000933)
Supplement: Figure S7 — Homology block alignments. Homology block alignments for (panel A–E): DBL, CIDR, NTS, ATS, and whole PfEMP1, with details of Figure 6, Figure 8, Figure 10 and Figure 12. (0.82 MB ZIP) [file Figure S7C - HB alignment - NTS.pdf › Figure S7C - HB alignment - NTS.pdf]

# NTS

| SeqID_start_stop | NTS class | Homology blocks | | | | | | | | |
|---|---|---|---|---|---|---|---|---|---|---|
| RAJ116var05_1_76 | NTSA3 | - | - | - | HB155 | - | - | - | HB20 | HB17 |
| RAJ116var07_1_74 | NTSA4 | - | - | - | HB155 | - | - | - | HB20 | HB17 |
| DD2var09b_1_70 | NTSA6 | - | - | - | HB155 | - | - | - | HB20 | HB17 |
| RAJ116var19_1_75 | NTSA7 | - | - | - | HB155 | - | - | - | HB20 | HB17 |
| IGHvar14_1_75 | NTSA2 | - | - | - | HB155 | - | - | - | HB20 | HB17 |
| IT4var02_1_70 | NTSA6 | - | - | - | HB155 | - | - | - | HB20 | HB17 |
| IT4var08_1_71 | NTSA6 | - | - | - | HB155 | - | - | - | HB20 | HB17 |
| PF11_0008_1_70 | NTSA6 | - | - | - | HB155 | - | - | - | HB20 | HB17 |
| PF08_0141_1_75 | NTSA5 | - | - | - | HB155 | - | - | - | HB20 | HB17 |
| PFCLINvar75_1_75 | NTSA5 | - | - | - | HB155 | - | - | - | HB20 | HB17 |
| PFCLINvar73_1_71 | NTSA2 | - | - | - | HB155 | - | - | - | HB20 | HB17 |
| IGHvar30_1_75 | NTSA2 | - | - | - | HB155 | - | - | - | HB20 | HB17 |
| DD2var03_1_74 | NTSA4 | - | - | - | HB155 | - | - | - | HB20 | HB17 |
| PFCLINvar68_1_75 | NTSA7 | - | - | - | HB155 | - | - | - | HB20 | HB17 |
| IGHvar27_1_71 | NTSA6 | - | - | - | HB155 | - | - | - | HB20 | HB17 |
| IGHvar23_1_75 | NTSA5 | - | - | - | HB155 | - | - | - | HB20 | HB17 |
| PFD0020c_1_76 | NTSA7 | - | - | - | HB155 | - | - | - | HB20 | HB17 |
| AAQ73927_1_74 | NTSA4 | - | - | - | HB155 | - | - | - | HB20 | HB17 |
| IT4var64_1_74 | NTSA5 | - | - | - | HB155 | - | - | - | HB20 | HB17 |
| RAJ116var17_1_75 | NTSA3 | - | - | - | HB155 | - | - | - | HB20 | HB17 |
| DD2var52_1_74 | NTSA5 | - | - | - | HB155 | - | - | - | HB20 | HB17 |
| PFCLINvar33_1_71 | NTSA6 | - | - | - | HB155 | - | - | - | HB20 | HB17 |
| PFCLINvar32_1_74 | NTSA3 | - | - | - | HB155 | - | - | - | HB20 | HB17 |
| PFCLINvar69_1_75 | NTSA5 | - | - | - | HB155 | - | - | - | HB20 | HB17 |
| HB3var04_1_74 | NTSA7 | - | - | - | HB155 | - | - | - | HB20 | HB17 |
| HB3var02_1_71 | NTSA2 | - | - | - | HB155 | - | - | - | HB20 | HB17 |
| IT4var18_1_74 | NTSA3 | - | - | - | HB155 | - | - | - | HB20 | HB17 |
| DD2var42_1_75 | NTSA5 | - | - | - | HB155 | - | - | - | HB20 | HB17 |
| IGHvar10_1_75 | NTSA2 | - | - | - | HB155 | - | - | - | HB20 | HB17 |
| IGHvar09_1_76 | NTSA3 | - | - | - | HB264 | - | - | - | HB20 | HB17 |
| PFCLINvar76_1_79 | NTSA1 | - | - | - | HB264 | - | - | - | HB20 | HB17 |
| IT4var35_1_76 | NTSA1 | - | - | - | HB264 | - | - | - | HB20 | HB17 |
| RAJ116var02_1_79 | NTSA1 | - | - | - | HB264 | - | - | - | HB20 | HB17 |
| PF13_0003_1_77 | NTSA3 | - | - | - | HB264 | - | - | - | HB20 | HB17 |
| IGHvar39_1_76 | NTSA7 | - | - | - | HB264 | - | - | - | HB20 | HB17 |
| RAJ116var38_1_72 | NTSA6 | - | - | - | HB264 | - | - | - | HB20 | HB17 |
| AJ420411_1_76 | NTSA1 | - | - | - | HB264 | - | - | - | HB20 | HB17 |
| PFD1225w_1_72 | NTSA2 | - | - | - | HB264 | - | - | - | HB20 | HB17 |
| HB3var1csa_1_77 | NTSA1 | - | - | - | HB264 | - | - | - | HB20 | HB17 |
| IGHvar11_1_76 | NTSA1 | - | - | - | HB264 | - | - | - | HB20 | HB17 |
| PFE1640w_1_78 | NTSA1 | - | - | - | HB264 | - | - | - | HB20 | HB17 |
| IT4var07_1_70 | NTSA6 | - | - | - | - | - | - | - | HB20 | HB17 |
| HB3var06_1_74 | NTSA2 | - | - | - | - | - | - | - | HB20 | HB17 |
| PF11_0521_1_70 | NTSA6 | - | - | - | - | - | - | - | HB20 | HB17 |
| RAJ116var16_1_69 | NTSA8 | - | - | - | - | - | - | - | HB20 | HB17 |
| IGHvar32_1_74 | NTSA3 | - | - | - | - | - | - | - | HB20 | HB17 |
| IT4var60_1_69 | NTSA7 | - | - | - | - | - | - | - | HB20 | HB17 |
| IGHvar22_1_68 | NTSA8 | - | - | - | - | - | - | - | HB20 | HB17 |
| DD2var22_1_74 | NTSA3 | - | - | - | - | - | - | - | HB20 | HB17 |
| PFCLINvar49_1_75 | NTSA7 | - | - | - | - | - | - | - | HB20 | HB17 |
| IGHvar24_1_68 | NTSA8 | - | - | - | - | - | - | - | HB20 | HB17 |
| HB3var01_1_75 | NTSA5 | - | - | - | - | - | - | - | HB20 | HB17 |
| DD2var32_1_70 | NTSA6 | - | - | - | - | - | - | - | HB20 | HB17 |
| AAQ73928_1_72 | NTSA6 | - | - | - | - | - | - | - | HB20 | HB17 |
| DD2var43_1_67 | NTSA3 | - | - | - | - | - | - | - | HB20 | HB17 |
| DD2var49_1_69 | NTSA8 | - | - | - | - | - | - | - | HB20 | HB17 |
| IT4var09_1_68 | NTSA8 | - | - | - | - | - | - | - | HB20 | HB17 |
| IGHvar26_1_77 | NTSA7 | - | - | - | - | - | - | - | HB20 | HB17 |
| IGHvar12_1_68 | NTSA8 | - | - | - | - | - | - | - | HB20 | HB17 |
| RAJ116var03_1_68 | NTSA8 | - | - | - | - | - | - | - | HB20 | HB17 |
| DD2var04_1_74 | NTSA5 | - | - | - | - | - | - | - | HB20 | HB17 |
| PFCLINvar23_1_62 | NTSB3 | - | - | - | HB216 | - | - | - | HB20 | HB17 |
| DD2var50_1_62 | NTSB3 | - | - | - | HB216 | - | - | - | HB20 | HB17 |
| PFI0005w_1_62 | NTSB3 | - | - | - | HB216 | - | - | - | HB20 | HB17 |
| IGHvar21_1_65 | NTSB3 | - | - | - | HB216 | - | - | - | HB20 | HB17 |
| IT4var44_1_62 | NTSB3 | - | - | - | HB216 | - | - | - | HB20 | HB17 |
| PFD0620c_1_60 | NTSB3 | - | - | - | HB216 | - | - | - | HB20 | HB17 |
| IGHvar37_1_62 | NTSB3 | - | - | - | HB216 | - | - | - | HB20 | HB17 |
| PFL0005w_1_69 | NTSB3 | - | - | - | HB216 | - | - | - | HB20 | HB17 |
| PFL1960w_1_62 | NTSB3 | - | - | - | HB216 | - | - | - | HB20 | HB17 |
| PFD0625c_1_63 | NTSB3 | - | - | - | HB216 | - | - | - | HB20 | HB17 |
| IGHvar36_1_62 | NTSB3 | - | - | - | HB216 | - | - | - | HB20 | HB17 |
| PFL0020w_1_75 | NTSB3 | - | - | - | HB216 | - | - | - | HB20 | HB17 |
| DD2var48_1_66 | NTSB3 | - | - | - | HB216 | - | - | - | HB20 | HB17 |
| DD2var30_1_64 | NTSB3 | - | - | - | HB216 | - | - | - | HB20 | HB17 |
| DD2var39_1_65 | NTSB3 | - | - | - | HB216 | - | - | - | HB20 | HB17 |
| TM284S2var1_1_66 | NTSB3 | - | - | - | HB216 | - | - | - | HB20 | HB17 |
| IT4var34_1_62 | NTSB3 | - | - | - | HB216 | - | - | - | HB20 | HB17 |
| PF08_0140_1_72 | NTSB5 | - | - | - | HB378 | - | HB29 | - | HB20 | HB17 |
| RAJ116var11_1_73 | NTSB5 | - | - | - | HB378 | - | HB29 | - | HB20 | HB17 |
| DD2var15_1_67 | NTSB2 | - | - | - | HB378 | - | - | - | HB20 | HB17 |
| PFCLINvar63_1_67 | NTSB2 | - | - | - | HB378 | - | - | - | HB20 | HB17 |
| HB3var22_1_69 | NTSB3 | - | - | - | HB378 | - | - | - | HB20 | HB17 |
| PFC1120c_1_67 | NTSB3 | - | - | - | HB378 | - | - | - | HB20 | HB17 |
| IT4var28_1_67 | NTSB2 | - | - | - | HB378 | - | - | - | HB20 | HB17 |
| HB3var36_1_67 | NTSB2 | - | - | - | HB378 | - | - | - | HB20 | HB17 |
| PFCLINvar66_1_58 | NTSB3 | - | - | - | HB525 | - | - | - | HB20 | HB17 |
| IGHvar34_1_57 | NTSB3 | - | - | - | HB525 | - | - | - | HB20 | HB17 |
| IT4var20_1_57 | NTSB3 | - | - | - | HB525 | - | - | - | HB20 | HB17 |
| IT4var13_1_58 | NTSB3 | - | - | - | HB525 | - | - | - | HB20 | HB17 |
| DD2var21_1_58 | NTSB3 | - | - | - | HB525 | - | - | - | HB20 | HB17 |
| DD2var47_1_57 | NTSB3 | - | - | - | HB525 | - | - | - | HB20 | HB17 |
| RAJ116var26_1_65 | NTSB3 | - | - | - | HB564 | - | - | - | HB20 | HB17 |
| IT4var39_1_65 | NTSB3 | - | - | - | HB564 | - | - | - | HB20 | HB17 |
| DD2var11_1_65 | NTSB3 | - | - | - | HB564 | - | - | - | HB20 | HB17 |
| PFCLINvar54_1_65 | NTSB3 | - | - | - | HB564 | - | - | - | HB20 | HB17 |
| PFA0765c_1_63 | NTSB3 | - | - | - | HB564 | - | - | - | HB20 | HB17 |
| HB3var26_1_65 | NTSB3 | - | - | - | - | - | - | HB599 | HB20 | HB17 |
| PFCLINvar28_1_67 | NTSB7 | - | - | - | - | - | - | HB599 | HB20 | HB17 |
| IT4var63_1_66 | NTSB3 | - | - | - | - | - | - | HB599 | HB20 | HB17 |
| DD2var44_1_66 | NTSB3 | - | - | - | - | - | - | HB599 | HB20 | HB17 |
| IGHvar15_1_66 | NTSB3 | - | - | - | - | - | - | HB599 | HB20 | HB17 |
| PFL1950w_1_68 | NTSB3 | - | - | - | - | - | - | - | HB20 | HB17 |
| RAJ116var22_1_67 | NTSB3 | - | - | - | - | - | - | - | HB20 | HB17 |
| PFCLINvar60_1_61 | NTSB1 | - | - | - | - | - | - | - | HB20 | HB17 |
| PFCLINvar61_1_60 | NTSB6 | - | - | - | - | - | - | - | HB20 | HB17 |
| PFI1830c_1_65 | NTSB3 | - | - | - | - | - | - | - | HB20 | HB17 |
| PF08_0142_1_67 | NTSB3 | - | - | - | - | - | - | - | HB20 | HB17 |
| MAL6P1.316_1_59 | NTSB3 | - | - | - | - | - | - | - | HB20 | HB17 |
| AY028643_1_63 | NTSB3 | - | - | - | - | - | - | - | HB20 | HB17 |
| IGHvar29_1_63 | NTSB3 | - | - | - | - | - | - | - | HB20 | HB17 |
| IGHvar28_1_58 | NTSB3 | - | - | - | - | - | - | - | HB20 | HB17 |
| PFCLINvar53_1_56 | NTSB4 | - | - | - | - | - | - | - | HB20 | HB17 |
| RAJ116var35_2_61 | NTSB6 | - | - | - | - | - | - | - | HB20 | HB17 |
| IT4var11_1_56 | NTSB3 | - | - | - | - | - | - | - | HB20 | HB17 |
| RAJ116var32_1_57 | NTSB3 | - | - | - | - | - | - | - | HB20 | HB17 |
| DD2var36_1_56 | NTSB1 | - | - | - | - | - | - | - | HB20 | HB17 |
| PFCLINvar45_1_59 | NTSB4 | - | - | - | - | - | - | - | HB20 | HB17 |
| PFD0615c_1_62 | NTSB3 | - | - | - | - | - | - | - | HB20 | HB17 |
| IT4var05_1_63 | NTSB3 | - | - | - | - | - | - | - | HB20 | HB17 |
| PFD1245c_1_62 | NTSB3 | - | - | - | - | - | - | - | HB20 | HB17 |
| RAJ116var33_1_61 | NTSB6 | - | - | - | - | - | - | - | HB20 | HB17 |
| HB3var48_1_60 | NTSB3 | - | - | - | - | - | - | - | HB20 | HB17 |
| PFCLINvar31_1_69 | NTSB5 | - | - | - | - | - | - | - | HB20 | HB17 |
| PF08_0103_1_62 | NTSB3 | - | - | - | - | - | - | - | HB20 | HB17 |
| IGHvar35_1_66 | NTSB3 | - | - | - | - | - | - | - | HB20 | HB17 |
| IGHvar08_1_68 | NTSB3 | - | - | - | - | - | - | - | HB20 | HB17 |
| HB3var07_1_62 | NTSB3 | - | - | - | - | - | - | - | HB20 | HB17 |
| RAJ116var06_1_64 | NTSB3 | - | - | - | - | - | - | - | HB20 | HB17 |
| PFB0010w_1_64 | NTSB3 | - | - | - | - | - | - | - | HB20 | HB17 |
| RAJ116var09_1_60 | NTSB3 | - | - | - | - | - | - | - | HB20 | HB17 |
| HB3var30_1_55 | NTSB3 | - | - | - | - | - | - | - | HB20 | HB17 |
| PREICHvar95_1_63 | NTSB5 | - | - | - | - | - | - | - | HB20 | HB17 |
| HB3var18_1_65 | NTSB3 | - | - | - | - | - | - | - | HB20 | HB17 |
| PFC0005w_1_67 | NTSB3 | - | - | - | - | - | - | - | HB20 | HB17 |
| DD2var16_1_65 | NTSB7 | - | - | - | - | - | - | - | HB20 | HB17 |
| IT4var67_1_67 | NTSB3 | - | - | - | - | - | - | - | HB20 | HB17 |
| RAJ116var14_1_63 | NTSB3 | - | - | - | - | - | - | - | HB20 | HB17 |
| PF08_0107_1_64 | NTSB3 | - | - | - | - | - | - | - | HB20 | HB17 |
| RAJ116var34_1_60 | NTSB3 | - | - | - | - | - | - | - | HB20 | HB17 |
| PFD1005c_1_67 | NTSB3 | - | - | - | - | - | - | - | HB20 | HB17 |
| DD2var45_1_67 | NTSB3 | - | - | - | - | - | - | - | HB20 | HB17 |
| PFCLINvar74_1_58 | NTSB3 | - | - | - | - | - | - | - | HB20 | HB17 |
| PREICHvar93_1_58 | NTSB3 | - | - | - | - | - | - | - | HB20 | HB17 |
| IT4var51_1_72 | NTSB3 | - | - | - | - | - | - | - | HB20 | HB17 |
| IGHvar18_1_58 | NTSB3 | - | - | - | - | - | - | - | HB20 | HB17 |
| HB3var47_1_64 | NTSB3 | - | - | - | - | - | - | - | HB20 | HB17 |
| DD2var20_1_64 | NTSB3 | - | - | - | - | - | - | - | HB20 | HB17 |
| HB3var20_1_66 | NTSB3 | - | - | - | - | - | - | - | HB20 | HB17 |
| IT4var29_1_56 | NTSB3 | - | - | - | - | - | - | - | HB20 | HB17 |
| PF13_0001_1_59 | NTSB3 | - | - | - | - | - | - | - | HB20 | HB17 |
| IGHvar16_1_66 | NTSB3 | - | - | - | - | - | - | - | HB20 | HB17 |
| IGHvar13_1_63 | NTSB3 | - | - | - | - | - | - | - | HB20 | HB17 |
| HB3var25_1_82 | NTSB3 | - | - | - | - | - | - | - | HB20 | HB17 |
| PF13_0364_1_61 | NTSB3 | - | - | - | - | - | - | - | HB20 | HB17 |
| IGHvar02_1_62 | NTSB3 | - | - | - | - | - | - | - | HB20 | HB17 |
| PFCLINvar52_1_61 | NTSB3 | - | - | - | - | - | - | - | HB20 | HB17 |
| IT4var01_1_60 | NTSB6 | - | - | - | - | - | - | - | HB20 | HB17 |
| IT4var41_1_61 | NTSB3 | - | - | - | - | - | - | - | HB20 | HB17 |
| PFCLINvar30_1_60 | NTSB1 | - | - | - | - | - | - | - | HB20 | HB17 |
| IGHvar38_1_59 | NTSB3 | - | - | - | - | - | - | - | HB20 | HB17 |
| DD2var38_1_65 | NTSB4 | - | - | - | - | - | - | HB120 | HB20 | HB17 |
| PFCLINvar15_1_65 | NTSB3 | - | - | - | - | - | - | - | HB20 | HB17 |
| PFB1055c_1_60 | NTSB3 | - | - | - | - | - | - | - | HB20 | HB17 |
| IGHvar20_1_64 | NTSB2 | - | - | - | - | - | - | - | HB20 | HB17 |
| PFCLINvar67_1_65 | NTSB3 | - | - | - | - | - | - | - | HB20 | HB17 |
| MAL6P1.252_1_61 | NTSB1 | - | - | - | - | - | - | - | HB20 | HB17 |
| AAB60251_1_61 | NTSB3 | - | - | - | - | - | - | - | HB20 | HB17 |
| RAJ116var08_1_61 | NTSB3 | - | - | - | - | - | - | - | HB20 | HB17 |
| PF08_0106_1_59 | NTSB3 | - | - | - | - | - | - | - | HB20 | HB17 |
| PF10_0406_1_67 | NTSB3 | - | - | - | - | - | - | - | HB20 | HB17 |
| HB3var23_1_68 | NTSB3 | - | - | - | - | - | - | - | HB20 | HB17 |
| HB3var12_1_63 | NTSB3 | - | - | - | - | - | - | - | HB20 | HB17 |
| PFCLINvar44_1_58 | NTSB3 | - | - | - | - | - | - | - | HB20 | HB17 |
| PFCLINvar40_1_67 | NTSB3 | - | - | - | - | - | - | - | HB20 | HB17 |
| RAJ116var10_1_66 | NTSB3 | - | - | - | - | - | - | - | HB20 | HB17 |
| PFL1955w_1_60 | NTSB3 | - | - | - | - | - | - | - | HB20 | HB17 |
| IGHvar04_1_58 | NTSB3 | - | - | - | - | - | - | - | HB20 | HB17 |
| IT4var17_1_64 | NTSB3 | - | - | - | - | - | - | - | HB20 | HB17 |
| IT4var31_1_56 | NTSB3 | - | - | - | - | - | - | - | HB20 | HB17 |
| PFCLINvar43_1_65 | NTSB3 | - | - | - | - | - | - | - | HB20 | HB17 |
| PFCLINvar46_1_90 | NTSB3 | - | - | - | - | - | - | - | HB20 | HB17 |
| MAL7P1.56_1_70 | NTSB3 | - | - | - | - | - | - | - | HB20 | HB17 |
| DD2var31_1_58 | NTSB3 | - | - | - | - | - | - | - | HB20 | HB17 |
| RAJ116var37_1_67 | NTSB7 | - | - | - | - | - | - | - | HB20 | HB17 |
| AAC05220_1_63 | NTSB3 | - | - | - | - | - | - | - | HB20 | HB17 |
| RAJ116var27_1_54 | NTSB3 | - | - | - | - | - | - | - | HB20 | HB17 |
| DD2var29_1_59 | NTSB3 | - | - | - | - | - | - | - | HB20 | HB17 |
| IGHvar06_1_62 | NTSB3 | - | - | - | - | - | - | - | HB20 | HB17 |
| PFL0935c_1_59 | NTSB3 | - | - | - | - | - | - | - | HB20 | HB17 |
| HB3var21_1_60 | NTSB4 | - | - | - | - | - | - | - | HB20 | HB17 |
| IT4var33_1_57 | NTSB3 | - | - | - | - | - | - | - | HB20 | HB17 |
| IGHvar17_1_65 | NTSB3 | - | - | - | - | - | - | - | HB20 | HB17 |
| IT4var16_1_64 | NTSB3 | - | - | - | - | - | - | - | HB20 | HB17 |
| DD2var34_1_68 | NTSB3 | - | - | - | - | - | - | - | HB20 | HB17 |
| IT4var27_1_65 | NTSB3 | - | - | - | - | - | - | - | HB20 | HB17 |
| DD2var46_1_58 | NTSB3 | - | - | - | - | - | - | - | HB20 | HB17 |
| RAJ116var29_1_64 | NTSB3 | - | - | - | - | - | - | - | HB20 | HB17 |
| PFCLINvar36_1_58 | NTSB3 | - | - | - | - | - | - | - | HB20 | HB17 |
| RAJ116var13_1_68 | NTSB3 | - | - | - | - | - | - | - | HB20 | HB17 |
| HB3var17_1_64 | NTSB4 | - | - | - | - | - | - | - | HB20 | HB17 |
| PFE0005w_1_59 | NTSB3 | - | - | - | - | - | - | - | HB20 | HB17 |
| DD2var19_1_75 | NTSB3 | - | - | HB217 | - | - | - | - | HB20 | HB17 |
| MAL6P1.4_1_64 | NTSB3 | - | - | HB217 | - | - | - | - | HB20 | HB17 |
| PFCLINvar64_1_68 | NTSB3 | - | - | HB217 | - | - | - | - | HB20 | HB17 |
| IT4var24_1_71 | NTSB3 | - | - | HB217 | - | - | - | - | HB20 | HB17 |
| PFL2665c_1_66 | NTSB3 | - | - | HB217 | - | - | - | - | HB20 | HB17 |
| IT4var58_1_60 | NTSB3 | - | - | HB217 | - | - | - | - | HB20 | HB17 |
| IT4var61_1_70 | NTSB3 | - | - | HB217 | - | - | - | - | HB20 | HB17 |
| IT4var66_1_68 | NTSB3 | - | - | HB217 | - | - | - | - | HB20 | HB17 |
| HB3var24_1_71 | NTSB3 | - | - | HB217 | - | - | - | - | HB20 | HB17 |
| IGHvar01_1_69 | NTSB3 | - | - | HB217 | - | - | - | - | HB20 | HB17 |
| RAJ116var04_1_72 | NTSB3 | - | - | HB217 | - | - | - | - | HB20 | HB17 |
| PF07_0048_1_62 | NTSB3 | - | - | HB217 | - | - | HB71 | - | HB20 | HB17 |
| PFCLINvar57_1_62 | NTSB3 | - | - | HB217 | - | - | HB71 | - | HB20 | HB17 |
| PF07_0139_1_70 | NTSB3 | - | - | HB217 | - | - | HB71 | - | HB20 | HB17 |
| MAL7P1.55_1_67 | NTSB3 | - | - | HB217 | - | - | HB71 | - | HB20 | HB17 |
| HB3var28_1_69 | NTSB3 | - | HB194 | HB217 | - | - | HB71 | - | HB20 | HB17 |
| DD2var41_1_67 | NTSB3 | - | HB194 | HB217 | - | - | HB71 | - | HB20 | HB17 |
| IT4var26_1_69 | NTSB3 | - | HB194 | - | - | - | HB71 | - | HB20 | HB17 |
| PREICHvar90_1_75 | NTSB3 | - | HB194 | - | - | - | HB71 | - | HB20 | HB17 |
| HB3var34_1_57 | NTSB3 | - | HB194 | - | - | - | HB71 | - | HB20 | HB17 |
| PFCLINvar58_1_74 | NTSB3 | - | HB194 | - | - | - | HB71 | - | HB20 | HB17 |
| PFCLINvar56_1_69 | NTSB3 | - | HB194 | - | - | - | HB71 | - | HB20 | HB17 |
| PF10_0001_1_65 | NTSB3 | - | HB194 | - | - | - | HB71 | - | HB20 | HB17 |
| PFA0005w_1_67 | NTSB3 | - | HB194 | - | - | - | HB71 | - | HB20 | HB17 |
| DD2var12_1_65 | NTSB3 | - | HB194 | - | - | - | HB71 | - | HB20 | HB17 |
| DD2var10_1_69 | NTSB3 | - | HB194 | - | - | - | - | - | HB20 | HB17 |
| PFCLINvar71_1_60 | NTSB3 | - | HB194 | - | - | - | - | - | HB20 | HB17 |
| IGHvar33_1_63 | NTSB3 | - | HB194 | - | - | - | - | - | HB20 | HB17 |
| DD2var24_1_70 | NTSB3 | - | HB194 | - | - | - | - | - | HB20 | HB17 |
| PF07_0049_1_63 | NTSB3 | - | HB194 | - | - | - | - | - | HB20 | HB17 |
| PFD1015c_1_64 | NTSB3 | - | HB194 | - | - | - | - | - | HB20 | HB17 |
| PFCLINvar24_1_61 | NTSB3 | - | HB194 | - | - | - | - | - | HB20 | HB17 |
| PFCLINvar11_1_63 | NTSB3 | - | HB194 | - | - | - | - | - | HB20 | HB17 |
| IT4var25_1_62 | NTSB3 | HB323 | - | - | - | - | - | - | HB20 | HB17 |
| IT4var47_1_61 | NTSB3 | HB323 | - | - | - | - | - | - | HB20 | HB17 |
| IT4var54_1_59 | NTSB3 | HB323 | - | - | - | - | - | - | HB20 | HB17 |
| IT4var22_1_62 | NTSB3 | HB323 | - | - | - | - | - | - | HB20 | HB17 |
| HB3var09_1_65 | NTSB3 | HB323 | - | - | - | - | - | - | HB20 | HB17 |
| MAL7P1.50_1_60 | NTSB3 | HB323 | - | - | - | - | - | - | HB20 | HB17 |
| IGHvar03_1_63 | NTSB3 | HB323 | HB194 | - | - | - | - | - | HB20 | HB17 |
| PFD0625c_1_62 | NTSB3 | HB323 | HB194 | - | - | - | - | - | HB20 | HB17 |
| RAJ116var15_1_76 | NTSB3 | HB323 | - | - | - | - | HB71 | - | HB20 | HB17 |
| MAL6P1.1_1_66 | NTSB3 | HB323 | - | - | - | - | HB71 | - | HB20 | HB17 |
| DD2var33_1_66 | NTSB3 | - | - | - | HB439 | - | - | - | HB20 | HB17 |
| IT4var46_1_66 | NTSB3 | - | - | - | HB439 | - | - | - | HB20 | HB17 |
| HB3var08_1_67 | NTSB3 | - | - | - | HB439 | - | - | - | HB20 | HB17 |
| IGHvar40_1_62 | NTSB3 | - | - | - | HB439 | - | - | - | HB20 | HB17 |
| MAL8P1.220_1_61 | NTSB3 | - | - | - | HB439 | - | - | - | HB20 | HB17 |
| DD2var28_1_75 | NTSB3 | - | - | - | HB439 | - | HB71 | - | HB20 | HB17 |
| HB3var10_1_70 | NTSB3 | - | - | - | HB439 | - | HB71 | - | HB20 | HB17 |
| DD2var13_1_68 | NTSB3 | - | - | - | - | - | HB71 | - | HB20 | HB17 |
| PF07_0050_1_56 | NTSB3 | - | - | - | - | - | HB71 | - | HB20 | HB17 |
| RAJ116var31_1_65 | NTSB3 | - | - | - | - | - | HB71 | - | HB20 | HB17 |
| RAJ116var30_1_82 | NTSB3 | - | - | - | HB71 | HB125 | HB71 | - | HB20 | HB17 |
| DD2var23_1_68 | NTSB3 | - | - | - | - | - | HB71 | - | HB20 | HB17 |
| HB3var29_1_74 | NTSB3 | - | - | - | - | - | HB71 | - | HB20 | HB17 |
| PFCLINvar55_1_70 | NTSB3 | - | - | - | - | - | HB71 | - | HB20 | HB17 |
| DD2var07_1_80 | NTSB3 | - | - | - | - | - | HB71 | - | HB20 | HB17 |
| RAJ116var01_1_66 | NTSB3 | - | - | - | - | - | HB71 | - | HB20 | HB17 |
| IGHvar25_1_73 | NTSB3 | - | - | - | - | - | HB71 | - | HB20 | HB17 |
| IT4var14_1_66 | NTSB3 | - | - | - | - | - | HB71 | - | HB20 | HB17 |
| IT4var62_1_69 | NTSB3 | - | - | - | - | - | HB71 | - | HB20 | HB17 |
| HB3var11_1_71 | NTSB3 | - | - | - | - | - | HB71 | - | HB20 | HB17 |
| IT4var06_1_132 | NTSB3 | HB71 | HB71 | HB190 | HB71 | - | HB71 | - | HB20 | HB17 |
| PFCLINvar06_1_60 | NTSB3 | - | - | - | - | - | HB71 | - | HB20 | HB17 |
| RAJ116var21_1_75 | NTSB3 | - | - | - | - | - | HB71 | - | HB20 | HB17 |
| HB3var50_1_67 | NTSB3 | - | - | - | - | - | HB71 | - | HB20 | HB17 |
| DD2var51_1_70 | NTSB3 | - | - | - | - | - | HB71 | - | HB20 | HB17 |
| IT4var32a_1_66 | NTSB3 | - | - | - | - | - | HB71 | - | HB20 | HB17 |
| RAJ116var36_1_67 | NTSB3 | - | - | - | - | - | HB71 | - | HB20 | HB17 |
| PFCLINvar41_1_67 | NTSB3 | - | - | - | - | - | HB71 | - | HB20 | HB17 |
| RAJ116var23_1_66 | NTSB3 | - | - | - | - | - | HB71 | - | HB20 | HB17 |
| RAJ116var39_1_78 | NTSB3 | - | - | - | - | - | HB71 | HB357 | HB20 | HB17 |
| IGHvar07_1_70 | NTSB3 | - | - | - | - | - | HB71 | - | HB20 | HB17 |
| PF11_0007_1_67 | NTSB3 | - | - | - | - | - | HB71 | - | HB20 | HB17 |
| HB3var40_1_72 | NTSB3 | - | - | - | - | - | HB71 | - | HB20 | HB17 |
| HB3var33_1_64 | NTSB3 | - | - | - | - | - | HB71 | - | HB20 | HB17 |
| HB3var14_1_72 | NTSB3 | - | - | - | - | - | HB71 | - | HB20 | HB17 |
| RAJ116var28_1_66 | NTSB3 | - | - | - | - | - | HB71 | - | HB20 | HB17 |
| HB3var31_1_73 | NTSB3 | - | - | - | - | - | HB71 | - | HB20 | HB17 |
| HB3var19_1_68 | NTSB3 | - | - | - | - | - | HB71 | - | HB20 | HB17 |
| IGHvar31_1_66 | NTSB3 | - | - | - | - | - | HB71 | - | HB20 | HB17 |
| MAL7P1.212_1_65 | NTSB3 | - | - | - | - | - | HB71 | - | HB20 | HB17 |
| HB3var16_1_64 | NTSB3 | - | - | - | - | - | HB71 | - | HB20 | HB17 |
| PFCLINvar65_1_75 | NTSB3 | - | - | - | - | - | HB71 | - | HB20 | HB17 |
| PFD0005w_1_77 | NTSB3 | - | - | - | - | - | HB71 | - | HB20 | HB17 |
| IT4var15_1_68 | NTSB3 | - | - | - | - | - | HB71 | - | HB20 | HB17 |
| PFD0995c_1_66 | NTSB3 | - | - | - | - | - | HB71 | - | HB20 | HB17 |
| PF07_0051_1_65 | NTSB3 | - | - | - | - | - | HB71 | - | HB20 | HB17 |
| RAJ116var18_1_67 | NTSB3 | - | - | - | - | - | HB71 | - | HB20 | HB17 |
| IGHvar05_1_64 | NTSB3 | - | - | - | - | - | HB71 | - | HB20 | HB17 |
| PFCLINvar47_1_65 | NTSB3 | - | - | - | - | - | HB71 | - | HB20 | HB17 |
| RAJ116var24_1_66 | NTSB3 | - | - | - | - | - | HB71 | - | HB20 | HB17 |
| PFD1000c_1_66 | NTSB3 | - | - | - | - | - | HB71 | - | HB20 | HB17 |
| DD2var37_1_57 | NTSB3 | - | - | - | - | - | HB71 | - | HB20 | HB17 |
| HB3var27_1_75 | NTSB3 | - | - | - | - | - | HB71 | - | HB20 | HB17 |
| IGHvar19_1_73 | NTSB3 | - | - | - | - | - | HB71 | - | HB20 | HB17 |
| DD2var35_1_49 | NTSB3 | - | - | - | - | - | HB71 | - | HB20 | - |
| IT4var45_1_43 | NTSB3 | - | - | - | - | - | - | - | - | HB17 |
| PFL0030c_1_50 | NTSpam | - | - | - | - | - | - | - | HB309 | HB65 |
| RAJ116var25_1_51 | NTSpam | - | - | - | - | - | - | - | HB309 | HB65 |
| PFCLINvar72_1_50 | NTSpam | - | - | - | - | - | - | - | HB309 | HB65 |
| DD2var06_1_50 | NTSpam | - | - | - | - | - | - | - | HB309 | HB65 |
| AAQ73930_1_51 | NTSpam | - | - | - | - | - | - | - | HB309 | HB65 |
| IGHvar41_1_51 | NTSpam | - | - | - | - | - | - | - | HB309 | HB65 |
| HB3var2csaA_1_51 | NTSpam | - | - | - | - | - | - | - | HB309 | HB65 |
| HB3var2csaB_1_51 | NTSpam | - | - | - | - | - | - | - | HB309 | HB65 |
| PFCLINvar35_1_51 | NTSpam | - | - | - | - | - | - | - | HB309 | HB65 |
| IT4var04_1_51 | NTSpam | - | - | - | - | - | - | - | HB309 | HB65 |
